# The logarithmic contributions to the $O(\alpha _s^3)$ asymptotic massive Wilson coefficients and operator matrix elements in deeply inelastic scattering

**DOI:** 10.1140/epjc/s10052-014-3033-x

**Published:** 2014-09-16

**Authors:** A. Behring, I. Bierenbaum, J. Blümlein, A. De Freitas, S. Klein, F. Wißbrock

**Affiliations:** 1 Deutsches Elektronen Synchrotron, DESY, Platanenallee 6, 15738 Zeuthen, Germany; 2 II. Institut für Theoretische Physik, Universität Hamburg, Luruper Chaussee 149, 22761 Hamburg, Germany; 3 Institut für Theoretische Teilchenphysik und Kosmologie, RWTH Aachen University, 52056 Aachen, Germany; 4 Research Institute for Symbolic Computation (RISC), Johannes Kepler University, Altenbergerstraße 69, 4040 Linz, Austria; 5 Present Address: IHES, 35 Route de Chartres, 91440 Bures-sur-Yvette, France

## Abstract

We calculate the logarithmic contributions to the massive Wilson coefficients for deep-inelastic scattering in the asymptotic region $Q^2 \gg m^2$ to 3-loop order in the fixed flavor number scheme and present the corresponding expressions for the massive operator matrix elements needed in the variable flavor number scheme. Explicit expressions are given in Mellin $N$-space.

## Introduction

The heavy flavor corrections to deep-inelastic structure functions amount to sizeable contributions, in particular in the region of small values of the Bjorken variable $x$. Starting from lower values of the virtuality, over a rather wide kinematic range, their scaling violations are very different from those of the massless contributions. Currently, the heavy flavor corrections are known in semi-analytic form to 2-loop (NLO) order [1–3].^1^ ${}^{,}$ 2 The present accuracy of the deep-inelastic data reaches the order of 1 % [9]. It therefore requires the next-to-next-to-leading order (NNLO) corrections for precision determinations of both the strong coupling constant $\alpha _s(M_Z^2)$ [10] and the parton distribution functions (PDFs) [11–21], as well as the detailed understanding of the heavy flavor production cross sections in lepton–nucleon scattering [22–29]. The precise knowledge of these quantities is also of central importance for the interpretation of the physics results at the Large Hadron Collider, LHC, [30–32], and a high-luminosity facility studying deep-inelastic scattering as the EIC [33, 34].

In the kinematic region at HERA, where the twist-2 contributions to the deep-inelastic scattering (DIS) dominate, cf. [35],^3^, i.e., $Q^2/m^2 \gtrsim 10$, with $m = m_c$ the charm-quark mass, it has been proven in Ref. [37] that the heavy flavor Wilson coefficients factorize into massive operator matrix elements (OMEs) and the massless Wilson coefficients. The massless Wilson coefficients for the structure function $F_2(x,Q^2)$ are known to 3-loop order [38]. In the region $Q^2 \gg m^2$, where $Q^2=-q^2$, with $q$ the space-like 4-momentum transfer and $m$ the heavy quark mass, the power corrections $O((m^2/Q^2)^k), k \ge 1$ to the heavy quark structure functions become very small.

In Ref. [39], a series of fixed Mellin moments $N$ up to $N = 10,\ldots ,14$, depending on the respective transition, has been calculated for all the OMEs at 3-loop order.^4^ Also the moments of the transition coefficients needed in the variable flavor scheme (VFNS) have been calculated. Here, the massive OMEs for given total spin $N$ were mapped onto massive tadpoles which have been computed using MATAD [41].

In the present paper, we calculate the logarithmic contributions to a series of unpolarized massive Wilson coefficients in the asymptotic region $Q^2 \gg m^2$ to 3-loop order and the massive OMEs needed in the VFNS. These include the logarithmic terms $\log (Q^2/m^2)$ and those parts of the constant term, which are obtained through renormalization, cf. [39]. Since the results for the OMEs $A_{gq}^{(3)}$ and the flavor non-singlet Wilson coefficients for the structure function $F_2(x,Q^2)$ have been calculated completely in the meantime and have been published in [42, 43], the corresponding expressions are not presented here. Results given in [36] will be discussed in Sect. 4. In the following, we set the factorization and renormalization scales equal $\mu _F = \mu _R \equiv \mu$ and exhibit the $\log (m^2/\mu ^2)$ dependence on the Wilson coefficients, besides their dependence on the virtuality $Q^2$. The logarithmic contributions are determined by the lower order massive OMEs [44–49], the mass and coupling constant renormalization constants, and the anomalous dimensions [50–52], as has been worked out in Ref. [39]. For the structure function $F_L(x,Q^2)$, the asymptotic heavy flavor Wilson coefficients at $O(\alpha _s^3)$ for tagged heavy flavor were calculated in [53]. Here, we present the result considering the *inclusive* final state for this structure function. In both cases, the asymptotic representation becomes effective only at much higher scales of $Q^2$ [37] if compared to those for $F_2(x,Q^2)$, however. We first choose the fixed flavor number scheme to express the heavy flavor contributions to the structure functions $F_2(x,Q^2)$ and $F_L(x,Q^2)$. This scheme has to be considered as the genuine scheme in quantum field theoretic calculations since the initial states, the twist-2 *massless* partons, can, at least to a good approximation, be considered as LSZ-states. The representations in the VFNS can be obtained using the respective transition coefficients within the appropriate regions, where one single heavy quark flavor becomes effectively massless. Here, appropriate matching scales have to be applied, which vary depending on the observable considered, cf. [54].

The constant contributions of the un-renormalized OMEs have been calculated in two cases $A_{qq,Q}^{\mathsf{PS}}(N)$ and $A_{qg,Q}(N)$, in Ref. [55]. Here, we present both the renormalized OMEs and the corresponding massive Wilson coefficients, which contribute first at 2- and 3-loop order. We also derive numerical results for the Wilson coefficients. The quantities being presented in the present paper derive from OMEs which were computed in terms of generalized hypergeometric functions [56, 57] and sums thereof, prior to the expansion in the dimensional variable $\varepsilon = D -4$, cf. [58–62]. Finally, they are represented in terms of nested sums over products of hypergeometric terms and harmonic sums, which can be calculated using modern summation techniques [63–76]. They are based on a refined difference field of [77] and generalize the summation paradigms presented in [78] to multi-summation. The results of this computation can be expressed in terms of nested harmonic sums [79, 80]. The results in Mellin $N$-space can be continued to complex values of $N$ as has been described in Refs. [58–60, 81–84].

It is the aim of the present paper to provide a detailed documentation of formulae in $N$-space for all logarithmic contributions and the terms implied by renormalization contributing to the constant part of the heavy flavor Wilson coefficients of the structure functions $F_2(x,Q^2)$ and $F_L(x,Q^2)$ and the massive OMEs needed in the variable flavor number scheme up to $O(\alpha _s^3)$ and to compare to results in the literature. Here, we refer to a minimal representation, i.e., we use all the algebraic relations between the harmonic sums and the harmonic polylogarithms, respectively, leading to a minimal number of basic functions. Based on the known Mellin moments [39] we also perform numerical comparisons between the different contributions to the Wilson coefficients and massive OMEs at $O(\alpha _s^3)$ referring to the parton distributions [21].

The paper is organized as follows. In Sect. 2, we briefly summarize the basic formalism. The Wilson coefficients $L_{q,2}^{\mathsf{PS}}$ and $L_{g,2}^{\mathsf{S}}$ are discussed in Sect. 3. As they are known in complete form we also present numerical results. In Sect. 4, the logarithmic contributions to the Wilson coefficients $H_{g,2}^{\mathsf{S}}$ and $H_{q,2}^{\mathsf{PS}}$ are derived and discussed. The corresponding inclusive Wilson coefficients for the longitudinal structure function $F_L(x,Q^2)$ in the asymptotic region are presented in Sect. 5. In Sect. 6, we compare the different loop contributions to the massive Wilson coefficients and OMEs for a series of Mellin moments in terms of the dependence on the virtuality $Q^2$. Section 7 contains the conclusions. In Appendix A, the massive OMEs dealt with in the present paper which are needed in the VFNS are given in Mellin $N$-space.

## The heavy flavor Wilson coefficients in the asymptotic region

We consider the heavy flavor contributions to the inclusive unpolarized structure functions $F_2(x,Q^2)$ and $F_L(x,Q^2)$ in deep-inelastic scattering, cf. [85, 86], in case of single electro-weak gauge boson exchange at large virtualities $Q^2$. At higher orders in the strong coupling constant these corrections receive both contributions from massive and massless partons in the hadronic final state, which is summed over completely. In the latter case, the heavy flavor corrections are also due to virtual contributions. We consider the situation in which the contributions to the twist-2 operators dominate in the Bjorken limit. Here, transverse momentum effects of the initial state do not contribute. In the present paper, we consider only heavy flavor contributions due to $N_F$ massless and one massive flavor of mass $m$.^5^ The Wilson coefficients are calculable perturbatively and are denoted by

1 $$\begin{aligned} {\mathcal C}^{\mathsf{S},\mathsf{PS},\mathsf{NS}}_{i, (2,L)} \left( x,N_F+1,\frac{Q^2}{\mu ^2},\frac{m^2}{\mu ^2}\right) . \end{aligned}$$

Here, $x$ denotes the Bjorken variable, the index $i$ refers to the respective initial state on-shell parton $i=q,g$ being a quark or gluon, and $\mathsf{S},\mathsf{PS},\mathsf{NS}$ label the flavor singlet, pure-singlet and non-singlet contributions, respectively.

The massless flavor contributions in (1) may be identified and separated in the Wilson coefficients into a purely light part $C_{i,(2,L)}$, and a heavy part by:

2 $$\begin{aligned}&\!\!\!{\mathcal C}^{\mathsf{S},\mathsf{PS},\mathsf{NS}}_{i,(2,L)} \left( x,N_F+1,\frac{Q^2}{\mu ^2},\frac{m^2}{\mu ^2}\right) \nonumber \\&\!\!\!\quad = C_{i,(2,L)}^\mathsf{S,PS,NS}\left( x,N_F,\frac{Q^2}{\mu ^2}\right) \nonumber \\&\!\!\!\qquad + H_{i,(2,L)}^{\mathsf{S}, \mathsf{PS}} \left( x,N_F+1,\frac{Q^2}{\mu ^2},\frac{m^2}{\mu ^2}\right) \nonumber \\&\!\!\!\qquad + L_{i,(2,L)}^{\mathsf{S},\mathsf{PS},\mathsf{NS}} \left( x,N_F+1,\frac{Q^2}{\mu ^2},\frac{m^2}{\mu ^2}\right) . \end{aligned}$$

The heavy flavor Wilson coefficients are defined by $L_{i,j}$ and $H_{i,j}$, depending on whether the exchanged electro-weak gauge boson couples to a light $(L)$ or heavy $(H)$ quark line. From this it follows that the light flavor Wilson coefficients $C_{i,j}$ depend on $N_F$ light flavors only, whereas $H_{i,j}$ and $L_{i,j}$ may contain light flavors in addition to the heavy quark, indicated by the argument $N_F+1$. $L_{q,(2,L)}^{\mathsf{S}}$ can be split into the flavor non-singlet and pure-singlet contributions

3 $$\begin{aligned} L_{q,(2,L)}^{\mathsf{S}}=L_{q,(2,L)}^{\mathsf{NS}}+L_{q,(2,L)}^{\mathsf{PS}}, \end{aligned}$$

and at $O(a_s^2)$ only the non-singlet term contributes. The pure-singlet term emerges at 3-loop order.

The heavy quark contribution to the structure functions $F_{(2,L)}(x,Q^2)$ for one heavy quark of mass $m$ and $N_F$ light flavors is then given by, cf. [44], in case of pure photon exchange^6^

4 $$\begin{aligned}&\!\!\!\frac{1}{x} F_{(2,L)}^{Q\overline{Q}}(x,N_F+1,Q^2,m^2) \nonumber \\&\!\!\!\quad =\sum _{k=1}^{N_F}e_k^2\Biggl \{ L_{q,(2,L)}^\mathsf{NS}\left( x,N_F+1,\frac{Q^2}{\mu ^2} ,\frac{m^2}{\mu ^2}\right) \nonumber \\&\!\!\!\qquad \otimes \Bigl [f_k(x,\mu ^2,N_F)+f_{\overline{k}}(x,\mu ^2,N_F)\Bigr ]\nonumber \\&\!\!\!\qquad +\frac{1}{N_F}L_{q,(2,L)}^{\mathsf{PS}}\left( x,N_F+1,\frac{Q^2}{\mu ^2} ,\frac{m^2}{\mu ^2}\right) \otimes \Sigma (x,\mu ^2,N_F) \nonumber \\&\!\!\!\qquad +\frac{1}{N_F}L_{g,(2,L)}^{\mathsf{S}}\left( x,N_F+1,\frac{Q^2}{\mu ^2}, \frac{m^2}{\mu ^2}\right) \otimes G(x,\mu ^2,N_F) \Biggr \}\nonumber \\&\!\!\!\qquad +e_Q^2\Biggl [ H_{q,(2,L)}^{\mathsf{PS}}\left( x,N_F+1,\frac{Q^2}{\mu ^2}, \frac{m^2}{\mu ^2}\right) \otimes \Sigma (x,\mu ^2,N_F) \nonumber \\&\!\!\!\qquad +H_{g,(2,L)}^{\mathsf{S}}\left( x,N_F+1,\frac{Q^2}{\mu ^2}, \frac{m^2}{\mu ^2}\right) \otimes G(x,\mu ^2,N_F) \Biggr ]. \end{aligned}$$

The meaning of the argument $(N_F +1)$ in Eq. (4) in the massive Wilson coefficients shall be interpreted as $N_F$ *massless* and one *massive flavor*. The symbol $\otimes$ denotes the Mellin convolution,^7^

5 $$\begin{aligned}{}[A \otimes B](x) = \int _0^1 \int _0^1 \mathrm{d}x_1 \mathrm{d}x_2~\delta (x - x_1 x_2) A(x_1) B(x_2).\nonumber \\ \end{aligned}$$

The charges of the light quarks are denoted by $e_k$ and that of the heavy quark by $e_Q$. The scale $\mu ^2$ is the factorization scale, and $f_k, f_{\overline{k}}, \Sigma$ and $G$ are the quark, anti-quark, flavor singlet and gluon distribution functions, with

6 $$\begin{aligned} \Sigma (x,\mu ^2,N_F) = \sum _{k=1}^{N_F} \left[ f_k(x,\mu ^2,N_F) + f_{\overline{k}}(x,\mu ^2,N_F)\right] .\nonumber \\ \end{aligned}$$

The representations in Mellin $N$-space are obtained performing the transformation

7 $$\begin{aligned} \mathbf{M}[f(x)](N) = \int _0^1~\mathrm{d}x~x^{N-1}~f(x). \end{aligned}$$

An important part of the kinematic region in case of heavy flavor production in DIS is located at larger values of $Q^2$, cf., e.g., [93, 94]. As has been shown in Ref. [37], the heavy flavor Wilson coefficients $H_{i,j},~L_{i,j}$ factorize in the limit $Q^2 \gg m^2$ into massive operator matrix elements $A_{ki}$ and the massless Wilson coefficients $C_{i,j}$, if one heavy quark flavor and $N_F$ light flavors are considered. The massive OMEs are process-independent quantities and contain all the mass dependence except for the power corrections $\propto ~(m^2/Q^2)^k,~k\ge 1$. The process dependence is implied by the massless Wilson coefficients. This allows the analytic calculation of the NLO heavy flavor Wilson coefficients, [37, 46]. Comparing these asymptotic expressions with the exact LO and NLO results obtained from Refs. [4–8] and [1–3], respectively, one finds that this approximation becomes valid in case of $F_2^{Q\overline{Q}}$ for $Q^2/m^2 \gtrsim 10$. These scales are sufficiently low and match with the region analyzed in deeply inelastic scattering for precision measurements. In case of $F_L^{Q\overline{Q}}$, this approximation is only valid for $Q^2/m^2 \gtrsim 800$, [37]. The different behavior if compared to $F_2(x,Q^2)$ is due to the emergence of terms $\propto (m^2/Q^2) \ln (m^2/Q^2)$, which only vanish slowly in the limit $Q^2/m^2 \rightarrow \infty$. Here, we present the inclusive corrections for the structure function itself. The renormalized massive OMEs depend on the ratio $m^2/\mu ^2$, while the scale ratio in the massless Wilson coefficients is $\mu ^2/Q^2$. The latter are pure functions of the momentum fraction $z$, or the Mellin variable $N$, if one sets $\mu ^2 = Q^2$. The mass dependence on the heavy flavor Wilson coefficients in the asymptotic region derives from the un-renormalized massive OMEs

8 $$\begin{aligned} \hat{A}^{(3)}_{ij}(\varepsilon ) = \frac{1}{\varepsilon ^3} {a}^{(3),3}_{ij} +\frac{1}{\varepsilon ^2} {a}^{(3),2}_{ij} +\frac{1}{\varepsilon } {a}^{(3),1}_{ij} + {a}^{(3),0}_{ij}, \end{aligned}$$

applying mass, coupling constant, and operator renormalization, as well as mass factorization, cf. Ref. [39]. The renormalized massive OMEs obey then the general structure

9 $$\begin{aligned} A^{(3)}_{ij}\left( \frac{m^2}{\mu ^2}\right)&= \hat{a}^{(3),3}_{ij} \ln ^3\left( \frac{m^2}{\mu ^2}\right) + \hat{a}^{(3),2}_{ij} \ln ^2\left( \frac{m^2}{\mu ^2}\right) \nonumber \\&+ \,\hat{a}^{(3),1}_{ij} \ln \left( \frac{m^2}{\mu ^2}\right) + \hat{a}^{(3),0}_{ij}. \end{aligned}$$

The subsequent calculations will be performed in the $\overline{\mathrm{MS}}$ scheme for the coupling constant and the on-shell scheme for the heavy quark mass $m$. The transition to the scheme in which $m$ is renormalized in the $\overline{\mathrm{MS}}$-scheme is described in Ref. [39]. The strong coupling constant is obtained as the *perturbative* solution of the equation

10 $$\begin{aligned} \frac{\mathrm{d} a_s(\mu ^2)}{\mathrm{d}\ln (\mu ^2)} = - \sum _{l=0}^\infty \beta _l a_s^{l+2}(\mu ^2) \end{aligned}$$

to 3-loop order, where $\beta _k$ are the expansion coefficients of the massless QCD $\beta$-function and $\mu ^2$ denotes the renormalization scale. For simplicity, we identify the factorization $(\mu _F)$ and renormalization $(\mu _R)$ scales from now on. In the subsequent sections, we present explicit expressions of the asymptotic heavy flavor Wilson coefficients in Mellin $N$-space. They depend on the logarithms

11 $$\begin{aligned} L_Q = \ln \left( \frac{Q^2}{\mu ^2}\right) \quad \text {and}\quad L_M = \ln \left( \frac{m^2}{\mu ^2}\right) , \end{aligned}$$

where $$\mu \equiv \mu _F = \mu _R$$.

Besides the Wilson coefficients (2), the massive OMEs are important themselves to establish the matching conditions in the variable flavor number scheme in describing the process of a single massive quark becoming massless^8^ at large enough scales $\mu ^2$, [39, 44]. Here, the PDFs for $N_F+1$ massless quarks are related to the former $N_F$ massless quarks process independently. The corresponding relations to 3-loop order read, cf. also [44]^9^:

12 $$\begin{aligned}&\!\!\!f_k(N_F+1, \mu ^2)+f_{\overline{k}}(N_F+1, \mu ^2) =A_{qq,Q}^{\mathsf{NS}}\Big (N_F,\frac{\mu ^2}{m^2}\Big )\nonumber \\&\!\!\!\quad \otimes \,\, \left[ f_k(N_F, \mu ^2) + f_{\overline{k}}(N_F, \mu ^2)\right] \nonumber \\&\!\!\!\quad +\,\, {\tilde{A}_{qq,Q}^{\mathsf{PS}}\Big (N_F, \frac{\mu ^2}{m^2}\Big )}\otimes {\Sigma (N_F, \mu ^2)}\nonumber \\&\!\!\!\quad +\,\, {\tilde{A}_{qg,Q}^{\mathsf{S}}\Big (N_F, \frac{\mu ^2}{m^2}\Big )}\otimes {G(N_F, \mu ^2)}\end{aligned}$$

13 $$\begin{aligned}&\!\!\!{f_{Q+\bar{Q}}(N_F+1, \mu ^2)}={A_{Qq}^{\mathsf{PS}}\left( N_F, \frac{\mu ^2}{m^2}\right) }\otimes {\Sigma (N_F, \mu ^2)}\nonumber \\&\!\!\!\quad +\,\, {A_{Qg}^{\mathsf{S}}\Big (N_F, \frac{\mu ^2}{m^2}\Big )} \otimes {G(N_F, \mu ^2)}\end{aligned}$$

14 $$\begin{aligned}&\!\!\!{G(N_F+1, \mu ^2)}={A_{gq,Q}^{\mathsf{S}}\Bigl (N_F, \frac{\mu ^2}{m^2}\Bigr )} \otimes {\Sigma (N_F,\mu ^2)}\nonumber \\&\!\!\!\quad +\,\, {A_{gg,Q}^\mathsf{S}\left( N_F,\frac{\mu ^2}{m^2}\right) } \otimes {G(N_F,\mu ^2)}\end{aligned}$$

15 $$\begin{aligned}&\!\!\!{\Sigma (N_F+1,\mu ^2)}=\left[ {A_{qq,Q}^\mathsf{NS}\left( N_F, \frac{\mu ^2}{m^2}\right) }\right. \nonumber \\&\!\!\!\quad +\,\, N_F {\tilde{A}_{qq,Q}^{\mathsf{PS}} \left( N_F, \frac{\mu ^2}{m^2}\right) } \left. + {{A}_{Qq}^{\mathsf{PS}} \left( N_F, \frac{\mu ^2}{m^2}\right) } \right] \nonumber \\&\!\!\!\quad \otimes \,\, {\Sigma (N_F,\mu ^2)} \nonumber \\&\!\!\!\quad +\,\,\left[ N_F {\tilde{A}^{\mathsf{S}}_{qg,Q}\left( N_F, \frac{\mu ^2}{m^2}\right) }+{{A}^{\mathsf{S}}_{Qg}\left( N_F, \frac{\mu ^2}{m^2}\right) } \right] \nonumber \\&\!\!\!\quad \otimes \,\, {G(N_F,\mu ^2)}. \end{aligned}$$

The meaning of the symbols $\tilde{f}$ and $\hat{f}$ applied to a function here and in the following was defined in [39], Eqs. (2.3) and (2.4). Here, the $N_F$-dependence of the OMEs is understood as functional and $\mu ^2$ denotes the matching scale, which for the heavy-to-light transitions is normally much larger than the mass scale $m^2$, [54]. We will present the corresponding OMEs in Appendix A. The results of the calculations given in the subsequent sections have been obtained making mutual use of the packages HarmonicSums [95–98] and Sigma [63–70].

## The Wilson coefficients $L_{q,2}^{\mathsf{PS}}$ and $L_{g,2}^{\mathsf{S}}$

The constant parts of the un-renormalized massive OMEs $A_{qq,Q}^{\mathsf{PS}}$ and $A_{qg,A}^{\mathsf{S}}$ have been calculated in Ref. [55]. The OMEs contribute for the first time at 3- and 2-loop order, respectively, and stem from processes in which the virtual electro-weak gauge boson couples to a massless quark. Here and in the following $N_F$ denotes the number of massless flavors.

As a shorthand notation, we define the function

16 $$\begin{aligned} \tilde{\gamma }_{qg}^{0} = -4 \frac{N^2 + N + 2}{N (N+1) (N+2)}, \end{aligned}$$

denoting the kinetic part of the leading order anomalous dimension $\gamma _{qg}^{(0)}$ separating off the corresponding color factor.

In Mellin $N$-space the Wilson coefficient $L_{q,2}^{\mathsf{PS}}$ reads:

$$\begin{aligned}&\!\!\!L_{q,2}^{\mathsf{PS}} = \tfrac{1}{2}\left[ 1 + (-1)^N\right] \nonumber \\&\!\!\!\quad \times \,\, {a_s^3} \Biggl \{ {C_F} {N_F} {T_F^2} \Biggl [ -\frac{32 P_{4} L_Q^2}{9 (N-1) N^3 (N+1)^3 (N+2)^2}\nonumber \\&\!\!\!\quad +\,\,L_Q \Biggl [\frac{64 P_{6}}{27 (N-1) N^4 (N+1)^4 (N+2)^3} \nonumber \\&\!\!\!\quad -\,\,\frac{256 P_{1} (-1)^N}{9 (N-1) N^2 (N+1)^3 (N+2)^3} +\frac{2 (\tilde{\gamma }_{qg}^{0})^2 (N+2) L_M^2}{3 (N-1)} \nonumber \\&\!\!\!\quad +\Biggl [ \frac{64 \big (N^2+N+2\big ) \big (8 N^3+13 N^2+27 N+16\big )}{9 (N-1) N^2 (N+1)^3 (N+2)}\nonumber \\&\!\!\!\quad -\,\,\frac{64 \big (N^2+N+2\big )^2 S_1}{3 (N-1) N^2 (N+1)^2 (N+2)}\Biggr ] L_M \nonumber \\&\!\!\!\quad +\,\,\frac{512 S_{-2}}{3 (N-1) N (N+1) (N+2)}\Biggr ]\nonumber \\&\!\!\quad -\,\,\frac{32 P_{4} L_M^2}{9 (N-1) N^3 (N+1)^3 (N+2)^2} \nonumber \\&\quad +\,\,\Biggl [ -\frac{32 P_{7}}{27 (N-1) N^4 (N+1)^4 (N+2)^3}\nonumber \\ \end{aligned}$$

17 $$\begin{aligned}&\quad +\,\,\frac{64 P_{2} S_1}{3 (N-1) N^3 (N+1)^3 (N+2)^2} \nonumber \\&\quad +\,\,\frac{\big (N^2+N+2\big )^2}{(N-1) N^2 (N+1)^2 (N+2)} \frac{32}{3} \left( S_1^2 - S_2\right) \Biggr ] L_M\nonumber \\&\quad -\,\,\frac{32 P_{9}}{243 (N-1) N^5 (N+1)^5 (N+2)^4}\nonumber \\&\quad +\,\,\frac{32 P_{8} S_1}{81 (N-1) N^4 (N+1)^4 (N+2)^3} \nonumber \\&\quad -\,\,\frac{16 P_{3} S_1^2}{27 (N-1) N^3 (N+1)^3 (N+2)^2} \nonumber \\&\quad -\,\,\frac{16 P_{5} S_2}{27 (N-1) N^3 (N+1)^3 (N+2)^2} \nonumber \\&\quad +\,\,\frac{32 L_Q^3 \big (N^2+N+2\big )^2}{9 (N-1) N^2 (N+1)^2 (N+2)}\nonumber \\&\quad -\,\,\frac{32\big (N^2+N+2\big )^2 L_M^3}{9 (N-1) N^2 (N+1)^2 (N+2)} \nonumber \\&\quad +\,\,\frac{\big (N^2+N+2\big )^2}{(N-1) N^2 (N+1)^2 (N+2)}\nonumber \\&\quad \times \,\,\Bigl [-\frac{64}{27} S_1^3 +\frac{32}{9} S_2 S_1 +\frac{160 S_3}{27} +\frac{256 \zeta _3}{9}\Bigr ]\Biggr ] \nonumber \\&\quad +\,\,{N_F} \hat{\tilde{C}}_{2,q}^{\mathsf{PS},(3)}({N_F})\Biggr \}, \end{aligned}$$

with the polynomials

18 $$\begin{aligned} P_{1}&= 4 N^6+22 N^5+48 N^4+53 N^3+45 N^2\nonumber \\&+\,\,36 N+8\end{aligned}$$

19 $$\begin{aligned} P_{2}&= N^7-15 N^5-58 N^4-92 N^3-76 N^2\nonumber \\&-\,\,48 N-16\end{aligned}$$

20 $$\begin{aligned} P_{3}&= N^7-37 N^6-248 N^5-799 N^4\nonumber \\&-\,\,1183 N^3-970 N^2-580 N-168\end{aligned}$$

21 $$\begin{aligned} P_{4}&= 11 N^7+37 N^6+53 N^5+7 N^4\nonumber \\&-\,\,68 N^3-56 N^2-80 N-48\end{aligned}$$

22 $$\begin{aligned} P_{5}&= 49 N^7+185 N^6+340 N^5+287 N^4\nonumber \\&+\,\,65 N^3+62 N^2-196 N-168\end{aligned}$$

23 $$\begin{aligned} P_{6}&= 85 N^{10}+530 N^9+1458 N^8+2112 N^7\nonumber \\&+\,\,1744 N^6+2016 N^5+3399 N^4+2968 N^3\nonumber \\&+\,\,1864 N^2+1248 N+432\end{aligned}$$

24 $$\begin{aligned} P_{7}&= 143 N^{10}+838 N^9+1995 N^8+1833 N^7\nonumber \\&-1609 N^6-5961 N^5-7503 N^4-6928 N^3\nonumber \\&-\,\,4024 N^2-816 N+144\end{aligned}$$

25 $$\begin{aligned} P_{8}&= 176 N^{10}+973 N^9+1824 N^8-948 N^7\nonumber \\&-\,\,10192 N^6-19173 N^5-20424 N^4-16036 N^3\nonumber \\&-\,\,7816 N^2-1248 N+288\end{aligned}$$

26 $$\begin{aligned} P_{9}&= 1717 N^{13}+16037 N^{12}+66983 N^{11}\nonumber \\&+\,\,161797 N^{10}+241447 N^9+216696 N^8+86480 N^7\nonumber \\&-\,\,67484 N^6-170003 N^5-165454 N^4\nonumber \\&-\,\,81976 N^3-15792 N^2-1008 N-864. \end{aligned}$$

For the massless 3-loop Wilson coefficients $C_{i,j}^{k}$ we refer to Ref. [38]. Here and in the following, their expression will be kept symbolically. The harmonic sums are defined recursively by, cf. [79, 80],

27 $$\begin{aligned} S_{b,\vec {a}}(N)&= \sum _{k=1}^N \frac{(\mathrm{sign}(b))^k}{k^{|b|}} S_{\vec {a}}(k),\nonumber \\&b, a_i \in \mathbb {Z} \backslash \{0\}, N \in \mathbb {N}, N \ge 1, S_\emptyset = 1. \end{aligned}$$

The color factors are $C_F = (N_c^2-1)/(2 N_c), C_A = N_c, T_F = 1/2$ for $SU(N_c)$ and $N_c = 3$ for QCD.

Likewise the Wilson coefficient $L_{g,2}^{\mathsf{S}}$ is given by:

$$\begin{aligned}&\!\!\!L_{g,2}^{\mathsf{S}} = \tfrac{1}{2} \left[ 1 + (-1)^N\right] \left\{ {a_s^2 T_F^2} {N_F} \Biggl \{ L_M\Biggl [\frac{4}{3} \tilde{\gamma }_{qg}^{0} S_1\right. \nonumber \\&\quad -\,\,\frac{16 \big (N^3-4 N^2-N-2\big )}{3 N^2 (N+1) (N+2)}\Biggr ]\nonumber \\&\quad \left. -\,\,\frac{4}{3} \tilde{\gamma }_{qg}^{0} L_Q L_M\right\} +{a_s^3} \left\{ {N_F} {T_F^3} \Biggl [ L_M^2 \Biggl [\frac{16}{9} \tilde{\gamma }_{qg}^{0} S_1\right. \nonumber \\&\quad -\,\,\frac{64 \big (N^3-4 N^2-N-2\big )}{9 N^2 (N+1) (N+2)}\Biggr ] -\frac{16}{9} \tilde{\gamma }_{qg}^{0} L_Q L_M^2\Biggr ] \nonumber \\&\quad +\,\,{C_A} {N_F} {T_F^2} \Biggl [ \Biggl [ \frac{64 \big (N^2+N+1\big ) \big (N^2+N+2\big )}{9 (N-1) N^2 (N+1)^2 (N+2)^2}\nonumber \\&\quad +\,\,\frac{8}{9} \tilde{\gamma }_{qg}^{0} S_1\Biggr ] L_Q^3 +\Biggl [ -\frac{64 (-1)^N \big (N^3+4 N^2+7 N+5\big )}{3 (N+1)^3 (N+2)^3}\nonumber \\&\quad +\,\,\frac{8 P_{25}}{9 (N-1) N^3 (N+1)^3 (N+2)^3}\nonumber \\&\quad +\,\,\frac{32 \big (8 N^4-7 N^3+5 N^2-17 N-13\big ) S_1}{9 (N-1) N (N+1)^2 (N+2)}\nonumber \\&\quad +\,\,L_M \Biggl [ \frac{64 \big (N^2+N+1\big ) \big (N^2+N+2\big )}{3 (N-1) N^2 (N+1)^2 (N+2)^2} +\frac{8}{3} \tilde{\gamma }_{qg}^{0} S_1\Biggr ]\nonumber \\ \end{aligned}$$

$$\begin{aligned}&\quad +\,\,\tilde{\gamma }_{qg}^{0} \Bigl [ -\frac{4}{3} S_1^2 +\frac{4 S_2}{3} +\frac{8}{3} S_{-2}\Bigr ]\Biggr ] L_Q^2\nonumber \\&\quad +\,\,\Biggl [ -\frac{32 \big (8 N^4-7 N^3+5 N^2-17 N-13\big ) S_1^2}{9 (N-1) N (N+1)^2 (N+2)}\nonumber \\&\quad +\,\,\frac{128 (-1)^N \big (N^3+4 N^2+7 N+5\big ) S_1}{3 (N+1)^3 (N+2)^3}\nonumber \\&\quad -\,\,\frac{32 P_{24} S_1}{27 (N-1) N^2 (N+1)^3 (N+2)^3}\nonumber \\&\quad +\,\,\frac{64(-1)^N P_{18}}{9 (N-1) N^2 (N+1)^4 (N+2)^4}\nonumber \\&\quad -\,\,\frac{16 P_{32}}{27 (N-1) N^3 (N+1)^4 (N+2)^4}\nonumber \\&\quad +\,\,L_M^2 \Biggl [ \frac{64 \big (N^2+N+1\big ) \big (N^2+N+2\big )}{3 (N-1) N^2 (N+1)^2(N+2)^2}\nonumber \\&\quad +\,\,\frac{8}{3} \tilde{\gamma }_{qg}^{0} S_1\Biggr ] +\frac{32 \big (8 N^4+13 N^3-22 N^2-9 N-26\big ) S_2}{9 (N-1) N (N+1) (N+2)^2}\nonumber \\&\quad +\,\,\frac{128 \big (N^2+N-1\big ) S_3}{9 N (N+1) (N+2)}\nonumber \\ \end{aligned}$$

$$\begin{aligned}&\quad +\,\,\frac{64 \big (8 N^5+15 N^4+6 N^3+11 N^2+16 N+16\big ) S_{-2}}{9 (N-1) N (N+1)^2 (N+2)^2}\nonumber \\&\quad +\,\,L_M \Biggl [ \frac{32 P_{26}}{9 (N-1) N^3 (N+1)^3 (N+2)^3}\nonumber \\&\quad -\,\,\frac{128 (-1)^N \big (N^3+4 N^2+7 N+5\big )}{3 (N+1)^3 (N+2)^3}\nonumber \\&\quad -\,\,\frac{64 (2 N-1) \big (N^3+9 N^2+7 N+7\big ) S_1}{9 (N-1) N (N+1)^2 (N+2)}\nonumber \\&\quad +\,\,\tilde{\gamma }_{qg}^{0} \Bigl [ -\frac{8}{3} S_1^2 +\frac{8 S_2}{3} +\,\,\frac{16}{3} S_{-2}\Bigr ]\Biggr ] -\frac{128 \big (N^2+N+3\big ) S_{-3}}{3 N (N+1) (N+2)}\nonumber \\&\quad +\tilde{\gamma }_{qg}^{0} \Bigl [\frac{8}{9} S_1^3-8 S_2 S_1 +\frac{32}{3} S_{2,1}\Bigr ]\nonumber \\&\quad \left. +\,\,\frac{256 S_{-2,1}}{3 N (N+1) (N+2)}+\frac{(N-1) \Bigl [\frac{64}{3} S_{-2} S_1-32 \zeta _3\Bigr ]}{N (N+1)}\right] L_Q\nonumber \\&\quad +\,\,\frac{16 P_{12} S_1^2}{81 N (N+1)^3 (N+2)^3}\nonumber \\&\quad +\,\,\frac{8 P_{39}}{243 (N-1) N^5 (N+1)^5 (N+2)^5}\nonumber \\&\quad +\,\,\frac{512}{9} \frac{\big (N^2+N+1\big ) \big (N^2+N+2\big )}{(N-1) N^2 (N+1)^2 (N+2)^2} \zeta _3\nonumber \\&\quad +\,\,\frac{8 P_{36} S_1}{243 (N-1) N^4 (N+1)^4 (N+2)^4}\nonumber \\&\quad +\,\,L_M^3 \Biggl [ -\frac{64 \big (N^2+N+1\big ) \big (N^2+N+2\big )}{9 (N-1) N^2 (N+1)^2 (N+2)^2}\nonumber \\ \end{aligned}$$

$$\begin{aligned}&\quad -\,\,\frac{8}{9} \tilde{\gamma }_{qg}^{0} S_1\Biggr ] -\frac{16 P_{13} S_2}{81 N (N+1)^3 (N+2)^3}\nonumber \\&\quad +\,\,\frac{64 \big (5 N^4+38 N^3+59 N^2+31 N+20\big ) S_3}{81 N (N+1)^2 (N+2)^2}\nonumber \\&\quad -\,\,\frac{32 \big (121 N^3+293 N^2+414 N+224\big ) S_{-2}}{81 N (N+1)^2 (N+2)}\nonumber \\&\quad +\,\,L_M^2 \Biggl [ -\frac{64 (-1)^N \big (N^3+4 N^2+7 N+5\big )}{3 (N+1)^3 (N+2)^3}\nonumber \\&\quad +\,\,\frac{8 P_{25}}{9 (N-1) N^3 (N+1)^3 (N+2)^3}\nonumber \\&\quad +\,\,\frac{32 \big (8 N^4-7 N^3+5 N^2-17 N-13\big ) S_1}{9 (N-1) N (N+1)^2 (N+2)}\nonumber \\&\quad +\,\,\tilde{\gamma }_{qg}^{0} \Bigl [ -\frac{4}{3} S_1^2+\,\,\frac{4 S_2}{3} +\frac{8}{3} S_{-2}\Bigr ]\Biggr ] \nonumber \\&\quad +\frac{128 \big (5 N^2+8 N+10\big ) S_{-3}}{27 N (N+1) (N+2)}\nonumber \\&\quad +\,\,\frac{\big (5 N^4+20 N^3+41 N^2+49 N+20\big ) \Bigl [\frac{32}{81} S_1^3 -\frac{32}{27} S_2 S_1 +\frac{128}{27} S_{2,1}\Bigr ]}{N (N+1)^2 (N+2)^2}\nonumber \\&\quad +\,\,L_M \Biggl [\frac{32 \big (2 N^5+21 N^4+27 N^3+11 N^2+25 N-14\big ) S_1^2}{9 (N-1) N (N+1)^2 (N+2)^2}\nonumber \\&\quad +\,\,\frac{16 P_{27} S_1}{27 (N-1) N^3 (N+1)^3 (N+2)^3}\nonumber \\ \end{aligned}$$

$$\begin{aligned}&\quad +\,\,\frac{128 (-1)^N \big (N^3+4 N^2+7 N+5\big ) S_1}{3 (N+1)^3 (N+2)^3} -\frac{16}{3} \tilde{\gamma }_{qg}^{0} S_2 S_1\nonumber \\&\quad -\,\,\frac{64 (-1)^N P_{14}}{9 (N-1) N^2 (N+1)^3 (N+2)^4}\nonumber \\&\quad +\,\,\frac{16 P_{34}}{27 (N-1) N^4 (N+1)^4 (N+2)^4}\nonumber \\&\quad -\,\,\frac{32 \big (2 N^5+21 N^4+51 N^3+23 N^2-11 N-14\big ) S_2}{9 (N-1) N (N+1)^2 (N+2)^2}\nonumber \\&\quad +\,\,\frac{64 S_3}{3 (N+2)}\nonumber \\&\quad -\,\,\frac{64 \big (2 N^5+21 N^4+36 N^3-7 N^2-68 N-56\big ) S_{-2}}{9 (N-1) N (N+1)^2 (N+2)^2}\nonumber \\&\quad +\frac{\frac{256}{3} S_{-2,1} -\frac{128}{3} S_{-3}}{N (N+1) (N+2)}\nonumber \\&\quad +\frac{(N-1) \big (\frac{64}{3} S_{-2} S_1-32 \zeta _3\big )}{N (N+1)}\Biggr ]+\tilde{\gamma }_{qg}^{0} \Bigl [\frac{1}{27} S_1^4 -\frac{2}{9} S_2 S_1^2\nonumber \\&\quad +\Bigl [\frac{16}{9} S_{2,1} -\frac{40 S_3}{27}\Bigr ] S_1 +\frac{64}{9}\zeta _3 S_1 +\frac{1}{9} S_2^2 +\frac{14 S_4}{9}\nonumber \\&\quad +\frac{32}{9} S_{-4} +\frac{32}{9} S_{3,1} -\frac{16}{9} S_{2,1,1}\Bigr ]\Biggr ] +{C_F} {N_F} {T_F^2} \nonumber \\&\quad \times \Biggl [ \Biggl [\frac{16 \big (N^2+N+1\big ) \big (N^2+N+2\big ) \big (3 N^4+6 N^3-N^2-4 N+12\big )}{9 (N-1) N^3 (N+1)^3 (N+2)^2}\nonumber \\&\quad +\frac{16}{9} \tilde{\gamma }_{qg}^{0} S_1\Biggr ] L_Q^3\nonumber \\&\quad +\Biggl [-\frac{4 P_{31}}{9 (N-1) N^4 (N+1)^4 (N+2)^3} +\frac{16 P_{21} S_1}{9 (N-1) N^3 (N+1)^3 (N+2)^2}\nonumber \\&\quad +\tilde{\gamma }_{qg}^{0} \Bigl [\frac{20 S_2}{3}-4 S_1^2\Bigr ]\nonumber \\ \end{aligned}$$

$$\begin{aligned}&\quad +L_M\Biggl [\frac{8 \big (N^2+N+2\big ) P_{10}}{3 (N-1) N^3 (N+1)^3 (N+2)^2} +\frac{8}{3} \tilde{\gamma }_{qg}^{0} S_1\Biggr ] \Biggr ] L_Q^2\nonumber \\&\quad +\Biggl [ \frac{16 L_M^2 \big (N^2+N+2\big )^3}{(N-1) N^3 (N+1)^3 (N+2)^2}\nonumber \\&\quad -\frac{16 P_{22} S_1^2}{9 (N-1) N^3 (N+1)^3(N+2)^2}\nonumber \\&\quad +\frac{64 (-1)^N P_{37}}{45 (N-2) (N-1)^2 N^3 (N+1)^4 (N+2)^4 (N+3)^3}\nonumber \\&\quad +\frac{4 P_{42}}{45 (N-1)^2 N^5 (N+1)^5 (N+2)^4 (N+3)^3}\nonumber \\&\quad -\frac{8 P_{30} S_1}{9 (N-1) N^4 (N+1)^4 (N+2)^3}\nonumber \\&\quad +\frac{16 P_{23} S_2}{9 (N-1) N^3 (N+1)^3 (N+2)^2}\nonumber \\&\quad +L_M \Biggl [ -\frac{16 P_{28}}{3 (N-1) N^4 (N+1)^4 (N+2)^3}\nonumber \\&\quad +\frac{16 P_{17} S_1}{3 (N-1) N^3 (N+1)^3 (N+2)^2}+\tilde{\gamma }_{qg}^{0} \left( \frac{16 S_2}{3} -\frac{16}{3} S_1^2\right) \Biggr ]\nonumber \\&\quad -\frac{256 \big (N^2+N+1\big ) S_3}{3 N (N+1) (N+2)}\nonumber \\&\quad +\frac{64 P_{16} S_{-2}}{3 (N-2) (N-1) N^2 (N+1)^2 (N+2)^2 (N+3)}\nonumber \\&\quad +\tilde{\gamma }_{qg}^{0} \Bigl [\frac{8}{3} S_1^3-8 S_2 S_1 -\frac{32}{3} S_{2,1}\Bigr ]\nonumber \\ \end{aligned}$$

$$\begin{aligned}&\quad +\frac{\frac{512}{3} S_1 S_{-2} +\frac{256}{3} S_{-3} -\frac{512}{3} S_{-2,1}}{N (N+1) (N+2)} +\frac{64 (N-1) \zeta _3}{N (N+1)}\Biggr ] L_Q \nonumber \\&\quad -\frac{64}{9} \frac{\big (N^2+N+2\big ) P_{10} \zeta _3}{(N-1) N^3 (N+1)^3 (N+2)^2}\nonumber \\&\quad +\frac{8 \big (215 N^4+481 N^3+930 N^2+748 N+120\big ) S_1^2}{81 N^2 (N+1)^2 (N+2)}\nonumber \\&\quad +\frac{P_{40}}{243 (N-1) N^6 (N+1)^6 (N+2)^5}\nonumber \\&\quad -\frac{4 P_{35} S_1}{243 (N-1) N^5 (N+1)^5 (N+2)^2}\nonumber \\&\quad +L_M^3 \Biggl [\frac{8 \big (N^2+N+2\big ) P_{10}}{9 (N-1) N^3 (N+1)^3 (N+2)^2} +\frac{8}{9} \tilde{\gamma }_{qg}^{0} S_1\Biggr ]\nonumber \\&\quad +L_M^2 \Biggl [\frac{4 P_{29}}{9 (N-1) N^4 (N+1)^4 (N+2)^3}\nonumber \\&\quad -\frac{16 P_{20} S_1}{9 (N-1) N^3 (N+1)^3 (N+2)^2}+\tilde{\gamma }_{qg}^{0}\Bigl [ -\frac{4}{3} S_1^2 -\frac{4 S_2}{3}\Bigr ]\Biggr ]\nonumber \\&\quad +\frac{8 \big (109 N^4+291 N^3+478 N^2+324 N+40\big ) S_2}{27 N^2 (N+1)^2 (N+2)}\nonumber \\&\quad +\frac{\big (10 N^3+13 N^2+29 N+6\big ) \Bigl [ -\frac{16}{81} S_1^3 -\frac{16}{27} S_2 S_1\Bigr ]}{N^2 (N+1) (N+2)}\nonumber \\&\quad +\frac{32 \big (5 N^3-16 N^2+N-6\big ) S_3}{81 N^2 (N+1) (N+2)}\nonumber \\ \end{aligned}$$

28 $$\begin{aligned}&\quad +\tilde{\gamma }_{qg}^{0} \Bigl [ -\frac{1}{27} S_1^4 -\frac{2}{9} S_2 S_1^2 -\frac{8}{27} S_3 S_1 -\frac{64}{9} \zeta _3 S_1-\frac{1}{9} S_2^2 +\frac{14 S_4}{9}\Bigr ] \nonumber \\&\quad +L_M \Biggl [ -\frac{8 P_{19} S_1^2}{9 (N-1) N^3 (N+1)^3 (N+2)^2}\nonumber \\&\quad -\frac{16 P_{33} S_1}{27 (N-1) N^4 (N+1)^4 (N+2)^3} \nonumber \\&\quad +\frac{64 (-1)^N P_{38}}{45 (N-2) (N-1)^2 N^3 (N+1)^4 (N+2)^4 (N+3)^3}\nonumber \\&\quad +\frac{8 \big (N^2+N+2\big ) P_{11} S_2}{9 (N-1) N^3 (N+1)^3 (N+2)^2} \nonumber \\&\quad +\frac{4 P_{41}}{135 (N-1)^2 N^5 (N+1)^5 (N+2)^4 (N+3)^3}\nonumber \\&\quad +\frac{64 P_{15} S_{-2}}{3 (N-2) (N-1) N^2 (N+1)^2 (N+2)^2 (N+3)} \nonumber \\&\quad +\tilde{\gamma }_{qg}^{0} \Big (\frac{8}{3} S_1^3 -\frac{8}{3} S_2 S_1 -\frac{16}{3} S_{2,1}\Big )\nonumber \\&\quad +\frac{\frac{512}{3} S_1 S_{-2} +\frac{256}{3} S_{-3} -\frac{512}{3} S_{-2,1}}{N (N+1)(N+2)}\nonumber \\&\quad \left. \left. +\frac{(N-1) \big (64 \zeta _3 -\frac{64 S_3}{3}\big )}{N (N+1)}\Biggr ]\Bigg ] +{N_F} \hat{\tilde{C}}_{2,g}^{\mathsf{S},(3)}({N_F})\right\} \right\} , \end{aligned}$$

where

29 $$\begin{aligned} P_{10}&= 3 N^6+9 N^5-N^4-17 N^3-38 N^2-28 N-24\end{aligned}$$

30 $$\begin{aligned} P_{11}&= 47 N^6+141 N^5+59 N^4-117 N^3+2 N^2+84 N+72\nonumber \\ \end{aligned}$$

31 $$\begin{aligned} P_{12}&= 65 N^6+455 N^5+1218 N^4\nonumber \\&+1820 N^3+1968 N^2+1460 N+448\end{aligned}$$

32 $$\begin{aligned} P_{13}&= 139 N^6+1093 N^5+3438 N^4\nonumber \\&+5776 N^3+5724 N^2+3220 N+752\end{aligned}$$

33 $$\begin{aligned} P_{14}&= 9 N^7+71 N^6+214 N^5+320 N^4\nonumber \\&+275 N^3+215 N^2+160 N+32\end{aligned}$$

34 $$\begin{aligned} P_{15}&= N^8+8 N^7-2 N^6-60 N^5-23 N^4\nonumber \\&+108 N^3+96 N^2+16 N+48\end{aligned}$$

35 $$\begin{aligned} P_{16}&= N^8+8 N^7-2 N^6-60 N^5+N^4\nonumber \\&+156 N^3+24 N^2-80 N-240\end{aligned}$$

36 $$\begin{aligned} P_{17}&= 3 N^8+8 N^7-2 N^6-24 N^5+15 N^4\nonumber \\&+88 N^3+152 N^2+96 N+48\end{aligned}$$

37 $$\begin{aligned} P_{18}&= 5 N^8-8 N^7-137 N^6-436 N^5\nonumber \\&-713 N^4-672 N^3-407 N^2-192 N-32\end{aligned}$$

38 $$\begin{aligned} P_{19}&= 7 N^8+4 N^7-90 N^6-224 N^5-21 N^4\nonumber \\&+388 N^3+608 N^2+336 N+144\end{aligned}$$

39 $$\begin{aligned} P_{20}&= 10 N^8+46 N^7+105 N^6+139 N^5\nonumber \\&+87 N^4-17 N^3+50 N^2+84 N+72\end{aligned}$$

40 $$\begin{aligned} P_{21}&= 19 N^8+70 N^7+63 N^6-41 N^5\nonumber \\&-192 N^4-221 N^3-142 N^2-60 N-72\end{aligned}$$

41 $$\begin{aligned} P_{22}&= 38 N^8+146 N^7+177 N^6+35 N^5\nonumber \\&-249 N^4-373 N^3-218 N^2-60 N-72\end{aligned}$$

42 $$\begin{aligned} P_{23}&= 56 N^8+194 N^7+213 N^6+83 N^5\nonumber \\&-231 N^4-469 N^3-290 N^2-60 N-72\end{aligned}$$

43 $$\begin{aligned} P_{24}&= 113 N^8+348 N^7+109 N^6-289 N^5\nonumber \\&-272 N^4-859 N^3-778 N^2-172 N+72\end{aligned}$$

44 $$\begin{aligned} P_{25}&= 9 N^9+54 N^8+56 N^7-110 N^6-381 N^5\nonumber \\&-568 N^4-364 N^3-72 N^2+128 N+96\end{aligned}$$

45 $$\begin{aligned} P_{26}&= 9 N^9+54 N^8+167 N^7+397 N^6+780 N^5\nonumber \\&+1241 N^4+1448 N^3+1200 N^2+608 N+144\end{aligned}$$

46 $$\begin{aligned} P_{27}&= 55 N^9+336 N^8+218 N^7-2180 N^6-6529 N^5\nonumber \\&-9764 N^4-9368 N^3-6032 N^2-2448 N-576\end{aligned}$$

47 $$\begin{aligned} P_{28}&= N^{11}-56 N^9-236 N^8-373 N^7+82 N^6\nonumber \\&+1244 N^5+2330 N^4+2560 N^3+1712 N^2\nonumber \\&+896 N+288 \end{aligned}$$

48 $$\begin{aligned} P_{29}&= 33 N^{11}+231 N^{10}+662 N^9+1254 N^8+1801 N^7\nonumber \\&+2759 N^6+5440 N^5+9884 N^4+12512 N^3\nonumber \\&+9200 N^2+5184 N+1728\end{aligned}$$

49 $$\begin{aligned} P_{30}&= 45 N^{11}+383 N^{10}+958 N^9+526 N^8-763 N^7\nonumber \\&+1375 N^6+7808 N^5+13028 N^4+12976 N^3\nonumber \\&+8016 N^2+4608 N+1728\end{aligned}$$

50 $$\begin{aligned} P_{31}&= 81 N^{11}+483 N^{10}+1142 N^9+1086 N^8-767 N^7\nonumber \\&-4645 N^6-8936 N^5-11980 N^4\nonumber \\&-12352 N^3-8272 N^2-4800 N-1728\end{aligned}$$

51 $$\begin{aligned} P_{32}&= 120 N^{11}+1017 N^{10}+2737 N^9+1292 N^8\nonumber \\&-8086 N^7-20743 N^6-24563 N^5-16702 N^4\nonumber \\&-6840 N^3+120 N^2+2432 N+960\end{aligned}$$

52 $$\begin{aligned} P_{33}&= 121 N^{11}+988 N^{10}+3554 N^9+6972 N^8\nonumber \\&+7131 N^7-846 N^6-14806 N^5-25354 N^4\nonumber \\&-26096 N^3-16752 N^2-8352 N-2592\end{aligned}$$

53 $$\begin{aligned} P_{34}&= 27 N^{12}+441 N^{11}+2206 N^{10}+5360 N^9\nonumber \\&+7445 N^8+8555 N^7+18766 N^6+44852 N^5\nonumber \\&+67572 N^4+63960 N^3+39632 N^2+15648 N+2880\nonumber \\\end{aligned}$$

54 $$\begin{aligned} P_{35}&= 2447 N^{12}+16902 N^{11}+59649 N^{10}\nonumber \\&+125860 N^9+128761 N^8-36530 N^7-248341 N^6\nonumber \\&-304460 N^5-162188 N^4-11724 N^3\nonumber \\&+29160 N^2+19440 N+7776\end{aligned}$$

55 $$\begin{aligned} P_{36}&= 3361 N^{12}+23769 N^{11}+62338 N^{10}+59992 N^9\nonumber \\&-63303 N^8-317823 N^7-585520 N^6\nonumber \\&-640602 N^5-430132 N^4-167536 N^3\nonumber \\&-27648 N^2+9504 N+5184\end{aligned}$$

56 $$\begin{aligned} P_{37}&= 76 N^{14}+802 N^{13}+2979 N^{12}+1847 N^{11}\nonumber \\&-19377 N^{10}-58253 N^9-26543 N^8+170601 N^7\nonumber \\&+362177 N^6+225119 N^5-103240 N^4-193092 N^3\nonumber \\&-137160 N^2-117072 N-25920\end{aligned}$$

57 $$\begin{aligned} P_{38}&= 76 N^{14}+1042 N^{13}+5979 N^{12}+16367 N^{11}\nonumber \\&+11883 N^{10}-47693 N^9-125723 N^8-86079 N^7\nonumber \\&+36437 N^6+22559 N^5-51700 N^4+24828 N^3\nonumber \\&+132840 N^2+116208 N+25920\end{aligned}$$

58 $$\begin{aligned} P_{39}&= 3180 N^{15}+38835 N^{14}+188728 N^{13}+456665 N^{12}\nonumber \\&+460954 N^{11}-406761 N^{10}-1972948 N^9\nonumber \\&-2827653 N^8-1857970 N^7+109786 N^6\nonumber \\&+1302824 N^5+1092456 N^4+265888 N^3\nonumber \\&-227616 N^2-194688 N-44928 \end{aligned}$$

59 $$\begin{aligned} P_{40}&= 28503 N^{17}+297639 N^{16}+1232041 N^{15}\nonumber \\&+2461407 N^{14}+2169615 N^{13}+662941 N^{12}\nonumber \\&+2110979 N^{11}+5346653 N^{10}+2021366 N^9\nonumber \\&-7290864 N^8-11721384 N^7-3689680 N^6\nonumber \\&+15676192 N^5+32276800 N^4+31869312 N^3\nonumber \\&+18809856 N^2+6856704 N+1244160\end{aligned}$$

60 $$\begin{aligned} P_{41}&= 75 N^{18}+3330 N^{17}+35497 N^{16}\nonumber \\&+175010 N^{15}+486862 N^{14}+966996 N^{13}+2037362 N^{12}\nonumber \\&+3604404 N^{11}-1625689 N^{10}-29506022 N^9\nonumber \\&-78753403 N^8-107977014 N^7-71548880 N^6\nonumber \\&+18344016 N^5+89016048 N^4+92657952 N^3\nonumber \\&+58942080 N^2+25505280 N+5598720\end{aligned}$$

61 $$\begin{aligned} P_{42}&= 325 N^{18}+4280 N^{17}+17759 N^{16}-14880 N^{15}\nonumber \\&-412326 N^{14}-1696848 N^{13}-3216546 N^{12}\nonumber \\&-1169232 N^{11}+8956857 N^{10}+23914216 N^9\nonumber \\&+31536899 N^8+25361392 N^7+9982840 N^6\nonumber \\&-10154128 N^5-26098704 N^4-26761536 N^3\nonumber \\&-17642880 N^2-8087040 N-1866240. \end{aligned}$$

In all the representations of the massive Wilson coefficients and OMEs in $N$-space we apply algebraic reduction [99]. The 2-loop term in (28) is purely multiplicative and induced by renormalization only, while the 3-loop contributions require the calculation of massive OMEs. The above Wilson coefficients depend on the harmonic sums

62 $$\begin{aligned} S_1, S_{-2}, S_2, S_{-3}, S_3, S_{-4}, S_4, S_{-2,1}, S_{2,1}, S_{3,1}, S_{2,1,1}, \end{aligned}$$

apart from those defining the massless 3-loop Wilson coefficients [38].^10^ In the above $\zeta _l = \sum _{k=1}^\infty 1/k^l, l \in \mathbb {N}, l \ge 2$ denote the Riemann $\zeta$-values, which are convergent harmonic sums in the limit $N \rightarrow \infty$. In the constant part of the other Wilson coefficients it is expected that more complicated multiple zeta values emerge, which have been dealt with in [101].

In Eq. (28), denominator terms $\propto 1/(N-2)$ occur. They cancel in the complete expression and the rightmost singularity is located at $N = 1$ as expected for this Wilson coefficient. Let us now consider both the small- and large-$x$ dominant terms for both Wilson coefficients. Those of the massless parts were given in [38] before. Both Wilson coefficients contain terms $\propto 1/(N-1)$. For simplicity, we consider the choice of scale $Q^2 = \mu ^2$ here. The expansion of the heavy flavor contribution, subtracting the massless 3-loop Wilson coefficients, denoted by $\widehat{L}_i$, around $N = 1 (x \rightarrow 0)$ and in the limit $N \rightarrow \infty (x \rightarrow 1)$, setting $Q^2 = \mu ^2$, is given by

63 $$\begin{aligned}&\!\!\!\widehat{L}_{q,2}^{\mathsf{PS}}(N \rightarrow 1)\propto \frac{1}{N-1} C_F T_F^2 N_F \Biggl \{ \frac{1024}{27} \zeta _3\nonumber \\&\!\!\!\quad -\,\,\frac{64}{729} \Biggl [ 54 L_M^3 - 81 L_M^2 +342 L_M +500 \Biggr ] \Biggr \}\end{aligned}$$

64 $$\begin{aligned}&\!\!\!\widehat{L}_{g,2}^{\mathsf{S}}(N \rightarrow 1) \propto \frac{1}{N-1} \Biggl \{C_A T_F^2 N_F \Biggl \{ \frac{512}{27} \zeta _3\nonumber \\&\!\!\!\quad -\,\,\frac{16}{729} \Biggl [108 L_M^3 + 540 L_M^2 + 54 L_M +3091\Biggr ]\Biggr \}\nonumber \\&\!\!\!\quad +\,\, C_F T_F^2 N_F \Biggl \{\frac{1024}{27} \zeta _3 - \frac{32}{729} \Biggl [108 L_M^3 -864 L_M^2\nonumber \\&\!\!\!\quad +\,\,1314 L_M -1091 \Biggr ] \Biggr \}\Biggr \}\end{aligned}$$

65 $$\begin{aligned}&\!\!\!\widehat{L}_{q,2}^{\mathsf{PS}}(N \rightarrow \infty )\propto - \frac{64}{27} C_F T_F^2 N_F \frac{\ln ^3(\bar{N})}{N^2}\end{aligned}$$

66 $$\begin{aligned}&\!\!\!\widehat{L}_{g,2}^{\mathsf{S}}(N \rightarrow \infty )\propto -\frac{4}{27} \frac{(N-2) \ln ^4(\bar{N})}{N^2} (C_A - C_F) T_F^2 N_F.\nonumber \\ \end{aligned}$$

The corresponding limits for the contributions of the massless Wilson coefficients behave like

67 $$\begin{aligned}&\!\!\!N_F \hat{\tilde{C}}_{2,q}^{\mathsf{PS},(3)}({N_F})(N \rightarrow 1) \propto \frac{4}{N-1} C_F T_F^2 N_F\nonumber \\&\!\!\!\quad \times \,\, \left[ \frac{22112}{729} - \frac{32}{9} \zeta _2 + \frac{128}{27} \zeta _3 \right] ~[38]\end{aligned}$$

68 $$\begin{aligned}&\!\!\!N_F \hat{\tilde{C}}_{2,g}^{\mathsf{S},(3)}({N_F}) (N \rightarrow 1) \propto \frac{4}{N-1} \Biggl \{ C_A T_F^2 N_F\nonumber \\&\quad \times \left[ -\frac{572}{729} + \frac{160}{27} \zeta _2 + \frac{64}{27} \zeta _3 \right] \nonumber \\&\quad +\,\, C_F T_F^2 N_F \left[ \frac{45468}{729} -\frac{512}{27} \zeta _2 + \frac{128}{27} \zeta _3 \right] \Biggr \}~[38]\end{aligned}$$

69 $$\begin{aligned}&N_F \hat{\tilde{C}}_{2,q}^{\mathsf{PS},(3)}({N_F})(N \rightarrow \infty ) \propto \frac{\ln ^3(\bar{N})}{N^3} C_F T_F^2 N_F \frac{64}{27}\end{aligned}$$

70 $$\begin{aligned}&N_F \hat{\tilde{C}}_{2,g}^{\mathsf{S},(3)}({N_F})(N \rightarrow \infty ) \propto \frac{\ln ^4(\bar{N})}{N}\nonumber \\&\!\!\!\quad \times \,\, \left[ \frac{68}{27} C_F T_F^2 N_F + \frac{28}{27} C_A T_F^2 N_F\right] , \end{aligned}$$

where $\bar{N} = N \exp (\gamma _E)$ and $\gamma _E$ denotes the Euler–Mascheroni constant.

In Fig. 1, we illustrate the contribution of $L_{q,2}^{\mathsf{PS}}$ to the structure function $F_2(x,Q^2)$ using the PDFs of Ref. [21], cf. Eq. (4). Likewise, Figs. 2 and 3 show the corresponding contributions by $L_{g,2}^{\mathsf{S}}$ at $O(a_s^2)$ and $O(a_s^3)$, respectively. The numerical results have been obtained using the representations in $z$-space, cf. Sect. 7, using numerical representations given in [102, 103].^11^ Note that the $O(a_s^2)$-terms, cf. also Ref. [49], are smaller than those at $O(a_s^3)$, which is caused by terms $\propto 1/z$ in the momentum fraction $z$-space representation of the 3-loop contribution to $L_{g,2}^{\mathsf{S}}$, which are absent at 2-loop order.

These contributions emerging at 2- and 3-loop level are minor compared to the values of the structure function $F_2(x,Q^2)$, for which typical values are given in Table 1.^12^ A global comparison of all heavy flavor contributions up to 3-loop order can presently only be performed using the known number of Mellin moments, cf. [39], given in Sect. 6.

**Table 1:** Values of the structure function $F_2(x,Q^2)$ in the low $x$ region using the PDF-parameterization [21]

$x$	$Q^2 = 20~\mathrm{GeV}^2$	$Q^2 = 100~\mathrm{GeV}^2$	$Q^2 = 1000~\mathrm{GeV}^2$
$10^{-4}$	1.946	3.200	5.340
$10^{-3}$	1.141	1.702	2.526
$10^{-2}$	0.641	0.825	1.040
$10^{-1}$	0.400	0.409	0.412

While the expression for $L_{q,2}^{\mathsf{PS}}$ is the same in the $\overline{\mathrm{MS}}$ and on-mass-shell scheme to $O(a_s^3)$, $L_{g,2}^{\mathsf{S}}$, in its 3-loop contribution, changes by the term

71 $$\begin{aligned}&\!\!\!L_{g,2}^{\mathsf{S}, (3),\overline{\mathrm{MS}}}(N)= L_{g,2}^{\mathsf{S}, (3),\mathrm OMS}(N) {+} a_s^3 \frac{32}{3} C_F T_F^2 N_F \left[ 3L_M {-} 4 \right] \nonumber \\&\!\!\!\quad \times \left[ \frac{\big (N^2+N+2\big )}{N (N+1) (N+2)} S_1 +\frac{\big (N^3-4 N^2-N-2\big )}{N^2 (N+1) (N+2)}\right] \nonumber \\ \end{aligned}$$

setting $Q^2 = \mu ^2$. Here, we have identified the logarithms $L_M$ in both schemes symbolically. In applications, either the on-shell or the $\overline{\mathrm{MS}}$ mass has to be used here.

## The logarithmic contributions to $H_{g,2}^{\mathsf{S}}$ and to $H_{q,2}^{\mathsf{PS}}$ at $O(a_s^3)$

The Wilson coefficient $H_{g,2}^{\mathsf{S}}$, except for the constant contribution $a_{Qg}^{(3),0}$, is given by:

$$\begin{aligned}&\!\!\!H_{g,2}^{\mathsf{S}} = \tfrac{1}{2}[1 + (-1)^N] \Biggl \{{a_s} {T_F} \Biggl \{-\tilde{\gamma }_{qg}^{0} L_Q -\frac{4 \big (N^3-4 N^2-N-2\big )}{N^2 (N+1) (N+2)}+\,\,\tilde{\gamma }_{qg}^{0}S_1+\tilde{\gamma }_{qg}^{0} L_M\Biggr \}\nonumber \\&\quad +\,\, {a_s^2} \Biggl \{ {T_F^2} \Biggl [\frac{4}{3} \tilde{\gamma }_{qg}^{0} L_M^2-\frac{4}{3} \tilde{\gamma }_{qg}^{0} L_Q L_M+\,\,\Biggl [\frac{4}{3} \tilde{\gamma }_{qg}^{0} S_1 -\frac{16 \big (N^3-4 N^2-N-2\big )}{3 N^2 (N+1) (N+2)}\Biggr ] L_M\Biggr ]\nonumber \\&\quad +\,\,{C_F} {T_F} \Biggl [ \Biggl [\frac{2 \big (N^2+N+2\big ) \big (3 N^2+3 N+2\big )}{N^2 (N+1)^2 (N+2)}+2 \tilde{\gamma }_{qg}^{0} S_1\Biggr ] L_Q^2+\Biggl [ -\frac{4 P_{53}}{N^3 (N+1)^3 (N+2)}\nonumber \\&\quad +\,\,\frac{4 \big (3 N^4+2 N^3-9 N^2-16 N-12\big ) S_1}{N^2 (N+1)^2 (N+2)}+\,\,L_M \Biggl [ -\frac{4 \big (N^2+N+2\big ) \big (3 N^2+3 N+2\big )}{N^2 (N+1)^2 (N+2)}-\,\,4\tilde{\gamma }_{qg}^{0} S_1\Biggr ] \nonumber \\&\quad +\tilde{\gamma }_{qg}^{0} \big (4 S_2-4 S_1^2\big )\Biggr ] L_Q-\,\,\frac{2 \big (9 N^4+6 N^3-15 N^2-28 N-20\big ) S_1^2}{N^2 (N+1)^2 (N+2)}\nonumber \\&\quad +\,\,\frac{16 (-1)^N P_{194}}{15 (N-2) (N-1)^2 N^2 (N+1)^4 (N+2)^4 (N+3)^3}+\,\,L_M^2 \Biggl [\frac{2 \big (N^2+N+2\big ) \big (3 N^2+3 N+2\big )}{N^2 (N+1)^2 (N+2)}\nonumber \\&\quad +\,\,2 \tilde{\gamma }_{qg}^{0} S_1\Biggr ] +\frac{4P_{207}}{15 (N-1)^2 N^4 (N+1)^4 (N+2)^4 (N+3)^3}+\,\,\frac{4 P_{48} S_1}{N^3 (N+1)^3 (N+2)}+\,\,L_M \Biggl [ \frac{4 P_{53}}{N^3 (N+1)^3 (N+2)}\nonumber \\&\quad -\,\,\frac{4 \big (3 N^4+2 N^3-9 N^2-16 N-12\big ) S_1}{N^2 (N+1)^2 (N+2)}+\,\,\tilde{\gamma }_{qg}^{0} \Bigl [4 S_1^2-4 S_2\Bigr ]\Biggr ]+\,\,\frac{2 \big (11 N^4+42 N^3+43 N^2-4 N-12\big ) S_2}{N^2 (N+1)^2 (N+2)}\nonumber \\ \end{aligned}$$

$$\begin{aligned}&\quad +\,\,\frac{16 P_{45} S_{-2}}{(N-2) N^2 (N+1)^2 (N+2) (N+3)}+\,\,\tilde{\gamma }_{qg}^{0} \Bigl [2 S_1^3-2 S_2 S_1-4 S_{2,1}\Bigr ]+\,\,\frac{128 S_1 S_{-2}+64 S_{-3}-128 S_{-2,1}}{N (N+1) (N+2)}\nonumber \\&\quad +\,\,\frac{(N-1) \Bigl [48 \zeta _3-16 S_3\Bigr ]}{N (N+1)}\Biggr ]+\,\,{C_A} {T_F} \Biggl [ \Biggl [\frac{16 \big (N^2+N+1\big ) \big (N^2+N+2\big )}{(N-1) N^2 (N+1)^2 (N+2)^2}+2 \tilde{\gamma }_{qg}^{0} S_1\Biggr ] L_Q^2\nonumber \\&\quad +\,\,\Biggl [ -\frac{32 (-1)^N \big (N^3+4 N^2+7 N+5\big )}{(N{+}1)^3 (N+2)^3}{+}\frac{8 P_{102}}{(N-1) N^3 (N+1)^2 (N+2)^3}+\,\,\frac{16 \big (N^2+1\big ) \big (N^2-4 N-1\big ) S_1}{(N-1) N (N+1)^2 (N+2)}\nonumber \\&\quad +\tilde{\gamma }_{qg}^{0} \Bigl [-2 S_1^2+2 S_2+4 S_{-2}\Bigr ]\Biggr ] L_Q-\,\,\frac{16 P_{107} S_1}{(N-1) N^3 (N+1)^2 (N+2)^3}-\,\,\frac{4 \big (2 N^5-3 N^4-5 N^3-3 N^2-33 N-6\big ) S_1^2}{(N-1) N (N+1)^2 (N+2)^2}\nonumber \\&\quad -\,\,\frac{16 (-1)^N P_{109}}{3 (N-1) N^2 (N+1)^4 (N+2)^4}-\,\,\frac{8 P_{181}}{3 (N-1) N^4 (N+1)^4 (N+2)^4} +\,\,\frac{32 (-1)^N \big (N^3+4 N^2+7 N+5\big ) S_1}{(N+1)^3 (N+2)^3}\nonumber \\&\quad +\,\,L_M^2 \Biggl [ -\frac{16 \big (N^2+N+1\big ) \big (N^2+N+2\big )}{(N-1) N^2 (N+1)^2 (N+2)^2} -2 \tilde{\gamma }_{qg}^{0} S_1\Biggr ]+\,\,\frac{4 P_{47} S_2}{(N-1) N^2 (N+1)^2 (N+2)^2} +\frac{8 \big (3 N^2+3 N+2\big ) S_3}{N (N+1) (N+2)}\nonumber \\&\quad +\,\,L_M \Biggl [ \frac{32 (-1)^N \big (N^3+4 N^2+7 N+5\big )}{(N+1)^3 (N+2)^3}-\,\,\frac{8 P_{103}}{(N-1) N^3 (N+1)^2 (N+2)^3} -\frac{32 (2 N+3) S_1}{(N+1)^2 (N+2)^2}\nonumber \\&\quad +\,\,\tilde{\gamma }_{qg}^{0} \Bigl [-2 S_1^2-2 S_2-4 S_{-2}\Bigr ]\Biggr ]+\,\,\frac{16 \big (N^5-N^4-5 N^3+3 N^2+14 N+12\big ) S_{-2}}{(N-1) N (N+1)^2 (N+2)^2}\nonumber \\&\quad -\,\,\frac{16\big (N^2+N+4\big ) S_{-3}}{N (N+1) (N+2)}+\,\,\frac{(N-1) \Bigl [16 S_{-2} S_1-16 S_{-2,1}\Bigr ]}{N (N+1)} +\frac{\big (N^2-N-4\big ) 16 (-1)^N S_{-2} }{(N+1)^2 (N+2)^2}-\,\,\frac{6 \big (5 N^2+5 N-6\big ) \zeta _3}{N (N+1) (N+2)}\nonumber \end{aligned}$$

$$\begin{aligned}&\quad +\,\,\tilde{\gamma }_{qg}^{0} \Bigl [-8 S_1 S_2-4 (-1)^N S_{-2} S_1-2 (-1)^N S_{-3}+\,\,4 S_{2,1} -\frac{3}{2} (-1)^N \zeta _3\Bigr ]\Biggr ]\Biggr \}\nonumber \\&\quad +\,\,{a_s^3} \Biggl \{ {T_F^3} \Biggl [ \frac{16}{9} \tilde{\gamma }_{qg}^{0} L_M^3 -\frac{16}{9} \tilde{\gamma }_{qg}^{0} L_Q L_M^2 +\Biggl [ \frac{16}{9} \tilde{\gamma }_{qg}^{0} S_1-\,\,\frac{64 \big (N^3-4 N^2-N-2\big )}{9 N^2 (N+1) (N+2)}\Biggr ] L_M^2 -\frac{16 \tilde{\gamma }_{qg}^{0} \zeta _3}{9}\Biggr ]\nonumber \\&\quad +\,\,{C_A} {T_F^2} \Biggl [ \Biggl [ \frac{64 \big (N^2+N+1\big ) \big (N^2+N+2\big )}{9 (N-1) N^2 (N+1)^2 (N+2)^2} +\frac{8}{9} \tilde{\gamma }_{qg}^{0} S_1\Biggr ] L_Q^3+\,\,\Biggl [ -\frac{64 (-1)^N \big (N^3+4 N^2+7 N+5\big )}{3 (N+1)^3 (N+2)^3}\nonumber \\&\quad +\,\,\frac{8 P_{136}}{9 (N-1) N^3 (N+1)^3 (N+2)^3} +\,\,\frac{32 \big (8 N^4-7 N^3+5 N^2-17 N-13\big ) S_1}{9 (N-1) N (N+1)^2 (N+2)}\nonumber \\&\quad +\,\,L_M \Biggl [\frac{64 \big (N^2+N+1\big ) \big (N^2+N+2\big )}{3 (N-1) N^2 (N+1)^2 (N+2)^2} +\frac{8}{3} \tilde{\gamma }_{qg}^{0} S_1\Biggr ]+\tilde{\gamma }_{qg}^{0} \Bigl [ -\frac{4}{3} S_1^2 +\frac{4 S_2}{3} +\frac{8}{3} S_{-2}\Bigr ]\Biggr ] L_Q^2 \nonumber \\&\quad +\,\,\Biggl [ {-}\frac{32 \big (8 N^4-7 N^3{+}5 N^2-17 N{-}13\big ) S_1^2}{9 (N-1) N (N{+}1)^2 (N+2)}+\frac{128 (-1)^N \big (N^3+4 N^2{+}7 N{+}5\big ) S_1}{3 (N+1)^3 (N{+}2)^3}-\,\,\frac{32 P_{128} S_1}{27 (N-1) N^2 (N+1)^3 (N+2)^3}\nonumber \\&\quad +\frac{64 ({-}1)^N P_{112}}{9 (N-1) N^2 (N{+}1)^4 (N+2)^4}{-}\frac{16 P_{175}}{27 (N{-}1) N^3 (N{+}1)^4 (N{+}2)^4}{+}L_M^2\Biggr [\frac{64 \big (N^2+N{+}1\big ) \big (N^2+N+2\big )}{3 (N-1) N^2 (N+1)^2 (N+2)^2} +\frac{8}{3} \tilde{\gamma }_{qg}^{0} S_1\Biggr ]\nonumber \\ \end{aligned}$$

$$\begin{aligned}&\quad +\,\,\frac{32 \big (8 N^4+13 N^3-22 N^2-9 N-26\big ) S_2}{9 (N-1) N (N+1) (N+2)^2}+\,\,\frac{64 \big (8 N^5+15 N^4+6 N^3+11 N^2+16 N+16\big ) S_{-2}}{9 (N-1) N (N+1)^2 (N+2)^2}\nonumber \\&\quad +\,\,\frac{128 \big (N^2+N-1\big ) S_3}{9 N (N+1) (N+2)}+\,\,L_M \Biggl [ -\frac{128 (-1)^N \big (N^3+4 N^2+7 N+5\big )}{3 (N+1)^3 (N+2)^3}+\,\,\frac{32 P_{137}}{9 (N-1) N^3 (N+1)^3 (N+2)^3}\nonumber \\&\quad -\,\,\frac{64 (2 N-1) \big (N^3+9 N^2+7 N+7\big ) S_1}{9 (N-1) N (N+1)^2 (N+2)}+\,\,\tilde{\gamma }_{qg}^{0} \Bigl [ -\frac{8}{3} S_1^2 +\frac{8 S_2}{3} +\frac{16}{3} S_{-2}\Bigr ]\Biggr ] -\frac{128 \big (N^2+N+3\big ) S_{-3}}{3 N (N+1) (N+2)}\nonumber \\&\quad +\,\,\tilde{\gamma }_{qg}^{0} \Bigl [\frac{8}{9} S_1^3-8 S_2 S_1 +\frac{32}{3} S_{2,1}\Bigr ]+\,\,\frac{256 S_{-2,1}}{3 N (N+1) (N+2)} +\frac{(N-1) \Bigl [\frac{64}{3} S_{-2} S_1 -32 \zeta _3\Bigr ]}{N (N+1)}\Biggr ] L_Q \nonumber \\&\quad +\,\,\frac{32 \big (N^3+8 N^2+11 N+2\big ) S_1^3}{9 N (N+1)^2 (N+2)^2} +\frac{8 P_{94} S_1^2}{27 N (N+1)^3 (N+2)^3}+\,\,\frac{4}{9} \frac{P_{143} \zeta _2}{(N-1) N^3 (N+1)^3 (N+2)^3}\nonumber \\ \end{aligned}$$

$$\begin{aligned}&\quad +\,\,\frac{8 P_{206}}{81 (N-1) N^5 (N+1)^5 (N+2)^5}+\,\,\frac{32}{3} \big (2 N^4+3 N^3+10 N^2+37 N+35\big ) \frac{(-1)^N \zeta _2}{(N+1)^3 (N+2)^3}\nonumber \\&\quad +\,\,\frac{32}{9} \frac{ \big (9 N^5-4 N^4+N^3+92 N^2+42 N+28\big ) \zeta _3}{(N-1) N^2 (N+1)^2 (N+2)^2}-\,\,\frac{8 P_{191} S_1}{81 (N-1) N^4 (N+1)^4 (N+2)^4}\nonumber \\&\quad -\,\,\frac{32}{9} \frac{\big (5 N^4+8 N^3+17 N^2+43 N+20\big ) \zeta _2}{N (N+1)^2 (N+2)^2} S_1+\,\,L_M^3 \Biggl [ -\frac{448 \big (N^2+N+1\big ) \big (N^2+N+2\big )}{9 (N-1) N^2 (N+1)^2 (N+2)^2}-\,\,\frac{56}{9} \tilde{\gamma }_{qg}^{0} S_1 \Biggr ]\nonumber \\&\quad +\frac{8 P_{134} S_2}{3 (N-1) N^3 (N+1)^3 (N+2)^3}-\,\,\frac{32 \big (3 N^3-12 N^2-27 N-2\big ) S_1 S_2}{3 N (N+1)^2 (N+2)^2}\nonumber \\&\quad +\,\,\frac{256 \big (N^5+10 N^4+9 N^3+3 N^2+7 N+6\big ) S_3}{9 (N-1) N^2 (N+1)^2 (N+2)^2}+\,\,L_M^2 \Biggl [ \frac{64 (-1)^N \big (N^3+4 N^2+7 N+5\big )}{(N+1)^3 (N+2)^3}\nonumber \\&\quad -\,\,\frac{8 P_{141}}{9 (N-1) N^3 (N+1)^3 (N+2)^3}+\,\,\frac{32 \big (8 N^5+9 N^4-57 N^3-31 N^2+25 N-26\big ) S_1}{9 (N-1) N (N+1)^2 (N+2)^2}\nonumber \\&\quad +\,\,\tilde{\gamma }_{qg}^{0} \Bigl [-\frac{20}{3} S_1^2-4 S_2-8 S_{-2}\Bigr ]\Biggr ]+\,\,\frac{128 (-1)^N \big (N^4+2 N^3+7 N^2+22 N+20\big ) S_{-2}}{3 (N+1)^3 (N+2)^3}\nonumber \end{aligned}$$

$$\begin{aligned}&\quad +\,\,\frac{\big (N^2-N-4\big ) }{(N+1)^2 (N+2)^2} \Bigl [ -\frac{256}{3} (-1)^N S_1 S_{-2} -\frac{128}{3} (-1)^N S_{-3}+\,\,\frac{256}{3} S_{-2,1} -\frac{128}{3} (-1)^N S_1 \zeta _2\nonumber \\&\quad -\,\,32 (-1)^N \zeta _3\Bigr ] +\tilde{\gamma }_{qg}^{0} \Bigl [\frac{2}{9} S_1^4 +\frac{20}{3} S_2 S_1^2+\frac{32}{3} (-1)^N S_{-3} S_1+\,\,\Big (\frac{160 S_3}{9} -\frac{64}{3} S_{-2,1}\Big ) S_1 +\frac{2}{3} S_2^2\nonumber \\&\quad +\,\,\frac{8}{9} \big (-2+9 (-1)^N\big ) \zeta _3 S_1 +S_{-2} \Big (\frac{32}{3} (-1)^N S_1^2 +\frac{32}{3} (-1)^N S_2\Big )\nonumber \\&\quad +\,\,12 S_4 +\frac{16}{3} (-1)^N S_{-4} -\frac{16}{3} S_{3,1} -\frac{32}{3} S_{-2,2} -\frac{32}{3} S_{-3,1}-\,\,\frac{16}{3} S_{2,1,1} +\frac{64}{3} S_{-2,1,1} +\Bigl [\frac{2}{3} \big (-3+8 (-1)^N\big ) S_1^2\nonumber \\&\quad +\,\,\frac{2}{3} \big (-3+8 (-1)^N\big ) S_2 +\frac{4}{3} \big (1+4 (-1)^N\big ) S_{-2}\Bigr ] \zeta _2\Bigr ]\quad +\,\,L_M \Biggl [\frac{64 \big (N^5+9 N^4+3 N^3+N^2+26 N-4\big ) S_1^2}{9 (N-1) N (N+1)^2 (N+2)^2}\nonumber \\&\quad +\,\,\frac{128 (-1)^N \big (N^3+4 N^2+7 N+5\big ) S_1}{3 (N+1)^3 (N+2)^3}+\,\,\frac{16 P_{140} S_1}{27 (N-1) N^3 (N+1)^3 (N+2)^3} +\frac{64 (N-1) S_{-2} S_1}{3 N (N+1)}\nonumber \\&\quad -\,\,\frac{64 (-1)^N P_{99}}{9 (N-1) N^2 (N+1)^3 (N+2)^4}+\,\,\frac{16 P_{184}}{27 (N-1) N^4 (N+1)^4 (N+2)^4} +\frac{64 \big (7 N^2+7 N+8\big ) S_3}{9 N (N+1) (N+2)}\nonumber \\&\quad -\,\,\frac{64 P_{46} S_2}{9 (N-1) N^2 (N+1)^2 (N+2)^2}-\,\,\frac{64 \big (2 N^5+21 N^4+36 N^3-7 N^2-68 N-56\big ) S_{-2}}{9 (N-1) N (N+1)^2 (N+2)^2}\nonumber \\ \end{aligned}$$

$$\begin{aligned}&\quad -\,\,\frac{128 S_{-3}}{3 N (N+1) (N+2)} -\frac{128 S_{-2,1}}{3 (N+2)}+\,\,\frac{\big (N^2-N-4\big ) \frac{128}{3} (-1)^N S_{-2} }{(N+1)^2 (N+2)^2} -\frac{16 \big (3 N^2+3 N-2\big ) \zeta _3}{N (N+1) (N+2)}\nonumber \\&\quad +\tilde{\gamma }_{qg}^{0} \Bigl [ {-}\frac{8}{9} S_1^3 {-}\frac{40}{3} S_2 S_1 {-}\frac{32}{3} (-1)^N S_{-2} S_1-\frac{16}{3} (-1)^N S_{-3}{-}4 ({-}1)^N \zeta _3\Bigr ] \Biggr ] \Biggr ]{+}{C_A} {T_F^2}{N_F} \Biggl [ \Biggl [ \frac{64 \big (N^2{+}N{+}1\big ) \big (N^2{+}N{+}2\big )}{9 (N{-}1) N^2 (N{+}1)^2 (N+2)^2} \nonumber \\&\quad +\,\,\frac{8}{9} \tilde{\gamma }_{qg}^{0} S_1\Biggr ] L_Q^3 +\Biggl [ -\frac{64 (-1)^N \big (N^3+4 N^2+7 N+5\big )}{3 (N+1)^3 (N+2)^3}+\,\,\frac{8 P_{136}}{9 (N-1) N^3 (N+1)^3 (N+2)^3} \nonumber \\&\quad +\,\, \frac{32 \big (8 N^4{-}7 N^3+5 N^2{-}17 N{-}13\big ) S_1}{9 (N{-}1) N (N{+}1)^2 (N{+}2)} {+}\tilde{\gamma }_{qg}^{0} \Bigl [ {-}\frac{4}{3} S_1^2+\frac{4 S_2}{3}+\,\,\frac{8}{3} S_{-2}\Bigr ] \Biggr ] L_Q^2 {+}\Biggl [ -\frac{32 \big (8 N^4-7 N^3{+}5 N^2-17 N-13\big ) S_1^2}{9 (N-1) N (N{+}1)^2 (N+2)}\nonumber \\&\quad +\,\,\frac{128 (-1)^N \big (N^3+4 N^2+7 N+5\big ) S_1}{3 (N+1)^3 (N+2)^3}-\,\,\frac{16 P_{129} S_1}{27 (N-1) N^2 (N+1)^3 (N+2)^3}+\,\,\frac{64 (-1)^N P_{112}}{9 (N-1) N^2 (N+1)^4 (N+2)^4} \nonumber \\ \end{aligned}$$

$$\begin{aligned}&\quad +\frac{128 \big (N^2+N-1\big ) S_3}{9 N (N+1) (N+2)}-\,\,\frac{8 P_{190}}{27 (N-1) N^4 (N+1)^4(N+2)^4}+\,\,\frac{32 \big (8 N^4+13 N^3-22 N^2-9 N-26\big ) S_2}{9 (N-1)N (N+1) (N+2)^2}\nonumber \\&\quad +\frac{64 \big (8 N^5+15 N^4+6 N^3+11 N^2+16 N+16\big ) S_{-2}}{9 (N-1) N (N+1)^2 (N+2)^2} -\frac{128 \big (N^2+N+3\big ) S_{-3}}{3 N (N+1) (N+2)}+\tilde{\gamma }_{qg}^{0} \Bigl [\frac{8}{9} S_1^3-8 S_2 S_1+\frac{32}{3} S_{2,1}\Bigr ]\nonumber \\&\quad +\frac{256 S_{-2,1}}{3 N (N+1) (N+2)}+\,\,\frac{(N-1) \Bigl [\frac{64}{3} S_{-2} S_1-32 \zeta _3\Bigr ]}{N (N+1)}\Biggr ] L_Q+\,\,\frac{16 \big (N^3+8 N^2+11 N+2\big ) S_1^3}{9 N (N+1)^2 (N+2)^2} +\frac{8 P_{44} S_1^2}{3 N (N+1)^3 (N+2)^3}\nonumber \\&\quad +\frac{4}{9} \frac{P_{117}\zeta _2}{(N-1) N^3 (N+1)^3 (N+2)^2} \!+\!\frac{16 P_{203}}{3 (N\!-\!1) N^5 (N\!+\!1)^5 (N\!+\!2)^5}\!+\!\frac{16}{9} \frac{\big (9 N^5-14 N^4-19 N^3+52 N^2+12 N+8\big )\zeta _3}{(N-1) N^2 (N+1)^2 (N+2)^2}\nonumber \\&\quad -\,\,\frac{16 P_{108} S_1}{3 N (N+1)^4 (N+2)^4}+\,\,\frac{16}{9}\frac{\big (5 N^4+32 N^3+47 N^2+28 N+20\big ) \zeta _2}{N (N+1)^2 (N+2)^2} S_1\nonumber \\&\quad +\,\,L_M^3 \Biggl [ -\frac{64 \big (N^2+N+1\big ) \big (N^2+N+2\big )}{9 (N-1) N^2 (N+1)^2 (N+2)^2} -\frac{8}{9} \tilde{\gamma }_{qg}^{0} S_1\Biggr ]+\,\,\frac{8 P_{133} S_2}{3 (N-1) N^3 (N+1)^3 (N+2)^3}\nonumber \\&\quad -\,\,\frac{16 \big (3 N^3-12 N^2-27 N-2\big ) S_1 S_2}{3 N (N+1)^2 (N+2)^2}+\,\,\frac{128 \big (N^5+10 N^4+9 N^3+3 N^2+7 N+6\big ) S_3}{9 (N-1) N^2 (N+1)^2 (N+2)^2}\nonumber \end{aligned}$$

$$\begin{aligned}&\quad +\,\,L_M^2 \Biggl [ \frac{64 (-1)^N \big (N^3+4 N^2+7 N+5\big )}{3 (N+1)^3 (N+2)^3}-\,\,\frac{8 P_{114}}{9 (N-1) N^2 (N+1)^3 (N+2)^3}\nonumber \\&\quad -\,\,\frac{32 \big (5 N^4+20 N^3+47 N^2+58 N+20\big ) S_1}{9 N (N+1)^2 (N+2)^2} +\tilde{\gamma }_{qg}^{0} \Bigl [ -\frac{4}{3} S_1^2 -\frac{4 S_2}{3}-\,\,\frac{8}{3} S_{-2}\Bigr ]\Biggr ]\nonumber \\&\quad +\frac{\big (N^4+2 N^3+7 N^2+22 N+20\big ) \Bigl [\frac{64}{3} (-1)^N S_{-2}+\frac{32}{3} (-1)^N \zeta _2\Bigr ]}{(N+1)^3 (N+2)^3}\nonumber \\&\quad +\,\,\frac{\big (N^2-N-4\big ) \Bigl [ -\frac{128}{3} (-1)^N S_1 S_{-2} -\frac{64}{3} (-1)^N S_{-3} +\frac{128}{3} S_{-2,1} -\frac{64}{3} (-1)^N S_1 \zeta _2-16 (-1)^N \zeta _3\Bigr ]}{(N+1)^2 (N+2)^2}\nonumber \\&\quad +\,\,\tilde{\gamma }_{qg}^{0} \Bigl [\frac{1}{9} S_1^4+\frac{10}{3} S_2 S_1^2+\frac{16}{3} (-1)^N S_{-3} S_1+\,\,\Bigl [\frac{80 S_3}{9} -\frac{32}{3} S_{-2,1}\Bigr ] S_1+\frac{4}{9} \big (-7+9 (-1)^N\big ) \zeta _3 S_1+\frac{1}{3} S_2^2\nonumber \\&\quad +\,\,S_{-2} \Bigl [\frac{16}{3} (-1)^N S_1^2+\frac{16}{3} (-1)^N S_2\Bigr ] +6 S_4+\frac{8}{3} (-1)^N S_{-4} -\frac{8}{3} S_{3,1}-\frac{16}{3} S_{-2,2} -\frac{16}{3} S_{-3,1} -\frac{8}{3} S_{2,1,1}+\,\,\frac{32}{3} S_{-2,1,1} \nonumber \\&\quad +\Bigl [\frac{4}{3} \big (-1+2 (-1)^N\big ) S_1^2+\frac{4}{3} \big (-1+2 (-1)^N\big ) S_2 +\,\,\frac{8}{3} (-1)^N S_{-2}\Bigr ] \zeta _2\Bigr ]\nonumber \\ \end{aligned}$$

$$\begin{aligned}&\quad +L_M \Biggl [ -\frac{16 \big (10 N^4+43 N^3+106 N^2+131 N+46\big ) S_1^2}{9 N (N+1)^2 (N+2)^2}+\,\,\frac{16 P_{82} S_1}{27 N (N+1)^3 (N+2)^3}\nonumber \\&\quad -\frac{64 (-1)^N \big (7 N^5+43 N^4+117 N^3+166 N^2+107 N+16\big )}{9 (N+1)^4 (N+2)^4}+\,\,\frac{8 P_{188}}{27 (N-1) N^4 (N+1)^4 (N+2)^4}\nonumber \\&\quad -\,\,\frac{16 P_{64} S_2}{9 (N-1) N^2 (N+1)^2 (N+2)^2} -\frac{64 \big (5 N^2+8 N+10\big ) S_{-2}}{9 N (N+1) (N+2)}+\,\,\frac{\big (N^2-N-4\big ) \frac{64}{3} (-1)^N S_{-2} }{(N+1)^2 (N+2)^2}\nonumber \\&\quad +\,\,\tilde{\gamma }_{qg}^{0} \Bigl [ -\frac{8}{9} S_1^3 -\frac{8}{3} S_2 S_1 -\frac{16}{3} (-1)^N S_{-2} S_1 -\frac{40 S_3}{9} -\,\,\frac{8}{3}\big (2+(-1)^N\big ) S_{-3} -\frac{16}{3} S_{2,1} +\frac{16}{3} S_{-2,1} \Bigr ]\Biggr ]\Biggr ]\nonumber \\&\quad +{C_F^2} {T_F} \Biggl [ -\frac{\big (15 N^4+6 N^3-25 N^2-32 N-28\big ) S_1^4}{3 N^2 (N+1)^2 (N+2)}+\frac{2 P_{101} S_1^2}{N^3 (N+1)^4 (N+2)}\nonumber \\&\quad -\,\,\frac{4 \big (3 N^5-47 N^4-147 N^3-93 N^2+8 N+12\big ) S_1^3}{3 N^3 (N+1)^2 (N+2)}- 4 \frac{\big (3 N^4+14 N^3+43 N^2+48 N+20\big ) \zeta _2}{N^2 (N+1)^2 (N+2)} S_1^2\nonumber \\&\quad -\,\,\frac{2 \big (5 N^4-14 N^3+53 N^2+120 N+28\big ) S_2 S_1^2}{N^2 (N+1)^2 (N+2)}-\,\,\frac{2 P_{153} S_1}{N^5 (N+1)^5 (N+2)}-\,\,\frac{4 P_{60} S_2 S_1}{N^3 (N+1)^3 (N+2)}\nonumber \\ \end{aligned}$$

$$\begin{aligned}&\quad -\,\,\frac{8 \big (3 N^4+90 N^3+83 N^2-44 N-4\big ) S_3 S_1}{3 N^2 (N+1)^2 (N+2)}-\,\,\frac{16 \big (3 N^4+2 N^3+19 N^2+28 N+12\big ) S_{2,1} S_1}{N^2 (N+1)^2 (N+2)}-\,\,\frac{8 P_{74} \zeta _2 S_1}{N^3 (N+1)^3 (N+2)}\nonumber \\&\quad -\,\,\frac{\big (11 N^4+142 N^3+147 N^2-32 N-12\big ) S_2^2}{N^2 (N+1)^2 (N+2)}-\,\,\frac{P_{189}}{N^6 (N+1)^6 (N+2)}+\,\,16 \frac{(-1)^N \big (N^2+N+2\big ) \zeta _2}{N (N+1)^4 (N+2)}\nonumber \\&\quad +\,\,\frac{2}{3} \big (N^2+N+2\big ) \frac{\big (153 N^4+306 N^3+165 N^2+12 N+4\big ) \zeta _3}{N^3 (N+1)^3 (N+2)}+\,\,L_Q^3 \Biggl [\frac{2 \big (N^2+N+2\big ) \big (3 N^2+3 N+2\big )^2}{3 N^3 (N+1)^3 (N+2)}\nonumber \\&\quad -\,\,\frac{16 \big (N^2+N+2\big ) S_1 \big (3 N^2+3 N+2\big )}{3 N^2 (N+1)^2 (N+2)}-\,\,\frac{8}{3} \tilde{\gamma }_{qg}^{0} S_1^2\Biggr ] +L_M^3 \Biggl [ -\frac{2 \big (N^2+N+2\big ) \big (3 N^2+3 N+2\big )^2}{3 N^3 (N+1)^3 (N+2)}\nonumber \\&\quad +\,\,\frac{16 \big (N^2+N+2\big ) S_1 \big (3 N^2+3 N+2\big )}{3 N^2 (N+1)^2 (N+2)}+\,\,\frac{8}{3} \tilde{\gamma }_{qg}^{0} S_1^2\Biggr ] -\frac{2 P_{124} S_2}{N^4 (N+1)^4 (N+2)}\nonumber \\&\quad +\,\, \frac{4 \big (21 N^5+217 N^4+415 N^3+351 N^2+152 N-4\big ) S_3}{3 N^2 (N+1)^3 (N+2)}-\,\,\frac{16 \big (N^2-3 N-2\big ) \big (3 N^2+3 N+2\big ) S_{2,1}}{N^3 (N+1)^2 (N+2)}\nonumber \end{aligned}$$

$$\begin{aligned}&\quad +\,\,L_M^2 \Biggl [ \frac{32 \big (N^3+5 N^2+6 N+4\big ) S_1^2}{N^2 (N+1)^2 (N+2)}+\,\,\frac{2 P_{80} S_1}{N^3 (N+1)^3 (N+2)} -\frac{32 (-1)^N \big (N^2+N+2\big )}{N (N+1)^4 (N+2)}-\frac{P_{120}}{N^4 (N+1)^4 (N+2)}\nonumber \\&\quad -\,\,\frac{16 \big (N^2+N+2\big ) \big (3 N^2+3 N+2\big ) S_2}{N^2 (N+1)^2 (N+2)} -\frac{32 \big (N^2+N+2\big ) S_{-2}}{N^2 (N+1)^2 (N+2)}+\tilde{\gamma }_{qg}^{0} \Bigl [8 S_1^3-16 S_2 S_1 -\,\,16 S_{-2} S_1-8 S_3\nonumber \\&\quad -8 S_{-3}+16 S_{-2,1}\Bigr ]\Biggr ] +\,\,L_Q^2\Biggl [ \frac{32 \big (N^3+5 N^2+6 N+4\big ) S_1^2}{N^2 (N+1)^2 (N+2)}+\frac{2 P_{80} S_1}{N^3 (N+1)^3 (N+2)}-\,\,\frac{32 (-1)^N \big (N^2+N+2\big )}{N (N+1)^4 (N+2)}\nonumber \\&\quad -\frac{P_{120}}{N^4 (N+1)^4 (N+2)} +\,\,L_M\Biggl [ -\frac{2 \big (N^2+N+2\big ) \big (3 N^2+3 N+2\big )^2}{N^3 (N+1)^3 (N+2)}+\,\,\frac{16 \big (N^2+N+2\big ) S_1 \big (3 N^2+3 N+2\big )}{N^2 (N+1)^2 (N+2)}\nonumber \\&\quad +8 \tilde{\gamma }_{qg}^{0} S_1^2\Biggr ]-\,\,\frac{16 \big (N^2+N+2\big ) \big (3 N^2+3 N+2\big ) S_2}{N^2 (N+1)^2 (N+2)}-\,\,\frac{32 \big (N^2+N+2\big ) S_{-2}}{N^2 (N+1)^2 (N+2)}+\,\,\tilde{\gamma }_{qg}^{0}\Bigl [8 S_1^3-16 S_2 S_1-16 S_{-2} S_1\nonumber \\&\quad -8 S_3-8 S_{-3}+16 S_{-2,1}\Bigr ]\Biggr ] +\,\,\frac{P_{132} \zeta _2}{2 N^4 (N+1)^4 (N+2)}+\frac{16 \big (N^2+N+2\big ) S_{-2} \zeta _2}{N^2 (N+1)^2 (N+2)}+\,\,96\tilde{\gamma }_{qg}^{0} \log (2) \zeta _2\nonumber \\ \end{aligned}$$

$$\begin{aligned}&\quad +\,\,\frac{\big (N^2+N+2\big ) \big (3 N^2+3 N+2\big ) \Bigl [6 S_4-16 S_{3,1}+32 S_{2,1,1}+8 S_2 \zeta _2 -\frac{16}{3} S_1 \zeta _3\Bigr ]}{N^2 (N+1)^2 (N+2)}\nonumber \\&\quad +\,\,\tilde{\gamma }_{qg}^{0} \Bigl [\frac{1}{3} S_1^5 -\frac{2}{3} S_2 S_1^3+\Big ( -\frac{16}{3} S_3-16 S_{2,1}\Big ) S_1^2-\,\,\frac{8}{3} \zeta _3 S_1^2 +\Bigl [-3 S_2^2+6 S_4-16 S_{3,1}+32 S_{2,1,1}\Bigr ] S_1\nonumber \\&\quad +\,\,\frac{8}{3} S_2 S_3 +\Bigl [-4 S_1^3+8 S_2 S_1+8 S_{-2} S_1+4 S_3+4 S_{-3} -\,\,8 S_{-2,1}\Bigr ] \zeta _2\Bigr ] \nonumber \\&\quad +\,\,L_Q \Biggl [ \frac{16 \big (3 N^4-13 N^2-18 N-12\big ) S_1^3}{N^2 (N+1)^2 (N+2)} -\frac{2 P_{78} S_1^2}{N^3 (N+1)^3 (N+2)}+\,\,\frac{64 (-1)^N \big (N^2+N+2\big ) S_1}{N (N+1)^4 (N+2)} \nonumber \\&\quad -\,\,\frac{2 P_{81} S_1}{N^4 (N+1)^3} -\frac{16 \big (7 N^4+20 N^3+7 N^2-22 N-20\big ) S_2 S_1}{N^2 (N+1)^2 (N+2)} -\,\,\frac{64 \big (2 N^3+N^2+3 N-10\big ) S_{-2} S_1}{N^2 (N+1)^2 (N+2)} \nonumber \\&\quad -\,\,\frac{32 (-1)^N P_{208}}{5 (N-2) (N-1)^2 N^3 (N+1)^5 (N+2)^5 (N+3)^3}+\,\,\frac{2 P_{219}}{5 (N-1)^2 N^5 (N+1)^5 (N+2)^5 (N+3)^3} \nonumber \\&\quad -\,\,48 \frac{\big (9 N^4+10 N^3+9 N^2-8 N+12\big ) \zeta _3}{N^2 (N+1)^2 (N+2)} +\,\,L_M^2 \Biggl [ \frac{2 \big (N^2+N+2\big ) \big (3 N^2+3 N+2\big )^2}{N^3 (N+1)^3 (N+2)} \nonumber \\ \end{aligned}$$

$$\begin{aligned}&\quad -\,\,\frac{16 \big (N^2+N+2\big ) S_1 \big (3 N^2+3 N+2\big )}{N^2 (N+1)^2 (N+2)} -8 \tilde{\gamma }_{qg}^{0} S_1^2\Biggr ]+\,\,\frac{2 P_{89} S_2}{N^3 (N+1)^3 (N+2)} \nonumber \\&\quad -\,\,\frac{16 \big (3 N^4+10 N^3+15 N^2+16 N-12\big ) S_3}{N^2 (N+1)^2 (N+2)}+\,\,\frac{128 (-1)^N \big (N^3+4 N^2+7 N+5\big ) S_{-2}}{(N+1)^3 (N+2)^3}\nonumber \\&\quad -\,\,\frac{64 P_{154} S_{-2}}{(N-2) N^3 (N+1)^3 (N+2)^3 (N+3)}-\,\,\frac{64 \big (N^2+3 N+4\big ) S_{-3}}{N (N+1)^2 (N+2)}\nonumber \\&\quad +\,\,\frac{16 \big (N^2+N+2\big ) \big (3 N^2+3 N+2\big ) S_{2,1}}{N^2 (N+1)^2 (N+2)}+\,\,L_M \Biggl [ -\frac{64 \big (N^3+5 N^2+6 N+4\big ) S_1^2}{N^2 (N+1)^2 (N+2)} -\,\,\frac{4 P_{80} S_1}{N^3 (N+1)^3 (N+2)}\nonumber \\&\quad +\frac{64 (-1)^N \big (N^2+N+2\big )}{N (N+1)^4 (N+2)}+\,\,\frac{2 P_{120}}{N^4 (N+1)^4 (N+2)}+\,\,\frac{32 \big (N^2+N+2\big ) \big (3 N^2+3 N+2\big ) S_2}{N^2 (N+1)^2 (N+2)} +\frac{64 \big (N^2+N+2\big ) S_{-2}}{N^2 (N+1)^2 (N+2)} \nonumber \\&\quad +\,\,\tilde{\gamma }_{qg}^{0} \Bigl [-16 S_1^3+32 S_2 S_1+32 S_{-2} S_1+\,\,16 S_3+16 S_{-3}-32 S_{-2,1}\Bigr ]\Biggr ] +\frac{128 (N-1) \big (N^2+2 N+4\big ) S_{-2,1}}{N^2 (N+1)^2 (N+2)}\nonumber \\&\quad +\,\,\tilde{\gamma }_{qg}^{0} \Bigl [-8 S_1^4+24 S_2 S_1^2-48 S_{-3} S_1 +\,\,\Bigl [16 S_{2,1}-32 S_{-2,1}\Bigr ] S_1-48 \zeta _3 S_1-16 S_2^2-32 S_{-2}^2 \nonumber \end{aligned}$$

$$\begin{aligned}&\quad +\,\,S_{-2}\Bigl [32 S_1^2-32 S_2\Bigr ]-32 S_4-80 S_{-4}-32 S_{3,1}+\,\,32 S_{-2,2}+64 S_{-3,1}\Bigr ]\Biggr ] +L_M \Biggl [ -\frac{16 \big (3 N^4-13 N^2-18 N-12\big ) S_1^3}{N^2 (N+1)^2 (N+2)}\nonumber \\&\quad +\,\,\frac{2 P_{78} S_1^2}{N^3 (N+1)^3 (N+2)}-\,\,\frac{64 (-1)^N \big (N^2+N+2\big ) S_1}{N (N+1)^4 (N+2)}+\frac{2 P_{81} S_1}{N^4 (N+1)^3}\nonumber \\&\quad +\,\,\frac{16 \big (7 N^4+20 N^3+7 N^2-22 N-20\big ) S_2 S_1}{N^2 (N+1)^2 (N+2)}+\,\,\frac{64 \big (2 N^3+N^2+3 N-10\big ) S_{-2} S_1}{N^2 (N+1)^2 (N+2)}\nonumber \\&\quad +\frac{32 ({-}1)^N P_{208}}{5 (N{-}2) (N-1)^2 N^3 (N+1)^5 (N+2)^5 (N+3)^3} {-}\frac{2 P_{219}}{5 (N-1)^2 N^5 (N{+}1)^5 (N+2)^5 (N+3)^3}\nonumber \\&\quad +\,\,48 \frac{\big (9 N^4+10 N^3+9 N^2-8 N+12\big ) \zeta _3}{N^2 (N+1)^2 (N+2)} -\,\,\frac{2 P_{89} S_2}{N^3 (N+1)^3 (N+2)}+\,\,\frac{16 \big (3 N^4+10 N^3+15 N^2+16 N-12\big ) S_3}{N^2 (N+1)^2 (N+2)}\nonumber \\&\quad -\,\,\frac{128 (-1)^N \big (N^3+4 N^2+7 N+5\big ) S_{-2}}{(N+1)^3 (N+2)^3}+\,\,\frac{64 P_{154} S_{-2}}{(N-2) N^3 (N+1)^3 (N+2)^3 (N+3)}\nonumber \\&\quad +\,\,\frac{64 \big (N^2+3 N+4\big ) S_{-3}}{N (N+1)^2 (N+2)}-\,\,\frac{16 \big (N^2+N+2\big ) \big (3 N^2+3 N+2\big ) S_{2,1}}{N^2 (N+1)^2 (N+2)} -\,\,\frac{128 (N-1) \big (N^2+2 N+4\big ) S_{-2,1}}{N^2 (N+1)^2 (N+2)}\nonumber \\&\quad +\,\,\tilde{\gamma }_{qg}^{0} \Bigl [8 S_1^4-24 S_2 S_1^2+48 S_{-3} S_1+\Bigl [32 S_{-2,1}-16 S_{2,1}\Bigr ] S_1 +\,\,48 \zeta _3 S_1+16 S_2^2+32 S_{-2}^2 +\,\,S_{-2} \Bigl [32 S_2-32 S_1^2\Bigr ]\nonumber \\ \end{aligned}$$

$$\begin{aligned}&\quad +32 S_4+80 S_{-4}+32 S_{3,1} -32 S_{-2,2} -\,\,64 S_{-3,1}\Bigr ]\Biggr ]\Biggr ] +\,\,{C_A^2} {T_F} \Biggl [ \frac{P_{65} S_1^4}{9 (N-1) N^2 (N+1)^2 (N+2)^2}\nonumber \\&\quad -\,\,\frac{4 P_{118} S_1^3}{9 (N-1) N^2 (N+1)^3 (N+2)^3} -\,\,\frac{4}{3} \frac{P_{73} S_1^2 \zeta _2}{(N-1) N^2 (N+1)^2 (N+2)^2}+\,\,\frac{2 P_{157} S_1^2}{3 (N-1) N^2 (N+1)^4 (N+2)^4} \nonumber \\&\quad +\,\,\frac{2 (N-2) \big (55 N^5+347 N^4+379 N^3+213 N^2+326 N+120\big ) S_2 S_1^2}{3 (N-1) N^2 (N+1)^2 (N+2)^2} -\,\,\frac{4}{9}\frac{P_{159} S_1\zeta _2}{(N-1)^2 N^3 (N+1)^3 (N+2)^3}\nonumber \\&\quad -\,\,\frac{4P_{204} S_1}{3 (N-1) N^5 (N+1)^5 (N+2)^5} -\,\,\frac{4 P_{135} S_2 S_1}{3 (N-1) N^3 (N+1)^3 (N+2)^3}+\,\,\frac{16 P_{86} S_3 S_1}{9 (N-1) N^2 (N+1)^2 (N+2)^2}\nonumber \\&\quad -\,\,\frac{8}{3} \frac{(-1)^N P_{155}\zeta _2}{(N-1) N^3 (N+1)^4 (N+2)^4}-\,\,\frac{2}{9}\frac{P_{193}\zeta _2}{(N-1)^2 N^4 (N+1)^4 (N+2)^4}\nonumber \\&\quad -\,\,\frac{4 \big (11 N^4+22 N^3-35 N^2-46 N-24\big ) P_{203}}{3 (N-1)^2 N^6 (N+1)^6 (N+2)^6}-\,\,\frac{4}{9} \frac{\zeta _3 P_{90} S_1}{(N-1) N^2 (N+1)^2 (N+2)^2}\nonumber \\ \end{aligned}$$

$$\begin{aligned}&\quad -\,\,\frac{4}{9} \frac{\big (11 N^4+22 N^3-35 N^2-46 N-24\big )\big (9 N^5-14 N^4-19 N^3+52 N^2+12 N+8\big )\zeta _3}{(N-1)^2 N^3 (N+1)^3 (N+2)^3}\nonumber \\&\quad +\,\,L_Q^3 \Biggl [ -\frac{8}{3} \tilde{\gamma }_{qg}^{0} S_1^2 +\frac{8 \big (N^2+N+2\big ) \big (11 N^4+22 N^3-59 N^2-70 N-48\big ) S_1}{9 (N-1) N^2 (N+1)^2 (N+2)^2}\nonumber \\&\quad -\,\,\frac{16 \big (N^2+N+1\big ) \big (N^2+N+2\big ) \big (11 N^4+22 N^3-35 N^2-46 N-24\big )}{9 (N-1)^2 N^3 (N+1)^3 (N+2)^3}\Biggr ]+\,\,L_M^3 \Biggl [ \frac{8}{3} \tilde{\gamma }_{qg}^{0} S_1^2 \nonumber \\&\quad -\,\,\frac{8 \big (N^2+N+2\big ) \big (11 N^4+22 N^3-59 N^2-70 N-48\big ) S_1}{9 (N-1) N^2 (N+1)^2 (N+2)^2}\nonumber \\&\quad +\,\,\frac{16 \big (N^2+N+1\big ) \big (N^2+N+2\big ) \big (11 N^4+22 N^3-35 N^2-46 N-24\big )}{9 (N-1)^2 N^3 (N+1)^3 (N+2)^3}\Biggr ]\nonumber \\&\quad -\,\,\frac{2 \big (11 N^4+22 N^3-35 N^2-46 N-24\big ) P_{133} S_2}{3 (N-1)^2 N^4 (N+1)^4 (N+2)^4}\nonumber \\&\quad -\,\,\frac{32 \big (11 N^4+22 N^3-35 N^2-46 N-24\big ) \big (N^5+10 N^4+9 N^3+3 N^2+7 N+6\big ) S_3}{9 (N-1)^2 N^3 (N+1)^3 (N+2)^3} \nonumber \\&\quad -\,\,\frac{16 (-1)^N \big (N^4+2 N^3+7 N^2+22 N+20\big ) \big (11 N^4+22 N^3-35 N^2-46 N-24\big ) S_{-2}}{3 (N-1) N (N+1)^4 (N+2)^4}\nonumber \end{aligned}$$

$$\begin{aligned}&\quad -32 \frac{\big (N^2{+}N+1\big ) \big (N^2{+}N{+}2\big ) \zeta _2}{(N-1) N^2 (N{+}1)^2 (N+2)^2} S_{-2} +\,\,L_M^2 \Biggl [ -\frac{4 P_{69} S_1^2}{3 (N-1) N^2 (N+1)^2 (N+2)^2}-\,\,\frac{64 (-1)^N \big (N^3+4 N^2{+}7 N+5\big ) S_1}{(N+1)^3 (N+2)^3}\nonumber \\&\quad +\,\,\frac{8 P_{158} S_1}{9 (N-1)^2 N^3 (N+1)^3 (N+2)^3}-\,\,\frac{16 (-1)^N P_{138}}{3 (N-1) N^3 (N+1)^4 (N+2)^4}+\,\,\frac{16 P_{183}}{9 (N-1)^2 N^4 (N+1)^3 (N+2)^4} \nonumber \\&\quad -\,\,\frac{4 \big (N^2\!+\!N\!+\!2\big ) \big (11 N^4+22 N^3-83 N^2-94 N\!-\!72\big ) S_2}{3 (N-1) N^2 (N+1)^2 (N+2)^2} \!-\!\frac{8 \big (N^2\!+\!N\!+\!2\big ) \big (11 N^4\!+\!22 N^3\!-\!59 N^2\!-\!70 N\!-\!48\big ) S_{-2}}{3 (N\!-\!1) N^2 (N\!+\!1)^2 (N+2)^2}\nonumber \\&\quad +\,\,\tilde{\gamma }_{qg}^{0} \Bigl [4 S_1^3+12 S_2 S_1+16 S_{-2} S_1 +\,\,4 S_3+4 S_{-3}-8 S_{-2,1}\Bigr ]\Biggr ]+L_Q^2 \Biggl [ -\frac{4 P_{83} S_1^2}{3 (N-1) N^2 (N+1)^2 (N+2)^2}\nonumber \\&\quad +\,\,\frac{64 (-1)^N \big (N^3+4 N^2+7 N+5\big ) S_1}{(N+1)^3 (N+2)^3}-\,\,\frac{8 P_{160} S_1}{9 (N-1)^2 N^3 (N+1)^3 (N+2)^3} \nonumber \\&\quad +\frac{16 (-1)^N P_{138}}{3 (N{-}1) N^3 (N+1)^4 (N{+}2)^4}{-}\frac{16 P_{182}}{9 (N{-}1)^2 N^4 (N{+}1)^3 (N{+}2)^4}\nonumber \\&\quad +\frac{4 \big (N^2{+}N{+}2\big ) \big (11 N^4+22 N^3-83 N^2-94 N-72\big ) S_2}{3 (N-1) N^2 (N+1)^2 (N+2)^2}+\frac{8 \big (N^2{+}N{+}2\big ) \big (11 N^4+22 N^3-59 N^2-70 N-48\big ) S_{-2}}{3 (N-1) N^2 (N+1)^2 (N+2)^2} \nonumber \\ \end{aligned}$$

$$\begin{aligned}&\quad +\tilde{\gamma }_{qg}^{0} \Bigl [4 S_1^3-12 S_2 S_1{-}16 S_{-2} S_1-4 S_3-4 S_{-3} {+}8 S_{-2,1}\Bigr ]\Biggr ] +\frac{\big (5 N^5-131 N^3-58 N^2+232 N+96\big ) }{(N-1) N (N{+}1)^2 (N+2)^3} \Bigl [\frac{32}{3} (-1)^N S_1 S_{-2}\nonumber \\&\quad +\,\,\frac{16}{3} (-1)^N S_1 \zeta _2\Bigr ] +\,\,\frac{\big (N^2+N+2\big ) \big (11 N^4+22 N^3-35 N^2-46 N-24\big )}{(N-1) N^2 (N+1)^2 (N+2)^2}\times \Bigl [\frac{1}{3} S_2^2+\frac{16}{3} (-1)^N S_{-2} S_2 +\,\,6 S_4\nonumber \\&\quad +\frac{8}{3} (-1)^N S_{-4} -\frac{8}{3} S_{3,1} -\frac{16}{3} S_{-2,2} -\frac{16}{3} S_{-3,1}-\,\,\frac{8}{3} S_{2,1,1} +\frac{32}{3} S_{-2,1,1} +\,\,\Big (\frac{8}{3} (-1)^N S_2+\frac{8}{3} (-1)^N S_{-2}\Big ) \zeta _2\Bigr ]\nonumber \\&\quad +\,\,\frac{\big (N^2-N-4\big ) \big (11 N^4+22 N^3-35 N^2-46 N-24\big )}{(N-1) N (N+1)^3 (N+2)^3}\times \Bigl [ 4 (-1)^N \zeta _3 +\frac{16}{3} (-1)^N S_{-3} -\frac{32}{3} S_{-2,1} \Bigr ] \nonumber \\&\quad +\,\,\frac{P_{72} \Bigl [\frac{16}{3} (-1)^N S_{-2} S_1^2 +\frac{8}{3} (-1)^N \zeta _2 S_1^2\Bigr ]}{(N-1) N^2 (N+1)^2 (N+2)^2}\nonumber \\&\quad +\,\,\frac{\big (11 N^5+34 N^4-49 N^3-24 N^2-68 N-48\big ) \Bigl [\frac{16}{3} (-1)^N S_1 S_{-3} -\frac{32}{3} S_1 S_{-2,1}+4 (-1)^N S_1 \zeta _3\Bigr ]}{(N-1) N^2 (N+1) (N+2)^2} \nonumber \\&\quad +\,\,L_Q \Biggl [ \frac{8 \big (47 N^4-133 N^3+11 N^2-155 N-58\big ) S_1^3}{9 (N-1) N (N+1)^2 (N+2)}+\,\,\frac{8 P_{165} S_1^2}{9 (N-1)^2 N^3 (N+1)^3 (N+2)^3} \nonumber \\ \end{aligned}$$

$$\begin{aligned}&\quad -\,\,\frac{32 (N-2) (N+3) S_{-2} S_1^2}{N (N+1) (N+2)} -\frac{32 (-1)^N P_{139} S_1}{3 (N-1) N^3 (N+1)^4 (N+2)^4}+\,\,\frac{8 P_{199} S_1}{27 (N-1)^2 N^4 (N+1)^4 (N+2)^4} \nonumber \\&\quad -\,\,\frac{8 P_{79} S_2 S_1}{(N-1) N^2 (N+1)^2 (N+2)^2} +\frac{32 \big (N^2+N+6\big ) S_3 S_1}{N (N+1) (N+2)}-\,\,\frac{16 P_{84} S_{-2} S_1}{3 (N-1) N^2 (N+1)^2 (N+2)^2}\nonumber \\&\quad +\,\,\frac{32 \big (9 N^2+9 N+22\big ) S_{-3} S_1}{N (N+1) (N+2)}-\,\,\frac{64 \big (3 N^2+3 N+10\big ) S_{-2,1} S_1}{N (N+1) (N+2)}-\frac{384 \zeta _3 S_1}{N (N+1) (N+2)}\nonumber \\&\quad -\,\,8 \frac{P_{59} \zeta _3}{(N-1) N^2 (N+1)^2 (N+2)^2}+\,\,\frac{16 (-1)^N P_{198}}{9 (N-1)^2 N^4 (N+1)^5 (N+2)^5} +\,\,\frac{4 P_{211}}{27 (N-1)^2 N^5 (N+1)^5 (N+2)^5}\nonumber \\&\quad -\,\,\frac{8 P_{161} S_2}{9 (N-1)^2 N^3 (N+1)^3 (N+2)^3} -\,\,\frac{8 P_{91} S_3}{9 (N-1) N^2 (N+1)^2 (N+2)^2}-\,\,\frac{16 P_{162} S_{-2}}{9 (N-1)^2 N^3 (N+1)^3 (N+2)^3}\nonumber \\&\quad +\,\,\frac{\big (N^3+4 N^2+7 N+5\big ) \big (-64 (-1)^N S_1^2+64 (-1)^N S_2+192 (-1)^N S_{-2}\big )}{(N+1)^3 (N+2)^3} +\,\,\frac{16 P_{75} S_{-3}}{3 (N-1) N^2 (N+1)^2 (N+2)^2}\nonumber \\&\quad +\,\,\frac{32 P_{54} S_{-2,1}}{3 (N-1) N^2 (N+1)^2 (N+2)^2} +\,\,\tilde{\gamma }_{qg}^{0} \Bigl [-2 S_1^4+16 S_2 S_1^2-2 S_2^2-12 S_{-2}^2-16 S_{-2} S_2-4 S_4-\,\,44 S_{-4} -\frac{88}{3} S_{2,1} \nonumber \\&\quad -16 S_{3,1}+56 S_{-2,2}+\,\,64 S_{-3,1}-96 S_{-2,1,1}\Bigr ]\Biggr ] +\tilde{\gamma }_{qg}^{0} \Bigl [ -\frac{1}{3} S_1^5-10 S_2 S_1^3 -\,\,16 (-1)^N S_{-3} S_1^2+\Bigl [32 S_{-2,1} -\frac{80 S_3}{3}\Bigr ] S_1^2 \nonumber \end{aligned}$$

$$\begin{aligned}&\quad -\,\,\frac{4}{3} \big (-7+9 (-1)^N\big ) \zeta _3 S_1^2 -8 (-1)^N S_{-4} S_1 +\,\,\Bigl [-S_2^2-18 S_4+8 S_{3,1}+16 S_{-2,2}+16 S_{-3,1}+8 S_{2,1,1}-\,\,32 S_{-2,1,1}\Bigr ] S_1 \nonumber \\&\quad +S_{-2} \Bigl [-16 (-1)^N S_1^3 -16 (-1)^N S_2 S_1\Bigr ] +\,\,\Bigl [-4 \big (-1+2 (-1)^N\big ) S_1^3 -8 (-1)^N S_2 S_1-\,\,4 \big (1+2 (-1)^N\big ) S_{-2} S_1 \nonumber \\&\quad +\frac{11 S_2}{3}\!-\!2 S_3 \!-\!2 S_{-3}\!+\!4 S_{-2,1}\Bigr ] \zeta _2\Bigr ] \!+\!\,\,L_M \Biggl [ \frac{128 (-1)^N P_{113} S_1}{3 (N\!-\!1) N^2 (N\!+\!1)^4 (N\!+\!2)^4}\!-\!\,\,\frac{8 \big (11 N^4+26 N^3-139 N^2-218 N\!+\!8\big ) S_1^3}{9 N (N\!+\!1)^2 (N\!+\!2)^2}\nonumber \\&\quad +\,\,\frac{4 P_{163} S_1^2}{9 (N-1)^2 N^3 (N+1)^3 (N+2)^3} -\,\,\frac{8 P_{196} S_1}{27 (N-1)^2 N^4 (N+1)^4 (N+2)^4}-\,\,\frac{8 P_{66} S_2 S_1}{3 (N-1) N^2 (N+1)^2 (N+2)^2}\nonumber \\&\quad -\,\,\frac{32 \big (2 N^5-23 N^4-32 N^3+13 N^2+4 N-12\big ) S_{-2} S_1}{(N-1) N^2 (N+1)^2 (N+2)^2}+\,\,2 \frac{P_{93}\zeta _3}{(N-1) N^2 (N+1)^2 (N+2)^2} \nonumber \\&\quad -\,\,\frac{16 (-1)^N P_{179}}{9 (N-1) N^3 (N+1)^5 (N+2)^5}-\,\,\frac{4P_{210}}{27 (N-1)^2 N^5 (N+1)^5 (N+2)^5} +\,\,\frac{4 P_{164} S_2}{9 (N-1)^2 N^3 (N+1)^3 (N+2)^3} \nonumber \\&\quad +\,\,\frac{\big (N^3+4 N^2+7 N+5\big ) \big (-64 (-1)^N S_1^2-64 (-1)^N S_2\big )}{(N+1)^3 (N+2)^3} -\,\,\frac{8 P_{87} S_3}{9 (N-1) N^2 (N+1)^2 (N+2)^2}\nonumber \\&\quad -\,\,\frac{16 (-1)^N P_{71} S_{-2}}{3 (N-1) N (N+1)^3 (N+2)^3} +\,\,\frac{16 P_{144} S_{-2}}{9 (N-1) N^3 (N+1)^3 (N+2)^3}-\,\,\frac{16 P_{68} S_{-3}}{3 (N-1) N^2 (N+1)^2 (N+2)^2}\nonumber \\&\quad -\,\,\frac{16 \big (N^2+N+2\big ) \big (11 N^4+22 N^3+13 N^2+2 N+24\big ) S_{2,1}}{3 (N-1) N^2 (N+1)^2 (N+2)^2}+\,\,\frac{\big (N^2+N+2\big ) \big (11 N^4+22 N^3-35 N^2-46 N-24\big ) }{(N-1) N^2 (N+1)^2 (N+2)^2}\nonumber \\ \end{aligned}$$

$$\begin{aligned}&\quad \Bigl [ -\frac{8}{3} (-1)^N S_{-3}-\,\,2 (-1)^N \zeta _3\Bigr ] +\,\,\frac{\big (11 N^5+34 N^4-49 N^3-24 N^2-68 N-48\big ) \Bigl [ -\frac{16}{3} (-1)^N S_1 S_{-2} \Bigr ]}{(N-1) N^2 (N+1) (N+2)^2}\nonumber \\&\quad +\,\,\tilde{\gamma }_{qg}^{0} \Bigl [2 S_1^4\!+\!32 S_2 S_1^2 \!+\!8 \big (8\!+\!(-1)^N\big ) S_{-3} S_1 \!+\!\,\,\Bigl [40 S_3\!-\!16 S_{2,1} -80 S_{-2,1}\Bigr ] S_1 +\,\,6 \big (-5+(-1)^N\big ) \zeta _3 S_1 +2 S_2^2 +12 S_{-2}^2 \nonumber \\&\quad +\,\,S_{-2} \Bigl [8 \big (3+2 (-1)^N\big ) S_1^2+16 S_2\Bigr ] +4 S_4 \!+\!44 S_{-4} \!+\!16 S_{3,1}-\,\,56 S_{-2,2} -64 S_{-3,1} \!+\!96 S_{-2,1,1}\Bigr ]\nonumber \\&\quad +\,\,\frac{16 P_{67} S_{-2,1}}{3 (N-1) N^2 (N+1)^2 (N+2)^2} \Biggr ]\Biggr ]+\,\,{C_F} {T_F^2} \Biggl [ \Biggl [ \frac{16 \big (N^2+N+1\big ) \big (N^2+N+2\big ) \big (3 N^4+6 N^3-N^2-4 N+12\big )}{9 (N-1) N^3 (N+1)^3 (N+2)^2} \nonumber \\&\quad +\,\,\frac{16}{9} \tilde{\gamma }_{qg}^{0} S_1 \Biggr ] L_Q^3 +\,\,\Biggl [-\frac{16 L_M \big (N^2+N+2\big )^3}{(N-1) N^3 (N+1)^3 (N+2)^2}-\,\,\frac{4 P_{174}}{9 (N-1) N^4 (N+1)^4 (N+2)^3} \nonumber \\&\quad +\,\,\frac{16 P_{119} S_1}{9 (N-1) N^3 (N+1)^3 (N+2)^2} +\tilde{\gamma }_{qg}^{0} \Bigl [\frac{20 S_2}{3}-4 S_1^2\Bigr ]\Biggr ] L_Q^2 +\,\,\Biggl [ -\frac{16 P_{123} S_1^2}{9 (N-1) N^3 (N+1)^3 (N+2)^2}\nonumber \\&\quad -\,\,\frac{8 P_{171} S_1}{9 (N-1) N^4 (N+1)^4 (N+2)^3}+\,\,\frac{64 (-1)^N P_{201}}{45 (N-2) (N-1)^2 N^3 (N+1)^4 (N+2)^4 (N+3)^3}\nonumber \\&\quad +\,\,\frac{4 P_{217}}{45 (N-1)^2 N^5 (N+1)^5 (N+2)^4 (N+3)^3}+\,\,L_M^2 \Biggl [ -\frac{16 \big (N^2+N+2\big ) P_{51}}{3 (N-1) N^3 (N+1)^3 (N+2)^2} -\,\,\frac{16}{3} \tilde{\gamma }_{qg}^{0} S_1\Biggr ]\nonumber \\ \end{aligned}$$

$$\begin{aligned}&\quad +L_M \Biggl [ \frac{8 P_{173}}{9 (N-1) N^4 (N+1)^4 (N+2)^3} -\,\,\frac{32 P_{115} S_1}{9 (N-1) N^3 (N+1)^3 (N+2)^2} +\,\,\tilde{\gamma }_{qg}^{0} \Bigl [ -\frac{8}{3} S_1^2 -\frac{8 S_2}{3}\Bigr ]\Biggr ]\nonumber \\&\quad +\frac{16 P_{125} S_2}{9 (N-1) N^3 (N+1)^3 (N+2)^2} -\,\,\frac{256 \big (N^2+N+1\big ) S_3}{3 N (N+1) (N+2)} +\,\,\frac{64 P_{105} S_{-2}}{3 (N-2) (N-1) N^2 (N+1)^2 (N+2)^2 (N+3)}\nonumber \\&\quad +\,\,\tilde{\gamma }_{qg}^{0} \Bigl [\frac{8}{3} S_1^3-8 S_2 S_1-\frac{32}{3} S_{2,1}\Bigr ]+\,\,\frac{\frac{512}{3} S_1 S_{-2} +\frac{256}{3} S_{-3} -\frac{512}{3} S_{-2,1}}{N (N+1) (N+2)} +\frac{64 (N-1) \zeta _3}{N (N+1)}\Biggr ] L_Q\nonumber \\&\quad -\,\,\frac{16 \big (N^4-5 N^3-32 N^2-18 N-4\big ) S_1^2}{3 N^2 (N+1)^2 (N+2)} -\,\,\frac{16}{9} \big (N^2+N+2\big ) \frac{\zeta _3}{(N-1) N^3 (N+1)^3 (N+2)^2} P_{55}\nonumber \\&\quad +\,\,\frac{2}{9} \frac{P_{176} \zeta _2}{(N-1) N^4 (N+1)^4 (N+2)^3} -\,\,\frac{2 P_{195}}{3 (N-1) N^5 (N+1)^6 (N+2)^2}+\,\,\frac{4 P_{186} S_1}{3 (N-1) N^5 (N+1)^5 (N+2)^2}\nonumber \\&\quad -\,\,\frac{80}{9} \frac{\big (N^3+4 N^2+11 N+6\big )\zeta _2}{N^2 (N+1) (N+2)} S_1+\,\,L_M^3 \Biggl [ \frac{16 \big (N^2+N+2\big ) P_{55}}{9 (N-1) N^3 (N+1)^3 (N+2)^2} +\,\,\frac{32}{9} \tilde{\gamma }_{qg}^{0} S_1\Biggr ] \nonumber \end{aligned}$$

$$\begin{aligned}&\quad +L_M^2 \Biggl [ -\frac{4 P_{169}}{9 (N-1) N^4 (N+1)^4 (N+2)^3} +\,\,\frac{16 P_{106} S_1}{9 (N-1) N^3 (N+1)^3 (N+2)^2} +\,\,\tilde{\gamma }_{qg}^{0} \Bigl [\frac{20}{3} S_1^2-4 S_2\Bigr ]\Biggr ]\nonumber \\&\quad -\frac{16 P_{52} S_2}{3 N^3 (N+1)^3 (N+2)}+\,\,\frac{(3 N+2) \Bigl [ -\frac{32}{9} S_1^3 -\frac{32}{3} S_2 S_1\Bigr ]}{N^2 (N+2)}-\,\,\frac{32 \big (3 N^4+48 N^3+43 N^2-22 N-8\big ) S_3}{9 N^2 (N+1)^2 (N+2)}\nonumber \\&\quad +\,\,\frac{128 \big (N^2-3 N-2\big ) S_{2,1}}{3 N^2 (N+1) (N+2)} +\,\,L_M \Biggl [ \frac{16 P_{116} S_1^2}{9 (N-1) N^3 (N+1)^3 (N+2)^2}+\,\,\frac{8 P_{172} S_1}{9 (N-1) N^4 (N+1)^4 (N+2)^3}\nonumber \\&\quad +\,\,\frac{64 (-1)^N P_{202}}{45 (N-2) (N-1)^2 N^3 (N+1)^4 (N+2)^4 (N+3)^3} +\,\,\frac{4 P_{216}}{45 (N-1)^2 N^5 (N+1)^5 (N+2)^4 (N+3)^3} \nonumber \\&\quad -\,\,\frac{16 P_{121} S_2}{9 (N-1) N^3 (N+1)^3 (N+2)^2} +\tilde{\gamma }_{qg}^{0} \Bigl [ \frac{8}{3} S_1^3+\frac{8}{3} S_2 S_1\Bigr ]+\,\,\frac{128 \big (N^2+N+4\big ) S_3}{3 N (N+1) (N+2)}\nonumber \\&\quad +\,\,\frac{64 P_{104} S_{-2}}{3 (N-2) (N-1) N^2 (N+1)^2 (N+2)^2 (N+3)} +\,\,\frac{\frac{512}{3} S_1 S_{-2}+\frac{256}{3} S_{-3} -\frac{512}{3} S_{-2,1}}{N (N+1) (N+2)}\nonumber \\&\quad +\,\,\frac{64 (N-1) \zeta _3}{N (N+1)}\Biggr ] +\tilde{\gamma }_{qg}^{0} \Bigl [ -\frac{2}{9} S_1^4 -\frac{4}{3} S_2 S_1^2+\Bigl [ -\frac{16}{9} S_3 -\frac{32}{3} S_{2,1}\Bigr ] S_1\nonumber -\,\,\frac{32}{9} \zeta _3 S_1 -\frac{2}{3} S_2^2+4 S_4 -\,\,\frac{32}{3} S_{3,1}+\frac{64}{3} S_{2,1,1}\nonumber \\ \end{aligned}$$

$$\begin{aligned}&\quad +\Bigl [2 S_2 -\frac{10}{3} S_1^2\Bigr ] \zeta _2\Bigr ] \Biggr ] +\,\,{C_F} {N_F} {T_F^2} \Biggl [\Biggl [ \frac{16}{9} \tilde{\gamma }_{qg}^{0} S_1 +\,\,\frac{16 \big (N^2+N+1\big ) \big (N^2+N+2\big ) \big (3 N^4+6 N^3-N^2-4 N+12\big )}{9 (N-1) N^3 (N+1)^3 (N+2)^2} \Biggr ] L_Q^3\nonumber \\&\quad +\,\,\Biggl [ -\frac{4 P_{174}}{9 (N-1) N^4 (N+1)^4 (N+2)^3}+\,\,\frac{16 P_{119} S_1}{9 (N-1) N^3 (N+1)^3 (N+2)^2}+\,\,L_M \Biggl [ -\frac{8 \big (N^2+N+2\big ) \big (3 N^2+3 N+2\big )}{3 N^2 (N+1)^2 (N+2)} \nonumber \\&\quad -\frac{8}{3} \tilde{\gamma }_{qg}^{0} S_1\Biggr ]+\,\,\tilde{\gamma }_{qg}^{0} \Bigl [\frac{20 S_2}{3}-4 S_1^2\Bigr ]\Biggr ] L_Q^2 +\,\,\Biggl [ -\frac{16 P_{123} S_1^2}{9 (N-1) N^3 (N+1)^3 (N+2)^2} -\,\,\frac{8 P_{171} S_1}{9 (N-1) N^4 (N+1)^4 (N+2)^3}\nonumber \\&\quad +\,\,\frac{64 (-1)^N P_{201}}{45 (N-2) (N-1)^2 N^3 (N+1)^4 (N+2)^4 (N+3)^3} +\,\,\frac{8 P_{218}}{45 (N-1)^2 N^5 (N+1)^5 (N+2)^4 (N+3)^3}\nonumber \\&\quad +\,\,L_M \Biggl [\frac{8 \big (N^2+N+2\big ) \big (57 N^4+72 N^3+29 N^2-22 N-24\big )}{9 N^3 (N+1)^3 (N+2)}+\,\,\tilde{\gamma }_{qg}^{0} \Bigl [\frac{8}{3} S_1^2-8 S_2\Bigr ]\nonumber \\&\quad -\,\,\frac{16 \big (N^2+N+2\big ) \big (29 N^2+29 N-6\big ) S_1}{9 N^2 (N+1)^2 (N+2)} \Biggr ]-\,\,\frac{256 \big (N^2+N+1\big ) S_3}{3 N (N+1) (N+2)}+\,\,\frac{16 P_{125} S_2}{9 (N-1) N^3 (N+1)^3 (N+2)^2} \nonumber \\&\quad +\,\,\frac{64 P_{105} S_{-2}}{3 (N-2) (N-1) N^2 (N+1)^2 (N+2)^2 (N+3)} +\,\,\tilde{\gamma }_{qg}^{0} \Bigl [\frac{8}{3} S_1^3-8 S_2 S_1 -\frac{32}{3} S_{2,1}\Bigr ] \nonumber \\ \end{aligned}$$

$$\begin{aligned}&\quad +\,\,\frac{\frac{512}{3} S_1 S_{-2}+\frac{256}{3} S_{-3} -\frac{512}{3} S_{-2,1}}{N (N+1) (N+2)} +\frac{64 (N-1) \zeta _3}{N (N+1)}\Biggr ]L_Q-\,\,\frac{2}{9} \frac{P_{177} \zeta _2}{(N-1) N^4 (N+1)^4 (N+2)^3}\nonumber \\&\quad -\,\,\frac{8 \big (N^4-5 N^3-32 N^2-18 N-4\big ) S_1^2}{3 N^2 (N+1)^2 (N+2)}-\,\,\frac{8}{9} \frac{\big (N^2+N+2\big ) \zeta _3}{(N-1) N^3 (N+1)^3 (N+2)^2} P_{50}\nonumber \\&\quad -\,\,\frac{4 P_{213}}{3 (N-1) N^6 (N+1)^6 (N+2)^5}+\,\,\frac{16 \big (2 N^5-2 N^4-11 N^3-19 N^2-44 N-12\big ) S_1}{3 N^2 (N+1)^3 (N+2)} \nonumber \\&\quad -\,\,\frac{16}{9} \frac{\big (5 N^3+11 N^2+28 N+12\big )\zeta _2}{N^2 (N+1) (N+2)} S_1+\,\,L_M^3 \Biggl [ \frac{8 \big (N^2+N+2\big ) P_{50}}{9 (N-1) N^3 (N+1)^3 (N+2)^2}+\,\,\frac{8}{9} \tilde{\gamma }_{qg}^{0} S_1\Biggr ] \nonumber \\&\quad -\,\,\frac{8 P_{167} S_2}{3 (N-1) N^4 (N+1)^4 (N+2)^3}+\,\,\frac{(3 N+2) \Bigl [ -\frac{16}{9} S_1^3 -\frac{16}{3} S_2 S_1\Bigr ]}{N^2 (N+2)}+\,\,L_M^2 \Biggl [ -\frac{4 P_{170}}{9 (N-1) N^4 (N+1)^4 (N+2)^3}\nonumber \\&\quad +\,\,\frac{32 \big (5 N^3+8 N^2+19 N+6\big ) S_1}{9 N^2 (N+1) (N+2)} +\,\,\tilde{\gamma }_{qg}^{0} \Bigl [\frac{4}{3} S_1^2+\frac{4 S_2}{3}\Bigr ]\Biggr ] -\,\,\frac{16 P_{111} S_3}{9 (N-1) N^3 (N+1)^3 (N+2)^2}\nonumber \end{aligned}$$

$$\begin{aligned}&\quad +\frac{64 \big (N^2-3 N-2\big ) S_{2,1}}{3 N^2 (N+1) (N+2)}+\,\,L_M \Biggl [ \frac{8 \big (49 N^4+122 N^3+213 N^2+164 N+12\big ) S_1^2}{9 N^2 (N+1)^2 (N+2)}\nonumber \\&\quad -\,\,\frac{8P_{200}}{9 (N-1) N^5 (N+1)^5 (N+2)^4}+\,\,\frac{8 P_{88} S_1}{9 N^3 (N+1)^3 (N+2)} -\frac{8 P_{126} S_2}{9 (N-1) N^3 (N+1)^3 (N+2)^2}\nonumber \\&\quad +\,\,\tilde{\gamma }_{qg}^{0} \Bigl [\frac{16}{3} S_1 S_2-16 S_3+\frac{16}{3} S_{2,1}\Bigr ]\Biggr ] +\,\,\tilde{\gamma }_{qg}^{0} \Bigl [ -\frac{1}{9} S_1^4 -\frac{2}{3} S_2 S_1^2 -\frac{4}{3} \zeta _2 S_1^2+\Bigl [ -\frac{8}{9} S_3 -\frac{16}{3} S_{2,1}\Bigr ] S_1\nonumber \\&\quad -\,\,\frac{8}{9} \zeta _3 S_1 -\frac{1}{3} S_2^2+2 S_4 -\frac{16}{3} S_{3,1} +\frac{32}{3} S_{2,1,1}\Bigr ]\Biggr ]+\,\,{C_A} {C_F} {T_F} \Biggl [ \frac{2 P_{76} S_1^4}{9 (N-1) N^2 (N+1)^2 (N+2)^2}\nonumber \\&\quad +\,\,\frac{4 P_{122} S_1^3}{9 (N-1) N^3 (N+1)^3 (N+2)^2}-\,\,\frac{4 P_{187} S_1^2}{3 (N-1) N^4 (N+1)^4 (N+2)^4}\nonumber \\&\quad -\,\,\frac{8}{3} \frac{\big (19 N^5-11 N^4-8 N^3-49 N^2-17 N+18\big ) \zeta _2}{(N-1) N^2 (N+1)^2 (N+2)} S_1^2+\,\,\frac{4 P_{85} S_2 S_1^2}{3 (N-1) N^2 (N+1)^2 (N+2)^2}\nonumber \\&\quad -\,\,\frac{2}{9} \frac{P_{97} S_1\zeta _3}{(N-1) N^2 (N+1)^2 (N+2)^2}+\,\,\frac{4}{9} \frac{P_{131} S_1\zeta _2}{(N-1) N^3 (N+1)^3 (N+2)^2}\nonumber \\ \end{aligned}$$

$$\begin{aligned}&\quad +\,\,\frac{4 P_{205} S_1}{3 (N-1) N^5 (N+1)^5 (N+2)^5}+\,\,\frac{4 P_{142} S_2 S_1}{3 (N-1) N^3 (N+1)^3 (N+2)^3}+\,\,\frac{8 P_{95} S_3 S_1}{9 (N-1) N^2 (N+1)^2 (N+2)^2}\nonumber \\&\quad -\,\,\frac{16 \big (11 N^5+45 N^4-3 N^3-145 N^2-176 N-20\big ) S_{2,1} S_1}{3 (N-1) N (N+1)^2 (N+2)^2}-\,\,\frac{2 P_{43} S_2^2}{3 (N-1) N^2 (N+1)^2 (N+2)^2}\nonumber \\&\quad -\,\,\frac{256 (-1)^N \big (N^3+4 N^2+7 N+5\big )}{(N+1)^3 (N+2)^3}+\,\,8\frac{(-1)^N \zeta _2}{N^2 (N+1)^4 (N+2)^3} P_{57}-\,\,\frac{2}{9} \frac{ P_{130} \zeta _3}{(N-1) N^3 (N+1)^3 (N+2)^2}\nonumber \\&\quad -\,\,\frac{1}{18} \frac{P_{150}\zeta _2}{(N-1) N^3 (N+1)^4 (N+2)^2}+\,\,\frac{P_{215}}{3 (N-1) N^6 (N+1)^6 (N+2)^5}+\,\,L_M^3 \Biggl [ -\frac{16}{3} \tilde{\gamma }_{qg}^{0} S_1^2\nonumber \\&\quad -\,\,\frac{8 \big (N^2+N+2\big ) \big (N^2+N+6\big ) \big (7 N^2+7 N+4\big ) S_1}{9 (N-1) N^2 (N+1)^2 (N+2)^2}\nonumber \\&\quad -\,\,\frac{2 \big (N^2+N+2\big ) \big (3 N^2+3 N+2\big ) \big (11 N^4+22 N^3-59 N^2-70 N-48\big )}{9 (N-1) N^3 (N+1)^3 (N+2)^2}\Biggr ]\nonumber \\&\quad +\,\,L_Q^3 \Biggl [ -\frac{8}{3} \tilde{\gamma }_{qg}^{0} S_1^2+\,\,\frac{8 \big (N^2+N+2\big ) \big (13 N^4+26 N^3-43 N^2-56 N-12\big ) S_1}{9 (N-1) N^2 (N+1)^2 (N+2)^2}\nonumber \\&\quad -\,\,\frac{4 \big (N^2+N+2\big ) \big (3 N^2+3 N+2\big ) \big (11 N^4+22 N^3-23 N^2-34 N-12\big )}{9 (N-1) N^3 (N+1)^3 (N+2)^2}\Biggr ]-\,\,4 \frac{\zeta _2 P_{49} S_2}{(N-1) N^2 (N+1)^2 (N+2)^2}\nonumber \\ \end{aligned}$$

$$\begin{aligned}&\quad -\,\,\frac{4 P_{185} S_2}{3 (N-1) N^4 (N+1)^4 (N+2)^4}+\,\,\frac{4 P_{127} S_3}{9 (N-1) N^3 (N+1)^3 (N+2)^2}\nonumber \\&\quad +\,\,\frac{4 \big (N^2+N+2\big ) \big (19 N^4+38 N^3-22 N^2-41 N-30\big ) S_4}{(N-1) N^2 (N+1)^2 (N+2)^2}+\,\,\frac{16 (-1)^N P_{58} S_{-2}}{N^2 (N+1)^4 (N+2)^3} \nonumber \\&\quad -\,\,\frac{16 \big (N^2\!-\!3 N\!-\!2\big ) \big (11 N^4\!+\!22 N^3\!-\!35 N^2\!-\!46 N\!-\!24\big ) S_{2,1}}{3 (N\!-\!1) N^3 (N\!+\!1)^2 (N\!+\!2)^2}-\,\,\frac{8 \big (N^2\!+\!N\!+\!2\big ) \big (31 N^4\!+\!62 N^3\!-\!73 N^2\!-\!104 N\!-\!60\big ) S_{3,1}}{3 (N\!-\!1) N^2 (N\!+\!1)^2 (N\!+\!2)^2}\nonumber \\&\quad +\,\,L_Q^2 \Biggl [ -\frac{8 \big (10 N^5+6 N^4+5 N^3-38 N^2-17 N+2\big ) S_1^2}{(N-1) N^2 (N+1)^2 (N+2)}+\,\,\frac{64 (-1)^N \big (N^3+4 N^2+7 N+5\big ) S_1}{(N+1)^3 (N+2)^3}\nonumber \\&\quad -\,\,\frac{4 P_{146} S_1}{9 (N-1) N^3 (N+1)^3 (N+2)^3}-\,\,\frac{16 (-1)^N \big (3 N^4+11 N^3+19 N^2+15 N+2\big )}{N (N+1)^3 (N+2)^3}+\,\,\frac{P_{180}}{9 (N-1) N^4 (N+1)^4 (N+2)^3}\nonumber \\&\quad +\,\,L_M \Biggl [ \frac{22 \big (N^2+N+2\big ) \big (3 N^2+3 N+2\big )}{3 N^2 (N+1)^2 (N+2)}+\,\,\frac{22}{3} \tilde{\gamma }_{qg}^{0} S_1\Biggr ] +\frac{8 \big (N^2+N+2\big ) \big (23 N^4+46 N^3-50 N^2-73 N-18\big ) S_2}{3 (N-1) N^2 (N+1)^2 (N+2)^2}\nonumber \end{aligned}$$

$$\begin{aligned}&\quad +\,\,\tilde{\gamma }_{qg}^{0} \Bigl [4 S_1^3-12 S_2 S_1 +4 S_3+6 S_{-2} +4 S_{-3}-8 S_{-2,1}\Bigr ]\Biggr ]+\,\,L_M^2 \Biggl [ \frac{8 P_{63} S_1^2}{3 (N-1) N^2 (N+1)^2 (N+2)^2}\nonumber \\&\quad +\,\,\frac{64 (-1)^N \big (N^3+4 N^2+7 N+5\big ) S_1}{(N+1)^3 (N+2)^3}-\,\,\frac{8 P_{145} S_1}{9 (N-1) N^3 (N+1)^3 (N+2)^3}\nonumber \\&\quad -\,\,\frac{16 (-1)^N \big (3 N^4+11 N^3+19 N^2+15 N+2\big )}{N (N+1)^3 (N+2)^3}+\,\,\frac{P_{166}}{9 (N-1) N^4 (N+1)^3 (N+2)^3}\nonumber \\&\quad +\,\,\frac{8 \big (N^2+N+2\big ) \big (N^4+2 N^3+20 N^2+19 N+30\big ) S_2}{3 (N-1) N^2 (N+1)^2 (N+2)^2}+\,\,\tilde{\gamma }_{qg}^{0} \Bigl [-12 S_1^3+4 S_2 S_1+4 S_3+6 S_{-2}\nonumber \\&\quad +\,\,4 S_{-3}-8 S_{-2,1}\Bigr ]\Biggr ]+\,\,\frac{8 \big (N^2+N+2\big ) \big (35 N^4+70 N^3-137 N^2-172 N-84\big ) S_{2,1,1}}{3 (N-1) N^2 (N+1)^2 (N+2)^2}\nonumber \\&\quad -\,\,\frac{8 \big (N^2+N+2\big ) S_{-2} \zeta _2}{N^2 (N+1)^2 (N+2)} -48 \tilde{\gamma }_{qg}^{0} \log (2) \zeta _2+\,\,\frac{P_{110} \Bigl [16 (-1)^N S_1 S_{-2} +8 (-1)^N S_1 \zeta _2\Bigr ]}{N^3 (N+1)^3 (N+2)^3}\nonumber \\&\quad +\,\,\frac{\big (N^4+3 N^3+8 N^2+12 N+4\big ) \Bigl [96 (-1)^N S_{-2} S_1^2+48 (-1)^N \zeta _2 S_1^2\Bigr ]}{N (N+1)^2 (N+2)^2}\nonumber \\ \end{aligned}$$

$$\begin{aligned}&\quad +\,\,\frac{ \big (3 N^5+10 N^4+25 N^3+38 N^2+20 N+8\big ) \Bigl [16 (-1)^N S_2 S_{-2}+8 (-1)^N S_2 \zeta _2\Bigr ]}{N^2 (N+1)^2 (N+2)^2}+\,\,\frac{\big (N^2+N+2\big ) \big (3 N^2+3 N+2\big )}{N^2 (N+1)^2 (N+2)}\nonumber \\&\quad \times \Bigl [ 8 (-1)^N S_{-2} \zeta _2 +8 (-1)^N S_{-4}-\,\,16 S_{-2,2} -16 S_{-3,1}+32 S_{-2,1,1}\Bigr ]+\,\,\frac{P_{98} \Bigl [8 (-1)^N S_{-3}-16 S_{-2,1}+6 (-1)^N \zeta _3\Bigr ]}{N^3 (N+1)^3 (N+2)^2}\nonumber \\&\quad +\,\,\frac{\big (9 N^5+28 N^4+73 N^3+110 N^2+44 N+8\big ) \Bigl [8 (-1)^N S_1 S_{-3}-16 S_1 S_{-2,1}+6 (-1)^N S_1 \zeta _3\Bigr ]}{N^2 (N+1)^2 (N+2)^2}\nonumber \\&\quad +\,\,\tilde{\gamma }_{qg}^{0} \Bigl [\frac{20}{3} S_2 S_1^3 +\Bigl [24 S_3+16 S_{2,1}-24 S_{-2,1}\Bigr ]S_1^2+\,\,8 (-1)^N S_{-4} S_1 +\Bigl [8 S_2^2+12 S_4+8 S_{3,1}-16 S_{-2,2}\nonumber \\&\quad -\,\,16 S_{-3,1}-40 S_{2,1,1}+32 S_{-2,1,1}\Bigr ] S_1+S_{-3}\Bigl [12 (-1)^N S_1^2+\,\,4 (-1)^N S_2\Bigr ] +S_2 \Bigl [\frac{16 S_3}{3}-8 S_{-2,1}\Bigr ]\nonumber \\&\quad +\,\,S_{-2} \Bigl [8 (-1)^N S_1^3+24 (-1)^N S_2 S_1+32\Bigr ]+\,\,\Bigl [4 \big (1+(-1)^N\big ) S_1^3 +4 \big (-1+2 (-1)^N\big ) S_{-2} S_1-2 S_3\nonumber \\&\quad -\,\,2 S_{-3}+4 S_{-2,1}\Bigr ] \zeta _2 +\Bigl [\frac{1}{3} \big (-11+27 (-1)^N\big ) S_1^2 \Bigr ] \zeta _3\Bigr ]+\,\,L_Q \Biggl [ \frac{8 \big (29 N^5+81 N^4+117 N^3-49 N^2-362 N-104\big ) S_1^3}{3 (N-1) N (N+1)^2 (N+2)^2}\nonumber \\ \end{aligned}$$

$$\begin{aligned}&\quad +\,\,\frac{4 P_{148} S_1^2}{9 (N-1) N^3 (N+1)^3 (N+2)^3} -\,\,\frac{32 (-1)^N P_{197} S_1}{15 (N-2) (N-1)^2 N^2 (N+1)^4 (N+2)^4 (N+3)^3} \nonumber \\&\quad +\,\,\frac{2 P_{212} S_1}{45 (N-1)^2 N^4 (N+1)^4 (N+2)^4 (N+3)^3} -\,\,\frac{8 \big (17 N^5+33 N^4-43 N^3-153 N^2-254 N-80\big ) S_2 S_1}{(N-1) N (N+1)^2 (N+2)^2} \nonumber \\&\quad +\,\,\frac{32 (N-2) (N+3) S_3 S_1}{N (N+1) (N+2)}-\,\,\frac{16 P_{100} S_{-2} S_1}{3 (N-2) (N-1) N (N+1)^2 (N+2)^2 (N+3)}+\,\,\frac{512 S_{-2,1} S_1}{N (N+1) (N+2)} \nonumber \\&\quad -\,\,\frac{24 \big (11 N^2+11 N-10\big ) \zeta _3 S_1}{N (N\!+\!1) (N+2)}\!+\!\,\,2 \frac{\zeta _3 P_{96}}{(N\!-\!1) N^2 (N\!+\!1)^2 (N\!+\!2)^2}\! +\!\,\,\frac{16 (-1)^N P_{214}}{45 (N\!-\!2) (N\!-\!1)^3 N^3 (N+1)^5 (N\!+\!2)^5 (N\!+\!3)^3} \nonumber \\&\quad -\,\,\frac{2 P_{221}}{45 (N-1)^3 N^5 (N+1)^5 (N+2)^5 (N+3)^3}+\,\,L_M^2 \Biggl [ 8 \tilde{\gamma }_{qg}^{0} S_1^2 +\frac{8 \big (N^2+N+2\big ) \big (3 N^4+6 N^3+7 N^2+4 N+4\big ) S_1}{(N-1) N^2 (N+1)^2 (N+2)^2} \nonumber \\&\quad -\,\,\frac{16 \big (N^2+N+1\big ) \big (N^2+N+2\big ) \big (3 N^2+3 N+2\big )}{(N-1) N^3 (N+1)^3 (N+2)^2}\Biggr ] +\,\,\frac{\big (N^2+N+10\big ) \Bigl [-64 S_{-2} S_1^2-32 S_{-3} S_1\Bigr ]}{N (N+1) (N+2)} \nonumber \\&\quad -\,\,\frac{4 P_{149} S_2}{9 (N-1) N^3 (N+1)^3 (N+2)^3} +\,\,\frac{\big (N^3+4 N^2+7 N+5\big ) \big (128 (-1)^N S_2-128 (-1)^N S_1^2\big )}{(N+1)^3 (N+2)^3}\nonumber \end{aligned}$$

$$\begin{aligned}&\quad +\,\,\frac{32 P_{77} S_3}{3 (N-1) N^2 (N+1)^2 (N+2)^2} +\,\,\frac{16 (-1)^N \big (3 N^5-6 N^4-61 N^3-124 N^2-96 N-16\big ) S_{-2}}{N (N+1)^3 (N+2)^3} \nonumber \\&\quad -\,\,\frac{16 P_{61} S_{-3}}{3 (N-1) N^2 (N+1)^2 (N+2)^2} +\,\,\frac{32 P_{168} S_{-2}}{3 (N-2) (N-1) N^3 (N+1)^3 (N+2)^3 (N+3)} \nonumber \\&\quad -\,\,\frac{16 \big (N^2+N+2\big ) \big (31 N^2+31 N+6\big ) S_{2,1}}{3 N^2 (N+1)^2 (N+2)}-\,\,\frac{16 P_{62} S_{-2,1}}{3 (N-1) N^2 (N+1)^2 (N+2)^2}\nonumber \\&\quad +\,\,L_M \Biggl [\frac{16 \big (10 N^5+40 N^4+121 N^3+161 N^2+52 N+12\big ) S_1^2}{3 N^2 (N+1)^2 (N+2)^2} +\,\,\frac{4 P_{147} S_1}{9 (N-1) N^3 (N+1)^3 (N+2)^3}\nonumber \\&\quad -\frac{128 (-1)^N \big (N^3{+}4 N^2{+}7 N{+}5\big ) S_1}{(N{+}1)^3 (N{+}2)^3}{+}\frac{32 (-1)^N \big (3 N^4+11 N^3+19 N^2+15 N+2\big )}{N (N+1)^3 (N+2)^3}{-}\frac{2 P_{178}}{9 (N-1) N^4 (N+1)^4 (N+2)^3}\nonumber \\&\quad -\frac{16\big (N^2{+}N{+}2\big ) \big (4 N^2{+}4 N{-}1\big ) S_2}{N^2 (N{+}1)^2 (N{+}2)}{+}\tilde{\gamma }_{qg}^{0} \Bigl [8 S_1^3{+}8 S_2 S_1{-}8 S_3{-}12 S_{-2}-8 S_{-3}+16 S_{-2,1}\Bigr ]\Biggr ]\nonumber \\&\quad +\frac{\big (3 N^5{+}8 N^4{+}27 N^3{+}46 N^2{+}20 N{+}8\big ) 16 (-1)^N S_1 S_{-2} }{N^2 (N{+}1)^2 (N+2)^2}{+}\frac{\big (N^2{+}N{+}2\big ) \big (3 N^2{+}3 N{+}2\big ) \Bigl [8 (-1)^N S_{-3}{+}6 (-1)^N \zeta _3\Bigr ]}{N^2 (N+1)^2 (N+2)}\nonumber \\ \end{aligned}$$

$$\begin{aligned}&\quad +\,\,\tilde{\gamma }_{qg}^{0} \Bigl [40 S_2 S_1^2+16 (-1)^N S_{-2} S_1^2 +\,\,8 (-1)^N S_{-3} S_1-16 S_{2,1} S_1+6 (-1)^N \zeta _3 S_1-8 S_2^2 \nonumber \\&\quad +\,\,24 S_{-2}^2+8 S_4+40 S_{-4} +\,\,32 S_{3,1}-16 S_{-3,1}-32 S_{-2,1,1}\Bigr ]\Biggr ] \nonumber \\&\quad +\,\,L_M \Biggl [ \frac{8 \big (3 N^5+2 N^4-61 N^3-112 N^2-56 N-24\big ) S_1^3}{N^2 (N+1)^2 (N+2)^2}-\,\,\frac{2 P_{151} S_1^2}{9 (N-1) N^3 (N+1)^3 (N+2)^3} \nonumber \\&\quad +\,\,\frac{32 (-1)^N \big (15 N^5+97 N^4+260 N^3+328 N^2+158 N-4\big ) S_1}{N (N+1)^3 (N+2)^4} -\,\,\frac{2 P_{192} S_1}{9 (N-1) N^4 (N+1)^4 (N+2)^4}\nonumber \\&\quad +\,\,\frac{8P_{56} S_2 S_1}{3 (N-1) N^2 (N+1)^2 (N+2)^2} +\,\,\frac{16 \big (3 N^3+2 N^2-47 N-62\big ) S_{-2} S_1}{N (N+1)^2 (N+2)}-\,\,6 \frac{\zeta _3 P_{92}}{(N-1) N^2 (N+1)^2 (N+2)^2} \nonumber \\&\quad -\,\,\frac{16 (-1)^N P_{209}}{5 (N-2) (N-1)^2 N^3 (N+1)^5 (N+2)^5 (N+3)^3}+\,\,\frac{2 P_{220}}{45 (N-1)^2 N^5 (N+1)^5 (N+2)^5 (N+3)^3}\nonumber \\&\quad +\,\,\frac{2 P_{152} S_2}{9 (N-1) N^3 (N+1)^3 (N+2)^3} +\,\,\frac{\big (N^3+4 N^2+7 N+5\big ) \big (128 (-1)^N S_1^2-128 (-1)^N S_2\big )}{(N+1)^3 (N+2)^3} \nonumber \\ \end{aligned}$$

72 $$\begin{aligned}&\quad -\,\,\frac{16 P_{70} S_3}{(N-1) N^2 (N+1)^2 (N+2)^2} -\,\,\frac{16 (-1)^N \big (3 N^5-6 N^4-61 N^3-124 N^2-96 N-16\big ) S_{-2}}{N (N+1)^3 (N+2)^3} \nonumber \\&\quad -\,\,\frac{16 P_{156} S_{-2}}{(N-2) N^3 (N+1)^3 (N+2)^3 (N+3)} +\,\,\frac{16 \big (3 N^4+4 N^3-9 N^2-14 N+8\big ) S_{-3}}{N^2 (N+1)^2 (N+2)} \nonumber \\&\quad +\,\,\frac{32 \big (N^2+N+2\big ) \big (10 N^4+20 N^3+5 N^2-5 N+6\big ) S_{2,1}}{3 (N-1) N^2 (N+1)^2 (N+2)^2}+\,\,\frac{16 \big (3 N^4+10 N^3+43 N^2+44 N-20\big ) S_{-2,1}}{N^2 (N+1)^2 (N+2)} \nonumber \\&\quad +\,\,\frac{\big (3 N^5+8 N^4+27 N^3+46 N^2+20 N+8\big ) \Bigl [-16 (-1)^N S_1 S_{-2} \Bigr ]}{N^2 (N+1)^2 (N+2)^2} \nonumber \\&\quad +\,\,\frac{\big (N^2+N+2\big ) \big (3 N^2+3 N+2\big ) \Bigl [-8 (-1)^N S_{-3}-6 (-1)^N \zeta _3\Bigr ]}{N^2 (N+1)^2 (N+2)} +\,\,\tilde{\gamma }_{qg}^{0} \Bigl [-8 S_1^4-32 S_2 S_1^2 -\,\,16 \big (1+(-1)^N\big ) S_{-2} S_1^2\nonumber \\&\quad -8 \big (1+(-1)^N\big ) S_{-3} S_1 +\,\,\Bigl [32 S_{2,1}-8 S_3\Bigr ] S_1 -\,\,6\big (3+(-1)^N\big ) \zeta _3 S_1 +8 S_2^2-24 S_{-2}^2 -8 S_4-40 S_{-4} \nonumber \\&\quad -\,\,32 S_{3,1} +16 S_{-3,1} +32 S_{-2,1,1} \Bigr ] \Biggr ]\Biggr ] +\,\,a_{Qg}^{(3),0} +\tilde{C}_{2,g}^{\mathsf{S},(3)}({N_F}+1) \Biggr \}\Biggr \}, \end{aligned}$$

with the polynomials

73 $$\begin{aligned} P_{43}&= N^6-81 N^5-264 N^4-185 N^3\nonumber \\&-\,\,307 N^2-256 N-204\end{aligned}$$

74 $$\begin{aligned} P_{44}&= N^6+6 N^5+7 N^4+4 N^3\nonumber \\&+18 N^2+16 N-8\end{aligned}$$

75 $$\begin{aligned} P_{45}&= N^6+7 N^5-7 N^4-39 N^3\nonumber \\&+\,\,14 N^2+40 N+48\end{aligned}$$

76 $$\begin{aligned} P_{46}&= N^6+21 N^5+57 N^4+31N^3\nonumber \\&+\,\,26 N^2+20 N+24\end{aligned}$$

77 $$\begin{aligned} P_{47}&= 2 N^6-7 N^5-41 N^4-31N^3\nonumber \\&-\,\,29 N^2-22 N-16\end{aligned}$$

78 $$\begin{aligned} P_{48}&= 2 N^6-7 N^5-24 N^4\nonumber \\&-\,\,35N^3-44 N^2-44 N-16\end{aligned}$$

79 $$\begin{aligned} P_{49}&= 3 N^6+5 N^5+27 N^4\nonumber \\&+\,\,35N^3+6 N^2+12 N+8\end{aligned}$$

80 $$\begin{aligned} P_{50}&= 3 N^6+9 N^5-N^4\nonumber \\&-\,\,17N^3-38 N^2-28 N-24\end{aligned}$$

81 $$\begin{aligned} P_{51}&= 3 N^6+9 N^5+2 N^4\nonumber \\&-\,\,11N^3-23 N^2-16 N-12\end{aligned}$$

82 $$\begin{aligned} P_{52}&= 3 N^6+30 N^5+15 N^4-64N^3\nonumber \\&-\,\,56 N^2-20 N-8\end{aligned}$$

83 $$\begin{aligned} P_{53}&= 4 N^6+5 N^5-10 N^4\nonumber \\&-\,\,39N^3-40 N^2-24 N-8\end{aligned}$$

84 $$\begin{aligned} P_{54}&= 6 N^6-12 N^5+17 N^4\nonumber \\&+\,\,106N^3+127 N^2+104 N+84\end{aligned}$$

85 $$\begin{aligned} P_{55}&= 6 N^6+18 N^5+7 N^4\nonumber \\&-\,\,16 N^3-31 N^2-20 N-12\end{aligned}$$

86 $$\begin{aligned} P_{56}&= 7 N^6-93 N^5-327 N^4\nonumber \\&-\,\,287 N^3-316 N^2-112 N-24\end{aligned}$$

87 $$\begin{aligned} P_{57}&= 7 N^6-20 N^5-176 N^4\nonumber \\&-\,\,335 N^3-276 N^2-116 N-16\end{aligned}$$

88 $$\begin{aligned} P_{58}&= 7 N^6-19 N^5-171 N^4\nonumber \\&-\,\,325 N^3-264 N^2-108 N-16\end{aligned}$$

89 $$\begin{aligned} P_{59}&= 7 N^6+21 N^5+5 N^4\nonumber \\&-\,\,25N^3-204 N^2-188 N-192\end{aligned}$$

90 $$\begin{aligned} P_{60}&= 8 N^6+13 N^5-111 N^4\nonumber \\&-\,\,193 N^3-89 N^2-56 N-20\end{aligned}$$

91 $$\begin{aligned} P_{61}&= 9 N^6+21 N^5+11 N^4\nonumber \\&-\,\,5 N^3-104 N^2-76 N-144\end{aligned}$$

92 $$\begin{aligned} P_{62}&= 9 N^6+39 N^5+53 N^4\nonumber \\&+\,\,25 N^3+94 N^2+44 N+312\end{aligned}$$

93 $$\begin{aligned} P_{63}&= 10 N^6+18 N^5-111 N^4\nonumber \\&-\,\,164 N^3-61 N^2-16 N+36\end{aligned}$$

94 $$\begin{aligned} P_{64}&= 10 N^6+63 N^5+105 N^4\nonumber \\&+\,\,31 N^3+17 N^2+14 N+48\end{aligned}$$

95 $$\begin{aligned} P_{65}&= 11 N^6-15 N^5-327 N^4\nonumber \\&-\,\,181 N^3+292 N^2-20 N-48\end{aligned}$$

96 $$\begin{aligned} P_{66}&= 11 N^6+15 N^5-285 N^4\nonumber \\&-\,\,319N^3-254 N^2-368 N-240\end{aligned}$$

97 $$\begin{aligned} P_{67}&= 11 N^6+33 N^5-189 N^4\nonumber \\&-\,\,361N^3-194 N^2-92 N-72\end{aligned}$$

98 $$\begin{aligned} P_{68}&= 11 N^6+33 N^5-114 N^4\nonumber \\&-\,\,247 N^3-263 N^2-176 N-108\end{aligned}$$

99 $$\begin{aligned} P_{69}&= 11 N^6+33 N^5-87 N^4\nonumber \\&-\,\,85 N^3+4 N^2-116 N-48\end{aligned}$$

100 $$\begin{aligned} P_{70}&= 11 N^6+35 N^5+59 N^4\nonumber \\&+\,\,57 N^3-38 N^2-68 N+40\end{aligned}$$

101 $$\begin{aligned} P_{71}&= 11 N^6+47 N^5+7 N^4\nonumber \\&+\,\,9 N^3+90 N^2+28 N+96\end{aligned}$$

102 $$\begin{aligned} P_{72}&= 11 N^6+57 N^5-39 N^4\nonumber \\&-\,\,109 N^3-44 N^2-116 N-48\end{aligned}$$

103 $$\begin{aligned} P_{73}&= 11 N^6+81 N^5+9 N^4\nonumber \\&-\,\,133 N^3-92 N^2-116 N-48\end{aligned}$$

104 $$\begin{aligned} P_{74}&= 13 N^6+36 N^5+39 N^4\nonumber \\&+\,\,8 N^3-21 N^2-29 N-10\end{aligned}$$

105 $$\begin{aligned} P_{75}&= 16 N^6+78 N^5-23 N^4\nonumber \\&-\,\,228 N^3-503 N^2-408 N-228\end{aligned}$$

106 $$\begin{aligned} P_{76}&= 17 N^6+111 N^5+234 N^4\nonumber \\&+\,\,203 N^3-89 N^2-296 N-36\end{aligned}$$

107 $$\begin{aligned} P_{77}&= 22 N^6+69 N^5+71 N^4\nonumber \\&+\,\,23 N^3-57 N^2-68 N+84\end{aligned}$$

108 $$\begin{aligned} P_{78}&= 23 N^6-7 N^5-237 N^4\nonumber \\&-\,\,593 N^3-678 N^2-548 N-200\end{aligned}$$

109 $$\begin{aligned} P_{79}&= 23 N^6+9 N^5-71 N^4\nonumber \\&-\,\,53 N^3-184 N^2-92 N-16\end{aligned}$$

110 $$\begin{aligned} P_{80}&= 25 N^6+35 N^5-55 N^4\nonumber \\&-\,\,243 N^3-286 N^2-204 N-72\end{aligned}$$

111 $$\begin{aligned} P_{81}&= 29 N^6+91 N^5+235 N^4\nonumber \\&+\,\,405 N^3+272 N^2+288 N+120\end{aligned}$$

112 $$\begin{aligned} P_{82}&= 29 N^6+176 N^5+777 N^4\nonumber \\&+\,\,1820 N^3+1878 N^2+776 N+232\end{aligned}$$

113 $$\begin{aligned} P_{83}&= 35 N^6-15 N^5-183 N^4\nonumber \\&-\,\,133 N^3-356 N^2-164 N-48\end{aligned}$$

114 $$\begin{aligned} P_{84}&= 35 N^6-15 N^5-101 N^4\nonumber \\&+\,\,31 N^3+54 N^2+164 N+120\end{aligned}$$

115 $$\begin{aligned} P_{85}&= 44 N^6+96 N^5+369 N^4\nonumber \\&+\,\,290 N^3-695 N^2-428 N-108\end{aligned}$$

116 $$\begin{aligned} P_{86}&= 55 N^6+141 N^5-195 N^4\nonumber \\&-\,\,401 N^3-772 N^2-748 N-384\end{aligned}$$

117 $$\begin{aligned} P_{87}&= 55 N^6+165 N^5-420 N^4\nonumber \\&-\,\,899 N^3-1561 N^2-1336 N-1188\end{aligned}$$

118 $$\begin{aligned} P_{88}&= 57 N^6+161 N^5-25 N^4\nonumber \\&-\,\,193 N^3-172 N^2-36 N+48\end{aligned}$$

119 $$\begin{aligned} P_{89}&= 65 N^6+199 N^5+197 N^4\nonumber \\&-\,\,143 N^3-330 N^2-316 N-120\end{aligned}$$

120 $$\begin{aligned} P_{90}&= 77 N^6+339 N^5-105 N^4\nonumber \\&-\,\,487 N^3-356 N^2-668 N-240\end{aligned}$$

121 $$\begin{aligned} P_{91}&= 80 N^6+60 N^5+9 N^4\nonumber \\&+\,\,230 N^3+901 N^2+988 N+1188\end{aligned}$$

122 $$\begin{aligned} P_{92}&= 81 N^6+211 N^5-23 N^4\nonumber \\&-\,\,355 N^3-334 N^2-4 N-344\end{aligned}$$

123 $$\begin{aligned} P_{93}&= 83 N^6+249 N^5-111 N^4\nonumber \\&-\,\,637 N^3-956 N^2-596 N-624\end{aligned}$$

124 $$\begin{aligned} P_{94}&= 130 N^6+865 N^5+2316 N^4\nonumber \\&+\,\,3811 N^3+4434 N^2+2884 N+536\end{aligned}$$

125 $$\begin{aligned} P_{95}&= 133 N^6+699 N^5+1395 N^4+\nonumber \\&217 N^3-880 N^2+164 N+288\end{aligned}$$

126 $$\begin{aligned} P_{96}&= 155 N^6+369 N^5+211 N^4\nonumber \\&-\,\,65 N^3-1002 N^2-556 N-1416\end{aligned}$$

127 $$\begin{aligned} P_{97}&= 215 N^6+429 N^5+891 N^4\nonumber \\&+\,\,491 N^3-2486 N^2-1436 N-408\end{aligned}$$

128 $$\begin{aligned} P_{98}&= 3 N^7+28 N^6+66 N^5+90 N^4\nonumber \\&+\,\,107 N^3+78 N^2+36 N+8\end{aligned}$$

129 $$\begin{aligned} P_{99}&= 9 N^7+71 N^6+214 N^5+320 N^4\nonumber \\&+\,\,275 N^3+215 N^2+160 N+32\end{aligned}$$

130 $$\begin{aligned} P_{100}&= 21 N^7+120 N^6-128 N^5-1038 N^4\nonumber \\&-\,\,89 N^3+2382 N^2+1636 N-600\end{aligned}$$

131 $$\begin{aligned} P_{101}&= 81 N^7+247 N^6+291 N^5+277 N^4\nonumber \\&+\,\,108 N^3-56 N^2+20 N+24\end{aligned}$$

132 $$\begin{aligned} P_{102}&= N^8+5 N^7+10 N^6+27 N^5+65 N^4\nonumber \\&+\,\,112 N^3+124 N^2+80 N+32\end{aligned}$$

133 $$\begin{aligned} P_{103}&= N^8+5 N^7+14 N^6+23 N^5+25 N^4\nonumber \\&+\,\,52 N^3+56 N^2+48 N+16\end{aligned}$$

134 $$\begin{aligned} P_{104}&= N^8+8 N^7-2 N^6-60 N^5-23 N^4\nonumber \\&+\,\,108 N^3+96 N^2+16 N+48\end{aligned}$$

135 $$\begin{aligned} P_{105}&= N^8+8 N^7-2 N^6-60 N^5+N^4\nonumber \\&+\,\,156 N^3+24 N^2-80 N-240\end{aligned}$$

136 $$\begin{aligned} P_{106}&= N^8+22 N^7+111 N^6+211 N^5\nonumber \\&+\,\,42 N^4-281 N^3-406 N^2-204 N-72\end{aligned}$$

137 $$\begin{aligned} P_{107}&= 2 N^8+N^7-6 N^6+26 N^5+64 N^4\nonumber \\&+\,\,51 N^3+54 N^2+28 N+8\end{aligned}$$

138 $$\begin{aligned} P_{108}&= 2 N^8+22 N^7+117 N^6+386 N^5\nonumber \\&+\,\,759 N^4+810 N^3+396 N^2+72 N+32\end{aligned}$$

139 $$\begin{aligned} P_{109}&= 2 N^8+44 N^7+211 N^6+485 N^5\nonumber \\&+\,\,654 N^4+581 N^3+391 N^2+192 N+32\end{aligned}$$

140 $$\begin{aligned} P_{110}&= 3 N^8+41 N^7+136 N^6+233 N^5\nonumber \\&+\,\,331 N^4+360 N^3+208 N^2+80 N+16\end{aligned}$$

141 $$\begin{aligned} P_{111}&= 3 N^8+54 N^7+118 N^6-44 N^5\nonumber \\&-\,\,353 N^4-314 N^3-272 N^2-200 N-144\end{aligned}$$

142 $$\begin{aligned} P_{112}&= 5 N^8-8 N^7-137 N^6-436 N^5\nonumber \\&-\,\,713 N^4-672 N^3-407 N^2-192 N-32\end{aligned}$$

143 $$\begin{aligned} P_{113}&= 7 N^8+40 N^7+110 N^6+193 N^5\nonumber \\&+\,\,261 N^4+313 N^3+260 N^2+96 N+16\end{aligned}$$

144 $$\begin{aligned} P_{114}&= 9 N^8+54 N^7+80 N^6-110 N^5\nonumber \\&-\,\,645 N^4-1168 N^3-1132 N^2-672 N-160\end{aligned}$$

145 $$\begin{aligned} P_{115}&= 10 N^8+46 N^7+87 N^6+85 N^5\nonumber \\&-\,\,75 N^4-251 N^3-274 N^2-132 N-72\end{aligned}$$

146 $$\begin{aligned} P_{116}&= 11 N^8+74 N^7+213 N^6+281 N^5\nonumber \\&-\,\,30 N^4-427 N^3-446 N^2-180 N-72\end{aligned}$$

147 $$\begin{aligned} P_{117}&= 15 N^8+36 N^7+50 N^6-252 N^5\nonumber \\&-\,\,357 N^4+152 N^3-68 N^2+88 N+48\end{aligned}$$

148 $$\begin{aligned} P_{118}&= 18 N^8+101 N^7+128 N^6+208 N^5\nonumber \\&+\,\,190 N^4-769 N^3-1200 N^2-212 N-48\end{aligned}$$

149 $$\begin{aligned} P_{119}&= 19 N^8+70 N^7+63 N^6-41 N^5\nonumber \\&-\,\,192 N^4-221 N^3-142 N^2-60 N-72\end{aligned}$$

150 $$\begin{aligned} P_{120}&= 21 N^8+42 N^7-38 N^6-360 N^5\nonumber \\&-\,\,631 N^4-730 N^3-472 N^2-216 N-48\end{aligned}$$

151 $$\begin{aligned} P_{121}&= 23 N^8+2 N^7-135 N^6+29 N^5\nonumber \\&+\,\,210 N^4-151 N^3-350 N^2-132 N-72\end{aligned}$$

152 $$\begin{aligned} P_{122}&= 27 N^8-36 N^7-956 N^6-1724 N^5\nonumber \\&+\,\,187 N^4+1288 N^3+70 N^2-224 N-72\end{aligned}$$

153 $$\begin{aligned} P_{123}&= 38 N^8+146 N^7+177 N^6+35 N^5\nonumber \\&-\,\,249 N^4-373 N^3-218 N^2-60 N-72\end{aligned}$$

154 $$\begin{aligned} P_{124}&= 41 N^8+5 N^7-195 N^6-97 N^5\nonumber \\&+\,\,326 N^4+424 N^3+208 N^2+72 N+16\end{aligned}$$

155 $$\begin{aligned} P_{125}&= 56 N^8+194 N^7+213 N^6+83 N^5\nonumber \\&-\,\,231 N^4-469 N^3-290 N^2-60 N-72\end{aligned}$$

156 $$\begin{aligned} P_{126}&= 79 N^8+196 N^7+132 N^6+274 N^5\nonumber \\&+\,\,465 N^4+82 N^3+332 N^2+456 N+288\end{aligned}$$

157 $$\begin{aligned} P_{127}&= 105 N^8+978 N^7+1688 N^6-1330 N^5\nonumber \\&-\,\,5245 N^4-4672 N^3-2212 N^2-544 N-288\end{aligned}$$

158 $$\begin{aligned} P_{128}&= 113 N^8+348 N^7+109 N^6-289 N^5\nonumber \\&-\,\,272 N^4-859 N^3-778 N^2-172 N+72\end{aligned}$$

159 $$\begin{aligned} P_{129}&= 170 N^8+369 N^7-521 N^6-1393 N^5\nonumber \\&-\,\,761 N^4-952 N^3-544 N^2+32 N+144\end{aligned}$$

160 $$\begin{aligned} P_{130}&= 264 N^8+1407 N^7+2246 N^6+1746 N^5\nonumber \\&+\,\,804 N^4-1069 N^3-674 N^2-92 N-24\end{aligned}$$

161 $$\begin{aligned} P_{131}&= 283 N^8+838 N^7+1482 N^6+628 N^5 \nonumber \\&-\,\,1497 N^4-1130 N^3-772 N^2+456 N+288\end{aligned}$$

162 $$\begin{aligned} P_{132}&= 633 N^8+2532 N^7+5036 N^6+6142 N^5\nonumber \\&+\,\,4275 N^4+1118 N^3-176 N^2-184 N-48\end{aligned}$$

163 $$\begin{aligned} P_{133}&= N^9+21 N^8+85 N^7+105 N^6+42 N^5\nonumber \\&+\,\,290 N^4+600 N^3+456 N^2+256 N+64\end{aligned}$$

164 $$\begin{aligned} P_{134}&= 4 N^9+53 N^8+193 N^7+233 N^6+87 N^5\nonumber \\&+\,\,554 N^4+1172 N^3+904 N^2+512 N+128\end{aligned}$$

165 $$\begin{aligned} P_{135}&= 6 N^9+93 N^8+576 N^7+1296 N^6+586 N^5\nonumber \\&+\,\,359 N^4+2000 N^3+1996 N^2 +1488 N+384\end{aligned}$$

166 $$\begin{aligned} P_{136}&= 9 N^9+54 N^8+56 N^7-110 N^6\nonumber \\&-\,\,381 N^5-568 N^4-364 N^3-72 N^2+128 N+96\nonumber \\ \end{aligned}$$

167 $$\begin{aligned} P_{137}&= 9 N^9+54 N^8+167 N^7+397 N^6+780 N^5+1241 N^4\nonumber \\&+\,\,1448 N^3+1200 N^2+608 N+144\end{aligned}$$

168 $$\begin{aligned} P_{138}&= 11 N^9+78 N^8+214 N^7+335 N^6+383 N^5\nonumber \\&+\,\,571 N^4+916 N^3+876 N^2+480 N+96\end{aligned}$$

169 $$\begin{aligned} P_{139}&= 35 N^9+150 N^8+232 N^7+137 N^6\nonumber \\&+\,\,119 N^5+661 N^4+1174 N^3+876 N^2+480 N+96\nonumber \\ \end{aligned}$$

170 $$\begin{aligned} P_{140}&= 37 N^9+210 N^8-52 N^7-2738 N^6-7249 N^5\nonumber \\&-\,\,9368 N^4-8216 N^3-5888 N^2-2448 N-576\nonumber \\\end{aligned}$$

171 $$\begin{aligned} P_{141}&= 45 N^9+270 N^8+820 N^7+1478 N^6+1683 N^5\nonumber \\&+\,\,1996 N^4+2356 N^3+2328 N^2 +1408 N+288\nonumber \\\end{aligned}$$

172 $$\begin{aligned} P_{142}&= 57 N^9+624 N^8+1756 N^7+1092 N^6-1803 N^5\nonumber \\&-\,\,1512 N^4+966 N^3+1116 N^2+920 N+528\nonumber \\\end{aligned}$$

173 $$\begin{aligned} P_{143}&= 69 N^9+366 N^8+1124 N^7+1966 N^6+2523 N^5\nonumber \\&+\,\,5228 N^4+7340 N^3+5352 N^2+3008 N+672\nonumber \\\end{aligned}$$

174 $$\begin{aligned} P_{144}&= 94 N^9+597 N^8+1616 N^7+2410 N^6+1841 N^5\nonumber \\&+\,\,1165 N^4+2191 N^3+3802 N^2+2916 N+648\nonumber \\\end{aligned}$$

175 $$\begin{aligned} P_{145}&= 121 N^9+696 N^8+1535 N^7+1585 N^6\nonumber \\&+\,\,416 N^5-749 N^4-836 N^3+16 N^2+528 N+144\nonumber \\ \end{aligned}$$

176 $$\begin{aligned} P_{146}&= 197 N^9+1242 N^8+2938 N^7+3524 N^6+2713 N^5\nonumber \\&+\,\,2234 N^4+3680 N^3+6176 N^2+4080 N+864\nonumber \\\end{aligned}$$

177 $$\begin{aligned} P_{147}&= 439 N^9+2634 N^8+6008 N^7\nonumber \\&+\,\,6694 N^6+3545 N^5+736 N^4+2008 N^3\nonumber \\&+\,\,6208 N^2+5136 N+1152\end{aligned}$$

178 $$\begin{aligned} P_{148}&= 538 N^9+3333 N^8+7802 N^7\nonumber \\&+\,\,7630 N^6+458 N^5-1415 N^4+7786 N^3\nonumber \\&+\,\,12340 N^2+5592 N+864\end{aligned}$$

179 $$\begin{aligned} P_{149}&= 664 N^9+3861 N^8+9038 N^7\nonumber \\&+\,\,11830 N^6+9344 N^5+3793 N^4+3874 N^3\nonumber \\&+\,\,11044 N^2+9624 N+2592\end{aligned}$$

180 $$\begin{aligned} P_{150}&= 891 N^9+4455 N^8+16078 N^7\nonumber \\&+\,\,28774 N^6+37047 N^5+45835 N^4+42192 N^3\nonumber \\&+\,\,28888 N^2+10640 N+1776\end{aligned}$$

181 $$\begin{aligned} P_{151}&= 923 N^9+5208 N^8+11824 N^7\nonumber \\&+\,\,12854 N^6+2185 N^5-7030 N^4\nonumber \\&+\,\,1436 N^3+15032 N^2+12864 N+3456\end{aligned}$$

182 $$\begin{aligned} P_{152}&= 965 N^9+4884 N^8+10816 N^7+20810 N^6\nonumber \\&+\,\,36895 N^5+40442 N^4+27692 N^3\nonumber \\&+\,\,22712 N^2+14496 N+3456\end{aligned}$$

183 $$\begin{aligned} P_{153}&= 2 N^{10}-46 N^9-98 N^8\nonumber \\&+\,\,282 N^7+1063 N^6+1569 N^5+1275 N^4\nonumber \\&+\,\,403 N^3-94 N^2-108 N-24\end{aligned}$$

184 $$\begin{aligned} P_{154}&= 2 N^{10}+12 N^9+24 N^8+11 N^7-48 N^6-151 N^5\nonumber \\&-\,\,282 N^4-480 N^3-664 N^2-576 N-288\end{aligned}$$

185 $$\begin{aligned} P_{155}&= 11 N^{10}+44 N^9+74 N^8\nonumber \\&+\,\,196 N^7+31 N^6-1426 N^5-3044 N^4\nonumber \\&-\,\,2762 N^3-1476 N^2 -480 N-96\end{aligned}$$

186 $$\begin{aligned} P_{156}&= 11 N^{10}+76 N^9+138 N^8-204 N^7\nonumber \\&-\,\,1041 N^6-988 N^5+752 N^4+1896 N^3\nonumber \\&+\,\,944 N^2-384 N-576\end{aligned}$$

187 $$\begin{aligned} P_{157}&= 37 N^{10}+392 N^9+2106 N^8+6514 N^7\nonumber \\&+\,\,9211 N^6+1258 N^5-9218 N^4-6116 N^3\nonumber \\&-\,\,72 N^2-752 N-192\end{aligned}$$

188 $$\begin{aligned} P_{158}&= 85 N^{10}+425 N^9+902 N^8+932 N^7\nonumber \\&-\,\,521 N^6-685 N^5+2022 N^4+2928 N^3\nonumber \\&+\,\,968 N^2 -1296 N-576 \end{aligned}$$

189 $$\begin{aligned} P_{159}&= 103 N^{10}+575 N^9+1124 N^8\nonumber \\&-\,\,334 N^7-1505 N^6+3755 N^5+4926 N^4\nonumber \\&+\,\,36 N^3-472 N^2 -2160 N-864\end{aligned}$$

190 $$\begin{aligned} P_{160}&= 118 N^{10}+425 N^9+197 N^8+86 N^7\nonumber \\&+\,\,1240 N^6+2489 N^5+4401 N^4+3480 N^3\nonumber \\&+\,\,524 N^2-1728 N-864\end{aligned}$$

191 $$\begin{aligned} P_{161}&= 118 N^{10}+557 N^9+461 N^8-94 N^7\nonumber \\&+\,\,1300 N^6+3521 N^5+4509 N^4+1920 N^3\nonumber \\&-\,\,1132 N^2 -2376 N-1008\end{aligned}$$

192 $$\begin{aligned} P_{162}&= 127 N^{10}+536 N^9+611 N^8+602 N^7\nonumber \\&+\,\,1474 N^6+2099 N^5+798 N^4\nonumber \\&-\,\,2301 N^3-4486 N^2-3708 N-936\end{aligned}$$

193 $$\begin{aligned} P_{163}&= 170 N^{10}+883 N^9+2041 N^8+2998 N^7\nonumber \\&-\,\,448 N^6-5465 N^5+129 N^4\nonumber \\&+\,\,6624 N^3+1132 N^2-2016 N-864\end{aligned}$$

194 $$\begin{aligned} P_{164}&= 170 N^{10}+1213 N^9+3235 N^8+2794 N^7\nonumber \\&-\,\,2692 N^6-3767 N^5-1293 N^4\nonumber \\&-\,\,1632 N^3-5324 N^2-6240 N-2016\end{aligned}$$

195 $$\begin{aligned} P_{165}&= 226 N^{10}+317 N^9-811 N^8\nonumber \\&+\,\,662 N^7+4552 N^6+3857 N^5+3933 N^4\nonumber \\&+\,\,2364 N^3 +236 N^2-1656 N-720\end{aligned}$$

196 $$\begin{aligned} P_{166}&= 489 N^{10}+2934 N^9+9364 N^8\nonumber \\&+\,\,18830 N^7+18627 N^6+124 N^5-19856 N^4\nonumber \\&-\,\,19296 N^3-10640 N^2-2880 N-1152\end{aligned}$$

197 $$\begin{aligned} P_{167}&= 3 N^{11}+42 N^{10}+144 N^9+74 N^8-459 N^7\nonumber \\&-\,\,1060 N^6-1152 N^5-1424 N^4-1688 N^3\nonumber \\&-\,\,1232 N^2-736 N-192\end{aligned}$$

198 $$\begin{aligned} P_{168}&= 11 N^{11}+37 N^{10}-27 N^9\nonumber \\&-\,\,118 N^8+21 N^7-249 N^6-1097 N^5\nonumber \\&-\,\,1138 N^4+552 N^3+3448 N^2\nonumber \\&+\,\,3456 N+2016\end{aligned}$$

199 $$\begin{aligned} P_{169}&= 21 N^{11}+231 N^{10}+1334 N^9+4086 N^8\nonumber \\&+\,\,6277 N^7+1775 N^6-9488 N^5-18076 N^4 \nonumber \\&-\,\,18208 N^3 -11344 N^2-5568 N-1728 \end{aligned}$$

200 $$\begin{aligned} P_{170}&= 33 N^{11}+231 N^{10}+698 N^9+1290 N^8\nonumber \\&+\,\,1513 N^7+1463 N^6+2236 N^5+5096 N^4\nonumber \\&+\,\,7328 N^3+5456 N^2+3456 N+1152\end{aligned}$$

201 $$\begin{aligned} P_{171}&= 45 N^{11}+383 N^{10}+958 N^9+526 N^8\nonumber \\&-\,\,763 N^7+1375 N^6+7808 N^5+13028 N^4\nonumber \\&+\,\,12976 N^3+8016 N^2+4608 N+1728\end{aligned}$$

202 $$\begin{aligned} P_{172}&= 51 N^{11}+269 N^{10}+46 N^9-1934 N^8-3973 N^7\nonumber \\&-\,\,875 N^6+7364 N^5+14972 N^4+16768 N^3\nonumber \\&+\,\,10896 N^2+5376 N+1728\end{aligned}$$

203 $$\begin{aligned} P_{173}&= 51 N^{11}+357 N^{10}+1238 N^9+2586 N^8+2755 N^7\nonumber \\&-\,\,1435 N^6-9212 N^5-15028 N^4-15280 N^3\nonumber \\&-\,\,9808 N^2-5184 N-1728\end{aligned}$$

204 $$\begin{aligned} P_{174}&= 81 N^{11}+483 N^{10}+1142 N^9+1086 N^8\nonumber \\&-\,\,767 N^7-4645 N^6-8936 N^5-11980 N^4\nonumber \\&-\,\,12352 N^3-8272 N^2-4800 N-1728\end{aligned}$$

205 $$\begin{aligned} P_{175}&= 120 N^{11}+1017 N^{10}+2737 N^9+1292 N^8\nonumber \\&-\,\,8086 N^7-20743 N^6-24563 N^5-16702 N^4\nonumber \\&-\,\,6840 N^3+120 N^2+2432 N+960\end{aligned}$$

206 $$\begin{aligned} P_{176}&= 243 N^{11}+1701 N^{10}+5378 N^9+10350 N^8\nonumber \\&+\,\,11479 N^7+1193 N^6-14684 N^5-20572 N^4\nonumber \\&-\,\,16288 N^3-8944 N^2-4992 N-1728\end{aligned}$$

207 $$\begin{aligned} P_{177}&= 333 N^{11}+2331 N^{10}+6556 N^9+9270 N^8\nonumber \\&+\,\,5081 N^7-6701 N^6-17554 N^5-20036 N^4\nonumber \\&-\,\,15680 N^3-9200 N^2-5664 N-1728\end{aligned}$$

208 $$\begin{aligned} P_{178}&= 753 N^{11}+4809 N^{10}+13174 N^9+20466 N^8\nonumber \\&+\,\,17717 N^7+6829 N^6+3908 N^5+15304 N^4\nonumber \\&+\,\,25408 N^3+20272 N^2+8448 N+1152\end{aligned}$$

209 $$\begin{aligned} P_{179}&= 837 N^{11}+7757 N^{10}+30120 N^9+68575 N^8\nonumber \\&+\,\,119176 N^7+191350 N^6+262979 N^5\nonumber \\&+\,\,258308 N^4+163106 N^3+63360 N^2\nonumber \\&+\,\,14848 N+1536\end{aligned}$$

210 $$\begin{aligned} P_{180}&= 1017 N^{11}+6195 N^{10}+14050 N^9\nonumber \\&+\,\,12738 N^8-2023 N^7-5093 N^6+27548 N^5\nonumber \\&+\,\,69760 N^4+80752 N^3+54064 N^2\nonumber \\&+\,\,20928 N+3456\end{aligned}$$

211 $$\begin{aligned} P_{181}&= 3 N^{12}+21 N^{11}+17 N^{10}-202 N^9-842 N^8\nonumber \\&-\,\,1924 N^7-3378 N^6-5059 N^5-6008 N^4\nonumber \\&-\,\,4860 N^3-2536 N^2-960 N-192\end{aligned}$$

212 $$\begin{aligned} P_{182}&= 9 N^{12}+63 N^{11}+38 N^{10}-414 N^9-1035 N^8\nonumber \\&-\,\,1341 N^7-1511 N^6-2972 N^5-6011 N^4\nonumber \\&-\,\,8038 N^3-6892 N^2-3432 N-864\end{aligned}$$

213 $$\begin{aligned} P_{183}&= 9 N^{12}+63 N^{11}+71 N^{10}-381 N^9-1536 N^8\nonumber \\&-\,\,2529 N^7-1946 N^6-1331 N^5-2096 N^4\nonumber \\&-\,\,4036 N^3-4144 N^2-2304 N-576\end{aligned}$$

214 $$\begin{aligned} P_{184}&= 39 N^{12}+585 N^{11}+2938 N^{10}+7136 N^9+9083 N^8\nonumber \\&+\,\,7745 N^7+14668 N^6+38246 N^5+59856 N^4\nonumber \\&+\,\,55560 N^3+32144 N^2+12480 N+2304\end{aligned}$$

215 $$\begin{aligned} P_{185}&= 48 N^{12}+459 N^{11}+2322 N^{10}+8290 N^9+20159 N^8\nonumber \\&+\,\,30862 N^7+28247 N^6+16109 N^5+9312 N^4\nonumber \\&+\,\,7488 N^3+4064 N^2+1328 N+192\end{aligned}$$

216 $$\begin{aligned} P_{186}&= 61 N^{12}+302 N^{11}+531 N^{10}+348 N^9-349 N^8\nonumber \\&-\,\,786 N^7+457 N^6+2524 N^5+2012 N^4+204 N^3\nonumber \\&-\,\,360 N^2-240 N-96\end{aligned}$$

217 $$\begin{aligned} P_{187}&= 92 N^{12}+796 N^{11}+3089 N^{10}\nonumber \\&+\,\,7550 N^9+10547 N^8+1029 N^7-19496 N^6\nonumber \\&-\,\,24199 N^5-8960 N^4+736 N^3\nonumber \\&+\,\,1744 N^2+816 N+192\end{aligned}$$

218 $$\begin{aligned} P_{188}&= 201 N^{12}+1845 N^{11}+6910 N^{10}\nonumber \\&+\,\,12854 N^9+8915 N^8-7741 N^7-17126 N^6\nonumber \\&-\,\,4294 N^5+16260 N^4+22080 N^3\nonumber \\&+\,\,12416 N^2+4128 N+576\end{aligned}$$

219 $$\begin{aligned} P_{189}&= 239 N^{12}+1338 N^{11}+3137 N^{10}+3164 N^9\nonumber \\&-\,\,983 N^8-6640 N^7-8123 N^6-4526 N^5\nonumber \\&-\,\,342 N^4+1232 N^3+848 N^2+256 N+32\end{aligned}$$

220 $$\begin{aligned} P_{190}&= 255 N^{12}+2169 N^{11}+6496 N^{10}\nonumber \\&+\,\,7694 N^9-127 N^8-6973 N^7+4132 N^6\nonumber \\&+\,\,25502 N^5+31956 N^4+22656 N^3\nonumber \\&+\,\,9632 N^2+864 N-576\end{aligned}$$

221 $$\begin{aligned} P_{191}&= 581 N^{12}+7035 N^{11}+37826 N^{10}\nonumber \\&+\,\,112904 N^9+190293 N^8+174327 N^7\nonumber \\&+\,\,92032 N^6+69438 N^5+78364 N^4\nonumber \\&+\,\,44464 N^3+11520 N^2-3168 N-1728\end{aligned}$$

222 $$\begin{aligned} P_{192}&= 825 N^{12}+7363 N^{11}+25396 N^{10}+40686 N^9\nonumber \\&+\,\,26213 N^8-12749 N^7-55498 N^6\nonumber \\&-\,\,89796 N^5-110552 N^4-134960 N^3\nonumber \\&-\,\,127584 N^2-64704 N-12672\end{aligned}$$

223 $$\begin{aligned} P_{193}&= 69 N^{13}+420 N^{12}+794 N^{11}\nonumber \\&-\,\,1357 N^{10}-10401 N^9-15678 N^8\nonumber \\&+\,\,532 N^7+239 N^6-40018 N^5-69432 N^4\nonumber \\&-\,\,69152 N^3-43792 N^2-18336 N-3456\end{aligned}$$

224 $$\begin{aligned} P_{194}&= 76 N^{13}+922 N^{12}+4479 N^{11}+9107 N^{10}\nonumber \\&-\,\,3747 N^9-52973 N^8-76133 N^7+42261 N^6\nonumber \\&+\,\,199307 N^5+123839 N^4-77470 N^3\nonumber \\&-\,\,84132 N^2-2160 N-432\end{aligned}$$

225 $$\begin{aligned} P_{195}&= 295 N^{13}+2387 N^{12}+8005 N^{11}\nonumber \\&+\,\,13687 N^{10}+10883 N^9+389 N^8-2641 N^7\nonumber \\&+\,\,6029 N^6+11034 N^5+6644 N^4+1384 N^3\nonumber \\&+\,\,80 N^2+128 N+64\end{aligned}$$

226 $$\begin{aligned} P_{196}&= 296 N^{13}+2368 N^{12}+10916 N^{11}\nonumber \\&+\,\,27006 N^{10}+23644 N^9-19764 N^8-61931 N^7\nonumber \\&-\,\,63733 N^6-52001 N^5-56865 N^4\nonumber \\&-\,\,38104 N^3+2664 N^2+7344 N+432\end{aligned}$$

227 $$\begin{aligned} P_{197}&= 377 N^{13}+4649 N^{12}+21813 N^{11}\nonumber \\&+\,\,38539 N^{10}-39339 N^9-272611 N^8-332971 N^7\nonumber \\&+\,\,220377 N^6+801934 N^5+384958 N^4-362030 N^3\nonumber \\&-\,\,297864 N^2-1080 N-864\end{aligned}$$

228 $$\begin{aligned} P_{198}&= 859 N^{13}+7376 N^{12}+25294 N^{11}\nonumber \\&+\,\,47088 N^{10}+63868 N^9+80876 N^8+63648 N^7\nonumber \\&-\,\,35856 N^6-146697 N^5-157168 N^4\nonumber \\&-\,\,91320 N^3-34800 N^2-8640 N-1152\end{aligned}$$

229 $$\begin{aligned} P_{199}&= 1211 N^{13}+5680 N^{12}+3338 N^{11}\nonumber \\&-\,\,17355 N^{10}-31517 N^9-48486 N^8-139667 N^7\nonumber \\&-\,\,278026 N^6-340745 N^5-269457 N^4-138568 N^3 \nonumber \\&-\,\,34632 N^2+9072 N+3888\end{aligned}$$

230 $$\begin{aligned} P_{200}&= 70 N^{14}+555 N^{13}+1599 N^{12}+1192 N^{11}\nonumber \\&-\,\,4430 N^{10}-13305 N^9-11835 N^8+8440 N^7\nonumber \\&+\,\,35816 N^6+57126 N^5+60340 N^4+44464 N^3\nonumber \\&+\,\,27808 N^2+12768 N+2880\end{aligned}$$

231 $$\begin{aligned} P_{201}&= 76 N^{14}+802 N^{13}+2979 N^{12}+1847 N^{11}\nonumber \\&-\,\,19377 N^{10}-58253 N^9-26543 N^8+170601 N^7\nonumber \\&+\,\,362177 N^6+225119 N^5-103240 N^4-193092 N^3\nonumber \\&-\,\,137160 N^2-117072 N-25920\end{aligned}$$

232 $$\begin{aligned} P_{202}&= 76 N^{14}+1042 N^{13}+5979 N^{12}+16367 N^{11}\nonumber \\&+\,\,11883 N^{10}-47693 N^9-125723 N^8-86079 N^7\nonumber \\&+\,\,36437 N^6+22559 N^5-51700 N^4+24828 N^3\nonumber \\&+\,\,132840 N^2+116208 N+25920\end{aligned}$$

233 $$\begin{aligned} P_{203}&= 4 N^{15}+50 N^{14}+267 N^{13}+765 N^{12}\nonumber \\&+\,\,1183 N^{11}+682 N^{10}-826 N^9-1858 N^8\nonumber \\&-\,\,1116 N^7+457 N^6+1500 N^5+2268 N^4+2400 N^3\nonumber \\&+\,\,1392 N^2+448 N+64\end{aligned}$$

234 $$\begin{aligned} P_{204}&= 26 N^{15}+314 N^{14}+1503 N^{13}+3222 N^{12}\nonumber \\&+\,\,2510 N^{11}+1996 N^{10}+15041 N^9+40728 N^8\nonumber \\&+\,\,54008 N^7+44956 N^6+31936 N^5+30416 N^4\nonumber \\&+\,\,29568 N^3+16704 N^2+5376 N+768\end{aligned}$$

235 $$\begin{aligned} P_{205}&= 101 N^{15}+1234 N^{14}+6867 N^{13}+21904 N^{12}\nonumber \\&+\,\,40098 N^{11}+32226 N^{10}-22057 N^9-86972 N^8\nonumber \\&-\,\,114557 N^7-111416 N^6-89204 N^5-37312 N^4\nonumber \\&+\,\,13392 N^3+23040 N^2+9792 N+1536\end{aligned}$$

236 $$\begin{aligned} P_{206}&= 390 N^{15}+5121 N^{14}+30556 N^{13}\nonumber \\&+\,\,114173 N^{12}+321958 N^{11}+771597 N^{10}\nonumber \\&+\,\,1583594 N^9+2637549 N^8+3381542 N^7\nonumber \\&+\,\,3199120 N^6+2183360 N^5+1123200 N^4\nonumber \\&+\,\,489952 N^3+178176 N^2+48384 N+6912\end{aligned}$$

237 $$\begin{aligned} P_{207}&= 75 N^{16}+1245 N^{15}+8291 N^{14}\nonumber \\&+\,\,27609 N^{13}+43437 N^{12}+14221 N^{11}\nonumber \\&-\,\,5995 N^{10}+182937 N^9+488696 N^8\nonumber \\&+\,\,296818 N^7-452292 N^6-730430 N^5\nonumber \\&-\,\,186180 N^4+259728 N^3\nonumber \\&+\,\,241056 N^2+116640 N+25920\end{aligned}$$

238 $$\begin{aligned} P_{208}&= 115 N^{16}+1838 N^{15}+11829 N^{14}\nonumber \\&+\,\,36114 N^{13}+30900 N^{12}-133946 N^{11}\nonumber \\&-\,\,454068 N^{10}-457420 N^9+249211 N^8\nonumber \\&+\,\,864716 N^7+312979 N^6-634466 N^5\nonumber \\&-\,\,587862 N^4-19556 N^3+104832 N^2\nonumber \\&+\,\,9504 N+1728\end{aligned}$$

239 $$\begin{aligned} P_{209}&= 185 N^{16}+2988 N^{15}+19694 N^{14}\nonumber \\&+\,\,62954 N^{13}+64470 N^{12}-207876 N^{11}\nonumber \\&-\,\,792388 N^{10}-861230 N^9+437231 N^8\nonumber \\&+\,\,1750616 N^7+869954 N^6-1016136 N^5\nonumber \\&-\,\,1130122 N^4-96596 N^3\nonumber \\&+\,\,199872 N^2+31104 N+1728\end{aligned}$$

240 $$\begin{aligned} P_{210}&= 939 N^{16}+10527 N^{15}+37207 N^{14}\nonumber \\&+\,\,18679 N^{13}-202006 N^{12}-617170 N^{11}\nonumber \\&-\,\,930025 N^{10}-882917 N^9-157123 N^8\nonumber \\&+\,\,1388549 N^7+2739376 N^6+2837500 N^5\nonumber \\&+\,\,2088640 N^4+1259696 N^3+622464 N^2\nonumber \\&+\,\,211392 N+34560\end{aligned}$$

241 $$\begin{aligned} P_{211}&= 1155 N^{16}+12417 N^{15}+37693 N^{14}\nonumber \\&-\,\,12293 N^{13}-285754 N^{12}-613900 N^{11}\nonumber \\&-\,\,571735 N^{10}-134309 N^9+778901 N^8\nonumber \\&+\,\,2698745 N^7+4995724 N^6+5915740 N^5\nonumber \\&+\,\,4978144 N^4+3161840 N^3+1498752 N^2\nonumber \\&+\,\,479808 N+76032\end{aligned}$$

242 $$\begin{aligned} P_{212}&= 1665 N^{16}+33005 N^{15}+287646 N^{14}\nonumber \\&+\,\,1402624 N^{13}+4031902 N^{12}+6199846 N^{11}\nonumber \\&+\,\,1054640 N^{10}-16668628 N^9-37272559 N^8\nonumber \\&-\,\,38892027 N^7-17387942 N^6+3962700 N^5\nonumber \\&+\,\,15625800 N^4+26960688 N^3+27379296 N^2\nonumber \\&+\,\,12985920 N+2332800\end{aligned}$$

243 $$\begin{aligned} P_{213}&= 87 N^{17}+1099 N^{16}+6055 N^{15}+19019 N^{14}\nonumber \\&+\,\,37119 N^{13}+45159 N^{12}+29583 N^{11}-2639 N^{10}\nonumber \\&-\,\,30218 N^9-40778 N^8-39994 N^7-35844 N^6\nonumber \\&-\,\,30808 N^5-30384 N^4-28256 N^3\nonumber \\&-\,\,16064 N^2-5248 N-768\end{aligned}$$

244 $$\begin{aligned} P_{214}&= 829 N^{17}+13413 N^{16}+83461 N^{15}+226391 N^{14}\nonumber \\&+\,\,55508 N^{13}-1239070 N^{12}-2862466 N^{11}\nonumber \\&-\,\,1217372 N^{10}+3372689 N^9+2779147 N^8\nonumber \\&-\,\,2705687 N^7+171733 N^6+8617302 N^5\nonumber \\&+\,\,5817902 N^4-3127236 N^3\nonumber \\&-\,\,3652560 N^2-336096 N-25920\end{aligned}$$

245 $$\begin{aligned} P_{215}&= 1407 N^{17}+18107 N^{16}+103463 N^{15}\nonumber \\&+\,\,347083 N^{14}+760095 N^{13}+1142715 N^{12}\nonumber \\&+\,\,1220067 N^{11}+983393 N^{10}+702746 N^9\nonumber \\&+\,\,533822 N^8+337702 N^7-3552 N^6-300296 N^5\nonumber \\&-\,\,332160 N^4-188128 N^3-63232 N^2\nonumber \\&-\,\,13184 N-1536\end{aligned}$$

246 $$\begin{aligned} P_{216}&= 95 N^{18}+3940 N^{17}+48989 N^{16}+308380 N^{15}\nonumber \\&+\,\,1166094 N^{14}+2843192 N^{13}+4428234 N^{12}\nonumber \\&+\,\,3171928 N^{11}-4692053 N^{10}-19875244 N^9\nonumber \\&-\,\,34305831 N^8-34774388 N^7-16392680 N^6\nonumber \\&+\,\,11584912 N^5+30493776 N^4+29700864 N^3\nonumber \\&+\,\,18783360 N^2+8294400 N+1866240\end{aligned}$$

247 $$\begin{aligned} P_{217}&= 325 N^{18}+4280 N^{17}+17759 N^{16}-14880 N^{15}\nonumber \\&-\,\,412326 N^{14}-1696848 N^{13}-3216546 N^{12}\nonumber \\&-\,\,1169232 N^{11}+8956857 N^{10}+23914216 N^9\nonumber \\&+\,\,31536899 N^8+25361392 N^7+9982840 N^6\nonumber \\&-\,\,10154128 N^5-26098704 N^4-26761536 N^3\nonumber \\&-\,\,17642880 N^2-8087040 N-1866240\end{aligned}$$

248 $$\begin{aligned} P_{218}&= 500 N^{18}+8215 N^{17}+56287 N^{16}+201810 N^{15}\nonumber \\&+\,\,361782 N^{14}+98826 N^{13}-759348 N^{12}\nonumber \\&-\,\,495786 N^{11}+3942186 N^{10}+11896133 N^9\nonumber \\&+\,\,16709737 N^8+13315736 N^7+3779660 N^6\nonumber \\&-\,\,7306454 N^5-14232852 N^4-13254768 N^3\nonumber \\&-\,\,8367840 N^2-3771360 N-855360\end{aligned}$$

249 $$\begin{aligned} P_{219}&= 150 N^{19}+2815 N^{18}+24285 N^{17}+131358 N^{16}\nonumber \\&+\,\,511310 N^{15}+1515954 N^{14}+3372978 N^{13}\nonumber \\&+\,\,5213980 N^{12}+4715522 N^{11}+980739 N^{10}\nonumber \\&-\,\,2709391 N^9-3741506 N^8-4630558 N^7\nonumber \\&-\,\,5623132 N^6-2333736 N^5+3419632 N^4\nonumber \\&+\,\,5238496 N^3+3231936 N^2+1123200 N\nonumber \\&+\,\,172800\end{aligned}$$

250 $$\begin{aligned} P_{220}&= 5410 N^{19}+98215 N^{18}+764965 N^{17}+3280996 N^{16}\nonumber \\&+\,\,8031920 N^{15}+8939378 N^{14}-7608074 N^{13}\nonumber \\&-\,\,44964380 N^{12}-74768226 N^{11}-57879177 N^{10}\nonumber \\&-\,\,5243187 N^9+13745888 N^8-28158216 N^7\nonumber \\&-\,\,49672024 N^6+14757808 N^5+94650144 N^4\nonumber \\&+\,\,100507392 N^3+53764992 N^2+15655680 N\nonumber \\&+\,\,1866240\end{aligned}$$

251 $$\begin{aligned} P_{221}&= 7060 N^{20}+123495 N^{19}+898682 N^{18}+3394183 N^{17}\nonumber \\&+\,\,6222824 N^{16}+376386 N^{15}-22032204 N^{14}\nonumber \\&-\,\,39912378 N^{13}-13976964 N^{12}+31985011 N^{11}\nonumber \\&+\,\,4994394 N^{10}-91499501 N^9-97243208 N^8\nonumber \\&+\,\,54501988 N^7+183103272 N^6+127073120 N^5\nonumber \\&-\,\,20272608 N^4-88410816 N^3-62225280 N^2\nonumber \\&-\,\,21772800 N-3110400. \end{aligned}$$

Our result for $H_{g,2}^{\mathsf{S}}$ differs from the one given in Eq. (B.7) in $z$-space in Ref. [36], which misses the term

252 $$\begin{aligned} C_F T_F^2 N_F \frac{4(N^2+N+2)}{N(N+1)(N+2)} \left[ 28 \zeta _2 -69\right] , \end{aligned}$$

given here in $N$-space. Note that the result of [36] is based on the calculation carried out by part of the present authors in Ref. [39], including the renormalization formulae being derived there. We have checked, however, that our result Eq. (72) is in full agreement with Eq. (2.15) of Ref. [39] and the moments having been calculated.

For the logarithmic contributions to the pure-singlet Wilson coefficient $H_{q,2}^{\mathsf{PS}}$, leaving the constant part of the un-renormalized OME $a_{Qq}^{\mathsf{PS},\mathrm (3),0}$ symbolic, we agree with the result given in [36]. Therefore, we refrain from presenting the corresponding expressions, also for the OME, contributing to the VFNS. The results on $A_{Qq}^{(3),\mathrm PS}$ will be given elsewhere.

## The asymptotic Wilson coefficients for the longitudinal structure function

The Wilson coefficients in the asymptotic region have been calculated in Ref. [53] for exclusive heavy flavor production, consisting of three contributions only. In total also here five Wilson coefficients contribute and the expressions are modified in the inclusive case of the complete structure function $F_L(x,Q^2)$ cf. [39]. In Mellin $N$-space they read:

253 $$\begin{aligned}&{L_{q,L}^{\mathsf{PS},(3)} = \tfrac{1}{2}[1 + (-1)^N]} \nonumber \\&\quad \times \Biggl \{{a_s^3} \Biggl \{{C_F} {N_F} {T_F^2} \Biggl [ \frac{128 L_Q^2 \big (N^2+N+2\big )}{3 (N-1) N (N+1)^2 (N+2)}\nonumber \\&\quad +\frac{128 \big (N^2+N+2\big ) L_M^2}{3 (N-1) N (N+1)^2 (N+2)}\nonumber \\&\quad -\frac{256 L_Q \big (11 N^5+35 N^4+59 N^3+55 N^2-4 N-12\big )}{9 (N-1) N^2 (N+1)^3 (N+2)^2}\nonumber \\&\quad +\Biggl [ \frac{256 \big (8 N^3+13 N^2+27 N+16\big )}{9 (N-1) N (N+1)^3 (N+2)} \nonumber \\&\quad -\frac{256 \big (N^2+N+2\big ) S_1}{3 (N-1) N (N+1)^2 (N+2)}\Biggr ]L_M \nonumber \\&\quad +\frac{64 \big (N^2+N+2\big ) \big [S_1^2+ S_2\big ]}{3(N-1) N (N+1)^2 (N+2)} \nonumber \\&\quad -\frac{128 \big (8 N^3+13 N^2+27 N+16\big ) S_1}{9 (N-1) N (N+1)^3(N+2)}\nonumber \\&\quad +\frac{128 \big (43 N^4+105 N^3+224 N^2+230 N+86\big )}{27 (N-1) N (N+1)^4 (N+2)}\Biggr ] \nonumber \\&\quad +{N_F} \hat{\tilde{C}}_{L,q}^{\mathsf{PS},(3)}({N_F})\Biggr \} \Biggr \}, \end{aligned}$$

$$\begin{aligned}&{L_{g,L}^{\mathsf{S}} = \tfrac{1}{2}[1 + (-1)^N]}\nonumber \\&\quad \times \Biggl \{{a_s^2} \frac{64 {N_F} {T_F^2} L_M }{3 (N+1) (N+2)} +{a_s^3} \Biggl \{{N_F} {T_F^3} \frac{256 L_M^2 }{9 (N+1) (N+2)}\nonumber \\&\quad +{C_A} {N_F} {T_F^2} \Biggl [ \Biggl [\frac{256 \big (N^2+N+1\big )}{3 (N-1) N (N+1)^2(N+2)^2}\nonumber \\&\quad -\frac{128 S_1}{3 (N+1) (N+2)}\Biggr ] L_Q^2\nonumber \\&\quad +\Biggl [\frac{256 (-1)^N \big (N^3+4 N^2+7 N+5\big )}{3 (N+1)^3 (N+2)^3}\nonumber \\&\quad +\frac{64 Q_2}{9 (N-1) N (N+1)^3 (N+2)^3}\nonumber \\&\quad +\frac{256 \big (11 N^3-6 N^2-8 N-3\big ) S_1}{9 (N-1) N (N+1)^2 (N+2)}\nonumber \\&\quad +L_M \Biggl [\frac{512 \big (N^2+N+1\big )}{3 (N-1) N (N+1)^2(N+2)^2}\nonumber \\&\quad -\frac{256 S_1}{3 (N+1) (N+2)}\Biggr ]\nonumber \\&\quad +\frac{1}{(N+1) (N+2)} \Bigl [\frac{128}{3} S_1^2 -\frac{128 S_2}{3}\nonumber \\&\quad -\frac{256}{3} S_{-2}\Bigr ]\Biggr ] L_Q +\frac{32 Q_5}{27 (N-1) N^3 (N+1)^4 (N+2)^2}\nonumber \\&\quad -\frac{64 (56 N+47) S_1}{27 (N+1)^2 (N+2)}\nonumber \\&\quad +L_M^2 \Biggl [ \frac{256 \big (N^2+N+1\big )}{3 (N-1) N (N+1)^2 (N+2)^2} -\frac{128 S_1}{3 (N+1) (N+2)}\Biggr ]\nonumber \\&\quad +L_M \Biggl [ \frac{256 (-1)^N \big (N^3+4 N^2+7 N+5\big )}{3 (N+1)^3(N+2)^3}\nonumber \\ \end{aligned}$$

254 $$\begin{aligned}&\quad +\frac{128 Q_4}{9 (N-1) N^2 (N+1)^3 (N+2)^3}\nonumber \\&\quad +\frac{256 \big (N^3-6 N^2+2 N-3\big ) S_1}{9 (N-1) N (N+1)^2 (N+2)} +\frac{\frac{128}{3} S_1^2 -\frac{128 S_2}{3} -\frac{256}{3} S_{-2}}{(N+1) (N+2)}\Biggr ]\Biggr ]\nonumber \\&\quad +{C_F} {T_F^2} {N_F} \Biggl [ \frac{64 \big (N^2+N+2\big ) \big (N^4+2 N^3+2 N^2+N+6\big ) L_Q^2}{3 (N-1) N^2 (N+1)^3 (N+2)^2}\nonumber \\&\quad +\Biggl [\frac{256 (-1)^N Q_6}{45 (N-2) (N-1)^2 N^2 (N+1)^3 (N+2)^2 (N+3)^3}\nonumber \\&\quad -\frac{32 Q_9}{45 (N-1)^2 N^3 (N+1)^4 (N+2)^3 (N+3)^3}\nonumber \\&\quad -\frac{128 Q_1 S_1}{3 (N-1) N^2 (N+1)^3 (N+2)^2}+\frac{256 (N-1) S_{-2}}{3 (N-2) (N+1) (N+3)}\nonumber \\&\quad +\frac{64 \big (N^2+N+2\big ) \big (N^4+2 N^3-7 N^2-8 N-12\big ) L_M}{3 (N-1) N^2 (N+1)^3 (N+2)^2}\Biggr ] L_Q\nonumber \\&\quad +\frac{64 \big (N^2+N+2\big )^2 L_M^2}{(N-1) N^2 (N+1)^3 (N+2)^2} -\frac{16 Q_7}{(N-1) N^4 (N+1)^5 (N+2)^2}\nonumber \\&\quad +L_M \Biggl [\frac{256 (-1)^N Q_6}{45 (N-2) (N-1)^2 N^2 (N+1)^3 (N+2)^2 (N+3)^3} \nonumber \\&\quad +\frac{64 Q_8}{45 (N-1)^2 N^3 (N+1)^4 (N+2)^3 (N+3)^3}\nonumber \\&\quad -\frac{64 Q_3 S_1}{3 (N-1) N^2 (N+1)^3 (N+2)^2} \nonumber \\&\quad +\frac{256 (N-1) S_{-2}}{3 (N-2) (N+1) (N+3)}\Biggr ]\Biggr ] +{N_F} \hat{\tilde{C}}_{L,g}^{\mathsf{S},(3)}({N_F})\Biggr \}\Biggr \}, \end{aligned}$$

with

255 $$\begin{aligned} Q_1&= 2 N^6+6 N^5+7 N^4+4 N^3+9 N^2+8 N+12\end{aligned}$$

256 $$\begin{aligned} Q_2&= 3 N^6+3 N^5-121 N^4-391 N^3\nonumber \\&-474 N^2-308 N-80\end{aligned}$$

257 $$\begin{aligned} Q_3&= 3 N^6+9 N^5-N^4-17 N^3-38 N^2\nonumber \\&-28 N-24\end{aligned}$$

258 $$\begin{aligned} Q_4&= 6 N^7+24 N^6+47 N^5+104 N^4\nonumber \\&+219 N^3+316 N^2+208 N+48\end{aligned}$$

259 $$\begin{aligned} Q_5&= 15 N^8+60 N^7+572 N^6+1470 N^5\nonumber \\&+2135 N^4+1794 N^3+722 N^2-24 N-72\end{aligned}$$

260 $$\begin{aligned} Q_6&= N^{10}-13 N^9-39 N^8+222 N^7\nonumber \\&+1132 N^6+1787 N^5+913 N^4\nonumber \\&+392 N^3+645 N^2-324 N-108\end{aligned}$$

261 $$\begin{aligned} Q_7&= 15 N^{10}+75 N^9+112 N^8+14 N^7\nonumber \\&-61 N^6+107 N^5+170 N^4+36 N^3\nonumber \\&-36 N^2-32 N-16\end{aligned}$$

262 $$\begin{aligned} Q_8&= 45 N^{13}+656 N^{12}+4397 N^{11}\nonumber \\&+17513 N^{10}+43665 N^9+63005 N^8\nonumber \\&+27977 N^7-71993 N^6-140386 N^5\nonumber \\&-78985 N^4+25350 N^3+80460 N^2\nonumber \\&+100008 N+38880\end{aligned}$$

263 $$\begin{aligned} Q_9&= 95 N^{13}+1218 N^{12}+6096 N^{11}\nonumber \\&+14484 N^{10}+11570 N^9-28440 N^8\nonumber \\&-117844 N^7-225884 N^6-238953 N^5\nonumber \\&-83290 N^4+57660 N^3+122040 N^2\nonumber \\&+182304 N+77760, \end{aligned}$$

$$\begin{aligned}&{L_{q,L}^\mathsf{NS} = \tfrac{1}{2}[1 + (-1)^N]} \nonumber \\&\quad \times \Biggl \{{a_s^2} {C_F} {T_F} \Biggl \{\frac{16 L_Q}{3 (N+1)} -\frac{8 \big (19 N^2+7 N-6\big )}{9 N (N+1)^2}\nonumber \\&\quad -\frac{16 S_1}{3 (N+1)}\Biggr \}\nonumber \\&\quad +{a_s^3} \Biggl \{ {C_F^2} {T_F} \Biggl [ \Biggl [\frac{8 \big (3 N^2+3 N+2\big )}{N (N+1)^2} -\frac{32 S_1}{N+1}\Biggr ] L_Q^2\nonumber \\&\quad +\Biggl [\frac{256 (-1)^N Q_{11}}{15 (N-2) (N-1)^2 N^2 (N+1)^4 (N+2)^2 (N+3)^3}\nonumber \\&\quad -\frac{32 Q_{12}}{45 (N-1)^2 N^2 (N+1)^4 (N+2)^2(N+3)^3}\nonumber \\&\quad -\frac{16 (N+10) (5 N+3) S_1}{9 N (N+1)^2}\nonumber \\&\quad +\frac{512 \big (N^4+2 N^3-N^2-2 N-6\big ) S_{-2}}{3 (N-2) N (N+1)^2(N+3)}\nonumber \\&\quad +\frac{1}{N+1} \Bigl [\frac{128}{3} S_1^2-\frac{512}{3} S_{-2} S_1-\frac{128 S_2}{3}-\frac{256 S_3}{3}\nonumber \\&\quad -\frac{256}{3} S_{-3}+\frac{512}{3} S_{-2,1}+256 \zeta _3\Bigr ]\Biggr ]L_Q +\frac{2 Q_{10}}{27 N^3 (N+1)^4}\nonumber \\&\quad +L_M^2 \Biggl [\frac{8 \big (3 N^2+3 N+2\big )}{3 N (N+1)^2} -\frac{32 S_1}{3 (N+1)}\Biggr ]\nonumber \\ \end{aligned}$$

264 $$\begin{aligned}&\quad +L_M \Biggl [\frac{8 \big (3 N^4+6 N^3+47 N^2+20 N-12\big )}{9 N^2 (N+1)^3}\nonumber \\&\quad +\frac{\frac{64 S_2}{3} -\frac{320 S_1}{9}}{N+1}\Biggr ]\nonumber \\&\quad +\frac{-\frac{896}{27} S_1+\frac{160 S_2}{9}-\frac{32 S_3}{3}}{N+1}\Biggr ] +{C_F} {T_F^2} \Biggl [\frac{64 L_Q^2}{9 (N+1)}\nonumber \\&\quad +\Biggl [-\frac{64 \big (19 N^2+7 N-6\big )}{27 N (N+1)^2} -\frac{128 S_1}{9 (N+1)}\Biggr ] L_Q \Biggr ]\nonumber \\&\quad + {C_F} {T_F^2} {N_F} \Biggl [ \frac{128 L_Q^2}{9 (N+1)} +\Biggl [-\frac{128 \big (19 N^2+7 N-6\big )}{27 N (N+1)^2}\nonumber \\&\quad -\frac{256 S_1}{9 (N+1)}\Biggr ] L_Q\Biggr ] +{C_F} {C_A} {T_F} \Biggl [L_Q \Biggl [\nonumber \\&\quad -\frac{128 (-1)^N Q_{11}}{15 (N-2) (N-1)^2 N^2 (N+1)^4 (N+2)^2(N+3)^3}\nonumber \\&\quad -\frac{256 \big (N^4+2 N^3-N^2-2 N-6\big ) S_{-2}}{3 (N-2) N (N+1)^2 (N+3)}\nonumber \\&\quad +\frac{16 Q_{13}}{135 (N-1)^2 N^2 (N+1)^4 (N+2)^2(N+3)^3}\nonumber \\&\quad +\frac{1}{N+1}\Bigl [\frac{256}{3} S_{-2} S_1 +\frac{1088 S_1}{9}+\frac{128 S_3}{3}\nonumber \\&\quad +\frac{128}{3} S_{-3} -\frac{256}{3} S_{-2,1}-128 \zeta _3\Bigr ]\Biggr ] -\frac{352 L_Q^2}{9 (N+1)}\Biggr ]\nonumber \\&\quad +\hat{C}_{L,q}^{\mathsf{NS},(3)}(N_F)\Biggr \} \Biggr \}, \end{aligned}$$

with

265 $$\begin{aligned} Q_{10}&= 219 N^6+657 N^5+1193 N^4+763 N^3-40 N^2\nonumber \\&-48 N+72\end{aligned}$$

266 $$\begin{aligned} Q_{11}&= 2 N^{11}+41 N^{10}+226 N^9+556 N^8+963 N^7\nonumber \\&+2733 N^6+7160 N^5+8610 N^4+1969 N^3\nonumber \\&-2748 N^2-864 N-216\end{aligned}$$

267 $$\begin{aligned} Q_{12}&= 180 N^{12}+2385 N^{11}+11798 N^{10}+23030 N^9\nonumber \\&-10466 N^8-131068 N^7-245294 N^6-196786 N^5\nonumber \\&-22282 N^4+86571 N^3+50688 N^2-7236 N-3888\nonumber \\\end{aligned}$$

268 $$\begin{aligned} Q_{13}&= 2345 N^{12}+31510 N^{11}+163614 N^{10}\nonumber \\&+380250 N^9+208092 N^8-794874 N^7\nonumber \\&-1604762 N^6-833938 N^5+451419 N^4\nonumber \\&+584028 N^3+113724 N^2-36288 N+7776, \end{aligned}$$

$$\begin{aligned}&{H_{q,L}^{\mathsf{PS}} = \tfrac{1}{2}[1 + (-1)^N]}\nonumber \\&\quad \times \Biggl \{ {a_s^2} {C_F} {T_F} \Biggl \{ -\frac{32 S_1 \big (N^2+N+2\big )}{(N-1) N (N+1)^2 (N+2)}\nonumber \\&\quad +\frac{32 L_Q \big (N^2+N+2\big )}{(N-1) N (N+1)^2 (N+2)}\nonumber \\&\quad -\frac{32 \big (N^5+2 N^4+2 N^3-5 N^2-12 N-4\big )}{(N-1) N^2 (N+1)^3 (N+2)^2}\Biggr \}\nonumber \\&\quad +{a_s^3} \Biggl \{{C_F^2} {T_F} \Biggl [\Biggl [ \frac{64 \big (N^2+N+1\big ) \big (N^2+N+2\big )}{(N-1) N^2 (N+1)^3 (N+2)}\nonumber \\&\quad -\frac{64 \big (N^2+N+2\big ) S_1}{(N-1) N (N+1)^2 (N+2)}\Biggr ]L_Q^2\nonumber \\&\quad +\Biggl [ \frac{128 (-1)^N \big (N^2+N+2\big ) Q_{16}}{15 (N-2) (N-1)^3 N^3 (N+1)^4 (N+2)^2 (N+3)^3}\nonumber \\&\quad -\frac{32 Q_{18}}{15 (N-1)^3 N^3 (N+1)^4 (N+2)^2 (N+3)^3}\nonumber \\&\quad +\frac{128 \big (2 N^5+5 N^4+7 N^3+2 N^2-12 N-8\big ) S_1}{(N-1) N^2 (N+1)^3 (N+2)^2} \nonumber \\&\quad +\frac{\big (N^2+N+2\big ) \big (64 S_1^2-64 S_2\big )}{(N-1) N (N+1)^2(N+2)}\nonumber \\&\quad +\frac{128 \big (N^2+N+2\big ) S_{-2}}{(N-2) N (N+1)^2 (N+3)}\Biggr ] L_Q\nonumber \\ \end{aligned}$$

$$\begin{aligned}&\quad -\frac{16 \big (N^2+N+2\big )^2 L_M^2}{(N-1) N^2 (N+1)^3(N+2)}\nonumber \\&\quad +\frac{16 Q_{17}}{(N-1) N^4 (N+1)^5 (N+2)^3}\nonumber \\&\quad -\frac{32 \big (N^2+N+2\big )^2 S_2}{(N-1) N^2 (N+1)^3 (N+2)}\nonumber \\&\quad -\frac{32 \big (N^2+5 N+2\big ) \big (5 N^3+7 N^2+4 N+4\big ) L_M}{(N-1) N^3 (N+1)^4 (N+2)^2} \Biggr ]\nonumber \\&\quad + {C_F}{T_F^2} {N_F}\Biggl [ \frac{128 L_Q^2 \big (N^2+N+2\big )}{3 (N-1) N (N+1)^2 (N+2)}\nonumber \\&\quad -\frac{256 L_Q \big (11 N^5\!+\!35 N^4\!+\!59 N^3\!+\!55 N^2\!-\!4 N\!-\!12\big )}{9 (N-1) N^2 (N+1)^3(N+2)^2}\Biggr ]\nonumber \\&\quad + {C_F} {T_F^2} \Biggl [ \frac{128 \big (N^2+N+2\big ) L_Q^2}{3 (N-1) N (N+1)^2 (N+2)}\nonumber \\&\quad -\frac{256 \big (11 N^5+35 N^4+59 N^3+55 N^2-4 N-12\big ) L_Q}{9 (N-1) N^2 (N+1)^3(N+2)^2}\nonumber \\&\quad +\frac{128 \big (N^2+N+2\big ) L_M^2}{3 (N-1)N (N+1)^2 (N+2)}\nonumber \\&\quad +\frac{128 \big (43 N^4+105 N^3+224 N^2+230 N+86\big )}{27 (N-1) N (N+1)^4(N+2)}\nonumber \\&\quad -\frac{128 \big (8 N^3+13 N^2+27 N+16\big ) S_1}{9 (N-1) N (N+1)^3 (N+2)}\nonumber \\ \end{aligned}$$

269 $$\begin{aligned}&\quad +L_M \Biggl [\frac{256 \big (8 N^3+13 N^2+27 N+16\big )}{9 (N-1) N (N+1)^3 (N+2)}\nonumber \\&\quad -\frac{256 \big (N^2+N+2\big ) S_1}{3 (N-1) N (N+1)^2 (N+2)}\Biggr ]\nonumber \\&\quad +\frac{\big (N^2+N+2\big ) \Bigl [\frac{64}{3} S_1^2+\frac{64 S_2}{3}\Bigr ]}{(N-1) N (N+1)^2 (N+2)}\Biggr ]\nonumber \\&\quad +{C_F} {C_A} {T_F}\nonumber \\&\quad \!\times \Biggl [\Biggl [-\frac{32 \big (N^2\!+\!N\!+\!2\big ) \big (11 N^4\!+\!22 N^3\!-\!23 N^2\!-\!34 N\!-\!12\big )}{3 (N\!-\!1)^2 N^2 (N\!+\!1)^3 (N\!+\!2)^2}\nonumber \\&\quad -\frac{64 \big (N^2+N+2\big ) S_1}{(N-1) N (N+1)^2 (N+2)}\Biggr ]L_Q^2\nonumber \\&\quad +\Biggl [\frac{128 (-1)^N Q_{14}}{(N-1) N^2 (N+1)^4 (N+2)^3}\nonumber \\&\quad +\frac{64 Q_{15}}{9 (N-1)^2 N^3 (N+1)^3 (N+2)^3}\nonumber \\&\quad +\frac{128 \big (N^4-N^3-4 N^2-11 N-1\big ) S_1}{(N-1)^2 N (N+1)^3 (N+2)}\nonumber \\&\quad +\frac{\big (N^2+N+2\big ) \Bigl [128 S_1^2-128 S_ 2-256 S_{-2}\Bigr ]}{(N-1) N (N+1)^2 (N+2)}\Biggr ]L_Q\Biggr ]\nonumber \\&\quad +\tilde{C}_{L,q}^{\mathsf{PS},(3)}\big ({N_F}+1)\Biggr \} \Biggr \}, \end{aligned}$$

with

270 $$\begin{aligned} Q_{14}&= N^6+8 N^5+30 N^4+58 N^3+65 N^2+42 N+8\nonumber \\\end{aligned}$$

271 $$\begin{aligned} Q_{15}&= 142 N^8+593 N^7+801 N^6+199 N^5\nonumber \\&-1067 N^4-900 N^3+976 N^2+1128 N+288\nonumber \\\end{aligned}$$

272 $$\begin{aligned} Q_{16}&= N^{10}-13 N^9-39 N^8+222 N^7\nonumber \\&+1132 N^6+1787 N^5+913 N^4\nonumber \\&+392 N^3+645 N^2-324 N-108\end{aligned}$$

273 $$\begin{aligned} Q_{17}&= N^{10}+8 N^9+29 N^8+49 N^7\nonumber \\&-11 N^6-131 N^5-161 N^4-160 N^3\nonumber \\&-168 N^2-80 N-16\end{aligned}$$

274 $$\begin{aligned} Q_{18}&= 225 N^{12}+2494 N^{11}\nonumber \\&+9980 N^{10}+14480 N^9-11602 N^8\nonumber \\&-68380 N^7-86828 N^6-15080 N^5\nonumber \\&+67401 N^4+60334 N^3-312 N^2\nonumber \\&-33912 N-12528, \end{aligned}$$

and

$$\begin{aligned}&{H_{g,L}^{\mathsf{S}} = \tfrac{1}{2}[1 + (-1)^N]}\nonumber \\&\quad \times \Biggl \{ {a_s} {T_F} \frac{16 }{(N+1)(N+2)} +{a_s^2} \Biggl \{\frac{64 L_M {T_F^2}}{3 (N+1) (N+2)}\nonumber \\&\quad +{C_A} {T_F} \Biggl [ \frac{64 (-1)^N \big (N^3+4 N^2+7 N+5\big )}{(N+1)^3 (N+2)^3}\nonumber \\&\quad -\frac{32 \big (2 N^5+9 N^4+5 N^3-12 N^2-20 N-8\big )}{(N-1) N^2 (N+1)^2 (N+2)^3}\nonumber \\&\quad +\frac{64 \big (2 N^3\!-\!2 N^2\!-\!N\!-\!1\big ) S_1}{(N\!-\!1) N (N\!+\!1)^2 (N\!+\!2)}\nonumber \\&\quad +L_Q \Biggl [ \frac{128 \big (N^2+N+1\big )}{(N-1) N (N+1)^2 (N+2)^2} -\frac{64 S_1}{(N+1) (N+2)}\Biggr ]\nonumber \\&\quad +\frac{32 S_1^2-32 S_2-64 S_{-2}}{(N+1) (N+2)}\Biggr ]\nonumber \\&\quad +{C_F} {T_F}\Biggl [ -\frac{16 L_M \big (N^2+N+2\big )}{N (N+1)^2 (N+2)} +\frac{16 L_Q \big (N^2+N+2\big )}{N (N+1)^2 (N+2)}\nonumber \\&\quad +\frac{16 Q_{29}}{15 (N-1)^2 N^2 (N+1)^3 (N+2)^2 (N+3)^3}\nonumber \\&\quad +\frac{64 (-1)^N Q_{30}}{15 (N-2) (N-1)^2 N^2 (N+1)^3 (N+2)^2 (N+3)^3}\nonumber \\&\quad -\frac{16 \big (3 N^2+3 N+2\big ) S_1}{N (N+1)^2 (N+2)}+\frac{64 (N-1) S_{-2}}{(N-2) (N+1) (N+3)}\Biggr ] \Biggr \}\nonumber \\&\quad +{a_s^3} \Biggl \{ \frac{256 L_M^2 {T_F^3}}{9 (N+1) (N+2)}+{C_A} {T_F^2}\Biggl [ \Biggl [\frac{256 \big (N^2\!+\!N\!+\!1\big )}{3 (N\!-\!1) N (N\!+\!1)^2 (N\!+\!2)^2}\nonumber \\&\quad -\frac{128 S_1}{3 (N\!+\!1) (N\!+\!2)}\Biggr ] L_Q^2\!+\!\Biggl [ \frac{256 (-1)^N \big (N^3\!+\!4 N^2\!+\!7 N\!+\!5\big )}{3 (N\!+\!1)^3 (N\!+\!2)^3}\nonumber \\ \end{aligned}$$

$$\begin{aligned}&\quad +\frac{64 Q_{22}}{9 (N\!-\!1) N (N\!+\!1)^3 (N\!+\!2)^3}+\frac{256 \big (11 N^3\!-\!6 N^2\!-\!8 N\!-\!3\big ) S_1}{9 (N\!-\!1) N (N\!+\!1)^2 (N\!+\!2)} \nonumber \\&\quad +L_M\Biggl [ \frac{512 \big (N^2+N+1\big )}{3 (N-1) N (N+1)^2 (N+2)^2} -\frac{256 S_1}{3 (N+1) (N+2)}\Biggr ] \nonumber \\&\quad +\frac{\frac{128}{3} S_1^2-\frac{128 S_2}{3}-\frac{256}{3} S_{-2}}{(N+1) (N+2)}\Biggr ] L_Q\nonumber \\&\quad +\frac{32 Q_{27}}{27 (N-1) N^3 (N+1)^4 (N+2)^2} -\frac{64 (56 N+47) S_1}{27 (N+1)^2 (N+2)}\nonumber \\&\quad +L_M^2 \Biggl [ \frac{256 \big (N^2+N+1\big )}{3 (N-1) N (N+1)^2 (N+2)^2} -\frac{128 S_1}{3 (N+1) (N+2)}\Biggr ]\nonumber \\&\quad +L_M \Biggl [\frac{256 (-1)^N \big (N^3+4 N^2+7 N+5\big )}{3 (N+1)^3 (N+2)^3}\nonumber \\&\quad +\frac{128 Q_{24}}{9 (N\!-\!1) N^2 (N\!+\!1)^3 (N\!+\!2)^3} +\frac{256 \big (N^3-6 N^2+2 N-3\big ) S_1}{9 (N\!-\!1) N (N\!+\!1)^2 (N\!+\!2)}\nonumber \\&\quad +\frac{\frac{128}{3} S_1^2-\frac{128 S_2}{3}-\frac{256}{3} S_{-2}}{(N+1) (N+2)}\Biggr ]\Biggr ] \nonumber \\&\quad +{C_A} {T_F^2} {N_F} \Biggl [ \Biggl [ \frac{256 \big (N^2+N+1\big )}{3 (N-1) N (N+1)^2 (N+2)^2}\nonumber \\&\quad -\frac{128 S_1}{3 (N\!+\!1) (N\!+\!2)}\Biggr ] L_Q^2\!+\!\Biggl [ \frac{256 (-1)^N \big (N^3\!+\!4 N^2\!+\!7 N\!+\!5\big )}{3 (N\!+\!1)^3 (N\!+\!2)^3}\nonumber \\&\quad +\frac{64 Q_{22}}{9 (N\!-\!1) N (N\!+\!1)^3 (N\!\!+2)^3}\!+\!\frac{256 \big (11 N^3\!-\!6 N^2\!-\!8 N\!-\!3\big ) S_1}{9 (N\!-\!1) N (N\!+\!1)^2 (N+2)}\nonumber \\&\quad +\frac{\frac{128}{3} S_1^2-\frac{128 S_2}{3}-\frac{256}{3} S_{-2}}{(N+1) (N+2)}\Biggr ] L_Q \Biggr ] +{C_A^2} {T_F}\Biggl [ \Biggl [\frac{128 S_1^2}{(N+1) (N+2)}\nonumber \\ \end{aligned}$$

$$\begin{aligned}&\quad +\frac{32 \big (11 N^4+22 N^3-59 N^2-70 N-48\big ) S_1}{3 (N-1) N (N+1)^2 (N+2)^2} \nonumber \\&\quad -\frac{64 \big (N^2+N+1\big ) \big (11 N^4+22 N^3-35 N^2-46 N-24\big )}{3 (N-1)^2 N^2 (N+1)^3 (N+2)^3}\Biggr ] L_Q^2 \nonumber \\&\quad +\Biggl [ -\frac{32 \big (59 N^4+70 N^3-155 N^2-118 N-72\big ) S_1^2}{3 (N-1) N (N+1)^2 (N+2)^2}\nonumber \\&\quad -\frac{256 (-1)^N \big (N^3+4 N^2+7 N+5\big ) S_1}{(N+1)^3 (N+2)^3} \nonumber \\&\quad -\frac{64 Q_{28} S_1}{9 (N-1)^2 N^2 (N+1)^3 (N+2)^3}\nonumber \\&\quad -\frac{64 (-1)^N Q_{25}}{3 (N-1) N^2 (N+1)^4 (N+2)^4}\nonumber \\&\quad -\frac{32 Q_{31}}{9 (N-1)^2 N^3 (N+1)^3 (N+2)^4}\nonumber \\&\quad +\frac{32 \big (11 N^4+22 N^3-83 N^2-94 N-72\big ) S_2}{3 (N-1) N (N+1)^2 (N+2)^2} \nonumber \\&\quad +\frac{64 \big (11 N^4+22 N^3-59 N^2-70 N-48\big ) S_{-2}}{3 (N-1) N (N+1)^2 (N+2)^2}\nonumber \\&\quad +\frac{1 }{(N+1) (N+2)} \Bigl [-128 S_1^3+384 S_2 S_1+512 S_{-2} S_1\nonumber \\&\quad +128 S_3 +128 S_{-3}-256 S_{-2,1}\Bigr ]\Biggr ] L_Q\Biggr ]\nonumber \\&\quad +{C_F^2} {T_F} \Biggl [ \Biggl [ \frac{8 \big (N^2+N+2\big ) \big (3 N^2+3 N+2\big )}{N^2 (N+1)^3 (N+2)}\nonumber \\ \end{aligned}$$

$$\begin{aligned}&\quad -\frac{32 \big (N^2+N+2\big ) S_1}{N (N+1)^2 (N+2)}\Biggr ] L_Q^2 \nonumber \\&\quad +\Biggl [\frac{128 (-1)^N \big (N^2+N+2\big ) Q_{34}}{5 (N-2) (N-1)^2 N^3 (N+1)^5 (N+2)^3 (N+3)^3}\nonumber \\&\quad -\frac{8 Q_{39}}{5 (N-1)^2 N^3 (N+1)^5 (N+2)^3 (N+3)^3}\nonumber \\&\quad -\frac{16 \big (9 N^4+26 N^3+49 N^2+48 N+12\big ) S_1}{N^2 (N+1)^3 (N+2)}\nonumber \\&\quad +L_M \Biggl [ \frac{64 \big (N^2+N+2\big ) S_1}{N (N+1)^2 (N+2)}\nonumber \\&\quad -\frac{16 \big (N^2+N+2\big ) \big (3 N^2+3 N+2\big )}{N^2 (N+1)^3 (N+2)}\Biggr ]\nonumber \\&\quad +\frac{256 \big (N^2+N+2\big ) \big (N^4+2 N^3-N^2-2 N-6\big ) S_{-2}}{(N-2) N^2 (N+1)^3 (N+2) (N+3)} \nonumber \\&\quad +\frac{\big (N^2+N+2\big ) }{N (N+1)^2 (N+2)}\Bigl [64 S_1^2-256 S_{-2} S_1\nonumber \\&\quad -64 S_2-128 S_3-128 S_{-3}+256 S_{-2,1}+384 \zeta _3\Bigr ]\Biggr ] L_Q \nonumber \\&\quad +\frac{16 (3 N+2) S_1^2}{N^2 (N+1) (N+2)} +\frac{8 Q_{26}}{N^4 (N+1)^5 (N+2)}\nonumber \\&\quad +\frac{16 \big (N^4-N^3-20 N^2-10 N-4\big ) S_1}{N^2 (N+1)^3 (N+2)}\nonumber \\&\quad +L_M^2 \Biggl [\frac{8 \big (N^2+N+2\big ) \big (3 N^2+3 N+2\big )}{N^2 (N+1)^3 (N+2)}\nonumber \\&\quad -\frac{32 \big (N^2+N+2\big ) S_1}{N (N+1)^2 (N+2)}\Biggr ]\nonumber \\ \end{aligned}$$

$$\begin{aligned}&\quad +\frac{16 \big (N^4+17 N^3+17 N^2-5 N-2\big ) S_2}{N^2 (N+1)^3 (N+2)}\nonumber \\&\quad +\frac{\big (N^2+N+2\big ) \Bigl [-\frac{16}{3} S_1^3-16 S_2 S_1+\frac{64 S_3}{3}\Bigr ]}{N (N+1)^2 (N+2)} \nonumber \\&\quad +\,L_M \Biggl [ -\frac{128 (-1)^N \big (N^2\!+\!N\!+\!2\big ) Q_{34}}{5 (N\!-\!2) (N\!-\!1)^2 N^3 (N\!+\!1)^5 (N\!+\!2)^3 (N+3)^3} \nonumber \\&\quad +\frac{8 Q_{39}}{5 (N-1)^2 N^3 (N+1)^5 (N+2)^3 (N+3)^3}\nonumber \\&\quad +\frac{16 \big (9 N^4+26 N^3+49 N^2+48 N+12\big ) S_1}{N^2 (N+1)^3 (N+2)}\nonumber \\&\quad -\frac{256 \big (N^2+N+2\big ) \big (N^4+2 N^3-N^2-2 N-6\big ) S_{-2}}{(N-2) N^2 (N+1)^3 (N+2) (N+3)}\nonumber \\&\quad +\frac{\big (N^2+N+2\big ) }{N (N+1)^2 (N+2)} \Bigl [-64 S_1^2+256 S_{-2} S_1+64 S_2\nonumber \\&\quad +128 S_3 +128 S_{-3}-256 S_{-2,1}-384 \zeta _3\Bigr ]\Biggr ]\Biggr ]\nonumber \\&\quad +{C_F} {T_F^2} \Biggl [ \frac{64 \big (N^2+N+2\big ) \big (N^4+2 N^3+2 N^2+N+6\big ) L_Q^2}{3 (N-1) N^2 (N+1)^3 (N+2)^2}\nonumber \\ \end{aligned}$$

$$\begin{aligned}&\quad +\Biggl [ -\frac{128 L_M \big (N^2+N+2\big )^2}{(N-1) N^2 (N+1)^3 (N+2)^2} \nonumber \\&\quad +\frac{256 (-1)^N Q_{30}}{45 (N-2) (N-1)^2 N^2 (N+1)^3 (N+2)^2 (N+3)^3}\nonumber \\&\quad -\frac{32 Q_{37}}{45 (N-1)^2 N^3 (N+1)^4 (N+2)^3 (N+3)^3} \nonumber \\&\quad -\frac{128 Q_{21} S_1}{3 (N\!-\!1) N^2 (N\!+\!1)^3 (N\!+\!2)^2} \!+\!\frac{256 (N\!-\!1) S_{-2}}{3 (N-2) (N\!+\!1) (N+3)}\Biggr ] L_Q\nonumber \\&\quad -\frac{64 (N-2) (N+3) \big (N^2+N+1\big ) \big (N^2+N+2\big ) L_M^2}{3 (N-1) N^2 (N+1)^3 (N+2)^2}\nonumber \\&\quad -\frac{16 Q_{32}}{(N-1) N^4 (N+1)^5 (N+2)^2}\nonumber \\&\quad +L_M \Biggl [ \frac{256 (-1)^N Q_{30}}{45 (N-2) (N-1)^2 N^2 (N+1)^3 (N+2)^2 (N+3)^3}\nonumber \\&\quad +\frac{256 (N-1) S_{-2}}{3 (N-2) (N+1) (N+3)}\nonumber \\&\quad +\frac{32 Q_{38}}{45 (N-1)^2 N^3 (N+1)^4 (N+2)^3 (N+3)^3}\nonumber \\&\quad -\frac{128 Q_{19} S_1}{3 (N-1) N^2 (N+1)^3 (N+2)^2} \Biggr ]\Biggr ]\nonumber \\&\quad +{C_F} {T_F^2} {N_F} \Biggl [ \frac{64 \big (N^2+N+2\big ) \big (N^4+2 N^3+2 N^2+N+6\big ) L_Q^2}{3 (N-1) N^2 (N+1)^3 (N+2)^2} \nonumber \\ \end{aligned}$$

$$\begin{aligned}&\quad +\Biggl [ \frac{256 (-1)^N Q_{30}}{45 (N-2) (N-1)^2 N^2 (N+1)^3 (N+2)^2 (N+3)^3}\nonumber \\&\quad -\frac{128 Q_{21} S_1}{3 (N-1) N^2 (N+1)^3 (N+2)^2}\nonumber \\&\quad -\frac{32 Q_{37}}{45 (N-1)^2 N^3 (N+1)^4 (N+2)^3 (N+3)^3}\nonumber \\&\quad +\frac{256 (N-1) S_{-2}}{3 (N-2) (N+1) (N+3)} -\frac{64 \big (N^2+N+2\big ) L_M}{3 N (N+1)^2 (N+2)}\Biggr ] L_Q \nonumber \\&\quad +L_M \Biggl [ \frac{32 \big (N^2+N+2\big ) \big (19 N^2+7 N-6\big )}{9 N^2 (N+1)^3 (N+2)}\nonumber \\&\quad +\frac{64 \big (N^2+N+2\big ) S_1}{3 N (N+1)^2 (N+2)}\Biggr ]\Biggr ]+{C_F} {C_A} {T_F} \nonumber \\&\quad \times \Biggl [ \Biggl [ -\frac{16 \big (N^2\!+\!N\!+\!2\big ) \big (11 N^4\!+\!22 N^3\!-\!23 N^2\!-\!34 N\!-\!12\big )}{3 (N\!-\!1) N^2 (N\!+\!1)^3(N\!+\!2)^2}\nonumber \\&\quad -\frac{32 \big (N^2+N+2\big ) S_1}{N (N+1)^2 (N+2)}\Biggr ] L_Q^2+\Biggl [ \frac{32 \big (5 N^2+5 N+2\big ) S_1^2}{N (N+1)^2 (N+2)}\nonumber \\&\quad -\frac{256 (-1)^N Q_{30} S_1}{15 (N-2) (N-1)^2 N^2 (N+1)^3 (N+2)^2 (N+3)^3} \nonumber \\&\quad +\frac{128 Q_{33} S_1}{15 (N-1)^2 N^2 (N+1)^3 (N+2)^2 (N+3)^3}\nonumber \\&\quad -\frac{128 \big (N^4+2 N^3+N^2+12\big ) S_{-2} S_1}{(N-2) N (N+1)^2 (N+2) (N+3)} \nonumber \\&\quad -\frac{64 (-1)^N Q_{41}}{45 (N-2) (N-1)^3 N^3 (N+1)^5 (N+2)^3 (N+3)^3}\nonumber \\ \end{aligned}$$

$$\begin{aligned}&\quad +\frac{8 Q_{42}}{45 (N-1)^3 N^3 (N+1)^5 (N+2)^3 (N+3)^3}\nonumber \\&\quad -\frac{128 Q_{23} S_{-2}}{3 (N-2) N^2 (N+1)^3 (N+2) (N+3)}+\frac{176 \big (N^2+N+2\big ) L_M}{3 N (N+1)^2 (N+2)}\nonumber \\&\quad +\frac{\big (N^2+N+2\big ) \Bigl [-32 S_2+64 S_3+64 S_{-3}-128 S_{-2,1}-192 \zeta _3\Bigr ]}{N (N+1)^2 (N+2)}\Biggr ] L_Q \nonumber \\&\quad -\frac{16 \big (N^3+8 N^2+11 N+2\big ) S_1^2}{N (N+1)^3 (N+2)^2}\nonumber \\&\quad +\frac{16 Q_{35}}{(N-1) N^4 (N+1)^5 (N+2)^4} -\frac{16 Q_{20} S_1}{N (N+1)^4 (N+2)^3}\nonumber \\&\quad +L_M^2 \Biggl [ \frac{32 \big (N^2+N+2\big ) S_1}{N (N+1)^2 (N+2)}\nonumber \\&\quad -\frac{64 \big (N^2+N+1\big ) \big (N^2+N+2\big )}{(N-1) N^2 (N+1)^3 (N+2)^2}\Biggr ] \nonumber \\&\quad -\frac{16 \big (7 N^5+21 N^4+13 N^3+21 N^2+18 N+16\big ) S_2}{(N-1) N^2 (N+1)^3 (N+2)^2}\nonumber \\&\quad +\frac{\big (N^2-N-4\big ) 64 (-1)^N S_{-2} }{(N+1)^3 (N+2)^2} \nonumber \\&\quad +\frac{\big (N^2+N+2\big ) }{N (N+1)^2 (N+2)} \Bigl [\frac{16}{3} S_1^3 +48 S_2 S_1+64 (-1)^N S_{-2} S_1\nonumber \\ \end{aligned}$$

275 $$\begin{aligned}&\quad +\frac{128 S_3}{3}+32 (-1)^N S_{-3}-64 S_{-2,1} \Bigr ]\nonumber \\&\quad +L_M \Biggl [ \frac{64 (-1)^N Q_{36}}{5 (N-2) (N-1)^2 N^3 (N+1)^5 (N+2)^3 (N+3)^3} \nonumber \\&\quad -\frac{8 Q_{40}}{45 (N-1)^2 N^3 (N+1)^5 (N+2)^3 (N+3)^3}\nonumber \\&\quad -\frac{16 \big (23 N^4+92 N^3+209 N^2+256 N+92\big ) S_1}{3 N (N+1)^3 (N+2)^2} \nonumber \\&\quad +\frac{64 \big (N^2+N+2\big ) \big (3 N^4+6 N^3-7 N^2-10 N-12\big ) S_{-2}}{(N-2) N^2 (N+1)^3 (N+2) (N+3)} \nonumber \\&\,\,+\frac{\big (N^2{+}N{+}2\big ) \Bigl [32 S_1^2{-}128 S_{-2} S_1{+}32 S_2 {-}64 S_3{-}64 S_{-3}{+}128 S_{-2,1}{+}192 \zeta _3\Bigr ]}{N (N{+}1)^2 (N{+}2)}\Biggr ]\Biggr ] \nonumber \\&\quad +\tilde{C}_{L,g}^{\mathsf{S},(3)}(N_F+1) \Biggr \} \Biggr \}, \end{aligned}$$

with

276 $$\begin{aligned} Q_{19}&= N^6+3 N^5-2 N^4-9 N^3-17 N^2-12 N-12 \end{aligned}$$

277 $$\begin{aligned} Q_{20}&= N^6+8 N^5+23 N^4+54 N^3+94 N^2+72 N+8 \end{aligned}$$

278 $$\begin{aligned} Q_{21}&= 2 N^6+6 N^5+7 N^4+4 N^3+9 N^2+8 N+12 \end{aligned}$$

279 $$\begin{aligned} Q_{22}&= 3 N^6+3 N^5-121 N^4-391 N^3-474 N^2-308 N-80 \end{aligned}$$

280 $$\begin{aligned} Q_{23}&= 10 N^6+30 N^5+N^4-48 N^3-89 N^2-60 N-36\end{aligned}$$

281 $$\begin{aligned} Q_{24}&= 6 N^7+24 N^6+47 N^5+104 N^4\nonumber \\&+219 N^3+316 N^2+208 N+48 \end{aligned}$$

282 $$\begin{aligned} Q_{25}&= 11 N^8+66 N^7+106 N^6-121 N^5-775 N^4\nonumber \\&-1325 N^3-1130 N^2-552 N-96 \end{aligned}$$

283 $$\begin{aligned} Q_{26}&= 12 N^8+52 N^7+132 N^6+216 N^5\nonumber \\&+191 N^4+54 N^3-25 N^2-20 N-4 \end{aligned}$$

284 $$\begin{aligned} Q_{27}&= 15 N^8+60 N^7+572 N^6+1470 N^5+2135 N^4\nonumber \\&+1794 N^3+722 N^2-24 N-72 \end{aligned}$$

285 $$\begin{aligned} Q_{28}&= 133 N^8+430 N^7-271 N^6-1361 N^5\nonumber \\&+110 N^4+2023 N^3+1684 N^2-12 N-144\end{aligned}$$

286 $$\begin{aligned} Q_{29}&= 26 N^9+539 N^8+3244 N^7+8465 N^6+9342 N^5\nonumber \\&+841 N^4-5720 N^3-2193 N^2 \nonumber \\&+2484 N+1404 \end{aligned}$$

287 $$\begin{aligned} Q_{30}&= N^{10}-13 N^9-39 N^8+222 N^7+1132 N^6\nonumber \\&+1787 N^5+913 N^4+392 N^3 \nonumber \\&+645 N^2-324 N-108 \end{aligned}$$

288 $$\begin{aligned} Q_{31}&= 3 N^{10}-48 N^9-856 N^8-2702 N^7-1961 N^6\nonumber \\&+2142 N^5+3122 N^4-1924 N^3 \nonumber \\&-5552 N^2-4032 N-1152 \end{aligned}$$

289 $$\begin{aligned} Q_{32}&= 15 N^{10}+75 N^9+112 N^8+14 N^7-61 N^6\nonumber \\&+107 N^5+170 N^4+36 N^3 \nonumber \\&-36 N^2-32 N-16 \end{aligned}$$

290 $$\begin{aligned} Q_{33}&= 35 N^{10}+372 N^9+1263 N^8+673 N^7-5090 N^6\nonumber \\&-11596 N^5-8413 N^4+2305 N^3 \nonumber \\&+8049 N^2 +3078 N+108\end{aligned}$$

291 $$\begin{aligned} Q_{34}&= 2 N^{11}+41 N^{10}+226 N^9+556 N^8\nonumber \\&+963 N^7+2733 N^6+7160 N^5+8610 N^4+1969 N^3 \nonumber \\&-2748 N^2-864 N-216 \end{aligned}$$

292 $$\begin{aligned} Q_{35}&= 2 N^{12}+20 N^{11}+86 N^{10}+192 N^9+199 N^8-N^7\nonumber \\&-297 N^6-495 N^5-514 N^4-488 N^3 \nonumber \\&-416 N^2-176 N-32 \end{aligned}$$

293 $$\begin{aligned} Q_{36}&= 12 N^{13}+143 N^{12}+591 N^{11}+954 N^{10}+371 N^9\nonumber \\&+1658 N^8+11559 N^7+26626 N^6 \nonumber \\&+29129 N^5 +14011 N^4-2374 N^3-6576 N^2-1944 N-432 \nonumber \\\end{aligned}$$

294 $$\begin{aligned} Q_{37}&= 95 N^{13}+1218 N^{12}+6096 N^{11}+14484 N^{10}\nonumber \\&+11570 N^9-28440 N^8-117844 N^7 \nonumber \\&-225884 N^6 -238953 N^5-83290 N^4\nonumber \\&+57660 N^3+122040 N^2+182304 N +77760 \end{aligned}$$

295 $$\begin{aligned} Q_{38}&= 185 N^{13}+2582 N^{12}+15584 N^{11}+53036 N^{10}\nonumber \\&+109190 N^9+124040 N^8+12604 N^7 \nonumber \\&-200836 N^6 -294247 N^5-116270 N^4+85260 N^3\nonumber \\&+158760 N^2+193536 N +77760\end{aligned}$$

296 $$\begin{aligned} Q_{39}&= 35 N^{14}+465 N^{13}+1962 N^{12}-348 N^{11}\nonumber \\&-32130 N^{10}-131686 N^9-280396 N^8 \nonumber \\&-363984 N^7 -290209 N^6-122547 N^5\nonumber \\&+6730 N^4+47316 N^3+11928 N^2 \nonumber \\&-21600 N-5184\end{aligned}$$

297 $$\begin{aligned} Q_{40}&= 1255 N^{14}+18165 N^{13}+107824 N^{12}+331744 N^{11}\nonumber \\&+515430 N^{10}+132498 N^9 \nonumber \\&-1057432 N^8 -2202648 N^7-1979173 N^6-534079 N^5\nonumber \\&+350880 N^4-29088 N^3 \nonumber \\&-519264 N^2-382320 N -62208\end{aligned}$$

298 $$\begin{aligned} Q_{41}&= 11 N^{15}-2 N^{14}+308 N^{13}+5275 N^{12}+24535 N^{11}\nonumber \\&+52925 N^{10}+50941 N^9-5977 N^8 \nonumber \\&-85550 N^7-191059 N^6-294877 N^5-248414 N^4\nonumber \\&-64728 N^3+57636 N^2+28944 N +6480\end{aligned}$$

299 $$\begin{aligned} Q_{42}&= 1255 N^{15}+16338 N^{14}+76085 N^{13}+117654 N^{12}\nonumber \\&-198422 N^{11}-971844 N^{10} \nonumber \\&-1002678 N^9+1019372 N^8+3525323 N^7\nonumber \\&+3236906 N^6+272625 N^5-1523746 N^4 \nonumber \\&-632844 N^3+606888 N^2+635904 N+129600. \end{aligned}$$

As has been outlined for the 2-loop results in Ref. [37] already, the scales at which the asymptotic expressions are dominating are estimated to be $Q^2/m^2 \gtrsim 800$. They are far outside the kinematic region in which the structure function $F_L(x,Q^2)$ can presently be measured in deep-inelastic scattering. At LHeC [105], the region of large values of $Q^2$ can be probed and the corresponding expressions may be used in phenomenological analyses there.

## Comparison of Mellin moments for the Wilson coefficients and OMEs

To compare the relative impact of the different Wilson coefficients on the structure function $F_2(x,Q^2)$, we will consider the Mellin moments for $N = 2$ to 10 in the following, convolved with the moments of the respective parton distribution functions in the flavor singlet case, i.e., the gluon $G(x,Q^2)$ and quark-singlet density $\Sigma (x,Q^2)$ for $N_F = 3$ and characteristic values of $Q^2$. Since only a series of Mellin moments has been calculated at large momentum transfer $Q^2$ in Ref. [39], a detailed numerical comparison is only possible in this way at the moment. The numerical results for the moments of the contributing parton densities are given in Table 2. Note that for $N \ge 2$, the moments for the singlet distribution are mostly larger than those of the gluon. We apply these parton densities to study the relative contributions of the different Wilson coefficients, normalizing to $H_{g,2}^{\mathsf{S}}$ within the respective order in $a_s$ using the following ratios:

$$\begin{aligned} R\left( L_{g,2}^{\mathsf{S}},H_{g,2}^{\mathsf{S}}\right)&= \frac{c_{N_F} \, L_{g,2}^{\mathsf{S}}\, G }{c_Q \, H_{g,2}^{\mathsf{S}}\, G} \\ R\left( L_{q,2}^{\mathsf{PS}},H_{g,2}^{\mathsf{S}}\right)&= \frac{c_{N_F} \, L_{q,2}^{\mathsf{PS}}\, \Sigma }{c_Q \, H_{g,2}^{\mathsf{S}}\, G} \\ R\left( H_{q,2}^{\mathsf{PS}},H_{g,2}^{\mathsf{S}}\right)&= \frac{c_Q \, H_{q,2}^{\mathsf{PS}}\, \Sigma }{c_Q \, H_{g,2}^{\mathsf{S}}\, G}, \end{aligned}$$

where

300 $$\begin{aligned} c_{N_F} = \frac{1}{N_F} \sum \limits _{k=1}^{N_F} e_k^2,\quad c_Q = e_Q^2. \end{aligned}$$

In the numerical examples, we set $e_Q = e_c = 2/3$ and use $m_c = 1.59~\mathrm{GeV}$.

**Table 2:** The moments $N =2,\ldots , 10$ of the gluon and quark-singlet momentum density using the parton distribution functions [21]

$Q^2$	$20\,\text {GeV}^2$				
$N$	$2$	$4$	$6$	$8$	$10$
$G$	$0.4583$	$0.0044$	$0.0003$	$3.62\times 10^{-5}$	$7.78\times 10^{-6}$
$\Sigma$	$0.5417$	$0.0353$	$0.0070$	$2.10\times 10^{-3}$	$8.01\times 10^{-4}$

$Q^2$	$100\,\text {GeV}^2$				
$N$	$2$	$4$	$6$	$8$	$10$
$G$	$0.4819$	$0.0038$	$0.0002$	$3.60\times 10^{-5}$	$8.55\times 10^{-6}$
$\Sigma$	$0.5181$	$0.0296$	$0.0056$	$1.61\times 10^{-3}$	$5.97\times 10^{-4}$

$Q^2$	$1000\,\text {GeV}^2$				
$N$	$2$	$4$	$6$	$8$	$10$
$G$	$0.5042$	$0.0032$	$0.0002$	$3.14\times 10^{-5}$	$7.60\times 10^{-6}$
$\Sigma$	$0.4958$	$0.0244$	$0.0043$	$1.20\times 10^{-3}$	$4.32\times 10^{-3}$

Before we discuss quantitative results, a remark on the contributions by the color factor $d_{abc} d_{abc}$ to the massless Wilson coefficients contributing to Eq. (2) is in order. Here we refer to Refs. [106–108] and [38, 109]. For $SU(n)$, one obtains

301 $$\begin{aligned} d_{abc} d_{abc} = \frac{(n^2-1)(n^2-4)}{n}. \end{aligned}$$

It emerges weighted by $1/N_c$ and $1/N_A$ for external quark and gluon lines, respectively, with $N_c = n$ and $N_A = n^2 -1$. In Refs. [106–108], this group-theoretic expression has been used, while in [38, 109], a factor of $16$ has been taken out and was absorbed into the Lorentz structure of the corresponding contribution to the Wilson coefficient. We agree with the $N_F$-dependence as given in Refs. [106–108]. Furthermore, we note a typographical error in Eq. (4.13) of [38]. Here, the corresponding term reads correctly^13^

302 $$\begin{aligned} c_{2,g}^{(3)}(z) \simeq - 932.089 N_F \frac{L_0}{z} ...,\quad \text {with}\ L_0 = \ln (z). \end{aligned}$$

Also in the pure-singlet case, the massless Wilson coefficients contain terms $\propto d_{abc} d_{abc}$, although with a generally different charge-weight factor, cf. [106–108].

Let us now consider the relative impact of the individual massive Wilson coefficients. The ratios at $O(a_s^2)$ and $O(a_s^3)$ for different values of $Q^2$ and the moments $N=2$ to 10 are given in Table 3. One first notes that at low values of $Q^2$ the ratio $R(L_{g,2}^{\mathsf{S}},H_{g,2}^{\mathsf{S}})$ changes sign, which is also the case for $R(H_{q,2}^{\mathsf{PS}},H_{g,2}^{\mathsf{S}})$ in the whole region up to $Q^2 = 1000~\mathrm{GeV}^2$. At $O(a_s^2)$ $L_{g,2}^{\mathsf{S}}$ is small for low moments and grows to $24~\%$ for $N=10$ compared to $H_{g,2}^{\mathsf{S}}$ at $Q^2 = 100~\mathrm{GeV}^2$, with lower values at higher $Q^2$. A comparable tendency is observed at $O(a_s^3)$. The fraction $|R(H_{q,2}^{\mathsf{PS}},H_{g,2}^{\mathsf{S}})|$ moves between $25~\%$ and $170~\%$ comparing the moments $N=2$ to $10$ at $Q^2 = 20~\mathrm{GeV}^2$ and upper values of $\sim 70~\%$ at $Q^2 = 1000~\mathrm{GeV}^2$.

**Table 3:** Relative impact of the moments $N = 2,\ldots , 10$ of the individual massive Wilson coefficients, weighted by moments of the corresponding parton distributions [21], at $O(a_s^2)$ and $O(a_s^3)$ normalized to the contribution to $H_{g,2}^{\mathsf{S}}$ for $Q^2 = 20, 100$ and $1000~\mathrm{GeV}^2$

$Q^2$	$20\,\text {GeV}^2$				
$N$	$2$	$4$	$6$	$8$	$10$
$$\Sigma /G$$	$$1.1821$$	$$ 7.9967$$	$$25.847$$	$$ 57.965$$	$$ 103.06$$
$O(a_s^2)$:					
$$R(L_{g,2}^{\mathsf{S}},H_{g,2}^{\mathsf{S}})$$	$$ 0.0387$$	$$ 0.1349$$	$$1.7000$$	$$-0.2592$$	$$-0.1384$$
$R(H_{q,2}^{\mathsf{PS}},H_{g,2}^{\mathsf{S}})$	$-0.2588$	$-0.3018$	$1.9946$	$-1.6153$	$-1.7222$
$O(a_s^3)$:					
$$R(L_{g,2}^{\mathsf{S}},H_{g,2}^{\mathsf{S}})$$	$$ 0.0829$$	$$ 0.1983$$	$$0.6628$$	$$-2.5018$$	$$-0.5957$$
$$R(L_{q,2}^{\mathsf{PS}},H_{g,2}^{\mathsf{S}})$$	$$ 0.0438$$	$$ 0.1042$$	$$0.7483$$	$$-5.7476$$	$$-2.3762$$
$R(H_{q,2}^{\mathsf{PS}},H_{g,2}^{\mathsf{S}})$	$-0.2259$	$-0.3472$	$0.3387$	$-9.3371$	$-4.5870$

$Q^2$	$100\,\text {GeV}^2$				
$$\Sigma /G$$	$$ 1.0753$$	$$7.7514$$	$$ 22.797$$	$$ 44.660$$	$$ 69.888$$
$O(a_s^2)$:					
$$R(L_{g,2}^{\mathsf{S}},H_{g,2}^{\mathsf{S}})$$	$$ 0.0313$$	$$ 0.0687$$	$$ 0.1071$$	$$ 0.1587$$	$$ 0.2418$$
$R(H_{q,2}^{\mathsf{PS}},H_{g,2}^{\mathsf{S}})$	$-0.2496$	$-0.3753$	$-0.5429$	$-0.6531$	$-0.6743$
$O(a_s^3)$:					
$$R(L_{g,2}^{\mathsf{S}},H_{g,2}^{\mathsf{S}})$$	$$ 0.0533$$	$$ 0.0853$$	$$ 0.1449$$	$$ 0.2186$$	$$ 0.3195$$
$$R(L_{q,2}^{\mathsf{PS}},H_{g,2}^{\mathsf{S}})$$	$$ 0.0340$$	$$ 0.0378$$	$$ 0.1006$$	$$ 0.2600$$	$$ 0.5828$$
$R(H_{q,2}^{\mathsf{PS}},H_{g,2}^{\mathsf{S}})$	$-0.3062$	$-0.5165$	$-0.7070$	$-0.7471$	$-0.5637$

$Q^2$	$1000\,\text {GeV}^2$				
$$\Sigma /G$$	$$ 0.9833$$	$$ 7.5948$$	$$ 20.958$$	$$ 38.236$$	$$ 56.876$$
$O(a_s^2)$:					
$$R(L_{g,2}^{\mathsf{S}},H_{g,2}^{\mathsf{S}})$$	$$ 0.0243$$	$$ 0.0420$$	$$ 0.0531$$	$$ 0.0615$$	$$ 0.0687$$
$R(H_{q,2}^{\mathsf{PS}},H_{g,2}^{\mathsf{S}})$	$-0.2837$	$-0.4085$	$-0.5690$	$-0.6707$	$-0.7237$
$O(a_s^3)$:					
$$R(L_{g,2}^{\mathsf{S}},H_{g,2}^{\mathsf{S}})$$	$$ 0.0337$$	$$ 0.0392$$	$$ 0.0597$$	$$ 0.0764$$	$$ 0.0907$$
$$R(L_{q,2}^{\mathsf{PS}},H_{g,2}^{\mathsf{S}})$$	$$ 0.0313$$	$$ 0.0209$$	$$ 0.0297$$	$$ 0.0505$$	$$ 0.0828$$
$R(H_{q,2}^{\mathsf{PS}},H_{g,2}^{\mathsf{S}})$	$-0.3825$	$-0.5903$	$-0.8058$	$-0.9253$	$-0.9679$

In the case of the comparison of the massive OMEs, we normalize to $A_{Qg}$ with PDFs according to their appearance in the singlet and gluon transitions from $N_F \rightarrow N_F + 1$ massless flavors in the variable flavor number scheme, cf. Eqs. (13–15):

$$\begin{aligned} R(A_{gg,Q},A_{Qg})&= \frac{A_{gg,Q}\, G }{A_{Qg}\, G}, \quad R\left( A_{Qq}^{\mathsf{PS}},A_{Qg}\right) = \frac{A_{Qq}^{\mathsf{PS}}\, \Sigma }{A_{Qg}\, G} \\ R\left( A_{qq,Q}^\mathsf{NS},A_{Qg}\right)&= \frac{A_{qq,Q}^\mathsf{NS}\, \Sigma }{A_{Qg}\, G}, \quad R(A_{gq,Q},A_{Qg}) = \frac{A_{gq,Q}\, \Sigma }{A_{Qg}\, G} \\ R(A_{qg,Q},A_{Qg})&= \frac{A_{qg,Q}\, G }{A_{Qg}\, G}, \quad R\left( A_{qq,Q}^{\mathsf{PS}},A_{Qg}\right) = \frac{A_{qq,Q}^{\mathsf{PS}}\, \Sigma }{A_{Qg}\, G}. \end{aligned}$$

Here, we have included also the moments of the OME $A_{qq,Q}^\mathsf{NS}$ since it contributes to the singlet term in the VFNS. These ratios describe the relative impact, within the corresponding order in $a_s$, of the massive OMEs in the variable flavor number scheme for the flavor singlet contributions.

**Table 4:** Relative impact of the moments $N = 2,\ldots , 10$ of the individual massive OMEs, weighted by moments of the corresponding parton distributions [21], at the different orders in $a_s$ normalized to the contribution to $A_{Qg}$ for $Q^2 = 20$ and 100 GeV$^2$

$Q^2$	$20\,\text {GeV}^2$				
$N$	$2$	$4$	$6$	$8$	$10$
$\Sigma /G$	$1.1821$	$7.9967$	$25.847$	$57.965$	$103.06$
$O(a_s)$:					
$R(A_{gg,Q},A_{Qg})$	$-1.0000$	$-1.8182$	$-2.5455$	$-3.2432$	$-3.9286$
$$O(a_s^2)$$					
$R(A_{gg,Q},A_{Qg})$	$-1.0000$	$-1.6395$	$-2.3808$	$-3.1781$	$-4.0262$
$R(A_{Qq}^{\mathsf{PS}},A_{Qg})$	$-0.1259$	$-0.3656$	$-0.7822$	$-1.3339$	$-1.9352$
$R(A_{qq,Q}^\mathsf{NS},A_{Qg})$	$-0.0584$	$-1.1306$	$-6.3206$	$-20.735$	$-49.508$
$$R(A_{gq,Q},A_{Qg})$$	$$0.1843$$	$$1.1422$$	$$ 3.9956$$	$$ 9.8073$$	$$ 18.995$$
$O(a_s^3)$:					
$R(A_{gg,Q},A_{Qg})$	$-1.0051$	$-1.3397$	$-1.8466$	$-2.4306$	$-3.0890$
$R(A_{Qq}^{\mathsf{PS}},A_{Qg})$	$-0.1604$	$-0.4838$	$-0.9635$	$-1.5449$	$-2.1295$
$R(A_{qq,Q}^\mathsf{NS},A_{Qg})$	$-0.0404$	$-0.5832$	$-2.9406$	$-9.1817$	$-21.375$
$$R(A_{gq,Q},A_{Qg})$$	$$ 0.1473$$	$$ 1.1265$$	$$ 3.7972$$	$$ 8.9925$$	$$ 16.961$$
$$R(A_{qg,Q},A_{Qg})$$	$$ 0.0051$$	$$-0.0202$$	$$-0.0326$$	$$-0.0445$$	$$-0.0567$$
$$R(A_{qq,Q}^{\mathsf{PS}},A_{Qg})$$	$$ 0.0534$$	$$ 0.1093$$	$$ 0.2460$$	$$ 0.4678$$	$$ 0.7619$$

$Q^2$	$100\,\text {GeV}^2$				
$\Sigma /G$	$1.0753$	$7.7514$	$22.797$	$44.660$	$69.888$
$O(a_s)$:					
$R(A_{gg,Q},A_{Qg})$	$-1.0000$	$-1.8182$	$-2.5455$	$-3.2432$	$-3.9286$
$O(a_s^2)$:					
$R(A_{gg,Q},A_{Qg})$	$-1.0000$	$-1.7746$	$-2.6448$	$-3.5805$	$-4.5771$
$R(A_{Qq}^{\mathsf{PS}},A_{Qg})$	$-0.1884$	$-0.4246$	$-0.7627$	$-1.0887$	$-1.3514$
$R(A_{qq,Q}^\mathsf{NS},A_{Qg})$	$-0.1247$	$-2.4385$	$-12.377$	$-35.460$	$-74.510$
$$R(A_{gq,Q},A_{Qg})$$	$$ 0.3131$$	$$ 1.3950$$	$$ 3.9075$$	$$ 7.7692$$	$$ 12.560$$
$O(a_s^3)$:					
$R(A_{gg,Q},A_{Qg})$	$-1.0048$	$-1.6120$	$-2.3201$	$-3.0734$	$-3.8667$
$R(A_{Qq}^{\mathsf{PS}},A_{Qg})$	$-0.2799$	$-0.5808$	$-0.9473$	$-1.2540$	$-1.4615$
$R(A_{qq,Q}^\mathsf{NS},A_{Qg})$	$-0.1772$	$-2.8698$	$-13.694$	$-37.928$	$-77.874$
$$R(A_{gq,Q},A_{Qg})$$	$$ 0.3924$$	$$ 1.4140$$	$$ 3.4078$$	$$6.0520$$	$$ 8.9253$$
$$R(A_{qg,Q},A_{Qg})$$	$$ 0.0048$$	$$-0.0375$$	$$-0.0491$$	$$-0.0580$$	$$-0.0657$$
$$R(A_{qq,Q}^{\mathsf{PS}},A_{Qg})$$	$$ 0.0647$$	$$ 0.0984$$	$$ 0.1726$$	$$ 0.2553$$	$$ 0.3319$$

The numerical values for different scales of $Q^2$ are given in Tables 4 and 5. $|A_{gg,Q}/A_{Qg}|$ rises from about 1 to higher values from $N = 2$ to 10, irrespective of the values of $Q^2$ and the order in $a_s$. The smallest contributions are $|A_{qg,Q}|$ and $A_{qq,Q}^{\mathsf{PS}}$ contributing the ratios $R$ by $\sim$0.5–5 % and form $\sim$5–$\sim$10 %, respectively, for $N = 2$ and 4, i.e., in the region dominated by lower values of the Bjorken variable $x$. The OMEs $|A_{Q,q}^{\mathsf{PS}}|$ and $A_{gq,Q}$ have contributions of 16–62 and 14–150 %, respectively, for $N = 2$ and $4$. Also the flavor non-singlet Wilson coefficient $|A_{qq,Q}^\mathsf{NS}|$ [43] contributes in the flavor singlet transitions and is weighted by the distribution $\Sigma$ here. Its relative impact rises with $Q^2$ and amounts from $\sim$4 to 370 % for the $R$-ratio considering the lower moments $N = 2$ and $N=4$ only.

**Table 5:** The same as Table 4 for $Q^2 = 1000~\mathrm{GeV}^2$

$Q^2$	$1000\,\text {GeV}^2$				
$N$	$2$	$4$	$6$	$8$	$10$
$\Sigma /G$	$0.9833$	$7.5948$	$20.958$	$38.236$	$56.876$
$O(a_s)$:					
$R(A_{gg,Q},A_{Qg})$	$-1.0000$	$-1.8182$	$-2.5455$	$-3.2432$	$-3.9286$
$O(a_s^2)$:					
$R(A_{gg,Q},A_{Qg})$	$-1.0000$	$-2.0101$	$-3.0170$	$-4.0596$	$-5.1403$
$R(A_{Qq}^{\mathsf{PS}},A_{Qg})$	$-0.2555$	$-0.4521$	$-0.6997$	$-0.8850$	$-1.0072$
$R(A_{qq,Q}^\mathsf{NS},A_{Qg})$	$-0.2048$	$-3.7111$	$-16.978$	$-44.037$	$-85.945$
$$R(A_{gq,Q},A_{Qg})$$	$$ 0.4603$$	$$ 1.5427$$	$$ 3.5816$$	$$ 6.1302$$	$$ 8.8857$$
$O(a_s^3)$:					
$R(A_{gg,Q},A_{Qg})$	$-1.0054$	$-1.7515$	$-2.4980$	$-3.2560$	$-4.0291$
$R(A_{Qq}^{\mathsf{PS}},A_{Qg})$	$-0.3731$	$-0.6275$	$-0.9067$	$-1.0906$	$-1.1928$
$R(A_{qq,Q}^\mathsf{NS},A_{Qg})$	$-0.2930$	$-4.1599$	$-17.532$	$-43.424$	$-82.110$
$$R(A_{gq,Q},A_{Qg})$$	$$ 0.5934$$	$$ 1.5060$$	$$ 2.9692$$	$$ 4.5100$$	$$ 5.9397$$
$$R(A_{qg,Q},A_{Qg})$$	$$ 0.0054$$	$$-0.0469$$	$$-0.0561$$	$$-0.0624$$	$$-0.0675$$
$$R(A_{qq,Q}^{\mathsf{PS}},A_{Qg})$$	$$ 0.0727$$	$$ 0.0902$$	$$ 0.1341$$	$$ 0.1721$$	$$ 0.2011$$

Right after having obtained a series of moments for the massive OMEs at 3-loops in [39], it became clear that the logarithmic contributions are of comparable order to the constant term. Moreover, there is a strong functional dependence w.r.t. $N$, as displayed in Tables 2, 3, 4 and 5. To obtain a definite answer, the calculation of the constant parts of the un-renormalized OMEs $a_{ij}^{(3),0}$ as a function of $N \in \mathbb {C}$ is necessary. In particular, predictions for the range of small values $x \simeq 10^{-4}$ appear to be rather difficult using constraints by a low number of moments only.

## Conclusions

We have derived the contributions of the massive Wilson coefficients to the structure functions $F_2(x,Q^2)$ and $F_L(x,Q^2)$ in deep-inelastic scattering and the corresponding massive OMEs to 3-loop order in the asymptotic region $Q^2 \gg m^2$ in Mellin $N$-space, except for the constant parts $a_{ij}^{(3),0}$ of the un-renormalized OMEs, which are not known for all quantities yet. Here, we retained both the scale dependence due to the virtuality $Q^2$ and the factorization and renormalization scales $\mu ^2$, which were set equal. This allows for scale variation studies in applications. Two of the Wilson coefficients, $L_{q,2}^{\mathsf{PS}}$ and $L_{g,2}^{\mathsf{S}}$, are known in complete form, and the corresponding results for $L_{q,2}^\mathsf{NS}$ are given in [43]. In the variable flavor number scheme being applied to describe the process through which an initially massive quark transmutes into a massless one at high momentum scales, moreover, the matching coefficients $A_{ij}$ are needed. Here, $A_{qq,Q}^{\mathsf{PS}}$ and $A_{qg,Q}$ are known in complete form to 3-loop order and the results for $A_{gq}$ and $A_{qq,Q}^\mathsf{NS}$ are given in [42] and [43], respectively.

We have given numerical results for the Wilson coefficients $L_{q,2}^{\mathsf{PS}}$ and $L_{g,2}^{\mathsf{S}}$. Using the available Mellin moments we have performed a numerical comparison of the different Wilson coefficients and operator matrix elements within the respective order in the coupling constant for the moments $N$ = 2–10 and in the $Q^2$ range between 20 and 1000 GeV$^2$. Here we have included also the OME from [42] and the Wilson coefficient and OME in the pure-singlet case for completeness. For numerical results in the non-singlet case see [43]. While some of the quantities studied are of minor importance, several others for the Wilson coefficients and OMEs are of similar size, which is varying in the kinematic range of experimental interest for present and future precision measurements. Even in case of the charm-quark contributions, the logarithmic terms are not dominant over the constant contributions in wide kinematic ranges, as, e.g., at HERA. Moreover, corrections of $O$ (0.5–1 %) are important, given that the experimental resolution at HERA reaches 1 %. Much higher accuracies are expected at planned facilities as the EIC [33, 34] and in part of the kinematical region at LHeC [105].

We have also derived the $z$-space expressions for all quantities given in the present paper, which were obtained from the $N$-space expressions using the package HarmonicSums [95–98]. Here, we have also reduced all expressions algebraically to obtain a compact basis representation [99]. These and the expressions in $N$-space may be obtained in form of computer-readable files on request via e-mail to Johannes.Bluemlein@desy.de.

## Appendix A: The massive operator matrix elements in $N$-space

In this appendix, we present the massive OMEs in Mellin space to be used in the matching coefficients in the variable flavor number scheme Eqs. (12–15). Thus far, the OMEs $A_{qq,Q}^{\mathsf{PS}}$ and $A_{qg,Q}$ are known completely. The other OMEs have been presented except for the 3-loop constant part $a_{ij}^{(3),0}$ in the un-renormalized OMEs. The OMEs $A_{qq,Q}^\mathsf{{NS}}$ and $A_{gq,Q}^\mathsf{{S}}$ are presented elsewhere [42, 43].

The transition matrix elements $A_{qq,Q}^{\mathsf{PS}}$ and $A_{qg,Q}$ are given by:

303 $$\begin{aligned} A_{qq,Q}^{\mathsf{PS}}&= \tfrac{1}{2}[1 + (-1)^N] \nonumber \\&\quad \times \left\{ {a_s^3} {C_F} {N_F} {T_F^2} \left\{ L_M^2 \Biggl [ \frac{32 \big (N^2+N+2\big )^2 S_1}{3 (N-1) N^2 (N+1)^2 (N+2)}\right. \right. \nonumber \\&\quad -\frac{32 P_{222}}{9 (N-1) N^3 (N+1)^3 (N+2)^2} \Biggr ] \nonumber \\&\quad +\left[ -\frac{32 P_{224}}{27 (N-1) N^4 (N+1)^4 (N+2)^3}\right. \nonumber \\&\quad +\frac{64 P_{222} S_1}{9 (N-1) N^3 (N+1)^3 (N+2)^2} \nonumber \\&\quad \left. +\frac{\big (N^2+N+2\big )^2 \Bigl [-\frac{32}{3} S_1^2-\frac{32 S_2}{3}\Bigr ]}{(N-1) N^2 (N+1)^2 (N+2)}\right] L_M \nonumber \\&\quad -\frac{32 P_{226}}{243 (N-1) N^5 (N+1)^5 (N+2)^4} \nonumber \\&\quad +\frac{32 P_{225} S_1}{81 (N-1) N^4 (N+1)^4 (N+2)^3} \nonumber \\&\quad -\frac{32 \big (N^2+N+2\big )^2 }{9 (N-1) N^2 (N+1)^2 (N+2)} L_M^3 \nonumber \\&\quad +\frac{P_{223} \Bigl [-\frac{16}{27} S_1^2-\frac{16 S_2}{27}\Bigr ]}{(N-1) N^3 (N+1)^3 (N+2)^2}\nonumber \\&\quad \left. \left. +\frac{\big (N^2+N+2\big )^2 \Bigl [\frac{80}{27} S_1^3 +\frac{80}{9} S_2 S_1+\frac{160 S_3}{27}+\frac{256 \zeta _3}{9}\Bigr ]}{(N-1) N^2 (N+1)^2 (N+2)}\right\} \right\} , \end{aligned}$$

with

304 $$\begin{aligned} P_{222}&=8 N^7+37 N^6+83 N^5+85 N^4\nonumber \\&\quad +61 N^3+58 N^2-20 N-24 \end{aligned}$$

305 $$\begin{aligned} P_{223}&=40 N^7+185 N^6+430 N^5+521 N^4\nonumber \\&\quad +452 N^3+404 N^2-16 N-96 \end{aligned}$$

306 $$\begin{aligned} P_{224}&=95 N^{10}+712 N^9+2379 N^8+4269 N^7\nonumber \\&\quad +4763 N^6+4569 N^5+3309 N^4+200 N^3 \nonumber \\&\quad -808 N^2-48 N+144\end{aligned}$$

307 $$\begin{aligned} P_{225}&=233 N^{10}+1744 N^9+5937 N^8+11454 N^7\nonumber \\&\quad +14606 N^6+15396 N^5+12030 N^4+3272 N^3 \nonumber \\&\quad -928 N^2-96 N+288 \end{aligned}$$

308 $$\begin{aligned} P_{226}&=1330 N^{13}+13931 N^{12}+66389 N^{11}+187681 N^{10}\nonumber \\&\quad +354532 N^9+492456 N^8+532664 N^7 \nonumber \\&\quad +423970 N^6+204541 N^5+34274 N^4\nonumber \\&\quad -11704 N^3-3408 N^2-1008 N-864 \end{aligned}$$

and

$$\begin{aligned} A_{qg,Q}&= \tfrac{1}{2}[1 + (-1)^N] \nonumber \\&\quad \times \Biggl \{ {a_s^3} \Biggl \{ {C_F} {N_F} {T_F^2} \Biggl [ \Biggl [\frac{8 \big (N^2+N+2\big ) P_{227}}{9 (N-1) N^3 (N+1)^3 (N+2)^2}+\frac{8}{9} \tilde{\gamma }_{qg}^{0} S_1\Biggr ] L_M^3 \nonumber \\&\quad +\Biggl [\frac{4 P_{233}}{9 (N-1) N^4 (N+1)^4 (N+2)^3}\nonumber \\&\quad -\frac{32 \big (5 N^3+8 N^2+19 N+6\big ) S_1}{9 N^2 (N+1) (N+2)}+\tilde{\gamma }_{qg}^{0} \Bigl [-\frac{4}{3} S_1^2-\frac{4 S_2}{3}\Bigr ]\Biggr ] L_M^2 \nonumber \\&\quad +\Biggl [ \frac{16 \big (10 N^3+13 N^2+29 N+6\big ) S_1^2}{9 N^2 (N+1) (N+2)}\nonumber \\&\quad -\frac{16 \big (103 N^4+257 N^3+594 N^2+524 N+120\big ) S_1}{27 N^2 (N+1)^2 (N+2)} \nonumber \\&\quad +\frac{4 P_{235}}{27 (N-1) N^5 (N+1)^5 (N+2)^4}\nonumber \\&\quad +\frac{16 \big (10 N^3+25 N^2+29 N+6\big ) S_2}{9 N^2 (N+1) (N+2)} \nonumber \\&\quad +\tilde{\gamma }_{qg}^{0} \Bigl [\frac{4}{9} S_1^3+\frac{4}{3} S_2 S_1-\frac{16 S_3}{9}\Bigr ]\Biggr ] L_M\nonumber \\&\quad +\frac{8 \big (215 N^4+481 N^3+930 N^2+748 N+120\big ) S_1^2}{81 N^2 (N+1)^2 (N+2)}\nonumber \\&\quad -\frac{64}{9} \frac{ \big (N^2+N+2\big ) P_{227} \zeta _3}{(N-1) N^3 (N+1)^3 (N+2)^2}\nonumber \\&\quad +\frac{P_{237}}{243 (N-1) N^6 (N+1)^6 (N+2)^5} \nonumber \\ \end{aligned}$$

$$\begin{aligned}&\quad -\frac{16 \big (1523 N^5+5124 N^4+11200 N^3+14077 N^2+7930 N+1344\big ) S_1}{243 N^2 (N+1)^3 (N+2)}\nonumber \\&\quad +\frac{8 \big (109 N^4+291 N^3+478 N^2+324 N+40\big ) S_2}{27 N^2 (N+1)^2 (N+2)}\nonumber \\&\quad +\frac{\big (10 N^3+13 N^2+29 N+6\big ) \Bigl [-\frac{16}{81} S_1^3-\frac{16}{27} S_2 S_1\Bigr ]}{N^2 (N+1) (N+2)} \nonumber \\&\quad +\frac{32 \big (5 N^3-16 N^2+N-6\big ) S_3}{81 N^2 (N+1) (N+2)}\nonumber \\&\quad +\tilde{\gamma }_{qg}^{0} \Bigl [-\frac{1}{27} S_1^4-\frac{2}{9} S_2 S_1^2-\frac{8}{27} S_3 S_1-\frac{64}{9} \zeta _3 S_1-\frac{1}{9} S_2^2+\frac{14 S_4}{9}\Bigr ]\Biggr ] \nonumber \\&\quad +{C_A} {N_F} {T_F^2} \Biggl [ L_M^3 \Biggl [ -\frac{64 \big (N^2+N+1\big ) \big (N^2+N+2\big )}{9 (N-1) N^2 (N+1)^2 (N+2)^2}-\frac{8}{9} \tilde{\gamma }_{qg}^{0} S_1\Biggr ]\nonumber \\&\quad +\Biggl [ \frac{8 P_{231}}{9 (N-1) N^2 (N+1)^3 (N+2)^3}\nonumber \\&\quad +\frac{32 \big (5 N^4+20 N^3+47 N^2+58 N+20\big ) S_1}{9 N (N+1)^2 (N+2)^2} \nonumber \\&\quad +\tilde{\gamma }_{qg}^{0} \Bigl [\frac{4}{3} S_1^2+\frac{4 S_2}{3}+\frac{8}{3} S_{-2}\Bigr ] \Biggr ] L_M^2 \nonumber \\&\quad +\Biggl [-\frac{32 \big (5 N^4+20 N^3+41 N^2+49 N+20\big ) S_1^2}{9 N (N+1)^2 (N+2)^2} \nonumber \\&\quad +\frac{16 P_{234}}{27 (N-1) N^4 (N+1)^4 (N+2)^4} \nonumber \\ \end{aligned}$$

309 $$\begin{aligned}&\quad -\frac{32 \big (5 N^4+26 N^3+47 N^2+43 N+20\big ) S_2}{9 N (N+1)^2 (N+2)^2} \nonumber \\&\quad +\frac{16 P_{228} S_1}{27 N (N+1)^3 (N+2)^3} -\frac{64 \big (5 N^2+8 N+10\big ) S_{-2}}{9 N (N+1) (N+2)} \nonumber \\&\quad +\tilde{\gamma }_{qg}^{0} \Bigl [-\frac{4}{9} S_1^3+\frac{4}{3} S_2 S_1-\frac{8 S_3}{9}-\frac{16}{3} S_{-3} \nonumber \\&\quad -\frac{16}{3} S_{2,1}\Bigr ]\Biggr ] L_M -\frac{16 P_{229} S_1^2}{81 N (N+1)^3 (N+2)^3}\nonumber \\&\quad +\frac{8 P_{236}}{243 (N-1) N^5 (N+1)^5 (N+2)^5} \nonumber \\&\quad +\frac{512}{9} \big (N^2+N+1\big ) \big (N^2+N+2\big ) \frac{\zeta _3}{(N-1) N^2 (N+1)^2 (N+2)^2}\nonumber \\&\quad +\frac{16 P_{232} S_1}{243 (N-1) N^2 (N+1)^4 (N+2)^4} \nonumber \\&\quad -\frac{16 P_{230} S_2}{81 N (N+1)^3 (N+2)^3}\nonumber \\&\quad +\frac{64 \big (5 N^4+38 N^3+59 N^2+31 N+20\big ) S_3}{81 N (N+1)^2 (N+2)^2} \nonumber \\&\quad -\frac{32 \big (121 N^3+293 N^2+414 N+224\big ) S_{-2}}{81 N (N+1)^2 (N+2)}\nonumber \\&\quad +\frac{128 \big (5 N^2+8 N+10\big ) S_{-3}}{27 N (N+1) (N+2)} \nonumber \\&\quad +\frac{\big (5 N^4+20 N^3+41 N^2+49 N+20\big ) \Bigl [\frac{32}{81} S_1^3-\frac{32}{27} S_2 S_1+\frac{128}{27} S_{2,1}\Bigr ]}{N (N+1)^2 (N+2)^2} \nonumber \\&\quad +\tilde{\gamma }_{qg}^{0} \Bigl [\frac{1}{27} S_1^4-\frac{2}{9} S_2 S_1^2 \nonumber \\&\quad +\Bigl [\frac{16}{9} S_{2,1}-\frac{40 S_3}{27}\Bigr ]S_1\nonumber \\&\quad +\frac{64}{9} \zeta _3 S_1+\frac{1}{9} S_2^2+\frac{14 S_4}{9}+\frac{32}{9} S_{-4}+\frac{32}{9} S_{3,1} -\frac{16}{9} S_{2,1,1}\Bigr ]\Biggr ] \Biggr \}\Biggr \}, \end{aligned}$$

with the polynomials

310 $$\begin{aligned} P_{227}&=3 N^6+9 N^5-N^4-17 N^3-38 N^2-28 N-24 \end{aligned}$$

311 $$\begin{aligned} P_{228}&=94 N^6+631 N^5+2106 N^4+4243 N^3\nonumber \\&\quad +4878 N^2+2812 N+680 \end{aligned}$$

312 $$\begin{aligned} P_{229}&=103 N^6+694 N^5+2148 N^4+3991 N^3\nonumber \\&\quad +4494 N^2+2704 N+680 \end{aligned}$$

313 $$\begin{aligned} P_{230}&=139 N^6+1093 N^5+3438 N^4\nonumber \\&\quad +5776 N^3+5724 N^2+3220 N+752 \end{aligned}$$

314 $$\begin{aligned} P_{231}&=9 N^8+54 N^7+56 N^6-182 N^5\nonumber \\&\quad -717 N^4-1120 N^3-1012 N^2-672 N-160 \end{aligned}$$

315 $$\begin{aligned} P_{232}&=1244 N^{10}+10557 N^9+40547 N^8+90323 N^7\nonumber \\&\quad +114495 N^6+49344 N^5-69902 N^4 \nonumber \\&\quad -115200 N^3 -64352 N^2-11264 N+864 \end{aligned}$$

316 $$\begin{aligned} P_{233}&=33 N^{11}+231 N^{10}+698 N^9+1290 N^8+1513 N^7\nonumber \\&\quad +1463 N^6+2236 N^5+5096 N^4+7328 N^3 \nonumber \\&\quad +5456 N^2+3456 N+1152 \end{aligned}$$

317 $$\begin{aligned} P_{234}&=99 N^{12}+891 N^{11}+2902 N^{10}+3392 N^9-4300 N^8\nonumber \\&\quad -20914 N^7-33059 N^6-28357 N^5 \nonumber \\&\quad -11406 N^4 +3840 N^3+7568 N^2+4176 N+864 \end{aligned}$$

318 $$\begin{aligned} P_{235}&=159 N^{14}+1590 N^{13}+7223 N^{12}+20982 N^{11}\nonumber \\&\quad +43703 N^{10}+65162 N^9+62553 N^8+30282 N^7 \nonumber \\&\quad -28286 N^6-145968 N^5-257720 N^4-241760 N^3\nonumber \\&\quad -158112 N^2-73728 N-17280 \end{aligned}$$

319 $$\begin{aligned} P_{236}&=3315 N^{15}+39780 N^{14}+194011 N^{13}+471164 N^{12}\nonumber \\&\quad +416251 N^{11}-860568 N^{10}-3525799 N^9 \nonumber \\&\quad -6015120 N^8-6333994 N^7-4373672 N^6\nonumber \\&\quad -1907512 N^5-499824 N^4-217952 N^3 \nonumber \\&\quad -264192 N^2 -160128 N-34560 \end{aligned}$$

320 $$\begin{aligned} P_{237}&=13923 N^{17}+180999 N^{16}+1064857 N^{15}\nonumber \\&\quad +3812487 N^{14}+9348807 N^{13}+16391845 N^{12} \nonumber \\&\quad +20248499 N^{11}+17070917 N^{10}+11536274 N^9\nonumber \\&\quad +11303496 N^8+13846104 N^7+16104128 N^6 \nonumber \\&\quad +22643488 N^5+29337472 N^4+26395008 N^3\nonumber \\&\quad +15388416 N^2+5612544 N+995328. \end{aligned}$$

Next, we present the OMEs, which are known except for the constant term in the un-renormalized massive OME at 3-loop order, $a_{ij}^{(3),0}$.

The OME $A_{Qg}$ except for the term $a_{Qg}^{(3),0}$ reads:

$$\begin{aligned} A_{Qg}&= \tfrac{1}{2}[1 + (-1)^N] \times \Biggl \{{a_s} \tilde{\gamma }_{qg}^{0} {T_F} L_M +{a_s^2} \Biggl \{ \frac{4}{3} \tilde{\gamma }_{qg}^{0} {T_F^2} L_M^2 +{C_F} {T_F} \nonumber \\&\quad \times \Biggl [ \frac{4 (3 N+2) S_1^2}{N^2 (N+2)}+\frac{4 \big (N^4-N^3-20 N^2-10 N-4\big ) S_1}{N^2 (N+1)^2 (N+2)} +\frac{2 P_{283}}{N^4 (N+1)^4 (N+2)}\nonumber \\&\quad +L_M^2 \Biggl [ \frac{2 \big (N^2+N+2\big ) \big (3 N^2+3 N+2\big )}{N^2 (N+1)^2 (N+2)}+2 \tilde{\gamma }_{qg}^{0} S_1\Biggr ]+L_M \Biggl [-\frac{4 P_{245}}{N^3 (N+1)^3 (N+2)}+\frac{16 S_1}{N^2}+\tilde{\gamma }_{qg}^{0} \Bigl [2 S_1^2-2 S_2\Bigr ]\Biggr ]\nonumber \\&\quad +\frac{4 \big (N^4+17 N^3+17 N^2-5 N-2\big ) S_2}{N^2 (N+1)^2 (N+2)} +\tilde{\gamma }_{qg}^{0} \Bigl [\frac{1}{3} S_1^3+S_2 S_1-\frac{4 S_3}{3}\Bigr ]\Biggr ]\nonumber \\&\quad +{C_A} {T_F} \Biggl [ -\frac{4 \big (N^3+8 N^2+11 N+2\big ) S_1^2}{N (N+1)^2 (N+2)^2}-\frac{4 P_{241} S_1}{N (N+1)^3 (N+2)^3} +\frac{4 P_{321}}{(N-1) N^4 (N+1)^4 (N+2)^4}+L_M^2\nonumber \\&\quad \times \Biggl [-\frac{16 \big (N^2+N+1\big ) \big (N^2+N+2\big )}{(N-1) N^2 (N+1)^2 (N+2)^2}-2 \tilde{\gamma }_{qg}^{0} S_1\Biggr ]-\frac{4 \big (7 N^5+21 N^4+13 N^3+21 N^2+18 N+16\big ) S_2}{(N-1) N^2 (N+1)^2 (N+2)^2}+L_M\nonumber \\&\quad \times \Biggl [-\frac{8 P_{291}}{(N-1) N^3 (N+1)^3 (N+2)^3} -\frac{32 (2 N+3) S_1}{(N+1)^2 (N+2)^2}+\tilde{\gamma }_{qg}^{0} \Bigl [-2 S_1^2-2 S_2-4 S_{-2}\Bigr ]\Biggr ]\nonumber \\ \end{aligned}$$

$$\begin{aligned}&\quad +\frac{\big (N^2-N-4\big )}{(N+1)^2 (N+2)^2} 16 (-1)^N S_{-2}+\tilde{\gamma }_{qg}^{0} \Bigl [-\frac{1}{3} S_1^3-3 S_2 S_1-4 (-1)^N S_{-2} S_1 -\frac{8 S_3}{3} -2 (-1)^N S_{-3}+4 S_{-2,1} \Bigr ]\Biggr ]\Biggr \} \nonumber \\&\quad +{a_s^3} \Biggl \{ {T_F^3} \Biggl [ \frac{16}{9} \tilde{\gamma }_{qg}^{0} L_M^3-\frac{16 \tilde{\gamma }_{qg}^{0} \zeta _3}{9}\Biggr ] +{C_A} {T_F^2} \Biggl [ \frac{32 \big (N^3+8 N^2+11 N+2\big ) S_1^3}{9 N (N+1)^2 (N+2)^2}+\frac{8 P_{242} S_1^2}{3 N (N+1)^3 (N+2)^3} \nonumber \\&\quad -\frac{16 P_{314} S_1}{81 (N-1) N^2 (N+1)^4 (N+2)^4}-\frac{32}{9} \frac{\big (5 N^4+8 N^3+17 N^2+43 N+20\big ) \zeta _2}{N (N+1)^2 (N+2)^2} S_1 \nonumber \\&\quad -\frac{32 \big (3 N^3-12 N^2-27 N-2\big ) S_2 S_1}{3 N (N+1)^2 (N+2)^2} +\frac{4}{9} \frac{P_{300} \zeta _2}{(N-1) N^3 (N+1)^3 (N+2)^3} \nonumber \\&\quad +\frac{8 P_{335}}{81 (N-1) N^5 (N+1)^5 (N+2)^5}+\frac{32 \big (9 N^5-4 N^4+N^3+92 N^2+42 N+28\big ) \zeta _3}{9 (N-1) N^2 (N+1)^2 (N+2)^2} \nonumber \\&\quad +L_M^3 \Biggl [ -\frac{448 \big (N^2+N+1\big ) \big (N^2+N+2\big )}{9 (N-1) N^2 (N+1)^2 (N+2)^2} -\frac{56}{9} \tilde{\gamma }_{qg}^{0} S_1\Biggr ]+\frac{8 P_{293} S_2}{3 (N-1) N^3 (N+1)^3 (N+2)^3}\nonumber \\&\quad +\frac{256 \big (N^5+10 N^4+9 N^3+3 N^2+7 N+6\big ) S_3}{9 (N-1) N^2 (N+1)^2 (N+2)^2}\nonumber \\ \end{aligned}$$

$$\begin{aligned}&\quad +L_M^2 \Biggl [ -\frac{8 P_{298}}{9 (N-1) N^3 (N+1)^3 (N+2)^3} +\frac{32 \big (5 N^4+20 N^3-N^2-14 N+20\big ) S_1}{9 N (N+1)^2 (N+2)^2}\nonumber \\&\quad +\tilde{\gamma }_{qg}^{0} \Bigl [-4 S_1^2-4 S_2-8 S_{-2}\Bigr ]\Biggr ] +\frac{\big (N^4+2 N^3+7 N^2+22 N+20\big ) \Bigl [\frac{128}{3} (-1)^N S_{-2}+\frac{64}{3} (-1)^N \zeta _2\Bigr ]}{(N+1)^3 (N+2)^3} \nonumber \\&\quad +\frac{\big (N^2-N-4\big ) \Bigl [-\frac{256}{3} (-1)^N S_1 S_{-2}-\frac{128}{3} (-1)^N S_{-3}+\frac{256}{3} S_{-2,1}-\frac{128}{3} (-1)^N S_1 \zeta _2-32 (-1)^N \zeta _3\Bigr ]}{(N+1)^2 (N+2)^2} \nonumber \\&\quad +\tilde{\gamma }_{qg}^{0} \Bigl [\frac{2}{9} S_1^4+\frac{20}{3} S_2 S_1^2+\frac{32}{3} (-1)^N S_{-3} S_1+\Bigl [\frac{160 S_3}{9}-\frac{64}{3} S_{-2,1}\Bigr ] S_1\nonumber \\&\quad +\frac{8}{9} \big (-2+9 (-1)^N\big ) \zeta _3 S_1+\frac{2}{3} S_2^2 +S_{-2} \big (\frac{32}{3} (-1)^N S_1^2+\frac{32}{3} (-1)^N S_2\big )+12 S_4\nonumber \\&\quad +\frac{16}{3} (-1)^N S_{-4}-\frac{16}{3} S_{3,1} -\frac{32}{3} S_{-2,2} -\frac{32}{3} S_{-3,1}-\frac{16}{3} S_{2,1,1} +\frac{64}{3} S_{-2,1,1}+\Bigl [\frac{2}{3} \big (-3+8 (-1)^N\big ) S_1^2\nonumber \end{aligned}$$

$$\begin{aligned}&\quad +\frac{2}{3} \Bigl [-3+8 (-1)^N\Bigr ] S_2 +\frac{4}{3} \big (1+4 (-1)^N\big ) S_{-2}\Bigr ] \zeta _2\Bigr ] +L_M \Biggl [ \frac{16 P_{323}}{27 (N-1) N^4 (N+1)^4 (N+2)^4}\nonumber \\&\quad -\frac{32 \big (5 N^4+23 N^3+65 N^2+82 N+26\big ) S_1^2}{9 N (N+1)^2 (N+2)^2}+\frac{16 P_{272} S_1}{27 N (N+1)^3 (N+2)^3} -\frac{32 P_{248} S_2}{9 (N-1) N^2 (N+1)^2 (N+2)^2}\nonumber \\&\quad -\frac{64 \big (5 N^2+8 N+10\big ) S_{-2}}{9 N (N+1) (N+2)} +\frac{\big (N^2-N-4\big ) \frac{128}{3} (-1)^N S_{-2} }{(N+1)^2 (N+2)^2}\nonumber \\&\quad +\tilde{\gamma }_{qg}^{0} \Bigl [ -\frac{4}{3} S_1^3-\frac{20}{3} S_2 S_1-\frac{32}{3} (-1)^N S_{-2} S_1 -8 S_3 -\frac{16}{3} \big (1+(-1)^N\big ) S_{-3} -\frac{16}{3} S_{2,1}+\frac{32}{3} S_{-2,1} \Bigr ]\Biggr ] \Biggr ]\nonumber \\&\quad +{C_A} {T_F^2} {N_F}\Bigl [ \frac{16 \big (N^3+8 N^2+11 N+2\big ) S_1^3}{9 N (N+1)^2 (N+2)^2}+\frac{8 P_{240} S_1^2}{3 N (N+1)^3 (N+2)^3}\nonumber \\&\quad +\frac{4}{9} \frac{P_{284} \zeta _2}{(N-1) N^3 (N+1)^3 (N+2)^2} \nonumber \\&\quad -\frac{16 P_{279} S_1}{3 N (N+1)^4 (N+2)^4} +\frac{16 P_{332}}{3 (N-1) N^5 (N+1)^5 (N+2)^5}-\frac{16 \big (3 N^3-12 N^2-27 N-2\big ) S_2 S_1}{3 N (N+1)^2 (N+2)^2}\nonumber \\&\quad +\frac{16}{9} \frac{\big (5 N^4+32 N^3+47 N^2+28 N+20\big ) \zeta _2}{N (N+1)^2 (N+2)^2} S_1+\frac{16}{9}\frac{\big (9 N^5-14 N^4-19 N^3+52 N^2+12 N+8\big ) \zeta _3}{(N-1) N^2 (N+1)^2 (N+2)^2} \nonumber \\ \end{aligned}$$

$$\begin{aligned}&\quad +L_M^3 \Biggl [ -\frac{64 \big (N^2+N+1\big ) \big (N^2+N+2\big )}{9 (N-1) N^2 (N+1)^2 (N+2)^2}-\frac{8}{9} \tilde{\gamma }_{qg}^{0} S_1\Biggr ] +\frac{8 P_{292} S_2}{3 (N-1) N^3 (N+1)^3 (N+2)^3} \nonumber \\&\quad +\frac{128 \big (N^5+10 N^4+9 N^3+3 N^2+7 N+6\big ) S_3}{9 (N-1) N^2 (N+1)^2 (N+2)^2} +L_M^2 \Biggl [ -\frac{8 P_{282}}{9 (N-1) N^2 (N+1)^3 (N+2)^3} \nonumber \\&\quad -\frac{32 \big (5 N^4+20 N^3+47 N^2+58 N+20\big ) S_1}{9 N (N+1)^2 (N+2)^2} +\tilde{\gamma }_{qg}^{0} \Bigl [ -\frac{4}{3} S_1^2 -\frac{4 S_2}{3} -\frac{8}{3} S_{-2}\Bigr ]\Biggr ]\nonumber \\&\quad +\frac{\big (N^4+2 N^3+7 N^2+22 N+20\big ) \Bigl [ \frac{64}{3} (-1)^N S_{-2} +\frac{32}{3} (-1)^N \zeta _2\Bigr ]}{(N+1)^3 (N+2)^3} \nonumber \\&\quad +\frac{\big (N^2-N-4\big ) }{(N+1)^2 (N+2)^2} \times \Bigl [ -\frac{128}{3} (-1)^N S_1 S_{-2} -\frac{64}{3} (-1)^N S_{-3}\nonumber \\&\quad +\frac{128}{3} S_{-2,1} -\frac{64}{3} (-1)^N S_1 \zeta _2 -16 (-1)^N \zeta _3\Bigr ] +\tilde{\gamma }_{qg}^{0} \Bigl [ \frac{1}{9} S_1^4 +\frac{10}{3} S_2 S_1^2 +\frac{16}{3} (-1)^N S_{-3} S_1\nonumber \\ \end{aligned}$$

$$\begin{aligned}&\quad +\Bigl [ \frac{80 S_3}{9} -\frac{32}{3} S_{-2,1}\Bigr ] S_1 +\frac{4}{9} \big (-7+9 (-1)^N\big ) \zeta _3 S_1 +\frac{1}{3} S_2^2 +S_{-2} \Bigl [ \frac{16}{3} (-1)^N S_1^2 +\frac{16}{3} (-1)^N S_2\Bigr ]\nonumber \\&\quad +6 S_4+\frac{8}{3} (-1)^N S_{-4} -\frac{8}{3} S_{3,1} -\frac{16}{3} S_{-2,2} -\frac{16}{3} S_{-3,1} -\frac{8}{3} S_{2,1,1}+\frac{32}{3} S_{-2,1,1} +\Bigl [ \frac{4}{3} \big (-1+2 (-1)^N\big ) S_1^2\nonumber \\&\quad +\frac{4}{3} \big (-1+2 (-1)^N\big ) S_2 +\frac{8}{3} (-1)^N S_{-2}\Bigr ] \zeta _2\Bigr ]+L_M\Biggl [ -\frac{16 \big (10 N^4+43 N^3+106 N^2+131 N+46\big ) S_1^2}{9 N (N+1)^2 (N+2)^2}\nonumber \\&\quad +\frac{8 P_{324}}{27 (N-1) N^4 (N+1)^4 (N+2)^4}+\frac{16 P_{265} S_1}{27 N (N+1)^3 (N+2)^3} -\frac{16 P_{253} S_2}{9 (N-1) N^2 (N+1)^2 (N+2)^2} \nonumber \\&\quad -\frac{64 \big (5 N^2+8 N+10\big ) S_{-2}}{9 N (N+1) (N+2)} +\frac{\big (N^2-N-4\big ) }{(N+1)^2 (N+2)^2} \frac{64}{3} (-1)^N S_{-2} +\tilde{\gamma }_{qg}^{0}\nonumber \\&\quad \times \Bigl [ -\frac{8}{9} S_1^3 -\frac{8}{3} S_2 S_1 -\frac{16}{3} (-1)^N S_{-2} S_1 -\frac{40 S_3}{9} -\frac{8}{3} \big (2+(-1)^N\big ) S_{-3} -\frac{16}{3} S_{2,1} +\frac{16}{3} S_{-2,1} \Bigr ]\Biggr ]\Biggr ]\nonumber \end{aligned}$$

$$\begin{aligned}&\quad +{C_F^2} {T_F} \Biggl [ -\frac{\big (3 N^4+54 N^3+139 N^2+120 N+36\big ) S_1^4}{3 N^2 (N+1)^2 (N+2)}-\frac{4\big (18 N^5-15 N^4-180 N^3-111 N^2-40 N-4\big ) S_1^3}{3 N^3 (N+1)^2 (N+2)} \nonumber \\&\quad +\frac{2 P_{267} S_1^2}{N^3 (N+1)^3 (N+2)}-\frac{4 \big (3 N^4+14 N^3+43 N^2+48 N+20\big )\zeta _2}{N^2 (N+1)^2 (N+2)} S_1^2 \nonumber \\&\quad -\frac{2 \big (3 N^3+24 N^2+27 N+10\big ) S_2 S_1^2}{N^2 (N+1)^2} -\frac{4 P_{306} S_1}{N^5 (N+1)^5 (N+2)} -\frac{4 P_{263} S_2 S_1}{N^3 (N+1)^3 (N+2)}\nonumber \\&\quad -\frac{8 \big (9 N^4+102 N^3+97 N^2-36 N-12\big ) S_3 S_1}{3 N^2 (N+1)^2 (N+2)}-\frac{16 \big (3 N^4+2 N^3+19 N^2+28 N+12\big ) S_{2,1} S_1}{N^2 (N+1)^2 (N+2)}\nonumber \\&\quad -\frac{8 P_{261} \zeta _2 S_1}{N^3 (N+1)^3 (N+2)} -\frac{P_{322}}{N^6 (N+1)^6 (N+2)}+\frac{2}{3} \big (N^2+N+2\big ) \big (153 N^4+306 N^3+165 N^2+12 N+4\big )\nonumber \\&\quad \times \frac{\zeta _3}{N^3 (N+1)^3 (N+2)}+L_M^3 \Biggl [ -\frac{2 \big (N^2+N+2\big ) \big (3 N^2+3 N+2\big )^2}{3 N^3 (N+1)^3 (N+2)}+\frac{16 \big (N^2+N+2\big ) S_1 \big (3 N^2+3 N+2\big )}{3 N^2 (N+1)^2 (N+2)} +\frac{8}{3} \tilde{\gamma }_{qg}^{0} S_1^2\Biggr ] \nonumber \\&\quad +\frac{2 P_{278} S_2}{N^4 (N+1)^4 (N+2)}+\frac{4 P_{270} S_3}{3 N^3 (N+1)^3 (N+2)} -\frac{16 \big (N^2-3 N-2\big ) \big (3 N^2+3 N+2\big ) S_{2,1}}{N^3 (N+1)^2 (N+2)} \nonumber \\&\quad +L_M^2 \Biggl [\frac{4 \big (3 N^4+14 N^3+43 N^2+48 N+20\big ) S_1^2}{N^2 (N+1)^2 (N+2)}-\frac{8 P_{246} S_1}{N^3 (N+1)^3 (N+2)}+\frac{P_{286}}{N^4 (N+1)^4 (N+2)} \nonumber \\&\quad -\frac{12 \big (N^2+N+2\big ) \big (3 N^2+3 N+2\big ) S_2}{N^2 (N+1)^2 (N+2)}-\frac{32 \big (N^2+N+2\big ) S_{-2}}{N^2 (N+1)^2 (N+2)} +\tilde{\gamma }_{qg}^{0} \Bigl [4 S_1^3-12 S_2 S_1 -16 S_{-2} S_1 \nonumber \\&\quad -8 S_3-8 S_{-3}+16 S_{-2,1}\Bigr ]\Biggr ] +\frac{P_{290} \zeta _2}{2 N^4 (N+1)^4 (N+2)}+\frac{16 \big (N^2+N+2\big ) S_{-2} \zeta _2}{N^2 (N+1)^2 (N+2)} +96 \tilde{\gamma }_{qg}^{0} \log (2) \zeta _2 \nonumber \\ \end{aligned}$$

$$\begin{aligned}&\quad +L_M \Biggl [ \frac{64 (N+1) S_1^3}{N^2 (N+2)}-\frac{8 P_{247} S_1^2}{N^3 (N+1)^3 (N+2)}+\frac{16 \big (N^4+10 N^3+3 N^2-18 N-4\big ) S_2 S_1}{N^2 (N+1)^2 (N+2)} \nonumber \\&\quad +\frac{8 P_{280} S_1}{N^4 (N+1)^4 (N+2)} -\frac{P_{311}}{2 N^5 (N+1)^5 (N+2)}-\frac{8 \big (N^2+5 N+2\big ) \big (3 N^2-N+2\big ) S_3}{N^2 (N+1)^2 (N+2)} \nonumber \\&\quad +\frac{4 P_{264} S_2}{N^3 (N+1)^3 (N+2)}+\frac{32 \big (2 N^5+4 N^4-N^3+N^2-2 N+8\big ) S_{-2}}{N^3 (N+1)^2 (N+2)}-\frac{64 (N-1) S_{-3}}{(N+1)^2 (N+2)}\nonumber \\&\quad -\frac{64 \big (N^2+N+2\big ) S_{2,1}}{N^2 (N+1)^2 (N+2)} +\frac{\big (N^2-N+2\big ) \Bigl [128 S_{-2,1}-128 S_{-2} S_1\Bigr ]}{N^2 (N+1) (N+2)}\!+\!\tilde{\gamma }_{qg}^{0} \Bigl [2 S_1^4\!+\!4 S_2 S_1^2+32 S_{-3} S_1+\big (24 S_3-32 S_{2,1}\big )S_1 \nonumber \\&\quad +6 S_2^2+8 S_{-2}^2+16 S_{-2} S_2+20 S_4+40 S_{-4} -8 S_{3,1}-16 S_{-2,2}-32 S_{-3,1}+24 S_{2,1,1}\Bigr ]\nonumber \\&\quad +\frac{48 (N\!-\!1) \big (3 N^2\!+\!3 N-2\big ) \zeta _3}{N^2 (N\!+\!1)^2}\Biggr ]\!+\!\frac{\big (N^2\!+\!N+2\big ) \big (3 N^2+3 N+2\big ) \Bigl [-S_2^2+8 \zeta _2 S_2+6 S_4-16 S_{3,1}+32 S_{2,1,1}-\frac{16}{3} S_1 \zeta _3\Bigr ]}{N^2 (N+1)^2 (N+2)}\nonumber \\&\quad +\tilde{\gamma }_{qg}^{0} \Bigl [-\frac{1}{3} S_1^5-2 S_2 S_1^3+\Bigl [-\frac{8}{3} S_3-16 S_{2,1}\Bigr ] S_1^2-\frac{8}{3} \zeta _3 S_1^2+\Bigl [-S_2^2+6 S_4-16 S_{3,1}+32 S_{2,1,1}\Bigr ] S_1\nonumber \\&\quad +\Bigl [-4 S_1^3+8 S_2 S_1+8 S_{-2} S_1+4 S_3+4 S_{-3}-8 S_{-2,1}\Bigr ] \zeta _2\Bigr ]\Biggr ]+{C_A^2} {T_F} \Biggl [ \frac{P_{254} S_1^4}{9 (N-1) N^2 (N+1)^2 (N+2)^2}\nonumber \\&\quad -\frac{4 P_{285} S_1^3}{9 (N-1) N^2 (N+1)^3 (N+2)^3}-\frac{4}{3}\frac{P_{260} S_1^2\zeta _2}{(N-1) N^2 (N+1)^2 (N+2)^2}+\frac{2 P_{308} S_1^2}{3 (N-1) N^2 (N+1)^4 (N+2)^4}\nonumber \\&\quad +\frac{2 (N-2) \big (55 N^5+347 N^4+379 N^3+213 N^2+326 N+120\big ) S_2 S_1^2}{3 (N-1) N^2 (N+1)^2 (N+2)^2}-\frac{4}{9} \frac{P_{274} S_1 \zeta _3}{(N-1) N^2 (N+1)^2 (N+2)^2} \nonumber \end{aligned}$$

$$\begin{aligned}&\quad -\frac{4}{9} \frac{P_{310} S_1 \zeta _2}{(N-1)^2 N^3 (N+1)^3 (N+2)^3} -\frac{4 P_{333} S_1}{3 (N-1) N^5 (N+1)^5 (N+2)^5}-\frac{4 P_{294} S_2 S_1}{3 (N-1) N^3 (N+1)^3 (N+2)^3}\nonumber \\&\quad +\frac{16 P_{268} S_3 S_1}{9 (N-1) N^2 (N+1)^2 (N+2)^2} -\frac{2}{9} \frac{P_{327}\zeta _2}{(N-1)^2 N^4 (N+1)^4 (N+2)^4}-\frac{4 \big (11 N^4+22 N^3-35 N^2-46 N-24\big ) P_{332}}{3 (N-1)^2 N^6 (N+1)^6 (N+2)^6}\nonumber \\&\quad -\frac{4 \big (11 N^4+22 N^3-35 N^2-46 N-24\big ) \big (9 N^5-14 N^4-19 N^3+52 N^2+12 N+8\big )\zeta _3}{9 (N-1)^2 N^3 (N+1)^3 (N+2)^3}\nonumber \\&\quad +L_M^3 \Biggl [ \frac{8}{3} \tilde{\gamma }_{qg}^{0} S_1^2-\frac{8 \big (N^2+N+2\big ) \big (11 N^4+22 N^3-59 N^2-70 N-48\big ) S_1}{9 (N-1) N^2 (N+1)^2 (N+2)^2}\nonumber \\&\quad +\frac{16 \big (N^2+N+1\big ) \big (N^2+N+2\big ) \big (11 N^4+22 N^3-35 N^2-46 N-24\big )}{9 (N-1)^2 N^3 (N+1)^3 (N+2)^3}\Biggr ]\nonumber \\&\quad -\frac{2 \big (11 N^4+22 N^3-35 N^2-46 N-24\big ) P_{292} S_2}{3 (N-1)^2 N^4 (N+1)^4 (N+2)^4}-32 \frac{\big (N^2+N+1\big ) \big (N^2+N+2\big ) \zeta _2}{(N-1) N^2 (N+1)^2 (N+2)^2} S_{-2} \nonumber \\&\quad -\frac{32 \big (11 N^4+22 N^3-35 N^2-46 N-24\big ) \big (N^5+10 N^4+9 N^3+3 N^2+7 N+6\big ) S_3}{9 (N-1)^2 N^3 (N+1)^3 (N+2)^3}\nonumber \\&\quad +L_M^2 \Biggl [-\frac{4 P_{258} S_1^2}{3 (N-1) N^2 (N+1)^2 (N+2)^2}+\frac{8 P_{309} S_1}{9 (N-1)^2 N^3 (N+1)^3 (N+2)^3}\nonumber \\&\quad +\frac{16 P_{326}}{9 (N-1)^2 N^4 (N+1)^4 (N+2)^4}-\frac{4 \big (N^2+N+2\big ) \big (11 N^4+22 N^3-83 N^2-94 N-72\big ) S_2}{3 (N-1) N^2 (N+1)^2 (N+2)^2}\nonumber \\ \end{aligned}$$

$$\begin{aligned}&\quad -\frac{8 \big (N^2+N+2\big ) \big (11 N^4+22 N^3-59 N^2-70 N-48\big ) S_{-2}}{3 (N-1) N^2 (N+1)^2 (N+2)^2}\nonumber \\&\quad +\tilde{\gamma }_{qg}^{0} \Bigl [4 S_1^3+12 S_2 S_1+16 S_{-2} S_1+4 S_3+4 S_{-3}-8 S_{-2,1}\Bigr ]\Biggr ]\nonumber \\&\quad +\frac{\big (N^4+2 N^3+7 N^2+22 N+20\big ) \big (11 N^4+22 N^3-35 N^2-46 N-24\big ) }{(N-1) N (N+1)^4 (N+2)^4}\times \Bigl [-\frac{16}{3} (-1)^N S_{-2} -\frac{8}{3} (-1)^N \zeta _2\Bigr ]\nonumber \\&\quad +\frac{\big (5 N^5-131 N^3-58 N^2+232 N+96\big ) \Bigl [\frac{32}{3} (-1)^N S_1 S_{-2}+\frac{16}{3} (-1)^N S_1 \zeta _2\Bigr ]}{(N-1) N (N+1)^2 (N+2)^3}\nonumber \\&\quad +\frac{P_{259} \Bigl [\frac{16}{3} (-1)^N S_{-2} S_1^2+\frac{8}{3} (-1)^N \zeta _2 S_1^2\Bigr ]}{(N-1) N^2 (N+1)^2 (N+2)^2}+\frac{\big (N^2+N+2\big ) \big (11 N^4+22 N^3-35 N^2-46 N-24\big ) }{(N-1) N^2 (N+1)^2 (N+2)^2}\nonumber \\&\quad \times \Bigl [\frac{1}{3} S_2^2+\frac{16}{3} (-1)^N S_{-2} S_2 +6 S_4+\frac{8}{3} (-1)^N S_{-4}-\frac{8}{3} S_{3,1}-\frac{16}{3} S_{-2,2} -\frac{16}{3} S_{-3,1}-\frac{8}{3} S_{2,1,1}+\frac{32}{3} S_{-2,1,1}\nonumber \\&\quad +\big (\frac{8}{3} (-1)^N S_2+\frac{8}{3} (-1)^N S_{-2}\big ) \zeta _2\Bigr ]\nonumber \\&\quad +\frac{\big (N^2-N-4\big ) \big (11 N^4+22 N^3-35 N^2-46 N-24\big ) \Big [\frac{16}{3} (-1)^N S_{-3}-\frac{32}{3} S_{-2,1}+4 (-1)^N \zeta _3\Bigr ]}{(N-1) N (N+1)^3 (N+2)^3}\nonumber \\&\quad +\frac{\big (11 N^5+34 N^4-49 N^3-24 N^2-68 N-48\big ) \Big [\frac{16}{3} (-1)^N S_1 S_{-3}-\frac{32}{3} S_1 S_{-2,1}+4 (-1)^N S_1 \zeta _3\Bigr ]}{(N-1) N^2 (N+1) (N+2)^2}\nonumber \\&\quad +\tilde{\gamma }_{qg}^{0} \Big [-\frac{1}{3} S_1^5-10 S_2 S_1^3-16 (-1)^N S_{-3} S_1^2+\Bigl [32 S_{-2,1}-\frac{80 S_3}{3}\Bigr ] S_1^2-\frac{4}{3} \big (-7+9 (-1)^N\big ) \zeta _3 S_1^2\nonumber \\&\quad -8 (-1)^N S_{-4} S_1+\Bigl [-S_2^2-18 S_4+8 S_{3,1}+16 S_{-2,2}+16 S_{-3,1}+8 S_{2,1,1} -32 S_{-2,1,1}\Bigr ] S_1\nonumber \end{aligned}$$

$$\begin{aligned}&\quad +S_{-2} \Bigl [-16 (-1)^N S_1^3-16 (-1)^N S_2 S_1\Bigr ]+\Bigl [-4 \big (-1+2 (-1)^N\big ) S_1^3-8 (-1)^N S_2 S_1 -4 \big (1+2 (-1)^N\big ) S_{-2} S_1\nonumber \\&\quad +\frac{11 S_2}{3}-2 S_3-2 S_{-3}+4 S_{-2,1}\Bigr ] \zeta _2\Bigr ] +L_M \Biggl [-\frac{8 \big (11 N^4+26 N^3-139 N^2-218 N+8\big ) S_1^3}{9 N (N+1)^2 (N+2)^2}\nonumber \\&\quad +\frac{4 P_{312} S_1^2}{9 (N-1)^2 N^3 (N+1)^3 (N+2)^3} -\frac{8 P_{328} S_1}{27 (N-1)^2 N^4 (N+1)^4 (N+2)^4}\nonumber \\&\quad -\frac{8 P_{255} S_2 S_1}{3 (N\!-\!1) N^2 (N\!+\!1)^2 (N\!+\!2)^2} \!-\!\frac{32 \big (2 N^5\!-\!23 N^4-32 N^3\!+\!13 N^2+4 N\!-\!12\big ) S_{-2} S_1}{(N\!-\!1) N^2 (N\!+\!1)^2 (N+2)^2}\!+\!2 \frac{\zeta _3 P_{275}}{(N\!-\!1) N^2 (N\!+\!1)^2 (N\!+\!2)^2}\nonumber \\&\quad -\frac{4 P_{336}}{27 (N-1)^2 N^5 (N+1)^5 (N+2)^5}+\frac{4 P_{313} S_2}{9 (N-1)^2 N^3 (N+1)^3 (N+2)^3}\nonumber \\&\quad -\frac{8 P_{269} S_3}{9 (N-1) N^2 (N+1)^2 (N+2)^2}+\frac{16 P_{302} S_{-2}}{9 (N-1) N^3 (N+1)^3 (N+2)^3}\nonumber \\&\quad +\frac{16 P_{256} S_{-2,1}}{3 (N-1) N^2 (N+1)^2 (N+2)^2} -\frac{16 P_{257} S_{-3}}{3 (N-1) N^2 (N+1)^2 (N+2)^2}\nonumber \\&\quad -\frac{16 \big (N^2+N+2\big ) \big (11 N^4+22 N^3+13 N^2+2 N+24\big ) S_{2,1}}{3 (N-1) N^2 (N+1)^2 (N+2)^2}\nonumber \\&\quad -\frac{\big (N^2-N-4\big ) \big (11 N^4+22 N^3-35 N^2-46 N-24\big ) \frac{16}{3} (-1)^N S_{-2} }{(N-1) N (N+1)^3 (N+2)^3}\nonumber \\&\quad -\frac{\big (11 N^5+34 N^4-49 N^3-24 N^2-68 N-48\big ) \frac{16}{3} (-1)^N S_1 S_{-2} }{(N-1) N^2 (N+1) (N+2)^2}\nonumber \\&\quad +\frac{\big (N^2+N+2\big ) \big (11 N^4+22 N^3-35 N^2-46 N-24\big ) \Bigl [-\frac{8}{3} (-1)^N S_{-3}-2 (-1)^N \zeta _3\Bigr ]}{(N-1) N^2 (N+1)^2 (N+2)^2}+\tilde{\gamma }_{qg}^{0} \Bigl [ 2 S_1^4 +2 S_2^2\nonumber \\ \end{aligned}$$

$$\begin{aligned}&\quad +32 S_2 S_1^2 +8 \big (8+(-1)^N\big ) S_{-3} S_1+\Bigl [40 S_3-16 S_{2,1}-80 S_{-2,1}\Bigr ] S_1 +6 \big (-5+(-1)^N\big ) \zeta _3 S_1 +12 S_{-2}^2\nonumber \\&\quad +S_{-2} \Bigl [8 \big (3+2 (-1)^N\big ) S_1^2+16 S_2\Bigr ]+4 S_4+44 S_{-4}+16 S_{3,1} -56 S_{-2,2}-64 S_{-3,1}+96 S_{-2,1,1} \Bigr ] \Biggr ]\Biggr ]\nonumber \\&\quad +{C_F} {T_F^2} \Biggl [ \Biggl [ \frac{16 \big (N^2+N+2\big ) P_{249}}{9 (N-1) N^3 (N+1)^3 (N+2)^2}+\frac{32}{9} \tilde{\gamma }_{qg}^{0} S_1\Biggr ]L_M^3+\Biggl [-\frac{4 P_{318}}{9 (N-1) N^4 (N+1)^4 (N+2)^3}\nonumber \\&\quad +\frac{32 \big (5 N^3+14 N^2+37 N+18\big ) S_1}{9 N^2 (N+1) (N+2)}+\tilde{\gamma }_{qg}^{0} \Bigl [4 S_1^2-\frac{4 S_2}{3}\Bigr ]\Biggr ]L_M^2 +\Biggl [\frac{16 \big (10 N^3+31 N^2+59 N+18\big ) S_1^2}{9 N^2 (N+1) (N+2)}\nonumber \\&\quad -\frac{16 \big (29 N^4+163 N^3+786 N^2+592 N+192\big ) S_1}{27 N^2 (N+1)^2 (N+2)}+\frac{2 P_{331}}{27 (N-1) N^5 (N+1)^5 (N+2)^4}\nonumber \\&\quad +\frac{16 \big (2 N^4+39 N^3+42 N^2-5 N-2\big ) S_2}{3 N^2 (N+1)^2 (N+2)}+\tilde{\gamma }_{qg}^{0} \Bigl [ \frac{4}{3} S_1^3+4 S_2 S_1-8 S_3\Bigr ]\Biggr ] L_M -\frac{16 \big (N^4-5 N^3-32 N^2-18 N-4\big ) S_1^2}{3 N^2 (N+1)^2 (N+2)}\nonumber \\&\quad -\frac{16}{9} \frac{\big (N^2+N+2\big ) P_{249} \zeta _3}{(N-1) N^3 (N+1)^3 (N+2)^2} +\frac{2}{9} \frac{P_{319} \zeta _2 }{(N-1) N^4 (N+1)^4 (N+2)^3}\nonumber \\&\quad +\frac{32 \big (2 N^5-2 N^4-11 N^3-19 N^2-44 N-12\big ) S_1}{3 N^2 (N+1)^3 (N+2)} -\frac{2 P_{329}}{3 (N-1) N^6 (N+1)^6 (N+2)^2}\nonumber \\&\quad -\frac{80}{9} \frac{\big (N^3+4 N^2+11 N+6\big ) \zeta _2}{N^2 (N+1) (N+2)}S_1-\frac{16 P_{244} S_2}{3 N^3 (N+1)^3 (N+2)} +\frac{(3 N+2) \big (-\frac{32}{9} S_1^3-\frac{32}{3} S_2 S_1\big )}{N^2 (N+2)}\nonumber \\&\quad -\frac{32 \big (3 N^4+48 N^3+43 N^2-22 N-8\big ) S_3}{9 N^2 (N+1)^2 (N+2)}+\frac{128 \big (N^2-3 N-2\big ) S_{2,1}}{3 N^2 (N+1) (N+2)}\nonumber \end{aligned}$$

$$\begin{aligned}&\quad +\tilde{\gamma }_{qg}^{0} \Bigl [-\frac{2}{9} S_1^4-\frac{4}{3} S_2 S_1^2+\big (-\frac{16}{9} S_3-\frac{32}{3} S_{2,1}\big ) S_1-\frac{32}{9} \zeta _3 S_1-\frac{2}{3} S_2^2+4 S_4\nonumber \\&\quad -\frac{32}{3} S_{3,1}+\frac{64}{3} S_{2,1,1}+\big (2 S_2-\frac{10}{3} S_1^2\big ) \zeta _2\Bigr ] \Biggr ] +{C_F} {T_F^2} {N_F}\Biggl [ \Biggl [ \frac{8 \big (N^2+N+2\big ) P_{243}}{9 (N-1) N^3 (N+1)^3 (N+2)^2} +\frac{8}{9} \tilde{\gamma }_{qg}^{0} S_1\Biggr ]L_M^3 \nonumber \\&\quad +\Biggl [ -\frac{4 P_{316}}{9 (N-1) N^4 (N+1)^4 (N+2)^3}+\frac{32 \big (5 N^3+8 N^2+19 N+6\big ) S_1}{9 N^2 (N+1) (N+2)} +\tilde{\gamma }_{qg}^{0} \Bigl [ \frac{4}{3} S_1^2 +\frac{4 S_2}{3}\Bigr ]\Biggr ]L_M^2 \nonumber \\&\quad +L_M \Biggl [ \frac{32 \big (5 N^3+11 N^2+22 N+6\big ) S_1^2}{9 N^2 (N+1) (N+2)} -\frac{32 \big (19 N^4+77 N^3+303 N^2+251 N+78\big ) S_1}{27 N^2 (N+1)^2 (N+2)}\nonumber \\&\quad +\frac{2 P_{330}}{27 (N-1) N^5 (N+1)^5 (N+2)^4}+\frac{16 P_{277} S_2}{3 (N-1) N^3 (N+1)^3 (N+2)^2} +\tilde{\gamma }_{qg}^{0} \Bigl [ \frac{8}{9} S_1^3 +\frac{8}{3} S_2 S_1 -\frac{56 S_3}{9}\Bigr ]\Biggr ]\nonumber \\&\quad -\frac{8}{9} \frac{\big (N^2+N+2\big ) P_{243} \zeta _3}{(N-1) N^3 (N+1)^3 (N+2)^2} -\frac{8 \big (N^4-5 N^3-32 N^2-18 N-4\big ) S_1^2}{3 N^2 (N+1)^2 (N+2)}-\frac{2}{9} \frac{P_{320} \zeta _2}{(N-1) N^4 (N+1)^4 (N+2)^3}\nonumber \\&\quad +\frac{16 \big (2 N^5-2 N^4-11 N^3-19 N^2-44 N-12\big ) S_1}{3 N^2 (N+1)^3 (N+2)}-\frac{16}{9} \frac{\big (5 N^3+11 N^2+28 N+12\big ) \zeta _2}{N^2 (N+1) (N+2)} S_1 \nonumber \\&\quad -\frac{4 P_{338}}{3 (N-1) N^6 (N+1)^6 (N+2)^5}-\frac{8 P_{315} S_2}{3 (N-1) N^4 (N+1)^4 (N+2)^3}\nonumber \\&\quad +\frac{(3 N+2) \Bigl [ -\frac{16}{9} S_1^3 -\frac{16}{3} S_2 S_1\Bigr ]}{N^2 (N+2)} -\frac{16 P_{281} S_3}{9 (N-1) N^3 (N+1)^3 (N+2)^2}\nonumber \\&\quad +\frac{64 \big (N^2-3 N-2\big ) S_{2,1}}{3 N^2 (N+1) (N+2)}+\tilde{\gamma }_{qg}^{0} \Bigl [ -\frac{1}{9} S_1^4 -\frac{2}{3} S_2 S_1^2\nonumber \\ \end{aligned}$$

$$\begin{aligned}&\quad -\frac{4}{3} \zeta _2 S_1^2+\Bigl [ -\frac{8}{9} S_3 -\frac{16}{3} S_{2,1}\Bigr ] S_1 -\frac{8}{9} \zeta _3 S_1 -\frac{1}{3} S_2^2+2 S_4 -\frac{16}{3} S_{3,1} +\frac{32}{3} S_{2,1,1}\Bigr ] \Biggr ]\nonumber \\&\quad +{C_F} {C_A} {T_F}\Biggl [ -\frac{2 P_{238} S_1^4}{9 (N-1) N^2 (N+1)^2 (N+2)^2}+\frac{8 P_{296} S_1^3}{9 (N-1) N^3 (N+1)^3 (N+2)^3}\nonumber \\&\quad -\frac{8}{3} \frac{P_{252} S_1^2 \zeta _2}{(N-1) N^2 (N+1)^2 (N+2)^2}-\frac{4 P_{317} S_1^2}{3 (N-1) N^3 (N+1)^4 (N+2)^4}\nonumber \\&\quad +\frac{4 P_{262} S_2 S_1^2}{3 (N-1) N^2 (N+1)^2 (N+2)^2}-\frac{4}{9} \frac{P_{271} S_1\zeta _3}{(N-1) N^2 (N+1)^2 (N+2)^2}\nonumber \\&\quad +\frac{8 P_{334} S_1}{3 (N-1) N^5 (N+1)^5 (N+2)^5}+\frac{4}{9} \frac{P_{287} S_1\zeta _2}{(N-1) N^3 (N+1)^3 (N+2)^2}\nonumber \\&\quad +\frac{8 P_{299} S_2 S_1}{3 (N-1) N^3 (N+1)^3 (N+2)^3}+\frac{8 P_{276} S_3 S_1}{9 (N-1) N^2 (N+1)^2 (N+2)^2}\nonumber \\&\quad -\frac{16 \big (11 N^5+45 N^4-3 N^3-145 N^2-176 N-20\big ) S_{2,1} S_1}{3 (N-1) N (N+1)^2 (N+2)^2}-\frac{2}{9} \frac{P_{289}\zeta _3}{(N-1) N^3 (N+1)^3 (N+2)^2}\nonumber \\&\quad -\frac{2 \big (N^2+N+2\big ) \big (N^4+2 N^3-16 N^2-17 N-6\big ) S_2^2}{3 (N-1) N^2 (N+1)^2 (N+2)^2}-\frac{1}{18} \frac{P_{305} \zeta _2}{(N-1) N^3 (N+1)^4 (N+2)^2}\nonumber \\&\quad +\frac{P_{337}}{3 (N-1) N^5 (N+1)^6 (N+2)^5}+L_M^3 \Biggl [ -\frac{8 \big (N^2+N+2\big ) \big (N^2+N+6\big ) \big (7 N^2+7 N+4\big ) S_1}{9 (N-1) N^2 (N+1)^2 (N+2)^2}\nonumber \\&\quad -\frac{16}{3} \tilde{\gamma }_{qg}^{0} S_1^2 -\frac{2 \big (N^2+N+2\big ) \big (3 N^2+3 N+2\big ) \big (11 N^4+22 N^3-59 N^2-70 N-48\big )}{9 (N-1) N^3 (N+1)^3 (N+2)^2}\Biggr ]\nonumber \\&\quad -\frac{4 P_{307} S_2}{3 (N-1) N^3 (N+1)^4 (N+2)^3}-4 \frac{\big (N^2+N+2\big ) \big (3 N^4+6 N^3+7 N^2+4 N+4\big ) \zeta _2}{(N-1) N^2 (N+1)^2 (N+2)^2} S_2\nonumber \\&\quad +\frac{4 P_{288} S_3}{9 (N-1) N^3 (N+1)^3 (N+2)^2}+\frac{4 \big (N^2+N+2\big ) \big (19 N^4+38 N^3-22 N^2-41 N-30\big ) S_4}{(N-1) N^2 (N+1)^2 (N+2)^2}\nonumber \end{aligned}$$

$$\begin{aligned}&\quad -\frac{16 \big (N^2-3 N-2\big ) \big (11 N^4+22 N^3-35 N^2-46 N-24\big ) S_{2,1}}{3 (N-1) N^3 (N+1)^2 (N+2)^2}\nonumber \\&\quad -\frac{8 \big (N^2+N+2\big ) \big (31 N^4+62 N^3-73 N^2-104 N-60\big ) S_{3,1}}{3 (N-1) N^2 (N+1)^2 (N+2)^2} +L_M^2 \Biggl [\frac{8 P_{239} S_1^2}{3 (N-1) N^2 (N+1)^2 (N+2)^2}\nonumber \\&\quad -\frac{8 P_{297} S_1}{9 (N-1) N^3 (N+1)^3 (N+2)^3} +\frac{P_{304}}{9 (N-1) N^3 (N+1)^3 (N+2)^3}+\frac{8 \big (N^2+N+2\big ) \big (N^4+2 N^3+8 N^2+7 N+18\big ) S_2}{3 (N-1) N^2 (N+1)^2 (N+2)^2}\nonumber \\&\quad +\tilde{\gamma }_{qg}^{0} \Bigl [-8 S_1^3+4 S_3+6 S_{-2}+4 S_{-3}-8 S_{-2,1}\Bigr ]\Biggr ]+\frac{8 \big (N^2+N+2\big ) \big (35 N^4+70 N^3-137 N^2-172 N-84\big ) S_{2,1,1}}{3 (N-1) N^2 (N+1)^2 (N+2)^2}\nonumber \\&\quad -\frac{8 \big (N^2+N+2\big ) S_{-2} \zeta _2}{N^2 (N+1)^2 (N+2)}-48 \tilde{\gamma }_{qg}^{0} \log (2) \zeta _2+\frac{P_{251} \Bigl [16 (-1)^N S_{-2}+8 (-1)^N \zeta _2\Bigr ]}{N (N+1)^4 (N+2)^3}\nonumber \\&\quad +\frac{P_{250} \Bigl [32 (-1)^N S_1 S_{-2}+16 (-1)^N S_1 \zeta _2\Bigr ]}{N (N+1)^3 (N+2)^3}+\frac{\big (3 N^5+4 N^4+31 N^3+62 N^2+20 N+8\big ) \Bigl [16 (-1)^N S_{-2} S_1^2+8 (-1)^N \zeta _2 S_1^2\Bigr ]}{N^2 (N+1)^2 (N+2)^2}\nonumber \\&\quad +\frac{\big (N^2+N+2\big ) \big (3 N^2+3 N+2\big )}{N^2 (N+1)^2 (N+2)}\times \Bigl [ \Bigl [8 (-1)^N S_2+8 (-1)^N S_{-2}\Bigr ] \zeta _2 +16 (-1)^N S_{-2} S_2+8 (-1)^N S_{-4}\nonumber \\&\quad -16 S_{-2,2}-16 S_{-3,1}+32 S_{-2,1,1}\Bigr ]+\frac{\big (6 N^5+33 N^4+66 N^3+77 N^2+58 N+16\big ) }{N (N+1)^3 (N+2)^2} \Bigl [16 (-1)^N S_{-3}\nonumber \\&\quad -32 S_{-2,1}+12 (-1)^N \zeta _3\Bigr ]+\frac{\big (3 N^5+8 N^4+27 N^3+46 N^2+20 N+8\big ) }{N^2 (N+1)^2 (N+2)^2} \Bigl [16 (-1)^N S_1 S_{-3}-32 S_1 S_{-2,1}\nonumber \\ \end{aligned}$$

$$\begin{aligned}&\quad +12 (-1)^N S_1 \zeta _3\Bigr ] +\tilde{\gamma }_{qg}^{0} \Bigl [ \frac{2}{3} S_1^5+12 S_2 S_1^3+16 (-1)^N S_{-3} S_1^2+\Bigl [\frac{88 S_3}{3}+16 S_{2,1}-32 S_{-2,1}\Bigr ] S_1^2\nonumber \\&\quad +\frac{4}{3} \big (-5+9 (-1)^N\big ) \zeta _3 S_1^2 +8 (-1)^N S_{-4} S_1+\Bigl [2 S_2^2+12 S_4+8 S_{3,1}-16 S_{-2,2}-16 S_{-3,1}-40 S_{2,1,1}\nonumber \\&\quad +32 S_{-2,1,1}\Bigr ] S_1+S_{-2} \Bigl [16 (-1)^N S_1^3+16 (-1)^N S_2 S_1+32\Bigr ]+\Bigl [8 (-1)^N S_1^3 +4 \big (-1+2(-1)^N\big ) S_{-2} S_1\nonumber \\&\quad -2 S_3-2 S_{-3} +4 S_{-2,1}\Bigr ] \zeta _2\Bigr ]+L_M \Biggl [ \frac{8 \big (11 N^5-46 N^4-499 N^3-866 N^2-496 N-144\big ) S_1^3}{9 N^2 (N+1)^2 (N+2)^2}\nonumber \\&\quad -\frac{4 P_{301} S_1^2}{9 (N-1) N^3 (N+1)^3 (N+2)^3}-\frac{8 (N+3) \big (13 N^5+114 N^4+93 N^3+52 N^2+8 N+8\big ) S_2 S_1}{3 (N-1) N^2 (N+1)^2 (N+2)^2}\nonumber \\&\quad +\frac{8 P_{303} S_1}{27 (N-1) N^4 (N+1)^3 (N+2)^2}+\frac{64 \big (N^3-13 N^2-14 N-2\big ) S_{-2} S_1}{N^2 (N+1)^2 (N+2)} -\frac{6 P_{266} \zeta _3}{(N-1) N^2 (N+1)^2 (N+2)^2}\nonumber \\&\quad -\frac{P_{325}}{54 (N-1) N^5 (N+1)^4 (N+2)^3}+\frac{4 P_{295} S_2}{3 (N-1) N^3 (N+1)^3 (N+2)^3} \nonumber \\&\quad -\frac{8 P_{273} S_3}{9 (N-1) N^2 (N+1)^2 (N+2)^2}-\frac{16 \big (3 N^5-40 N^4-87 N^3-54 N^2-10 N+12\big ) S_{-2}}{N^3 (N+1)^3 (N+2)} \nonumber \\&\quad +\frac{16 \big (3 N^4+8 N^3-5 N^2-6 N+8\big ) S_{-3}}{N^2 (N+1)^2 (N+2)}+\frac{16 \big (3 N^4+2 N^3+39 N^2+32 N-12\big ) S_{-2,1}}{N^2 (N+1)^2 (N+2)}\nonumber \\ \end{aligned}$$

321 $$\begin{aligned}&\quad +\frac{48 \big (N^2+N+2\big )^2 S_{2,1}}{(N-1) N (N+1) (N+2)^2}-\frac{\big (N^2-N-4\big ) \big (3 N^2+3 N+2\big ) 16 (-1)^N S_{-2} }{N (N+1)^3 (N+2)^2} \nonumber \\&\quad -\frac{\big (3 N^5+8 N^4\!+\!27 N^3+46 N^2+20 N\!+\!8\big ) 16 (-1)^N S_1 S_{-2} }{N^2 (N\!+\!1)^2 (N\!+\!2)^2} \!+\!\frac{\big (N^2\!+\!N\!+\!2\big ) \big (3 N^2+3 N\!+\!2\big ) \big (-8 (-1)^N S_{-3}\!-\!6 (-1)^N \zeta _3\big )}{N^2 (N+1)^2 (N\!+\!2)}\nonumber \\&\quad +\tilde{\gamma }_{qg}^{0} \Bigl [-4 S_1^4-36 S_2 S_1^2 -8 \big (3+2 (-1)^N\big ) S_{-2} S_1^2 -8 (-1)^N S_{-3} S_1\nonumber \\&\quad +\big (-32 S_3+48 S_{2,1}+16 S_{-2,1}\big ) S_1 -6 \big (-5+(-1)^N\big ) \zeta _3 S_1 +8 S_2^2-12 S_{-2}^2-20 S_{-4}-8 S_{3,1}\nonumber \\&\quad -8 S_{-2,2}-24 S_{2,1,1}+32 S_{-2,1,1}\Bigr ]\Biggr ] \Biggr ]+a_{Qg}^{(3),0} \Biggr \} \Biggr \}, \end{aligned}$$

where

322 $$\begin{aligned} P_{238}&= N^6-93 N^5-444 N^4-317 N^3\nonumber \\&\quad +\,329 N^2+296 N+84 \end{aligned}$$

323 $$\begin{aligned} P_{239}&= N^6-9 N^5-120 N^4-137 N^3\nonumber \\&\quad +\,29 N^2+56 N+36\end{aligned}$$

324 $$\begin{aligned} P_{240}&= N^6+6 N^5+7 N^4+4 N^3\nonumber \\&\quad +\,18 N^2+16 N-8\end{aligned}$$

325 $$\begin{aligned} P_{241}&= N^6+8 N^5+23 N^4+54 N^3\nonumber \\&\quad +\,94 N^2+72 N+8 \end{aligned}$$

326 $$\begin{aligned} P_{242}&= 2 N^6+11 N^5+8 N^4-7 N^3\nonumber \\&\quad +\,14 N^2+12 N-24\end{aligned}$$

327 $$\begin{aligned} P_{243}&= 3 N^6+9 N^5-N^4-17 N^3\nonumber \\&\quad -\,38 N^2-28 N-24\end{aligned}$$

328 $$\begin{aligned} P_{244}&= 3 N^6+30 N^5+15 N^4-64 N^3\nonumber \\&\quad -\,56 N^2-20 N-8\end{aligned}$$

329 $$\begin{aligned} P_{245}&= 5 N^6+15 N^5+36 N^4+51 N^3\nonumber \\&\quad +\,25 N^2+8 N+4\end{aligned}$$

330 $$\begin{aligned} P_{246}&= 5 N^6+18 N^5+51 N^4+84 N^3\nonumber \\&\quad +\,60 N^2+34 N+12\end{aligned}$$

331 $$\begin{aligned} P_{247}&= 5 N^6+26 N^5+97 N^4+160 N^3\nonumber \\&\quad +\,135 N^2+79 N+22\end{aligned}$$

332 $$\begin{aligned} P_{248}&= 5 N^6+42 N^5+84 N^4+35 N^3\nonumber \\&\quad +\,40 N^2+34 N+48\end{aligned}$$

333 $$\begin{aligned} P_{249}&= 6 N^6+18 N^5+7 N^4-16 N^3\nonumber \\&\quad -\,31 N^2-20 N-12\end{aligned}$$

334 $$\begin{aligned} P_{250}&= 6 N^6+47 N^5+136 N^4+223 N^3\nonumber \\&\quad +\,256 N^2+172 N+32\end{aligned}$$

335 $$\begin{aligned} P_{251}&= 9 N^6+27 N^5-65 N^4-319 N^3\nonumber \\&\quad -\,404 N^2-200 N-40\end{aligned}$$

336 $$\begin{aligned} P_{252}&= 10 N^6-6 N^5-39 N^4-44 N^3\nonumber \\&\quad -\,97 N^2+20 N+12\end{aligned}$$

337 $$\begin{aligned} P_{253}&= 10 N^6+63 N^5+105 N^4+31 N^3\nonumber \\&\quad +\,17 N^2+14 N+48\end{aligned}$$

338 $$\begin{aligned} P_{254}&= 11 N^6-15 N^5-327 N^4-181 N^3\nonumber \\&\quad +\,292 N^2-20 N-48\end{aligned}$$

339 $$\begin{aligned} P_{255}&= 11 N^6+15 N^5-285 N^4-319 N^3\nonumber \\&\quad -\,254 N^2-368 N-240\end{aligned}$$

340 $$\begin{aligned} P_{256}&= 11 N^6+33 N^5-189 N^4-361 N^3\nonumber \\&\quad -\,194 N^2-92 N-72\end{aligned}$$

341 $$\begin{aligned} P_{257}&= 11 N^6+33 N^5-114 N^4-247 N^3\nonumber \\&\quad -\,263 N^2-176 N-108\end{aligned}$$

342 $$\begin{aligned} P_{258}&= 11 N^6+33 N^5-87 N^4-85 N^3\nonumber \\&\quad +\,4 N^2-116 N-48\end{aligned}$$

343 $$\begin{aligned} P_{259}&= 11 N^6+57 N^5-39 N^4-109 N^3\nonumber \\&\quad -\,44 N^2-116 N-48\end{aligned}$$

344 $$\begin{aligned} P_{260}&= 11 N^6+81 N^5+9 N^4-133 N^3\nonumber \\&\quad -\,92 N^2-116 N-48\end{aligned}$$

345 $$\begin{aligned} P_{261}&= 13 N^6+36 N^5+39 N^4+8 N^3\nonumber \\&\quad -\,21 N^2-29 N-10\end{aligned}$$

346 $$\begin{aligned} P_{262}&= 17 N^6+51 N^5+390 N^4+359 N^3\nonumber \\&\quad -\,389 N^2-200 N-84\end{aligned}$$

347 $$\begin{aligned} P_{263}&= 18 N^6+87 N^5+57 N^4-119 N^3\nonumber \\&\quad -\,131 N^2-60 N-20\end{aligned}$$

348 $$\begin{aligned} P_{264}&= 23 N^6+39 N^5+75 N^4+157 N^3\nonumber \\&\quad +\,96 N^2+70 N+28 \end{aligned}$$

349 $$\begin{aligned} P_{265}&=29 N^6+176 N^5+777 N^4+1820 N^3\nonumber \\&\quad +1878 N^2+776 N+232\end{aligned}$$

350 $$\begin{aligned} P_{266}&=45 N^6+135 N^5-91 N^4-407N^3\nonumber \\&\quad -214 N^2+12 N-248\end{aligned}$$

351 $$\begin{aligned} P_{267}&=51 N^6+140 N^5+227 N^4+208N^3\nonumber \\&\quad -202 N^2-96 N-8\end{aligned}$$

352 $$\begin{aligned} P_{268}&=55 N^6+141 N^5-195 N^4-401N^3 \nonumber \\&\quad -772 N^2-748 N-384\end{aligned}$$

353 $$\begin{aligned} P_{269}&=55 N^6+165 N^5-420 N^4-899N^3 \nonumber \\&\quad -1561 N^2-1336 N-1188\end{aligned}$$

354 $$\begin{aligned} P_{270}&=57 N^6+297 N^5+519 N^4+399N^3 \nonumber \\&\quad +92 N^2-68 N-16\end{aligned}$$

355 $$\begin{aligned} P_{271}&=67 N^6+93 N^5+351 N^4+259N^3 \nonumber \\&\quad -1054 N^2-556 N-312\end{aligned}$$

356 $$\begin{aligned} P_{272}&=76 N^6+487 N^5+1692 N^4+3271N^3\nonumber \\&\quad +3186 N^2+1516 N+536\end{aligned}$$

357 $$\begin{aligned} P_{273}&=77 N^6+195 N^5+627 N^4+977N^3\nonumber \\&\quad -128 N^2-452 N+432\end{aligned}$$

358 $$\begin{aligned} P_{274}&=77 N^6+339 N^5-105 N^4-487N^3 \nonumber \\&\quad -356 N^2-668 N-240\end{aligned}$$

359 $$\begin{aligned} P_{275}&=83 N^6+249 N^5-111 N^4-637N^3 \nonumber \\&\quad -956 N^2-596 N-624\end{aligned}$$

360 $$\begin{aligned} P_{276}&=97 N^6+591 N^5+1311 N^4+229N^3 \nonumber \\&\quad -712 N^2+308 N+192\end{aligned}$$

361 $$\begin{aligned} P_{277}&=N^8+24 N^7+62 N^6+8 N^5-123N^4\nonumber \\&\quad -128 N^3-108 N^2-72 N-48\end{aligned}$$

362 $$\begin{aligned} P_{278}&=N^8+427 N^7+1133 N^6+697 N^5-434N^4 \nonumber \\&\quad -636 N^3-244 N^2-64 N-16\end{aligned}$$

363 $$\begin{aligned} P_{279}&=2 N^8+22 N^7+117 N^6+386 N^5+759N^4\nonumber \\&\quad +810 N^3+396 N^2+72 N+32\end{aligned}$$

364 $$\begin{aligned} P_{280}&=2 N^8+29 N^7+179 N^6+441 N^5+529N^4 \nonumber \\&\quad +332 N^3+172 N^2+92 N+24\end{aligned}$$

365 $$\begin{aligned} P_{281}&=3 N^8+54 N^7+118 N^6-44 N^5-353N^4\nonumber \\&\quad -314 N^3-272 N^2-200 N-144\end{aligned}$$

366 $$\begin{aligned} P_{282}&=9 N^8+54 N^7+56 N^6-182 N^5-717N^4\nonumber \\&\quad -1120 N^3-1012 N^2-672 N-160\end{aligned}$$

367 $$\begin{aligned} P_{283}&=12 N^8+52 N^7+132 N^6+216 N^5+191N^4\nonumber \\&\quad +54 N^3-25 N^2-20 N-4\end{aligned}$$

368 $$\begin{aligned} P_{284}&=15 N^8+36 N^7+50 N^6-252 N^5-357N^4 \nonumber \\&\quad +152 N^3-68 N^2+88 N+48\end{aligned}$$

369 $$\begin{aligned} P_{285}&=18 N^8+101 N^7+128 N^6+208 N^5+190N^4\nonumber \\&\quad -769 N^3-1200 N^2-212 N-48 \end{aligned}$$

370 $$\begin{aligned} P_{286}&=33 N^8+132 N^7+350 N^6+636 N^5+685N^4\nonumber \\&\quad +528 N^3+292 N^2+128 N+32\end{aligned}$$

371 $$\begin{aligned} P_{287}&=121 N^8+370 N^7+924 N^6+358 N^5-381N^4\nonumber \\&\quad +184 N^3-1096 N^2-48 N+144\end{aligned}$$

372 $$\begin{aligned} P_{288}&=321 N^8+1674 N^7+2360 N^6-1378 N^5-6565N^4 \nonumber \\&\quad -5992 N^3-1972 N^2+128 N-96\end{aligned}$$

373 $$\begin{aligned} P_{289}&=507 N^8+2190 N^7+3002 N^6+1692 N^5-681N^4\nonumber \\&\quad -2554 N^3-404 N^2+664 N+192\end{aligned}$$

374 $$\begin{aligned} P_{290}&=633 N^8+2532 N^7+5036 N^6+6174 N^5+4307N^4\nonumber \\&\quad +1182 N^3-176 N^2-184 N-48\end{aligned}$$

375 $$\begin{aligned} P_{291}&=N^9+6 N^8+15 N^7+25 N^6+36 N^5+85N^4 \nonumber \\&\quad +128 N^3+104 N^2+64 N+16\end{aligned}$$

376 $$\begin{aligned} P_{292}&=N^9+21 N^8+85 N^7+105 N^6+42 N^5+290N^4\nonumber \\&\quad +600 N^3+456 N^2+256 N+64\end{aligned}$$

377 $$\begin{aligned} P_{293}&=4 N^9+53 N^8+193 N^7+233 N^6+87 N^5+554N^4\nonumber \\&\quad +1172 N^3+904 N^2+512 N+128\end{aligned}$$

378 $$\begin{aligned} P_{294}&=6 N^9+93 N^8+576 N^7+1296 N^6+586N^5 \nonumber \\&\quad +359 N^4+2000 N^3+1996 N^2\nonumber \\&\quad +1488 N+384 \end{aligned}$$

379 $$\begin{aligned} P_{295}&=25 N^9-43 N^8-424 N^7+462 N^6\nonumber \\&\quad +4345 N^5+7513 N^4+6446 N^3\nonumber \\&\quad +4020 N^2+1944 N+480\end{aligned}$$

380 $$\begin{aligned} P_{296}&=36 N^9+156 N^8-115 N^7-1116 N^6\nonumber \\&\quad -1251 N^5-78 N^4+300 N^3\nonumber \\&\quad +84 N^2-128 N-48\end{aligned}$$

381 $$\begin{aligned} P_{297}&=40 N^9+273 N^8+635 N^7+613 N^6\nonumber \\&\quad +119 N^5-2 N^4-314 N^3-668 N^2\nonumber \\&\quad +24 N+144\end{aligned}$$

382 $$\begin{aligned} P_{298}&=45 N^9+270 N^8+724 N^7+1262 N^6\nonumber \\&\quad +1731 N^5+2740 N^4+3484 N^3+2928 N^2\nonumber \\&\quad +1696 N+384\end{aligned}$$

383 $$\begin{aligned} P_{299}&=66 N^9+534 N^8+1409 N^7+1080 N^6\nonumber \\&\quad -933 N^5-1116 N^4+588 N^3+996 N^2\nonumber \\&\quad +736 N+240\end{aligned}$$

384 $$\begin{aligned} P_{300}&=69 N^9+366 N^8+1100 N^7+1894 N^6\nonumber \\&\quad +2451 N^5+5276 N^4+7460 N^3+5352 N^2\nonumber \\&\quad +3008 N+672\end{aligned}$$

385 $$\begin{aligned} P_{301}&=80 N^9+441 N^8+568 N^7-592 N^6\nonumber \\&\quad -1202 N^5+2003 N^4+4106 N^3+3116 N^2\nonumber \\&\quad +2712 N+864\end{aligned}$$

386 $$\begin{aligned} P_{302}&=94 N^9+597 N^8+1508 N^7\nonumber \\&\quad +2086 N^6+1517 N^5+1381 N^4+2731 N^3\nonumber \\&\quad +3802 N^2+2916 N+648\end{aligned}$$

387 $$\begin{aligned} P_{303}&=251 N^9+1586 N^8+4206 N^7\nonumber \\&\quad +6764 N^6+4008 N^5-2242 N^4\nonumber \\&\quad +13 N^3+7122 N^2\nonumber \\&\quad +6156 N+1944\end{aligned}$$

388 $$\begin{aligned} P_{304}&=489 N^9+2934 N^8+7636 N^7\nonumber \\&\quad +12206 N^6+6675 N^5-12692 N^4\nonumber \\&\quad -24608N^3-16272 N^2\nonumber \\&\quad -2864 N+2304\end{aligned}$$

389 $$\begin{aligned} P_{305}&=891 N^9+5751 N^8+15070 N^7\nonumber \\&\quad +21430 N^6+37623 N^5+55339 N^4\nonumber \\&\quad +44064N^3+25144 N^2\nonumber \\&\quad +9488 N+1776\end{aligned}$$

390 $$\begin{aligned} P_{306}&=10 N^{10}+62 N^9+407 N^8\nonumber \\&\quad +1119 N^7+1405 N^6+889 N^5\nonumber \\&\quad +240 N^4-90 N^3-114 N^2\nonumber \\&\quad -48 N-8 \end{aligned}$$

391 $$\begin{aligned} P_{307}&=36 N^{10}+456 N^9+2448 N^8\nonumber \\&\quad +7171 N^7+12399 N^6+13213 N^5\nonumber \\&\quad +8997 N^4+5000N^3+2888 N^2\nonumber \\&\quad +992 N+112\end{aligned}$$

392 $$\begin{aligned} P_{308}&=37 N^{10}+392 N^9+2106 N^8\nonumber \\&\quad +6514 N^7+9211 N^6+1258 N^5\nonumber \\&\quad -9218 N^4-6116N^3-72 N^2\nonumber \\&\quad -752 N-192\end{aligned}$$

393 $$\begin{aligned} P_{309}&=85 N^{10}+425 N^9+830 N^8\nonumber \\&\quad +788 N^7-521 N^6-325 N^5\nonumber \\&\quad +2238 N^4+2568N^3+968 N^2\nonumber \\&\quad -1296 N-576\end{aligned}$$

394 $$\begin{aligned} P_{310}&=103 N^{10}+575 N^9+1124 N^8\nonumber \\&\quad -334 N^7-1505 N^6+3755 N^5\nonumber \\&\quad +4926 N^4+36N^3-472 N^2\nonumber \\&\quad -2160 N-864\end{aligned}$$

395 $$\begin{aligned} P_{311}&=149 N^{10}+793 N^9+2368 N^8\nonumber \\&\quad +5026 N^7+6853 N^6+6277 N^5\nonumber \\&\quad +5062 N^4+3168N^3+1296 N^2\nonumber \\&\quad +400 N+96\end{aligned}$$

396 $$\begin{aligned} P_{312}&=170 N^{10}+883 N^9\nonumber \\&\quad +1897 N^8+2710 N^7-448 N^6\nonumber \\&\quad -4745 N^5+561 N^4+5904N^3\nonumber \\&\quad +1132 N^2-2016 N-864\end{aligned}$$

397 $$\begin{aligned} P_{313}&=170 N^{10}+1213 N^9\nonumber \\&\quad +3091 N^8+2506 N^7-2692 N^6\nonumber \\&\quad -3047 N^5-861 N^4-2352 N^3\nonumber \\&\quad -5324 N^2-6240 N-2016\end{aligned}$$

398 $$\begin{aligned} P_{314}&=436 N^{10}+3960 N^9+15787 N^8\nonumber \\&\quad +36343 N^7 +46431 N^6+17745 N^5\nonumber \\&\quad -28270N^4-33648 N^3\nonumber \\&\quad -11056 N^2-1936 N+864\end{aligned}$$

399 $$\begin{aligned} P_{315}&=3 N^{11}+42 N^{10}+144 N^9\nonumber \\&\quad +74 N^8-459 N^7-1060 N^6\nonumber \\&\quad -1152 N^5-1424 N^4-1688 N^3\nonumber \\&\quad -1232 N^2-736 N-192\end{aligned}$$

400 $$\begin{aligned} P_{316}&=33 N^{11}+231 N^{10}+698 N^9\nonumber \\&\quad +1290 N^8+1513 N^7+1463 N^6\nonumber \\&\quad +2236 N^5+5096N^4+7328 N^3\nonumber \\&\quad +5456 N^2+3456 N+1152\end{aligned}$$

401 $$\begin{aligned} P_{317}&=95 N^{11}+853 N^{10}+3599 N^9\nonumber \\&\quad +9245 N^8+12320 N^7-282 N^6\nonumber \\&\quad -23342N^5-26920 N^4\nonumber \\&\quad -10832 N^3-1712 N^2-416 N-192\end{aligned}$$

402 $$\begin{aligned} P_{318}&=129 N^{11}+903 N^{10}+2894 N^9\nonumber \\&\quad +5730 N^8+6505 N^7+383 N^6-9464 N^5\nonumber \\&\quad -13912 N^4-11680 N^3\nonumber \\&\quad -6640 N^2-3648 N-1152\end{aligned}$$

403 $$\begin{aligned} P_{319}&=243 N^{11}+1701 N^{10}+5378 N^9\nonumber \\&\quad +10350 N^8+11479 N^7+1193 N^6\nonumber \\&\quad -14684 N^5-20572 N^4\nonumber \\&\quad -16288 N^3-8944 N^2-4992 N-1728\end{aligned}$$

404 $$\begin{aligned} P_{320}&=333 N^{11}+2331 N^{10}+6556 N^9\nonumber \\&\quad +9270 N^8+5081 N^7-6701 N^6\nonumber \\&\quad -17554 N^5-20036 N^4\nonumber \\&\quad -15680 N^3-9200 N^2-5664 N-1728\end{aligned}$$

405 $$\begin{aligned} P_{321}&=2 N^{12}+20 N^{11}+86 N^{10}\nonumber \\&\quad +192 N^9+199 N^8-N^7-297 N^6\nonumber \\&\quad -495 N^5-514 N^4-488 N^3\nonumber \\&\quad -416 N^2-176 N-32\end{aligned}$$

406 $$\begin{aligned} P_{322}&=23 N^{12}+138 N^{11}-311 N^{10}\nonumber \\&\quad -3148 N^9-7605 N^8-8462 N^7\nonumber \\&\quad -4163 N^6+246 N^5+1540 N^4\nonumber \\&\quad +1066 N^3+444 N^2+120 N+16\end{aligned}$$

407 $$\begin{aligned} P_{323}&=111 N^{12}+1035 N^{11}\nonumber \\&\quad +3634 N^{10}+5168 N^9-2662 N^8\nonumber \\&\quad -21724 N^7-37157 N^6-34963 N^5\nonumber \\&\quad -19122 N^4-4560 N^3\nonumber \\&\quad +80 N^2+1008 N+288\end{aligned}$$

408 $$\begin{aligned} P_{324}&=201 N^{12}+1845 N^{11}\nonumber \\&\quad +6742 N^{10}+11990 N^9\nonumber \\&\quad +7139 N^8-8917 N^7-15710 N^6\nonumber \\&\quad -2110 N^5+16644 N^4+22080 N^3\nonumber \\&\quad +12416 N^2+4128 N+576\end{aligned}$$

409 $$\begin{aligned} P_{325}&=7299 N^{12}+53973 N^{11}\nonumber \\&\quad +206656 N^{10}+532170 N^9\nonumber \\&\quad +820775 N^8+650149 N^7\nonumber \\&\quad +204230 N^6+189820 N^5\nonumber \\&\quad +606016 N^4+664624 N^3\nonumber \\&\quad +372192 N^2+143424 N+27648\end{aligned}$$

410 $$\begin{aligned} P_{326}&=9 N^{13}+72 N^{12}+101 N^{11}\nonumber \\&\quad -511 N^{10}-2325 N^9-4428 N^8\nonumber \\&\quad -4619 N^7-3841 N^6-4462 N^5-6012 N^4\nonumber \\&\quad -6992 N^3-5296 N^2-2592 N-576\end{aligned}$$

411 $$\begin{aligned} P_{327}&=69 N^{13}+420 N^{12}+794 N^{11}\nonumber \\&\quad -1501 N^{10}-11265 N^9\nonumber \\&\quad -18414 N^8-4436 N^7-5017 N^6\nonumber \\&\quad -41818 N^5-65616 N^4-62960 N^3\nonumber \\&\quad -39184 N^2-17184 N-3456\end{aligned}$$

412 $$\begin{aligned} P_{328}&=296 N^{13}+2368 N^{12}\nonumber \\&\quad +9908 N^{11}+22254 N^{10}\nonumber \\&\quad +13564 N^9-31716 N^8-71723 N^7\nonumber \\&\quad -71221 N^6-44369 N^5-33249 N^4\nonumber \\&\quad -26584 N^3+4968 N^2+7344 N+432\end{aligned}$$

413 $$\begin{aligned} P_{329}&=385 N^{14}+2567 N^{13}\nonumber \\&\quad +6877 N^{12}+9235 N^{11}\nonumber \\&\quad +5375 N^{10}-1207 N^9-3313 N^8\nonumber \\&\quad +905 N^7 +3876 N^6+1676 N^5+256 N^4\nonumber \\&\quad +1544 N^3+1616 N^2+736 N+192\end{aligned}$$

414 $$\begin{aligned} P_{330}&=531 N^{14}+5454 N^{13}\nonumber \\&\quad +25877 N^{12}+77604 N^{11}\nonumber \\&\quad +159437 N^{10}+205070 N^9+82971 N^8\nonumber \\&\quad -207408 N^7-490544 N^6-694320 N^5\nonumber \\&\quad -735104 N^4-562304 N^3\nonumber \\&\quad -355584 N^2-158976 N -34560\end{aligned}$$

415 $$\begin{aligned} P_{331}&=1773 N^{14}+18018 N^{13}\nonumber \\&\quad +80795 N^{12}+214620 N^{11}\nonumber \\&\quad +371423N^{10}+398930 N^9+154773 N^8\nonumber \\&\quad -228072 N^7-435356 N^6-492936 N^5\nonumber \\&\quad -534656 N^4-453440 N^3-299712 N^2\nonumber \\&\quad -144000 N-34560\end{aligned}$$

416 $$\begin{aligned} P_{332}&=4 N^{15}+50 N^{14}+267 N^{13}\nonumber \\&\quad +765 N^{12}+1183 N^{11}+682 N^{10}\nonumber \\&\quad -826 N^9-1858 N^8-1116 N^7+457 N^6\nonumber \\&\quad +1500 N^5+2268 N^4+2400 N^3\nonumber \\&\quad +1392 N^2+448 N+64\end{aligned}$$

417 $$\begin{aligned} P_{333}&=26 N^{15}+314 N^{14}\nonumber \\&\quad +1503 N^{13}+3222 N^{12}\nonumber \\&\quad +2510 N^{11}+1996 N^{10}\nonumber \\&\quad +15041 N^9+40728 N^8+54008 N^7\nonumber \\&\quad +44956 N^6+31936 N^5+30416 N^4\nonumber \\&\quad +29568 N^3+16704 N^2+5376 N+768\end{aligned}$$

418 $$\begin{aligned} P_{334}&=28 N^{15}+335 N^{14}+1953 N^{13}\nonumber \\&\quad +6497 N^{12}+11508 N^{11}+6624 N^{10}\nonumber \\&\quad -11753 N^9-27541 N^8 -33352 N^7-40915 N^6\nonumber \\&\quad -40468 N^5-16628 N^4+7584 N^3+10416 N^2\nonumber \\&\quad +4032 N+576\end{aligned}$$

419 $$\begin{aligned} P_{335}&=435 N^{15}+5436 N^{14}+32317 N^{13}\nonumber \\&\quad +119006 N^{12}+307057 N^{11}+620328 N^{10}\nonumber \\&\quad +1065977 N^9+1575060 N^8+1889534 N^7\nonumber \\&\quad +1704634 N^6+1113248 N^5+592440 N^4\nonumber \\&\quad +328672 N^3+165984 N^2+59904 N+10368\end{aligned}$$

420 $$\begin{aligned} P_{336}&=939 N^{16}+10527 N^{15}+47251 N^{14}\nonumber \\&\quad +101719 N^{13}+66350 N^{12}-155710 N^{11}\nonumber \\&\quad -322813 N^{10}-16829 N^9+702425 N^8\nonumber \\&\quad +1332497 N^7+1596952 N^6+1640548 N^5\nonumber \\&\quad +1506496 N^4+1099952 N^3+604032 N^2\nonumber \\&\quad +211392 N+34560 \end{aligned}$$

421 $$\begin{aligned} P_{337}&=1623 N^{16}+20963 N^{15}+119399 N^{14}\nonumber \\&\quad +394315 N^{13}+831483 N^{12}+1160715 N^{11}\nonumber \\&\quad +1086519 N^{10}+712841 N^9+425270 N^8\nonumber \\&\quad +337718 N^7+207634 N^6-73752 N^5\nonumber \\&\quad -261272 N^4-200160 N^3-64672 N^2\nonumber \\&\quad -4480 N+1408\end{aligned}$$

422 $$\begin{aligned} P_{338}&=87 N^{17}+1099 N^{16}+6055 N^{15}\nonumber \\&\quad +19019 N^{14}+37119 N^{13}+45159 N^{12}\nonumber \\&\quad +29583 N^{11}-2639 N^{10}-30218 N^9\nonumber \\&\quad -40778 N^8-39994 N^7-35844 N^6\nonumber \\&\quad -30808 N^5-30384 N^4-28256 N^3\nonumber \\&\quad -16064 N^2 -5248 N-768. \end{aligned}$$

The operator matrix element $A_{gg,Q}$ except for the term $a_{gg,Q}^{(3),0}$ is given by:

$$\begin{aligned} A_{gg,Q}&= \tfrac{1}{2}[1 + (-1)^N] \times \Biggl \{1 + {a_s} \frac{4}{3} {T_F} L_M + {a_s^2} \Biggl \{\frac{16}{9} {T_F^2} L_M^2\nonumber \\&\quad + {C_A T_F} \Biggl [ \Biggl [ \frac{16 \big (N^2+N+1\big )}{3 (N-1) N (N+1) (N+2)}-\frac{8 S_1}{3} \Biggr ] L_M^2\nonumber \\&\quad +\Biggl [\frac{16 P_{341}}{9 (N-1) N^2 (N+1)^2 (N+2)}-\frac{80 S_1}{9}\Biggr ] L_M \nonumber \\&\quad +\frac{2 P_{359}}{27 (N-1) N^3 (N+1)^3 (N+2)} -\frac{4 (56 N+47) S_1}{27 (N+1)}\Biggr ] \nonumber \\&\quad + {C_F T_F} \Bigg [\frac{4 \big (N^2+N+2\big )^2 }{(N-1) N^2 (N+1)^2 (N+2)} L_M^2 \nonumber \\&\quad +\frac{4 P_{353}}{(N-1) N^3 (N+1)^3 (N+2)} L_M-\frac{P_{369}}{(N-1) N^4 (N+1)^4 (N+2)} \Biggr ] \Biggr \} \nonumber \\&\quad + {a_s^3} \Biggl \{ \Biggl [ {T_F^3} \Biggl [ \frac{64}{27} L_M^3 -\frac{64 \zeta _3}{27} \Biggr ] \nonumber \\&\quad + {C_A T_F^2} \Biggl [ \Biggl [ \frac{448 \big (N^2+N+1\big )}{27 (N-1) N (N+1) (N+2)}-\frac{224 S_1}{27} \Biggr ] L_M^3 \nonumber \\&\quad +\Biggl [ \frac{8 P_{349}}{27 (N-1) N^2 (N+1)^2 (N+2)}-\frac{640 S_1}{27} \Biggr ] L_M^2 \nonumber \\&\quad +\Biggl [ -\frac{2 P_{362}}{27 (N\!-\!1) N^3 (N\!+\!1)^3 (N\!+\!2)} -\frac{8 P_{348} S_1}{9 (N\!-\!1) N^2 (N\!+\!1)^2 (N+2)}\Biggr ] L_M \nonumber \\&\quad +\frac{8 S_1^2}{3 (N+1)}-\frac{4}{27} \frac{P_{350} \zeta _2}{(N-1) N^2 (N+1)^2 (N+2)}\nonumber \\&\quad -\frac{8 P_{368}}{81 (N-1) N^4 (N+1)^4 (N+2)} \nonumber \\ \end{aligned}$$

$$\begin{aligned}&\quad +\frac{16 \big (328 N^4+256 N^3-247 N^2-175 N+54\big ) S_1}{81 (N-1) N (N+1)^2} \nonumber \\&\quad -\frac{448}{27} \frac{\big (N^2+N+1\big ) \zeta _3}{(N-1) N (N+1) (N+2)} -\frac{8 (2 N+1) S_2}{3 (N+1)} \nonumber \\&\quad +\frac{560}{27} S_1 \zeta _2 +\frac{224}{27} S_1 \zeta _3 \nonumber \\&\quad + {N_F} \Biggl [ \Biggl [ \frac{128 \big (N^2+N+1\big )}{27 (N-1) N (N+1) (N+2)}-\frac{64 S_1}{27}\Biggr ] L_M^3 \nonumber \\&\quad +\Biggl [ -\frac{4 P_{365}}{81 (N-1) N^3 (N+1)^3 (N+2)}\nonumber \\&\quad -\frac{16 P_{351} S_1}{81 (N-1) N^2 (N+1)^2 (N+2)}\Biggr ] L_M +\frac{16 S_1^2}{9 (N+1)} \nonumber \\&\quad -\frac{4}{27} \frac{\zeta _2 P_{345}}{(N-1) N^2 (N+1)^2 (N+2)} \nonumber \\&\quad +\frac{32 P_{373}}{243 (N-1) N^4 (N+1)^4 (N+2)}\nonumber \\&\quad +\frac{32 \big (328 N^4+256 N^3-247 N^2-175 N+54\big ) S_1}{243 (N-1) N (N+1)^2} \nonumber \\&\quad -\frac{128}{27} \frac{\big (N^2+N+1\big )\zeta _3}{(N-1) N (N+1) (N+2)} -\frac{16 (2 N+1) S_2}{9 (N+1)} \nonumber \\&\quad +\frac{160}{27} S_1 \zeta _2 +\frac{64}{27} S_1 \zeta _3 \Biggr ] \Biggr ] \nonumber \\&\quad + {C_A^2 T_F} \Biggl [ \Biggl [ \frac{176 S_1}{27} -\frac{352 \big (N^2+N+1\big )}{27 (N-1) N (N+1) (N+2)} \Biggr ] L_M^3 \nonumber \\&\quad +\Biggl [ -\frac{2 P_{377}}{9 (N-1)^2 N^3 (N+1)^3 (N+2)^3} \nonumber \\&\quad -\frac{8 P_{360} S_1}{9 (N-1)^2 N^2 (N+1)^2 (N+2)^2} +\frac{64}{3} S_{-2} S_1 \nonumber \\ \end{aligned}$$

$$\begin{aligned}&\quad +\frac{64}{3} S_1 S_2 +\frac{\big (N^2+N+1\big ) \Bigl [-\frac{128}{3} S_2-\frac{128}{3} S_{-2}\Bigr ]}{(N-1) N (N+1) (N+2)}\nonumber \\&\quad +\frac{32 S_3}{3} +\frac{32}{3} S_{-3}-\frac{64}{3} S_{-2,1} \Biggr ] L_M^2 \nonumber \\&\quad +\Biggl [ \frac{32}{3} S_{-2}^2 -\frac{16 P_{364} S_{-2}}{9 (N-1)^2 N^3 (N+1)^3 (N+2)^2} \nonumber \\&\quad +\frac{32 P_{347} S_1 S_{-2}}{9 (N-1) N^2 (N+1)^2 (N+2)} \nonumber \\&\quad +64 \frac{\big (2 N^4+4 N^3+7 N^2+5 N+6\big ) \zeta _3}{(N-1) N^2 (N+1)^2 (N+2)} \nonumber \\&\quad +\frac{P_{387}}{81 (N-1)^2 N^4 (N+1)^4 (N+2)^3}\nonumber \\&\quad -\frac{4 P_{382} S_1}{81 (N-1)^2 N^4 (N+1)^4 (N+2)^2} + \Biggl [\frac{640 S_2}{9}-\frac{32 S_3}{3}\Biggr ] S_1\nonumber \\&\quad +\frac{16 P_{340} S_2}{9 (N-1) N^2 (N+1)^2 (N+2)} \nonumber \\&\quad +\frac{8 \big (40 N^4+80 N^3+73 N^2+33 N+54\big ) S_3}{9 N^2 (N+1)^2} -64 S_1 \zeta _3 \nonumber \\&\quad +\frac{P_{346} \big (\frac{16}{9} S_{-3}-\frac{32}{9} S_{-2,1}\big )}{(N-1) N^2 (N+1)^2 (N+2)} \Biggr ] L_M\nonumber \\&\quad -\frac{44 S_1^2}{9 (N+1)} -\frac{8 P_{376}}{243 (N-1) N^4 (N+1)^4 (N+2)}\nonumber \\&\quad +\frac{4}{27} \frac{P_{378} \zeta _2}{(N-1)^2 N^3 (N+1)^3 (N+2)^3}\nonumber \\&\quad -\frac{8 \big (2834 N^4+2042 N^3-1943 N^2-1151 N+594\big ) S_1}{243 (N-1) N (N+1)^2}\nonumber \\&\quad +\frac{16}{27} \frac{P_{352} S_1 \zeta _2}{(N-1)^2 N^2 (N+1)^2 (N+2)^2} +\frac{44 (2 N+1) S_2}{9 (N+1)} \nonumber \\&\quad +\Bigl [-\frac{32}{3} S_1 S_2-\frac{32}{3} S_{-2} S_1-\frac{16 S_3}{3}\nonumber \\&\quad -\frac{16}{3} S_{-3}+\frac{32}{3} S_{-2,1}\Bigr ]\zeta _2 -\frac{176}{27} S_1 \zeta _3 \nonumber \\ \end{aligned}$$

$$\begin{aligned}&\quad +\frac{\big (N^2+N+1\big ) \big (\big (\frac{64 S_2}{3}+\frac{64}{3} S_{-2}\big ) \zeta _2+\frac{352 \zeta _3}{27}\big )}{(N-1) N (N+1) (N+2)}\Biggr ] \nonumber \\&\quad + {C_F^2 T_F} \Biggl [ \Biggl [\frac{16 \big (N^2+N+2\big )^2 S_1}{3 (N-1) N^2 (N+1)^2(N+2)} \nonumber \\&\quad -\frac{4 \big (N^2+N+2\big )^2 \big (3 N^2+3 N+2\big )}{3 (N-1) N^3 (N+1)^3 (N+2)} \Biggr ] L_M^3\nonumber \\&\quad +\Biggl [\frac{8 \big (5 N^2+N-2\big ) S_1 \big (N^2+N+2\big )^2}{(N-1) N^3 (N+1)^3 (N+2)}-\frac{16 S_2 \big (N^2+N+2\big )^2}{(N-1) N^2 (N+1)^2 (N+2)} \nonumber \\&\quad -\frac{4 P_{344} \big (N^2+N+2\big )}{(N-1) N^4 (N+1)^4 (N+2)} \Biggr ] L_M^2 \nonumber \\&\quad +\Biggl [ \frac{\big (-\frac{8}{3} S_1^3+24 S_2 S_1-32 S_{2,1}\big ) \big (N^2+N+2\big )^2}{(N-1) N^2 (N+1)^2 (N+2)}\nonumber \\&\quad +\frac{4 \big (5 N^3+4 N^2+9 N+6\big ) S_1^2 \big (N^2+N+2\big )}{(N-1) N^2 (N+1)^3 (N+2)} \nonumber \\&\quad +\frac{16 \big (5 N^2+5 N-14\big ) S_3 \big (N^2+N+2\big )}{3 (N-1) N^2 (N+1)^2 (N+2)}\nonumber \\&\quad -\frac{4 \big (17 N^4+48 N^3+69 N^2+10 N-8\big ) S_2 \big (N^2+N+2\big )}{(N-1) N^3 (N+1)^3 (N+2)} \nonumber \\&\quad -\frac{96 \zeta _3 \big (N^2+N+2\big )}{N^2 (N+1)^2}\nonumber \\&\quad +\frac{\big (-256 S_1 S_{-2}-128 S_{-3}+256 S_{-2,1}\big ) \big (N^2+N+2\big )}{(N-1) N^2 (N+1)^2 (N+2)}\nonumber \\&\quad -\frac{2 P_{379}}{(N-1) N^5 (N+1)^5 (N+2)}\nonumber \\&\quad -\frac{8 P_{356} S_1}{(N-1) N^4 (N+1)^4 (N+2)} \nonumber \\&\quad -\frac{32 \big (N^6+3 N^5+N^4-3 N^3-26 N^2-24 N-16\big ) S_{-2}}{(N-1) N^3 (N+1)^3 (N+2)}\Biggr ] L_M \nonumber \\&\quad +\frac{4 \big (N^2+N+2\big ) \big (N^4-5 N^3-32 N^2-18 N-4\big ) S_1^2}{(N-1) N^3 (N+1)^3 (N+2)}\nonumber \\ \end{aligned}$$

$$\begin{aligned}&\quad -\frac{4}{3} \frac{P_{361} \zeta _3}{(N-1) N^3 (N+1)^3 (N+2)} \nonumber \\&\quad -2 \frac{P_{372} \zeta _2}{(N-1) N^4 (N+1)^4 (N+2)} \nonumber \\&\quad -\frac{8 \big (N^2+N+2\big ) \big (2 N^5-2 N^4-11 N^3-19 N^2-44 N-12\big ) S_1}{(N-1) N^3 (N+1)^4 (N+2)}\nonumber \\&\quad -\frac{P_{388}}{(N-1) N^6 (N+1)^6 (N+2)} \nonumber \\&\quad -4 \frac{\big (N^2+N+2\big ) \big (5 N^4+4 N^3+N^2-10 N-8\big ) \zeta _2}{(N-1) N^3 (N+1)^3 (N+2)} S_1 \nonumber \\&\quad +\frac{4 \big (N^2+N+2\big ) P_{342} S_2}{(N-1) N^4 (N+1)^4 (N+2)}\nonumber \\&\quad +\frac{(3 N+2) \big (N^2+N+2\big ) \big (\frac{8}{3} S_1^3+8 S_2 S_1\big )}{(N-1) N^3 (N+1) (N+2)} +128 \log (2) \zeta _2 \nonumber \\&\quad +\frac{8 \big (N^2+N+2\big ) \big (3 N^4+48 N^3+43 N^2-22 N-8\big ) S_3}{3 (N-1) N^3 (N+1)^3 (N+2)}\nonumber \\&\quad -\frac{32 \big (N^2-3 N-2\big ) \big (N^2+N+2\big ) S_{2,1}}{(N-1) N^3 (N+1)^2 (N+2)}\nonumber \\&\quad +\frac{\big (N^2+N+2\big )^2}{(N-1) N^2 (N+1)^2 (N+2)} \Bigg [-\frac{2}{3} S_1^4-4 S_2 S_1^2 \nonumber \\&\quad +\Bigl [-\frac{16}{3} S_3-32 S_{2,1}\Bigr ] S_1-\frac{16}{3} \zeta _3 S_1-2 S_2^2+12 S_4\nonumber \\&\quad -32 S_{3,1} +64 S_{2,1,1}+\Bigl [12 S_2-4 S_1^2\Bigr ] \zeta _2\Bigr ] \Biggr ]\nonumber \\&\quad + {C_F T_F^2} \Biggl [ \frac{80 \big (N^2+N+2\big )^2}{9 (N-1) N^2 (N+1)^2 (N+2)} L_M^3 \nonumber \\ \end{aligned}$$

$$\begin{aligned}&\quad +\Biggl [\frac{32 \big (N^2+N+2\big )^2 S_1}{3 (N-1) N^2 (N+1)^2 (N+2)} +\frac{8 P_{357}}{9 (N-1) N^3 (N+1)^3 (N+2)} \Biggr ] L_M^2 \nonumber \\&\quad +\Biggl [\frac{\big (\frac{16}{3} S_1^2-16 S_2\big ) \big (N^2+N+2\big )^2}{(N-1) N^2 (N+1)^2 (N+2)} -\frac{8 P_{375}}{27 (N-1) N^4 (N+1)^4 (N+2)} \nonumber \\&\quad +\frac{32 P_{343} S_1}{9 (N-1) N^3 (N+1)^3 (N+2)}\Biggr ] L_M \nonumber \\&\quad -\frac{8}{9} \frac{P_{358} \zeta _2}{(N-1) N^3 (N+1)^3 (N+2)}+\frac{2 P_{383}}{9 (N-1) N^5 (N+1)^5 (N+2)}\nonumber \\&\quad + {N_F} \Biggl [ \frac{64 \big (N^2+N+2\big )^2}{9(N-1) N^2 (N+1)^2 (N+2)} L_M^3 \nonumber \\&\quad +\Biggl [\frac{\Bigl [16 S_1^2-\frac{80 S_2}{3}\Bigr ] \big (N^2+N+2\big )^2}{(N-1) N^2 (N+1)^2 (N+2)}-\frac{4 P_{374}}{9 (N-1) N^4 (N+1)^4 (N+2)} \nonumber \\&\quad -\frac{32 \big (5 N^5+52 N^4+109 N^3+90 N^2+48 N+16\big ) S_1}{3 (N-1) N^3 (N+1)^3 (N+2)}\Biggr ] L_M \nonumber \\&\quad +\frac{4}{9} \frac{P_{363} \zeta _2}{(N-1) N^3 (N+1)^3 (N+2)} \nonumber \\&\quad -\frac{16 \big (N^2+N+2\big ) \big (8 N^3+13 N^2+27 N+16\big ) \big (S_1^2+ S_2 \big )}{9 (N-1) N^2 (N+1)^3 (N+2)} \nonumber \\&\quad +\frac{2 P_{384}}{81 (N-1) N^5 (N+1)^5 (N+2)} \nonumber \\&\quad +\frac{32 \big (N^2+N+2\big ) \big (43 N^4+105 N^3+224 N^2+230 N+86\big ) S_1}{27 (N-1) N^2 (N+1)^4 (N+2)}\nonumber \\&\quad +\frac{\big (N^2+N+2\big )^2}{(N-1) N^2 (N+1)^2 (N+2)} \Bigr [\frac{16}{9} S_1^3 \nonumber \\&\quad +\frac{16}{3} S_2 S_1+\frac{16}{3} \zeta _2 S_1+\frac{32 S_3}{9}-\frac{64 \zeta _3}{9}\Bigl ] \Biggr ]\nonumber \\ \end{aligned}$$

$$\begin{aligned}&\quad + \frac{\big (N^2+N+2\big )^2 \big (-\frac{16}{3} S_1 \zeta _2-\frac{80 \zeta _3}{9}\big )}{(N-1) N^2 (N+1)^2 (N+2)}\Biggr ]\nonumber \\&\quad + {C_A C_F T_F} \Biggl [ -\frac{8 \big (N^2+N+2\big ) \big (N^3+8 N^2+11 N+2\big ) S_1^3}{3 (N-1) N^2 (N+1)^3 (N+2)^2} \nonumber \\&\quad -\frac{4 \big (N^2+N+2\big ) P_{339} S_1^2}{(N-1) N^2 (N+1)^4 (N+2)^3} -\frac{4}{3} \frac{P_{371} \zeta _2}{(N-1)^2 N^3 (N+1)^3 (N+2)^2} S_1 \nonumber \\&\quad -\frac{2 P_{381} S_1}{9 (N-1) N^2 (N+1)^5 (N+2)^4}\nonumber \\&\quad +\frac{8 \big (N^2+N+2\big ) \big (3 N^3-12 N^2-27 N-2\big ) S_2 S_1}{(N-1) N^2 (N+1)^3 (N+2)^2} \nonumber \\&\quad +\frac{8}{9} \frac{\zeta _3}{(N-1)^2 N^3 (N+1)^3 (N+2)^2} P_{370} \nonumber \\&\quad +\frac{4}{9} \frac{P_{385}\zeta _2}{(N-1)^2 N^4 (N+1)^4 (N+2)^3}-\frac{P_{390}}{18 (N-1)^2 N^6 (N+1)^6 (N+2)^5}\nonumber \\&\quad +L_M^3 \Biggl [ -\frac{8 \big (11 N^4+22 N^3-23 N^2-34 N-12\big ) \big (N^2+N+2\big )^2}{9 (N-1)^2 N^3 (N+1)^3 (N+2)^2}\nonumber \\&\quad -\frac{16 S_1 \big (N^2+N+2\big )^2}{3 (N-1) N^2 (N+1)^2 (N+2)} \Biggr ] \nonumber \\&\quad -\frac{4 \big (N^2+N+2\big ) P_{366} S_2}{(N-1)^2 N^4 (N+1)^4 (N+2)^3} \nonumber \\&\quad -\frac{64 \big (N^2+N+2\big ) \big (N^5+10 N^4+9 N^3+3 N^2+7 N+6\big ) S_3}{3 (N-1)^2 N^3 (N+1)^3 (N+2)^2} \nonumber \nonumber \\ \end{aligned}$$

$$\begin{aligned}&\quad +L_M^2 \Biggl [ \frac{\Bigl [-16 S_2-32 S_{-2}\Bigr ] \big (N^2+N+2\big )^2}{(N-1) N^2 (N+1)^2 (N+2)} \nonumber \\&\quad -\frac{2 P_{386}}{9 (N-1)^2 N^4 (N+1)^4 (N+2)^3}\nonumber \\&\quad -\frac{8 P_{367} S_1}{3 (N-1)^2 N^3 (N+1)^3 (N+2)^2}\Biggr ]-64 \log (2) \zeta _2 \nonumber \nonumber \\&+\frac{\big (N^2{+}N{+}2\big ) \big (N^4{+}2 N^3{+}7 N^2{+}22 N{+}20\big ) \Bigl [-32 ({-}1)^N S_{-2}-16 (-1)^N \zeta _2\Bigr ]}{(N-1) N (N{+}1)^4 (N{+}2)^3}\nonumber \nonumber \\&{+}\frac{\big (N^2-N-4\big ) \big (N^2{+}N{+}2\big )}{(N-1) N (N{+}1)^3 (N{+}2)^2} \Bigl [64 ({-}1)^N S_1 S_{-2}+32 (-1)^N S_{-3}\nonumber \nonumber \\&\quad -64 S_{-2,1}+32 (-1)^N S_1 \zeta _2 +24 (-1)^N \zeta _3\Bigr ]\nonumber \nonumber \\&\quad +\frac{\big (N^2+N+2\big )^2}{(N-1) N^2 (N+1)^2 (N+2)} \Bigl [\frac{2}{3} S_1^4 +20 S_2 S_1^2+32 (-1)^N S_{-3} S_1 \nonumber \nonumber \\&\quad +\Bigl [\frac{160 S_3}{3}-64 S_{-2,1}\Bigr ] S_1 +\frac{8}{3} \big (-7+9 (-1)^N\big ) \zeta _3 S_1 \nonumber \nonumber \\&\quad +2 S_2^2 +S_{-2} \Bigl [32 (-1)^N S_1^2+32 (-1)^N S_2\Bigr ]\nonumber \\&\quad +36 S_4+16 (-1)^N S_{-4}-16 S_{3,1}-32 S_{-2,2}-32 S_{-3,1}\nonumber \\&\quad -16 S_{2,1,1} +64 S_{-2,1,1} +\Bigl [ 4 \big (-3+4 (-1)^N\big ) S_1^2 \nonumber \\&\quad +4 \big ({-}1+4 (-1)^N\big ) S_2 +8 \big (1+2 (-1)^N\big ) S_{-2} \Bigr ] \zeta _2\Bigr ] \nonumber \\&\quad +L_M \Biggl [ \frac{\big (N^2+N+2\big )^2}{(N-1) N^2 (N+1)^2 (N+2)} \Bigl [ \frac{8}{3} S_1^3 -40 S_2 S_1 \nonumber \\ \end{aligned}$$

423 $$\begin{aligned}&\quad -32 (-1)^N S_{-2} S_1 -16 (-1)^N S_{-3} +32 S_{2,1} -12 (-1)^N \zeta _3 \Bigr ] \nonumber \\&\quad -\frac{16 \big (5 N^2+5 N-26\big ) S_3 \big (N^2+N+2\big )}{3 (N-1) N^2 (N+1)^2 (N+2)} \nonumber \\&\quad -\frac{4 \big (17 N^4-6 N^3+41 N^2-16 N-12\big ) S_1^2 \big (N^2+N+2\big )}{3 (N-1)^2 N^3 (N+1)^2 (N+2)} \nonumber \\&\quad +\frac{96 \big (N^2+N+4\big ) S_{-3} \big (N^2+N+2\big )}{(N-1) N^2 (N+1)^2 (N+2)}\nonumber \\&\quad -\frac{32 \big (N^2+N+14\big ) S_{-2,1} \big (N^2+N+2\big )}{(N-1) N^2 (N+1)^2 (N+2)} \nonumber \\&\quad -\frac{\big (N^2-N-4\big ) 32 (-1)^N S_{-2} \big (N^2+N+2\big )}{(N-1) N (N+1)^3 (N+2)^2}\nonumber \\&\quad +\frac{8 P_{389}}{27 (N-1)^2 N^5 (N+1)^5 (N+2)^4} \nonumber \\&\quad - \frac{4 \big (N^2+N+10\big ) \big (5 N^2+5 N+18\big ) \zeta _3}{(N-1) N^2 (N+1)^2 (N\!+\!2)}\!-\!\frac{8 P_{380} S_1}{9 (N\!-\!1)^2 N^4 (N\!+\!1)^4 (N+2)^2}\nonumber \\&\quad +\frac{64 \big (N^4+2 N^3+7 N^2+6 N+16\big ) S_{-2} S_1}{(N-1) N^2 (N+1)^2 (N+2)}\nonumber \\&\quad -\frac{4 P_{354} S_2}{(N-1)^2 N^3 (N+1)^3 (N+2)^2}-\frac{32 P_{355} S_{-2}}{(N-1)^2 N^3 (N+1)^3 (N+2)^2}\nonumber \\&\quad +64 S_1 \zeta _3 \Biggr ] \Biggr ] \Biggr ] + a_{gg,Q}^{(3),0} \Biggr \} \Biggr \}, \end{aligned}$$

with

424 $$\begin{aligned} P_{339}&=N^6+6 N^5+7 N^4+4 N^3+18 N^2\nonumber \\&\quad +16 N-8\end{aligned}$$

425 $$\begin{aligned} P_{340}&=3 N^6+9 N^5-113 N^4-241 N^3-274 N^2\nonumber \\&\quad -152 N-24 \end{aligned}$$

426 $$\begin{aligned} P_{341}&=3 N^6+9 N^5+22 N^4+29 N^3+41 N^2\nonumber \\&\quad +28 N+6 \end{aligned}$$

427 $$\begin{aligned} P_{342}&=3 N^6+30 N^5+15 N^4-64 N^3-56 N^2\nonumber \\&\quad -20 N-8 \end{aligned}$$

428 $$\begin{aligned} P_{343}&=4 N^6+3 N^5-50 N^4-129 N^3-100 N^2\nonumber \\&\quad -56 N-24 \end{aligned}$$

429 $$\begin{aligned} P_{344}&=7 N^6+15 N^5+7 N^4-23 N^3-26 N^2\nonumber \\&\quad -20 N-8 \end{aligned}$$

430 $$\begin{aligned} P_{345}&=9 N^6+27 N^5+161 N^4+277 N^3+358 N^2\nonumber \\&\quad +224 N+48\end{aligned}$$

431 $$\begin{aligned} P_{346}&=20 N^6+60 N^5+11 N^4-78 N^3-121 N^2\nonumber \\&\quad -72 N-108 \end{aligned}$$

432 $$\begin{aligned} P_{347}&=20 N^6+60 N^5+11 N^4-78 N^3-85 N^2\nonumber \\&\quad -36 N-108 \end{aligned}$$

433 $$\begin{aligned} P_{348}&=40 N^6+114 N^5+19 N^4-132 N^3-147 N^2\nonumber \\&\quad -70 N-32 \end{aligned}$$

434 $$\begin{aligned} P_{349}&=63 N^6+189 N^5+367 N^4+419 N^3+626 N^2\nonumber \\&\quad +448 N+96\end{aligned}$$

435 $$\begin{aligned} P_{350}&=99 N^6+297 N^5+631 N^4+767 N^3+1118 N^2\nonumber \\&\quad +784 N+168\end{aligned}$$

436 $$\begin{aligned} P_{351}&=136 N^6+390 N^5+19 N^4-552 N^3-947 N^2\nonumber \\&\quad -630 N-288\end{aligned}$$

437 $$\begin{aligned} P_{352}&=N^8+4 N^7+2 N^6+64 N^5+173 N^4+292 N^3\nonumber \\&\quad +256 N^2-72 N-72\end{aligned}$$

438 $$\begin{aligned} P_{353}&=N^8+4 N^7+8 N^6+6 N^5-3 N^4-22 N^3\nonumber \\&\quad -10 N^2-8 N-8 \end{aligned}$$

439 $$\begin{aligned} P_{354}&=3 N^8-14 N^7-164 N^6-454 N^5-527 N^4\nonumber \\&\quad -204 N^3-112 N^2+80 N+48 \end{aligned}$$

440 $$\begin{aligned} P_{355}&=3 N^8+10 N^7+13 N^6+N^5+28 N^4+81 N^3\nonumber \\&\quad +4 N^2-12 N-32 \end{aligned}$$

441 $$\begin{aligned} P_{356}&=3 N^8+23 N^7+51 N^6+95 N^5+142 N^4\nonumber \\&\quad +158 N^3+56 N^2-32 N-16\end{aligned}$$

442 $$\begin{aligned} P_{357}&=15 N^8+60 N^7+76 N^6-18 N^5-275 N^4\nonumber \\&\quad -546 N^3-400 N^2-224 N-96\end{aligned}$$

443 $$\begin{aligned} P_{358}&=15 N^8+60 N^7+86 N^6+12 N^5-166 N^4\nonumber \\&\quad -378 N^3-245 N^2-148 N-84\end{aligned}$$

444 $$\begin{aligned} P_{359}&=15 N^8+60 N^7+572 N^6+1470 N^5\nonumber \\&\quad +2135 N^4+1794 N^3+722 N^2-24 N-72\end{aligned}$$

445 $$\begin{aligned} P_{360}&=23 N^8+92 N^7+46 N^6-88 N^5+79 N^4\nonumber \\&\quad +476 N^3+428 N^2-96 N-96\end{aligned}$$

446 $$\begin{aligned} P_{361}&=24 N^8+96 N^7+93 N^6-57 N^5-143 N^4\nonumber \\&\quad -79 N^3-34 N^2-20 N-8 \end{aligned}$$

447 $$\begin{aligned} P_{362}&=27 N^8+108 N^7-1440 N^6-4554 N^5-5931 N^4\nonumber \\&\quad -3762 N^3-256 N^2+1184 N+480 \end{aligned}$$

448 $$\begin{aligned} P_{363}&=63 N^8+252 N^7+196 N^6-258 N^5-551 N^4\nonumber \\&\quad -282 N^3-220 N^2-80 N+48\end{aligned}$$

449 $$\begin{aligned} P_{364}&=131 N^8+524 N^7+691 N^6+239 N^5-848 N^4\nonumber \\&\quad -1483 N^3-586 N^2+108 N+360\end{aligned}$$

450 $$\begin{aligned} P_{365}&=297 N^8+1188 N^7+640 N^6-2094 N^5-1193 N^4\nonumber \\&\quad +2874 N^3+5008 N^2+3360 N+864\end{aligned}$$

451 $$\begin{aligned} P_{366}&=N^9+21 N^8+85 N^7+105 N^6+42 N^5+290 N^4\nonumber \\&\quad +600 N^3+456 N^2+256 N+64\end{aligned}$$

452 $$\begin{aligned} P_{367}&=3 N^{10}+15 N^9+35 N^8+50 N^7+91 N^6\nonumber \\&\quad +233 N^5+255 N^4+150 N^3-24 N^2-184 N-48\end{aligned}$$

453 $$\begin{aligned} P_{368}&=3 N^{10}+15 N^9+3316 N^8+12778 N^7\nonumber \\&\quad +22951 N^6+23815 N^5+14212 N^4+3556 N^3\nonumber \\&\quad -30 N^2+288 N+216 \end{aligned}$$

454 $$\begin{aligned} P_{369}&=15 N^{10}+75 N^9+112 N^8+14 N^7-61 N^6\nonumber \\&\quad +107 N^5+170 N^4+36 N^3-36 N^2-32 N-16\end{aligned}$$

455 $$\begin{aligned} P_{370}&=18 N^{10}+90 N^9+119 N^8-91 N^7\nonumber \\&\quad -167 N^6+101 N^5+162 N^4-72 N^3\nonumber \\&-504 N^2-184 N-48\end{aligned}$$

456 $$\begin{aligned} P_{371}&=30 N^{10}+150 N^9+163 N^8\nonumber \\&\quad -224 N^7-586 N^6-368 N^5-39 N^4\nonumber \\&\quad -78 N^3+144 N^2+184 N+48\end{aligned}$$

457 $$\begin{aligned} P_{372}&=40 N^{10}+200 N^9+282 N^8\nonumber \\&\quad -66 N^7-615 N^6-753 N^5-509 N^4\nonumber \\&\quad -205 N^3-2 N^2+68 N+24\end{aligned}$$

458 $$\begin{aligned} P_{373}&=63 N^{10}+315 N^9-1142 N^8\nonumber \\&\quad -6260 N^7-11927 N^6-12359 N^5\nonumber \\&\quad -7235 N^4-1778 N^3+15 N^2-144 N-108\end{aligned}$$

459 $$\begin{aligned} P_{374}&=67 N^{10}+335 N^9+368 N^8\nonumber \\&\quad -762 N^7-3349 N^6-6669 N^5-8310 N^4\nonumber \\&\quad -7656 N^3-4648 N^2-1600 N-288\end{aligned}$$

460 $$\begin{aligned} P_{375}&=219 N^{10}+1095 N^9+1640 N^8\nonumber \\&\quad -82 N^7-2467 N^6-2947 N^5-3242 N^4\nonumber \\&\quad -4326N^3-3466 N^2-1488 N-360\end{aligned}$$

461 $$\begin{aligned} P_{376}&=693 N^{10}+3465 N^9-11014 N^8\nonumber \\&\quad -62668 N^7-120361 N^6-125113 N^5\nonumber \\&\quad -73393 N^4-18010 N^3+165 N^2-1584 N-1188\end{aligned}$$

462 $$\begin{aligned} P_{377}&=3 N^{11}+21 N^{10}-124 N^9\nonumber \\&\quad -1014 N^8-2185 N^7-2099 N^6-934 N^5\nonumber \\&\quad -2060 N^4-4632 N^3-4256 N^2-2688 N-768\end{aligned}$$

463 $$\begin{aligned} P_{378}&=27 N^{11}+189 N^{10}+631 N^9+1356 N^8\nonumber \\&\quad +2155 N^7+2207 N^6+211 N^5-4984 N^4\nonumber \\&\quad -8400 N^3-5824 N^2-2544 N-576 \end{aligned}$$

464 $$\begin{aligned} P_{379}&=N^{12}+6 N^{11}-5 N^{10}-80 N^9\nonumber \\&\quad -379 N^8-846 N^7-1057 N^6-786 N^5\nonumber \\&\quad +84N^4+490 N^3+324 N^2+152 N+48\end{aligned}$$

465 $$\begin{aligned} P_{380}&=15 N^{12}+90 N^{11}+80 N^{10}\nonumber \\&\quad -452 N^9-1401 N^8-2298 N^7-5002 N^6\nonumber \\&\quad -6516 N^5-1116 N^4 +2960 N^3 \nonumber \\&\quad +3464 N^2+2400 N+864\end{aligned}$$

466 $$\begin{aligned} P_{381}&=233 N^{12}+2724 N^{11}+13349 N^{10}\nonumber \\&\quad +34680 N^9+46703 N^8+12096 N^7-69461 N^6\nonumber \\&\quad -137724 N^5-141176 N^4-91776 N^3\nonumber \\&\quad -34832 N^2-6336 N-2304\end{aligned}$$

467 $$\begin{aligned} P_{382}&=310 N^{12}+2058 N^{11}+5939 N^{10}\nonumber \\&\quad +17235 N^9+44700 N^8+93240 N^7+140861 N^6\nonumber \\&\quad +113169 N^5+14578 N^4-40374 N^3\nonumber \\&\quad -33372 N^2-12312 N-3888\end{aligned}$$

468 $$\begin{aligned} P_{383}&=391 N^{12}+2346 N^{11}+4795 N^{10}\nonumber \\&\quad +2758 N^9-2243 N^8+1150 N^7+7713 N^6\nonumber \\&\quad +4546 N^5-792 N^4+1224 N^2+864 N+288\end{aligned}$$

469 $$\begin{aligned} P_{384}&=1593 N^{12}+9558 N^{11}+15013 N^{10}\nonumber \\&\quad -8758 N^9-62269 N^8-82318 N^7-79041 N^6\nonumber \\&\quad -90898 N^5-70928 N^4-15872 N^3\nonumber \\&\quad +7344 N^2+5184 N+1728\end{aligned}$$

470 $$\begin{aligned} P_{385}&=15 N^{13}+120 N^{12}+530 N^{11}\nonumber \\&\quad +1562 N^{10}+2042 N^9-1680 N^8-9220 N^7\nonumber \\&\quad -12524 N^6-7911 N^5-5230 N^4-5880 N^3\nonumber \\&\quad -3344 N^2-2544 N-864\end{aligned}$$

471 $$\begin{aligned} P_{386}&=33 N^{13}+264 N^{12}+479 N^{11}\nonumber \\&\quad -1366 N^{10}-8809 N^9-23124 N^8\nonumber \\&\quad -34351 N^7-26198 N^6-3624 N^5 \nonumber \\&\quad +5240 N^4-2496 N^3-7232 N^2-7104 N-2304\end{aligned}$$

472 $$\begin{aligned} P_{387}&=2493 N^{13}+19944 N^{12}\nonumber \\&\quad +79295 N^{11}+208394 N^{10}+375431 N^9\nonumber \\&\quad +531516 N^8+623697 N^7+733338 N^6\nonumber \\&\quad +963340 N^5+1047352 N^4+895648 N^3\nonumber \\&\quad +559488 N^2+222336 N+41472\end{aligned}$$

473 $$\begin{aligned} P_{388}&=39 N^{14}+273 N^{13}+741 N^{12}\nonumber \\&\quad +1025 N^{11}+1343 N^{10}+3479 N^9\nonumber \\&\quad +6707 N^8+6555 N^7+2258 N^6-1520 N^5\nonumber \\&\quad -1944 N^4-532 N^3+280 N^2+208 N+32\end{aligned}$$

474 $$\begin{aligned} P_{389}&=276 N^{16}+3036 N^{15}+13660 N^{14}\nonumber \\&\quad +30172 N^{13}+22446 N^{12}-50653 N^{11}\nonumber \\&\quad -171627 N^{10}-246412 N^9-204934 N^8\nonumber \\&\quad -83791 N^7+28263 N^6+43144 N^5-33372 N^4\nonumber \\&\quad -82640 N^3-79152 N^2-47232 N -12096 \end{aligned}$$

475 $$\begin{aligned} P_{390}&=3135 N^{19}+43890 N^{18}+257636 N^{17}\nonumber \\&\quad +794084 N^{16}+1224418 N^{15}+244448 N^{14}\nonumber \\&\quad -2371724 N^{13}-3594388 N^{12}-792201 N^{11}\nonumber \\&\quad +2719198 N^{10}+2284064 N^9-85568 N^8\nonumber \\&\quad -227344 N^7 +952768 N^6 +1160704 N^5\nonumber \\&\quad +807552 N^4+574464 N^3+305664 N^2\nonumber \\&\quad +104448 N+18432. \end{aligned}$$

## Footnotes

1 The leading order contributions were given in Refs. [4–8].

2 Precise representations in Mellin space to $O(a_s^2)$ were derived in [3].

3 For higher order corrections to the gluonic contributions in the threshold region, cf. [36].

4 For the corresponding contributions in case of transversity see [40].

5 At 3-loop order, there are also contributions by graphs carrying heavy quark lines of different mass. These are dealt with elsewhere [87, 88].

6 For the heavy flavor corrections in case of $W^\pm$-boson exchange up to $O(\alpha _s^2)$ see [89–92].

7 Note that the heavy flavor threshold in the limit $Q^2 \gg m^2$ is again $x$ and not $x(1+4m^2/Q^2)$, which is the case retaining also power corrections.

8 For the VFNS in case of both the bottom and charm quarks transmuting into massless states, see [87, 88].

9 We have corrected some typographical errors in (13–15) in [44], in accordance with the appendix of Ref. [44].

10 For the algebraically reduced representations see [100].

11 For other implementations see [104].

12 Note that the kinematic region at small $x$ probed at HERA is limited to values $x \ge Q^2/(yS)$, with $S \simeq 10^5~\mathrm{GeV}^2$ and $y \in [0,1]$.

13 The expression in the parameterization given at http://www.liv.ac.uk/~avogt/ is correct, however.

## References

[1] Laenen E, Riemersma S, Smith J, van Neerven WL (1993). Nucl. Phys. B.

[2] S. Riemersma, J. Smith, W.L. van Neerven, Phys. Lett. B **347**, 143 (1995). hep-ph/9411431

[3] S.I. Alekhin, J. Blümlein, Phys. Lett. B **594**, 299 (2004). hep-ph/0404034

[4] Witten E (1976). Nucl. Phys. B.

[5] Babcock J, Sivers DW, Wolfram S (1978). Phys. Rev. D.

[6] Shifman MA, Vainshtein AI, Zakharov VI (1978). Nucl. Phys. B.

[7] Leveille JP, Weiler TJ (1979). Nucl. Phys. B.

[8] Glück M, Hoffmann E, Reya E (1982). Z. Phys. C.

[9] Aaron FD (2010). H1 and ZEUS collaborations. JHEP.

[10] S. Bethke et al., Workshop on precision measurements of $$\alpha _s$$. arXiv:1110.0016 [hep-ph]

[11] Alekhin S, Blümlein J, Klein S, Moch S (2010). Phys. Rev. D.

[12] Alekhin S, Blümlein J, Moch S (2012). Phys. Rev. D.

[13] J. Blümlein, H. Böttcher, A. Guffanti, Nucl. Phys. B **774**, 182 (2007). hep-ph/0607200

[14] J. Blümlein, H. Böttcher, A. Guffanti, Nucl. Phys. Proc. Suppl. **135**, 152 (2004). hep-ph/0407089

[15] M. Glück, E. Reya, C. Schuck, Nucl. Phys. B **754**, 178 (2006). hep-ph/0604116

[16] Jimenez-Delgado P, Reya E (2009). Phys. Rev. D.

[17] Jimenez-Delgado P, Reya E (2014). Phys. Rev. D.

[18] Martin AD, Stirling WJ, Thorne RS, Watt G (2009). Eur. Phys. J. C.

[19] Gao J, Guzzi M, Huston J, Lai H-L, Li Z, Nadolsky P, Pumplin J, Stump D (2014). Phys. Rev. D.

[20] Ball RD, Bertone V, Carrazza S, Deans CS, Del Debbio L, Forte S, Guffanti A, Hartland NP (2013). Nucl. Phys. B.

[21] Alekhin S, Blümlein J, Moch S (2014). Phys. Rev. D.

[22] F.D. Aaron et al., [H1 collaboration]. arXiv:1008.1731 [hep-ex]

[23] Aaron FD (2010). Eur. Phys. J. C.

[24] A. Aktas et al., [H1 collaboration]. Eur. Phys. J. C **47**, 597 (2006). hep-ex/0605016

[25] A. Aktas et al., Eur. Phys. J. C **41**, 453 (2005). hep-ex/0502010

[26] S. Chekanov et al., ZEUS collaboration. Eur. Phys. J. C **65**, 65 (2010). arXiv:0904.3487 [hep-ex]

[27] S. Chekanov et al., ZEUS collaboration. JHEP **0902**, 032 (2009). arXiv:0811.0894 [hep-ex]

[28] S. Chekanov et al., [ZEUS collaboration]. Phys. Lett. B **599**, 173 (2004). hep-ex/0405069

[29] H. Abramowicz et al., ZEUS collaboration. Eur. Phys. J. C **69**, 347 (2010). arXiv:1005.3396 [hep-ex]

[30] M. Dittmar et al. hep-ph/0511119

[31] S. Alekhin et al. hep-ph/0601012; hep-ph/0601013

[32] Z.J. Ajaltouni et al. arXiv:0903.3861 [hep-ph]

[33] D. Boer, M. Diehl, R. Milner, R. Venugopalan, W. Vogelsang, D. Kaplan, H. Montgomery, S. Vigdor et al. arXiv:1108.1713 [nucl-th]

[34] A. Accardi, J.L. Albacete, M. Anselmino, N. Armesto, E.C. Aschenauer, A. Bacchetta, D. Boer, W. Brooks et al. arXiv:1212.1701 [nucl-ex]

[35] Blümlein J, Böttcher H (2008). Phys. Lett. B.

[36] H. Kawamura, N.A. Lo Presti, S. Moch, A. Vogt, Nucl. Phys. B **864**, 399 (2012). arXiv:1205.5727 [hep-ph]

[37] M. Buza, Y. Matiounine, J. Smith, R. Migneron, W.L. van Neerven, Nucl. Phys. B **472**, 611 (1996). hep-ph/9601302

[38] J.A.M. Vermaseren, A. Vogt, S. Moch, Nucl. Phys. B **724**, 3 (2005). hep-ph/0504242

[39] I. Bierenbaum, J. Blümlein, S. Klein, Nucl. Phys. B **820**, 417 (2009). hep-ph/0904.3563

[40] Blümlein J, Klein S, Tödtli B (2009). Phys. Rev. D.

[41] M. Steinhauser, Comput. Phys. Commun. **134**, 335 (2001). hep-ph/0009029

[42] Ablinger J, Blümlein J, De Freitas A, Hasselhuhn A, von Manteuffel A, Round M, Schneider C, Wißbrock F (2014). Nucl. Phys. B.

[43] J. Ablinger, A. Behring, J. Blümlein, A. De Freitas, A. Hasselhuhn, A. von Manteuffel, M. Round, C. Schneider, F. Wißbrock, Nucl. Phys. B **886**, 733 (2014). arXiv:1406.4654 [hep-ph]

[44] M. Buza, Y. Matiounine, J. Smith, W.L. van Neerven, Eur. Phys. J. C **1**, 301 (1998). hep-ph/9612398

[45] M. Buza, Y. Matiounine, J. Smith, W.L. van Neerven, Nucl. Phys. B **485**, 420 (1997). hep-ph/9608342

[46] I. Bierenbaum, J. Blümlein, S. Klein, Nucl. Phys. B **780**, 40 (2007). hep-ph/0703285

[47] I. Bierenbaum, J. Blümlein, S. Klein, Phys. Lett. B **648**, 195 (2007). hep-ph/0702265

[48] I. Bierenbaum, J. Blümlein, S. Klein, C. Schneider, Nucl. Phys. B **803**, 1 (2008). hep-ph/0803.0273

[49] I. Bierenbaum, J. Blümlein, S. Klein, Phys. Lett. B **672**, 401 (2009). hep-ph/0901.0669

[50] J.A. Gracey, Phys. Lett. B **322**, 141 (1994). hep-ph/9401214

[51] S. Moch, J.A.M. Vermaseren, A. Vogt, Nucl. Phys. B **688**, 101 (2004). hep-ph/0403192

[52] S. Moch, J.A.M. Vermaseren, A. Vogt, Nucl. Phys. B **691**, 129 (2004). hep-ph/0404111

[53] J. Blümlein, A. De Freitas, W.L. van Neerven, S. Klein, Nucl. Phys. B **755**, 272 (2006). hep-ph/0608024

[54] J. Blümlein, W.L. van Neerven, Phys. Lett. B **450**, 417 (1999). hep-ph/9811351

[55] Ablinger J, Blümlein J, Klein S, Schneider C, Wißbrock F (2011). Nucl. Phys. B.

[56] W. Bailey, Generalized Hypergeometric Series (Cambridge University Press, Cambridge, 1935)

[57] L. Slater, Generalized Hypergeometric Functions (Cambridge University Press, Cambridge, 1966)

[58] Blümlein J (2009). Comput. Phys. Commun..

[59] J. Blümlein, in Proceeings of the Workshop *“Motives, Quantum Field Theory, and Pseudodifferential Operators”, held at the Clay Mathematics Institute, Boston University, June 2–13, 2008*, ed. by A. Carey, D. Ellwood, S. Paycha, S. Rosenberg. Clay Mathematics Proceedings, vol 12 (2010), p. 167. arXiv:0901.0837 [math-ph]

[60] J. Ablinger, J. Blümlein, C. Schneider (in preparation)

[61] J. Blümlein, S. Klein, C. Schneider, F. Stan, J. Symb. Comput. **47**, 1267 (2012). arXiv:1011.2656 [cs.SC]

[62] V.V. Bytev, M.Y. Kalmykov, B.A. Kniehl, Nucl. Phys. B **836**, 129 (2010). arXiv:0904.0214 [hep-th]

[63] C. Schneider, arXiv:1310.0160 [cs.SC]

[64] C. Schneider, J. Symb. Comput. **43**(9), 611 (2008). arXiv:0808.2543 [cs.SC]

[65] Schneider C (2005). Ann. Comb..

[66] Schneider C (2005). J. Differ. Equ. Appl..

[67] C. Schneider, Ann. Comb., **14**(4), 533 (2010). arXiv:0808.2596 [cs.SC]

[68] C. Schneider, in Proceedings of the Workshop *“Motives, Quantum Field Theory, and Pseudodifferential Operators”, held at the Clay Mathematics Institute, Boston University, June 2–13, 2008*, ed. by A. Carey, D. Ellwood, S. Paycha, S. Rosenberg. Clay Mathematics Proceedings, vol 12 (2010), p. 285. arXiv:0904.2323 [cs.SC]

[69] C. Schneider, Sém. Lothar. Combin., **56** (2006) Article B56b

[70] C. Schneider, Multi-Summation in Difference Fields, Habilitationsschrift, Johannes Kepler Universität Linz, Austria, 2007, and references therein

[71] J. Ablinger, J. Blümlein, S. Klein, C. Schneider, Nucl. Phys. Proc. Suppl., **205–206**, 110 (2010). arXiv:1006.4797 [math-ph]

[72] J. Blümlein, A. Hasselhuhn, C. Schneider, PoS (RADCOR2011) 032. arXiv:1202.4303 [math-ph]

[73] C. Schneider, in *Computer Algebra in Quantum Field Theory: Integration, Summation and Special Functions*, ed. by J. Blümlein, C. Schneider (Springer, Berlin, 2013), p. 325

[74] Schneider C (2005). Adv. Appl. Math.

[75] J. Ablinger, J. Blümlein, M. Round, C. Schneider, PoS (LL2012) 050. arXiv:1210.1685 [cs.SC]

[76] M. Round et al. (in preparation)

[77] Karr M (1981). J. ACM..

[78] M. Petkovšek, H.S. Wilf, D. Zeilberger, *A = B* (A. K. Peters, Wellesley, 1996)

[79] J.A.M. Vermaseren, Int. J. Mod. Phys. A **14**, 2037 (1999). hep-ph/9806280

[80] J. Blümlein, S. Kurth, Phys. Rev. D **60**, 014018 (1999). hep-ph/9810241

[81] J. Blümlein, Comput. Phys. Commun. **133**, 76 (2000). hep-ph/0003100

[82] J. Blümlein, S.-O. Moch, Phys. Lett. B **614**, 53 (2005). hep-ph/0503188

[83] A. V. Kotikov, V. N. Velizhanin. hep-ph/0501274

[84] S. Albino, Phys. Lett. B **674**, 41 (2009). arXiv:0902.2148 [hep-ph]

[85] A. Arbuzov, D.Y. Bardin, J. Blümlein, L. Kalinovskaya, T. Riemann, Comput. Phys. Commun. **94**, 128 (1996). hep-ph/9511434

[86] J. Blümlein, Prog. Part. Nucl. Phys. **69**, 28 (2013). arXiv:1208.6087 [hep-ph]

[87] J. Ablinger, J. Blümlein, S. Klein, C. Schneider, F. Wißbrock, arXiv:1106.5937 [hep-ph]

[88] J. Blümlein, F. Wißbrock (in preparation)

[89] M. Glück, S. Kretzer, E. Reya, Phys. Lett. B **380**, 171 (1996).[Erratum-ibid. B **405**, 391 (1997)]. hep-ph/9603304

[90] J. Blümlein, A. Hasselhuhn, P. Kovacikova, S. Moch, Phys. Lett. B **700**, 294 (2011). arXiv:1104.3449 [hep-ph]

[91] M. Buza, W.L. van Neerven, Nucl. Phys. B **500**, 301 (1997). hep-ph/9702242

[92] Blümlein J, Hasselhuhn A, Pfoh T (2014). Nucl. Phys. B.

[93] M. Glück, R. M. Godbole, E. Reya, Z. Phys. C **38**, 441 (1988). [Erratum-ibid. **39**, 590 (1988)]

[94] Ingelman G, Schuler GA (1988). Z. Phys. C.

[95] J. Ablinger. arXiv:1011.1176 [math-ph]

[96] Ablinger J, Blümlein J, Schneider C (2011). J. Math. Phys..

[97] J. Ablinger, J. Blümlein, C. Schneider, J. Math. Phys. **54**, 082301 (2013). arXiv:1302.0378 [math-ph]

[98] J. Ablinger. arXiv:1305.0687 [math-ph]

[99] J. Blümlein, Comput. Phys. Commun. **159**, 19 (2004). hep-ph/0311046

[100] Blümlein J, Kauers M, Klein S, Schneider C (2009). Comput. Phys. Commun..

[101] J. Blümlein, D.J. Broadhurst, J.A.M. Vermaseren, Comput. Phys. Commun. **181**, 582 (2010). arXiv:0907.2557 [math-ph] and references therein

[102] T. Gehrmann, E. Remiddi, Comput. Phys. Commun. **141**, 296 (2001). hep-ph/0107173

[103] D. Maitre, Comput. Phys. Commun. **174**, 222 (2006). hep-ph/0507152

[104] J. Vollinga, S. Weinzierl, Comput. Phys. Commun. **167**, 177 (2005). hep-ph/0410259

[105] J. L. Abelleira Fernandez et al. [LHeC Study Group Collaboration], J. Phys. G **39**, 075001 (2012). arXiv:1206.2913 [physics.acc-ph]

[106] Larin SA, van Ritbergen T, Vermaseren JAM (1994). Nucl. Phys. B.

[107] S.A. Larin, P. Nogueira, T. van Ritbergen, J.A.M. Vermaseren, Nucl. Phys. B **492**, 338 (1997). hep-ph/9605317

[108] A. Retey, J.A.M. Vermaseren, Nucl. Phys. B **604**, 281 (2001). hep-ph/0007294

[109] J. Blümlein, J.A.M. Vermaseren, Phys. Lett. B **606**, 130 (2005). hep-ph/0411111

